# The Dynamical Regime of Sensory Cortex: Stable Dynamics around a Single Stimulus-Tuned Attractor Account for Patterns of Noise Variability

**DOI:** 10.1016/j.neuron.2018.04.017

**Published:** 2018-05-16

**Authors:** Guillaume Hennequin, Yashar Ahmadian, Daniel B. Rubin, Máté Lengyel, Kenneth D. Miller

**Affiliations:** 1 Computational and Biological Learning Lab, Department of Engineering, University of Cambridge, Cambridge CB2 1PZ, UK; 2 Center for Theoretical Neuroscience, College of Physicians and Surgeons, Columbia University, New York, NY 10032, USA; 3 Department of Neuroscience, Swartz Program in Theoretical Neuroscience, Kavli Institute for Brain Science, College of Physicians and Surgeons, Columbia University, New York, NY 10032, USA; 4 Centre de Neurophysique, Physiologie, et Pathologie, CNRS, 75270 Paris Cedex 06, France; 5 Institute of Neuroscience, Department of Biology and Mathematics, University of Oregon, Eugene, OR 97403, USA; 6 Department of Neurology, Massachusetts General Hospital and Brigham and Women’s Hospital, Harvard Medical School, Boston, MA 02115, USA; 7 Department of Cognitive Science, Central European University, 1051 Budapest, Hungary

**Keywords:** cortical variability, circuit dynamics, noise correlations, variability quenching, V1, MT, theoretical neuroscience

## Abstract

Correlated variability in cortical activity is ubiquitously quenched following stimulus onset, in a stimulus-dependent manner. These modulations have been attributed to circuit dynamics involving either multiple stable states (“attractors”) or chaotic activity. Here we show that a qualitatively different dynamical regime, involving fluctuations about a single, stimulus-driven attractor in a loosely balanced excitatory-inhibitory network (the stochastic “stabilized supralinear network”), best explains these modulations. Given the supralinear input/output functions of cortical neurons, increased stimulus drive strengthens effective network connectivity. This shifts the balance from interactions that amplify variability to suppressive inhibitory feedback, quenching correlated variability around more strongly driven steady states. Comparing to previously published and original data analyses, we show that this mechanism, unlike previous proposals, uniquely accounts for the spatial patterns and fast temporal dynamics of variability suppression. Specifying the cortical operating regime is key to understanding the computations underlying perception.

## Highlights

• A simple network model explains stimulus-tuning of cortical variability suppression
• Inhibition stabilizes recurrently interacting neurons with supralinear I/O functions
• Stimuli strengthen inhibitory stabilization around a stable state, quenching variability
• Single-trial V1 data are compatible with this model and rules out competing proposals

## Teaser

Stimuli suppress cortical correlated variability. Hennequin et al. show that a cortical operating regime of inhibitory stabilization around a single stable state—the “stabilized supralinear network”—explains this suppression’s tuning and timing, while alternative proposed regimes do not.

## Introduction

Neuronal activity throughout cerebral cortex is variable, both temporally during epochs of stationary dynamics and across repeated trials despite constant stimulus or task conditions (Softky and Koch, 1993, Churchland et al., 2010). Moreover, variability is modulated by a variety of factors, most notably by external sensory stimuli (Churchland et al., 2010, Kohn and Smith, 2005, Ponce-Alvarez et al., 2013), planning and execution of limb movements (Churchland et al., 2006, Churchland et al., 2010), and attention (Cohen and Maunsell, 2009, Mitchell et al., 2009). Modulation of variability occurs at the level of single-neuron activity, e.g., membrane potentials or spike counts (Finn et al., 2007, Poulet and Petersen, 2008, Cardin et al., 2008, Gentet et al., 2010, Churchland et al., 2010, Tan et al., 2014), as well as in the patterns of joint activity across populations, as seen in multiunit activity or the local field potential (LFP) (Tan et al., 2014, Chen et al., 2014, Lin et al., 2015). Variability modulation shows stereotypical patterns. First, the onset of a stimulus quenches variability overall and, in particular, correlated variability in firing rates that is “shared” across many neurons (Lin et al., 2015, Goris et al., 2014, Ecker et al., 2014, Ecker et al., 2016, Churchland et al., 2010). Moreover, the degree of variability reduction can depend systematically on the tuning of individual cells. For example, in area MT, variability is quenched more strongly in cells that respond best to the stimulus, and correlations decrease more among neurons with similar stimulus preferences (Ponce-Alvarez et al., 2013, Lombardo et al., 2015). Although these patterned modulations of variability are increasingly included in quantitative analyses of neural recordings (Renart and Machens, 2014, Orbán et al., 2016), it is still unclear what they imply about the dynamical regime in which the cortex operates.

**Figure 1:**
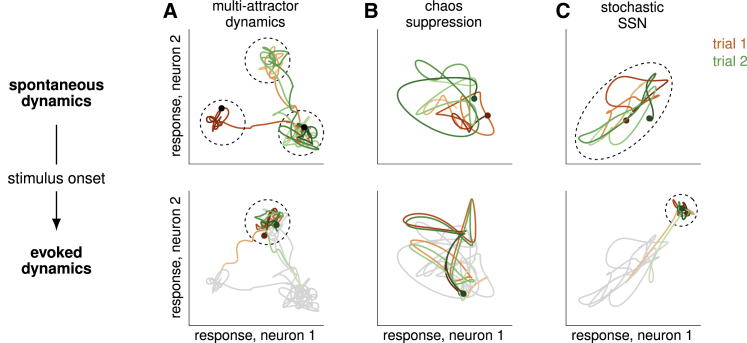
Three Different Dynamical Regimes that Could Explain Variability Modulation by Stimuli (A–C) Two schematic neural trajectories (red and green) corresponding to two separate trials are plotted for each dynamical regime, before (top) and after (bottom) stimulus onset. Spontaneous activity is redrawn in gray beneath evoked activity to allow comparison of variability. Dotted ellipses outline activity covariances around the fixed point(s) of the dynamics (if any exist). (A) Multi-attractor dynamics: spontaneous activity wanders stochastically between a set of attractor states (three shown), resulting in large trial-by-trial variability (top). Stimulus onset constrains fluctuations to the vicinity of a single attractor, reducing variability across both time and trials (bottom). (B) Chaos suppression: chaos yields large across-trial variability in spontaneous dynamics (top), which is suppressed by the stimulus, leading to a reduction of variability across trials but not necessarily across time (bottom). (C) Stochastic SSN: both spontaneous and evoked dynamics are stable with a single fixed point, but the stimulus can shrink the effective size of the basin of attraction of the fixed point (as well as shifting its location), resulting in a reduction of both across-time and across-trial variability.

There have been two dynamical mechanisms proposed to explain selected aspects of the modulation of cortical variability by stimuli. In “multi-attractor” models, the network operates in a multi-stable regime in the absence of a stimulus, such that it noisily wanders among multiple possible stable states (“attractors”). This wandering among attractors occurs in a concerted way across the population, resulting in substantial shared variability (Figure 1A, top). Stimuli then suppress this shared variability by pinning fluctuations to the vicinity of one particular attractor (Figure 1A, bottom; Blumenfeld et al., 2006, Litwin-Kumar and Doiron, 2012, Deco and Hugues, 2012, Burak and Fiete, 2012, Ponce-Alvarez et al., 2013, Doiron and Litwin-Kumar, 2014, Mochol et al., 2015). In chaotic network models (Sompolinsky et al., 1988), firing rates exhibit strong chaotic fluctuations, and certain types of stimuli can suppress chaos by forcing the dynamical state of the network to follow a specific trajectory, thus quenching across-trial variability (Figure 1B; Molgedey et al., 1992, Bertschinger and Natschläger, 2004, Sussillo and Abbott, 2009, Rajan et al., 2010). While both the multi-attractor and the chaotic mechanisms can explain the general phenomenon of stimulus-induced reduction of variability, only the former has been proposed to explain the stimulus-tuning of variability reduction. However, even in that case, a considerable fine-tuning of parameters or very strong noise was needed to keep the network in the regime with multiple attractors, such that the system stays near attractors, yet noise can move the system between them (Ponce-Alvarez et al., 2013).

Here, we explore a qualitatively different regime of cortical dynamics. We describe activity fluctuations as being driven by noise but shaped by nonlinear, recurrent interactions. In contrast to previous models, our network operates around a single stable point that depends on the stimulus (Figure 1C). Crucially, individual neurons have supralinear (expansive) input/output functions. This causes the gains of neurons, and thus the effective synaptic strengths in the network, to increase with network activation. This is a stochastic generalization of the stabilized supralinear network (SSN) model that has successfully accounted for a range of phenomena related to the stimulus dependence of trial-averaged responses in visual cortex (Ahmadian et al., 2013, Rubin et al., 2015). Introducing stochasticity allows us to model the variability of responses and thus use data on neural variability to identify hallmarks of this regime and distinguish it from previous proposals.

In our network, stimulus-dependent changes in effective connectivity shape the magnitude and structure of activity fluctuations in the network. Specifically, stimuli change the balance of two opposing effects of recurrent network dynamics on variability: hidden feedforward interactions (“balanced amplification”; Murphy and Miller, 2009, Hennequin et al., 2014) and recurrent excitation, which amplify variability and dominate for very weak (spontaneous) inputs; and stabilizing inhibitory feedback, which quenches variability (Renart et al., 2010, Tetzlaff et al., 2012) and dominates for stronger inputs.

By studying this network mechanism in a progression of recurrent architectures with increasingly detailed structure, we find that it naturally and robustly explains the modulation of shared cortical variability by stimuli, including its tuning dependence. We first analyze variability in the simplest instantiation of the model, with two unstructured populations of excitatory (E) and inhibitory (I) cells, and find that an external stimulus can strongly modulate the variability of population activities. In particular, the model predicts stimulus-induced quenching of variability, as well as a reduction of the low-temporal-frequency coherence between local population activity and single-cell responses, as found experimentally (Poulet and Petersen, 2008, Churchland et al., 2010, Chen et al., 2014, Tan et al., 2014). Next, we extend our analysis to a more detailed architecture with structured connectivity to account for the tuning-dependent modulations of Fano factors and noise correlations by stimuli. Critically, these results reveal robust qualitative differences between the predictions of our model and those of previously proposed network mechanisms, based on multi-attractor or chaotic dynamics, for both the spatial patterns and temporal dynamics of variability suppression. We tested these predictions against experimental data and found the SSN model to be the most consistent with previously analyzed data from primary visual cortex (V1) and MT (Churchland et al., 2010, Ponce-Alvarez et al., 2013) as well as with our novel analyses of published V1 recordings in the awake monkey (Ecker et al., 2010). Such comparisons of different models are crucial for guiding future experiments that can make targeted measurements to fully resolve the dynamical regime in which the cortex operates—a key first step in identifying the computational strategies underlying perception.

## Results

**Figure 2:**
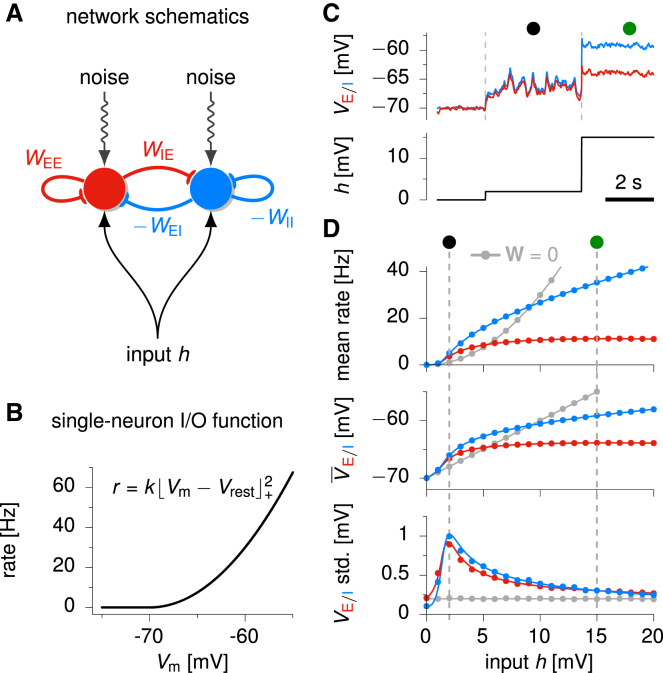
Activity Variability in a Reduced, Two-Population Stochastic SSN (A) The network is composed of two recurrently connected units, summarizing the activity of two populations of excitatory (red) and inhibitory (blue) neurons. Both units receive private input noise and a common constant input *h*. (B) Threshold-quadratic neural input/output function determining the relationship between membrane potential and momentary firing rate of model neurons (Equation 2). (C) Sample $V_{E/I}$ traces for the two units (top), as the input is increased in steps from h=0 to 2 mV to 15 mV (bottom). (D) Dependence of population activity statistics on stimulus strength *h*. Top: mean E (red) and I (blue) firing rates; middle: mean $V_{E/I}$; bottom: standard deviation of $V_{E/I}$ fluctuations. The comparison with a purely feedforward network (W=0) receiving the same input *h* is shown in gray. Dots are based on numerical simulations of 500 trials. Solid lines show analytical approximations (Hennequin and Lengyel, 2016).

We used a standard model to study the dynamical evolution of momentary firing rates in a recurrently coupled network of excitatory and inhibitory neurons (Figure 2A; Dayan and Abbott, 2001; see also STAR Methods). In this model, neurons integrate their external and recurrent inputs linearly in their membrane potentials, $V_{\text{m}}$, but their output firing rates, *r*, are a nonlinear function of the voltage: $r=f\left(V_{\text{m}}\right)$ (Figure 2B). Crucially, we studied variants of this model in which the nonlinearity *f* is an expansive (supralinear) function (Figure 2B) and in which inhibition was both sufficiently fast and strong and appropriately structured to stabilize the network in the face of recurrent excitation and the supralinear input/output function. This is the stabilized supralinear network (SSN) model (Ahmadian et al., 2013). In order to study response variability, we added to this model a stochastic component (slow noise) in the membrane potential dynamics of all cells. Stabilization meant that the network operated around a single steady state, albeit a stimulus-dependent one.

Real neurons, of course, have an input/output function that ultimately saturates. We focus on an expansive, non-saturating input/output function because V1 cortical neurons show such a relationship between mean voltage and firing rate across their full dynamic range, without saturation even for the strongest visual stimuli (Priebe and Ferster, 2008). Thus, saturation does not appear to play a role in stabilizing cortical activity, a fact that we capture by using a non-saturating input/output function. Such an expansive input/output function arises in spiking neurons when their firing is driven by voltage fluctuations, with the mean voltage sub- or peri-threshold (Hansel and van Vreeswijk, 2002, Miller and Troyer, 2002), a firing regime that produces the highly variable spiking seen in cortical neurons (Troyer and Miller, 1997, Amit and Brunel, 1997). We assume that the voltage fluctuations giving rise to the expansive input/output function are fast compared to the timescales of variability studied here and do not explicitly model them.

We focused on analyzing how the intrinsic dynamics of the network shaped fixed input noise to give rise to stimulus-dependent patterns of response variability. We studied a progression of connectivity architectures of increasing complexity, all involving two separate populations of excitatory and inhibitory neurons. We also validated our results in large-scale simulations of spiking neuronal networks.

### Variability of Population Activity: Modulation by External Input

We first considered a simple circuit motif: an excitatory (E) unit and an inhibitory (I) unit, recurrently coupled and receiving the same mean external input *h* as well as their own independent noise (Figure 2A). In this reduced model, the two units represent two randomly connected populations of E and I neurons, a canonical model of cortical networks (Amit and Brunel, 1997, Vogels et al., 2005). Thus, their time-varying activity, $V_{E}\left(t\right)$ and $V_{I}\left(t\right)$, represents the momentary population-average membrane potential of all the E and I cells, respectively. Despite its simplicity, this architecture accounted well for the overall population response properties in the larger networks, with more detailed connectivity patterns, that we analyzed later.

Activity in the network exhibited temporal variability due to the stochastic component of the dynamics. We found that this (correlated) variability of $V_{E}$ and $V_{I}$ fluctuations, together with their means, ${\bar{V}}_{E/I}$, was strongly modulated by the external steady input *h* (Figures 2C and 2D). When h=0, there was no input to drive the network, and $V_{E}$ and $V_{I}$ both hovered around $V_{\text{rest}}=-70$ mV, fluctuating virtually independently, with standard deviations essentially matching those that would arise without recurrent connections (gray line in Figure 2D, bottom). For a somewhat larger input, h=2 mV, both E and I populations fired at moderate rates (3–4 Hz) (Figure 2D, top), but now also exhibited large and synchronous population $V_{\text{m}}$ fluctuations (Figure 2C, black circle mark). For yet larger inputs (h=15 mV), fluctuations remained highly correlated, but their magnitude was strongly quenched (Figure 2C, green circle mark).

Figure 2D shows how the temporal (or, equivalently, the across-trial) mean and variability of activities varied over a broad range of input strengths. We observed that population mean $V_{\text{m}}$ increased monotonically with growing external input, first linearly or supralinearly for small inputs, but strongly sublinearly for larger inputs, with ${\bar{V}}_{I}$ growing faster than ${\bar{V}}_{E}$ (Figure 2D, middle; Ahmadian et al., 2013, Rubin et al., 2015). In contrast, variability in both $V_{E}$ and $V_{I}$ typically increased for small inputs, peaking around this transition between supralinear and sublinear growth, and then decreased with increasing input (Figure 2D, bottom). Importantly, input modulation of variability required recurrent network interactions. This was revealed by comparing our network to a purely feedforward circuit that exhibited qualitatively different behavior (Figure 2D, gray). In the feedforward circuit, mean $V_{\text{m}}$ remained linear in *h*, so that mean rates rose quadratically with $V_{\text{m}}$ or *h* (reflecting the input/output nonlinearity; Figure 2B), and fluctuations in $V_{\text{m}}$ no longer depended on the input strength.

### Variability Suppression with a Single Stable State Is a Robust Phenomenon

In order to demonstrate that the overall dynamical regime of the stabilized supralinear network, rather than just a particular instantiation of our model, underlies variability modulation, we used a combination of numerical simulations and analytical results to confirm the robustness of our findings.

We simulated 1,000 model networks with random parameter values within wide brackets. We found that variability suppression was robust over a broad range of network parameters (connection weights, input strengths and correlations, and the exponent and scale of the firing-rate nonlinearity), as long as they ensured dynamical stability even for strong inputs (Figures S1 and S2). Although the precise amplitude and position of the peak of $V_{\text{m}}$ variance depended on network parameters, the overall non-monotonic shape of variability modulation was largely conserved. In particular, we could show analytically that variability suppression occurs earlier (for smaller input *h*) in networks with strong connections or, for fixed overall connection strength, in networks that are more dominated by feedback inhibition (Methods S3). More generally, we found that the firing rates at the peak of variability are typically low (2.5 Hz on average over a thousand randomly parameterized stable networks and below 6 Hz for 90% of them; *cf.* Methods S2). As these rates are comparable to cortical spontaneous firing rates, this predicts that increased sensory drive should generally result in variability quenching in cortical LFPs.

In order to better understand the robustness of variability suppression in the model, we took advantage of the fact that our network was characterized by a single attractor at each level of the input, *h*, and analyzed the dynamics of small activity fluctuations, δV, around this stable state (such that $V=\bar{V}\left(h\right)+\delta V$, where $\bar{V}\left(h\right)$ is the mean activity in the stable state; STAR Methods). These dynamics are governed by a set of effective connection weights, $W^{\text{eff}}$, that quantify the impact of a small momentary change in the $V_{\text{m}}$ of the presynaptic neuron on the total input to its postsynaptic partner. The dependence of the effective connection weights on the stable state and thus on the external input, *h*, that determines the stable state is simply given by:

(Equation 1) $$W_{ij}^{\text{eff}}\left(h\right)\propto W_{ij}\;f^{\text{'}}\;\left[{\bar{V}}_{j}\left(h\right)\right]$$

where $W_{ij}$ is the strength of the “biophysical” connection from unit *j* to unit *i*, and $f^{'}$ is the slope of the single-neuron firing-rate nonlinearity at the stable state. Importantly, $f^{'}$ increases with increasing $\bar{V}\left(h\right)$, because *f* is an expansive, convex nonlinearity (Figure 2B). Thus, in general, effective connectivity increases with increasing *h*, reflecting the growth of $\bar{V}\left(h\right)$ (Figure 2D, middle).

An increase in effective connectivity can have conflicting effects: it can increase excitatory or driving effects that amplify fluctuations and increase variability (Murphy and Miller, 2009, Hennequin et al., 2014), but it can also increase inhibitory feedback, suppressing fluctuations and decreasing variability (Renart et al., 2010, Tetzlaff et al., 2012). Thus, understanding how changes in effective connectivity translate into changes in variability required further analysis (Methods S3). We found that the net behavior of the network indeed included a combination of both effects (Figure S3). As the input grew from zero, variability first rapidly increased, due primarily to the growth of effective feedforward weights (“balanced amplification”; Murphy and Miller, 2009) but also of recurrent excitatory loops. Then, beginning at firing rates comparable to spontaneous activity as described above, variability steadily decreased with increasing stimulus strength due to increasingly strong inhibitory feedback (Figure 2D, bottom).

Crucially, we were able to show analytically that variability quenching effects must ultimately dominate, leading to progressively stronger quenching of variability as the input increases. This is due to the faster growth of I activity relative to E activity in the network, which is a robust outcome of dynamic stabilization by feedback inhibition (Figure S1; Ahmadian et al., 2013, Rubin et al., 2015) and which has been observed in rodent S1 (Shao et al., 2013) and V1 (Adesnik, 2017). We also found that ignoring the variability-increasing effects, which are characteristic of excitatory-inhibitory dynamics (Kriener et al., 2008, Murphy and Miller, 2009) and thus largely absent from models that do not include separate excitatory and inhibitory populations, can fail to capture the full extent of variability modulation and lead to an underestimation of the level of spontaneous variability obtained at zero-to-weak input levels (Figure S4).

### Variability Quenching and Synchronization in Single Neurons

**Figure 3:**
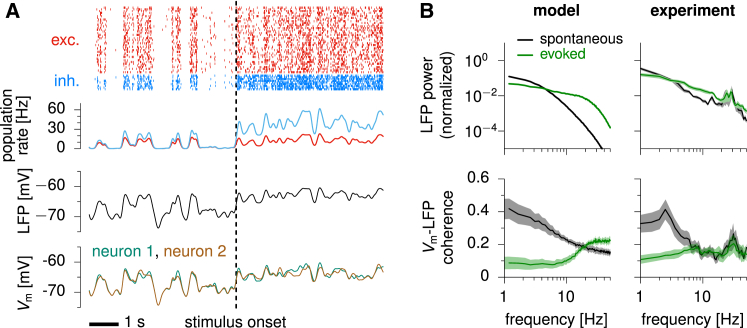
Modulation of Variability in a Randomly Connected Stochastic Spiking SSN (A) Top: raster plot of spiking activity, for 40 (out of 4,000) excitatory neurons (red) and 10 (out of 1,000) inhibitory neurons (blue). Upper middle: momentary E and I population firing rates. Lower middle: LFP (momentary population-averaged $V_{\text{m}}$). Bottom: $V_{\text{m}}$ of two randomly chosen excitatory neurons. The dashed vertical line marks the onset of stimulus, when *h* switches from 2 mV to 15 mV. Population firing rates, LFP, and $V_{\text{m}}$ traces were smoothed with a Gaussian kernel of 50 ms width. (B) Top, normalized LFP power in spontaneous (black) and evoked (green) conditions; bottom, average (± SEM) spectral coherence between single-cell $V_{\text{m}}$ and the LFP; left, model; right, data from V1 of the awake monkey, reproduced from Tan et al. (2014).

In order to study variability in single neurons and at the level of spike counts, we implemented the two-population architecture of Figure 2A in a network of spiking neurons (Figure 3; STAR Methods). The network consisted of 4,000 E neurons and 1,000 I neurons, randomly connected with low probability and with synaptic weights chosen such that the overall connectivity matched that of the reduced model. Each neuron emitted action potentials stochastically with an instantaneous rate given by Equation 3 (this additional stochasticity accounted for the effects of unmodelled fluctuations in synaptic inputs that occur on timescales faster than the 30 ms effective time resolution of our model; Methods S4). The external input to the network again included a constant term, *h*, and a noise term that was temporally correlated on a 50 ms timescale with uniform spatial correlations of strength 0.2.

At the population level, the network behaved as predicted by the reduced model. Neurons fired irregularly (Figure 3A, top), with firing rates that grew superlinearly with small input *h* but sublinearly with stronger input (Figure S5). Moreover, fluctuations in E and I population activities were strongly synchronized (Figure 3A, upper middle), and LFP variability decreased with increasing *h* (Figure 3A, lower middle). Importantly, variability quenching also occurred at the level of individual neurons’ $V_{\text{m}}$, accompanied by a reduction of pairwise correlations (Figure 3A, bottom; these required that single neurons shared part of their input noise; Methods S3).

The model primarily suppressed shared rather than private (to individual neurons) variability (Figure S5), as in experiments (Churchland et al., 2010). This was because the spatially uniform average connectivity of the network meant that its dynamics were only significantly coupled to patterns of uniform activity across E or across I cells. These patterns were thus the ones affected by stimulus-induced changes in effective connectivity (Figure S3). Correlated noise drove such uniform patterns so that they carried significant variability. Thus, these shared excitatory and inhibitory activity patterns behaved as the activity of the individual units of the previous reduced two-population model, and so variability suppression in the reduced model implied the suppression specifically of shared variability in this more detailed model.

Our model also accounted for the stimulus-induced modulation of the power spectrum and cross-coherence of LFP and single-cell $V_{\text{m}}$ fluctuations, as observed in V1 of the awake monkey (Figure 3B; Tan et al., 2014). Strong external input reduced the LFP power at low frequencies, due to enhanced effects of feedback inhibition; increased it at intermediate frequencies, due to the faster timescales associated with relatively enhanced inhibition; and also increased it at high frequencies, due to the larger firing rates, which contributed additional, high-frequency fluctuations in synaptic drive (Figure 3B, top left). This asymmetric modulation of LFP power at low and high frequencies is also seen in experiments (Figure 3B, top right). Moreover, as increasing inputs suppressed variability at the population level, the private noise in the activity of each neuron had a proportionately larger contribution to its overall variability, leading to a drop in pairwise correlations (Figure 3A) and $V_{\text{m}}$-LFP coherence specifically at low frequencies where the suppression of population variability occurred, as seen in experiments (Figure 3B, bottom).

### Stimulus-Tuning of Variability Suppression in V1

Neuronal recordings in visual areas have shown that Fano factors drop at the onset of the stimulus (drifting gratings or plaids) in almost every neuron, which was well accounted for by the randomly connected network we studied above. However, in the experiments, variability did not drop uniformly across cells, but exhibited systematic dependencies on stimulus tuning (Ponce-Alvarez et al., 2013, Lombardo et al., 2015, Lin et al., 2015). This could not be explained by randomly connected architectures, so we extended our model to include tuning dependence in connectivity and input noise correlations.

**Figure 4:**
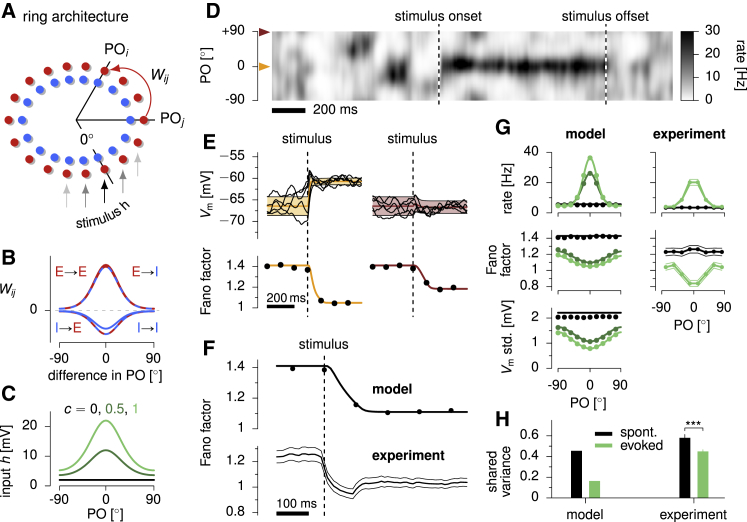
Modulation of Variability in a Stochastic SSN with a Ring Architecture (A) Schematics of the ring architecture. Excitatory (red) and inhibitory neurons (blue) are arranged on a ring, their angular position indicating their preferred stimulus (expressed here as preferred stimulus orientation, PO). The stimulus is presented at $0^{\circ }$. (B) Synaptic connectivities all follow the same circular Gaussian profiles with peak strengths that depend on the type of pre- and post-synaptic populations (excitatory, E, or inhibitory, I). (C) Each neuron receives a constant input with a baseline (black line, 0% contrast), which drives spontaneous activity, and a tuned component with a bell-shaped dependence on the neuron’s preferred orientation and proportional to contrast, *c* (dark and light green, 50% and 100% contrast, respectively). Neurons also receive spatially and temporally correlated noise, with spatial correlations that decrease with tuning difference (see Figure 5D). (D) Single-trial network activity (E cells), before and after the onset of the stimulus (100% contrast). Neurons are arranged on the y axis according to their preferred stimuli. (E) Reduction in membrane potential variability across trials: membrane potential traces in 5 independent trials (top) and Fano factors (bottom) for an E cell tuned to the stimulus orientation (left) or tuned to the orthogonal orientation (right). For $V_{m}$, orange and brown lines and shading show (analytical approximation of) across-trial mean ± SD. (F) Reduction of average spike count Fano factor in the population following stimulus onset in the model (top) and experimental data (bottom). Spikes were counted in 100 ms time windows centered on the corresponding time points. (G) Mean firing rates (top), Fano factors (middle), and std. of voltage fluctuations (bottom) at different contrast levels as a function of the neuron’s preferred stimulus in the model (left) and, for rate and Fano factor, experimental data (right, averaged across 99 neurons). Colors indicate different contrast levels (model: colors as in C; data: black, spontaneous, green, 100% contrast). (H) Shared variability in normalized spike counts, as estimated via factor analysis (STAR Methods; Churchland et al., 2010), before (spontaneous, black) and after stimulus onset (evoked, green) in the model (left) and experimental data (right). Dots in (F) and (G) are based on numerical simulations of 500 trials. For the model, colored lines and shaded areas in (E) and solid lines in (F) and (G) show analytical approximations (Hennequin and Lengyel, 2016). Experimental data analyzed in (F)–(H) are from awake monkey V1 (Ecker et al., 2010), with error bars denoting 95% CI.

We studied an architecture in which the preferred stimulus of E/I neuron pairs varied systematically around a “ring” representing an angular stimulus variable, such as stimulus orientation in V1 or motion direction in MT (Figure 4A; STAR Methods). We describe the case in which the variable is orientation, which ranges from 0 to $180^{\text{o}}$; identical results describe direction if all angles are doubled. The average input to a cell (either E or I) was composed of a constant baseline, which drove spontaneous activity in the network, and a term that depended on the angular distance between the stimulus orientation and the preferred orientation (PO) of the cell, and that scaled with image contrast, *c* (Figure 4C). Input noise correlations depended on tuning differences (STAR Methods): cells with more similar tuning received more strongly correlated inputs. The strength of recurrent connections depended on the difference in preferred orientation between pre- and postsynaptic neurons and whether they were excitatory or inhibitory (Figure 4B).

The bump of stimulus-driven input drove a similar, but narrower, bump of network response (Figures 4D and 4G). Although this architecture appears similar to a form of multi-attractor model that has a continuum of attractors—a bump of activity that (in the absence of stimuli) can be centered at any location (the so-called “ring attractor model”; Goldberg et al., 2004, Ben-Yishai et al., 1995, Ponce-Alvarez et al., 2013)—our model was actually quite different. While multi-attractor networks show a bump of sustained activity even once the stimulus is removed (leaving only non-specific background excitation), in our network the bump of activity depends on the similar bump of stimulus-driven input. When the stimulus is removed, our network returns within a single membrane time constant to a homogeneous level of baseline activity, driven by the homogeneous baseline input (Figure 4D). As we show below, this dynamical regime is also characterized by fundamentally different patterns of response variability than multi-attractor dynamics.

We applied this model to study the stimulus dependence of variability quenching in V1 and compared our results to a new analysis we performed of previously published recordings in V1 of the awake monkey (Ecker et al., 2010). In the absence of visual input (0% contrast), the network exhibited spatially patterned fluctuations in momentary firing rates around a few Hz (Figure 4D) with large across-trial variability in single-cell $V_{\text{m}}$ (Figure 4E). In evoked conditions, the input drove a hill of network activity around the stimulus orientation as in the data (Figures 4D and 4G), resulting in approximately contrast-invariant tuning curves (Priebe and Ferster, 2008). At stimulus offset, activity rapidly decayed back to spontaneous levels with the cellular time constant (Figure 4D), as observed in cortex when thalamic input is silenced (Reinhold et al., 2015, Guo et al., 2017).

The fluctuating firing rates in spontaneous activity implied super-Poisson variability in spike counts—Fano factors greater than 1 (Figure 4F, top) —given the stochastic spiking mechanism described above (Figure 3). This was consistent with the high level of spontaneous variability in the data (Figure 4F, bottom). Both the model and the data exhibited a pronounced drop in Fano factor following stimulus onset (Figure 4F) and displayed a U-shaped tuning of variability suppression with stimulus orientation (Figure 4G, middle), such that variability suppression was stronger for cells whose preferred orientation was close to the stimulus. The model made similar predictions for variability in membrane potentials: a U-shaped profile of $V_{\text{m}}$ variance suppression in stimulus-evoked conditions relative to spontaneous fluctuations (Figure 4G, bottom).

Notably, for similar reasons as in the randomly connected network (Figure 3; Figure S5), it was primarily the shared and not the private part of variability that was quenched by stimuli in the model (Figure 4H, left), and this required some degree of spatial correlations in the input noise (Figure S6). This was because the spatially smooth nature of the connectivity meant that only spatially smooth patterns of activity were strongly coupled to the network dynamics. A substantial suppression of shared variability at stimulus onset has been observed across many cortical areas (Churchland et al., 2010) as well as in our analysis of the V1 data (Figure 4H, right; Ecker et al., 2010; see also Lombardo et al., 2015).

We again explored a broad range of parameters to show that the tuning of variability suppression was a robust outcome of the model. We found that Fano factor and $V_{\text{m}}$ variance were always most strongly suppressed in the neurons that were most strongly driven by the stimulus (the “dip” of the U shape) consistent with the V1 data (see above). Interestingly, there were some cases when neurons tuned to the opposite stimulus also showed a strong reduction of Fano factor (though not of membrane potential variance; Figure S7)—consistent with recent findings of an M-shaped modulation of Fano factors (and spike count correlations of similarly tuned cells) in area MT of the awake macaque (Figure S7; Ponce-Alvarez et al., 2013). However, while such an M-shaped modulation was previously attributed to marginally stable multi-attractor dynamics (Ben-Yishai et al., 1995, Ponce-Alvarez et al., 2013), our model still produced this with a single stable attractor: the spike count variability of oppositely tuned cells dropped when input tuning in the model was as narrow as, or narrower than, the tuning of recurrent connections. In this configuration, oppositely tuned cells received so small a net input on average that their membrane potential fluctuations barely crossed the threshold of the firing rate nonlinearity, thus producing very little spiking variability. In turn, this loss of firing rate variance even overcame the effect of dividing by very small firing rates in computing Fano factors for these neurons. Under the same conditions, a similar M shape was apparent for spike count correlations between similarly tuned neurons, as a function of their (common) preferred orientation (Figure S7).

### Patterns of Noise Variability Arise from Low-Dimensional Bump Kinetics

**Figure 5:**
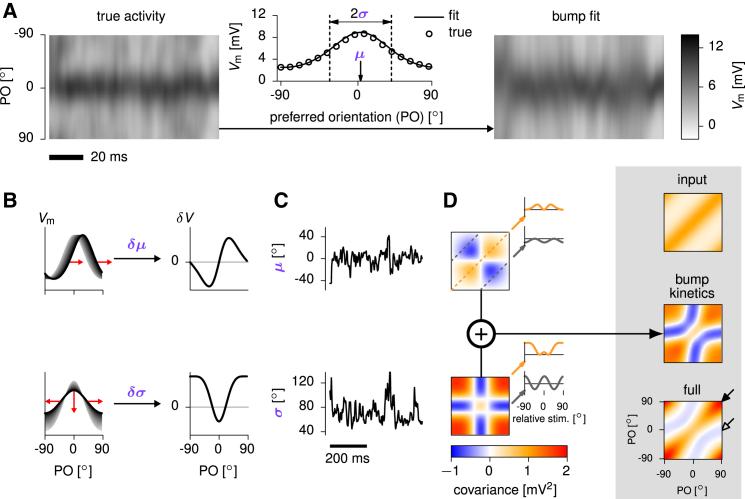
Low-Dimensional Bump Kinetics Explain Noise Variability in the Ring SSN (A) Sample of $V_{\text{m}}$ fluctuations across the network in the evoked condition (left, “true activity,” 100% contrast), to which we fitted a circular-Gaussian function (bump) $V_{i}\left(t\right)=a\left(t\right)\;\text{exp}\left[\left(cos\left(\theta_{i}-\mu \left(t\right)\right)-1\right)/\sigma^{2}\left(t\right)\right]$ across the excitatory population in each time step (center), parametrized by its location, μ, and width, σ. The amplitude of the bump, *a*, was chosen in each time step so as to keep total population firing rate constant. Fluctuations in location and width were independent, and the fit captured 87% of the variability in $V_{\text{m}}$ (right). (B) The two principal modes of bump kinetics: small changes (red arrows) in location (top) and width (bottom) of the activity bump result in the hill of network activity deviating from the prototypical bump (gray shadings). Plots on the right show how the activity of each neuron changes due to these modes of bump kinetics. (C) Time series of μ and σ extracted from the fit. (D) Ongoing fluctuations in each bump parameter contribute a template matrix of $V_{\text{m}}$ covariances (color maps show covariances between cells with preferred orientation [PO] indicated on the axes of the “full” matrix, bottom right), obtained from (the outer product of) the differential patterns on the right of (B). Insets show $V_{\text{m}}$ covariance implied by each template for pairs of identically tuned cells (orange, PO difference $≃0^{\circ }$) and orthogonally tuned cells (gray, PO difference $=90^{\circ }$), as a function of stimulus orientation relative to the average PO of the two cells. The two templates sum up to a total covariance matrix (“bump kinetics”), which captures the key qualitative features of the full $V_{\text{m}}$ covariance matrix (“full”). The covariance matrix of the input noise (“input”) is also shown above for reference. The stimulus is at $0^{\circ }$ throughout.

Next, we analyzed the origin and mechanism of the stimulus-tuning of noise variability in the ring architecture. As mentioned above, for a fixed stimulus, the most prominent feature of population activity was a “bump” of high $V_{\text{m}}$ in the cells with preferred orientations near the stimulus orientation and a lower baseline of activity in the surround (Figure 5A, left and middle). In general, variability in the bump and the baseline captured most of the network’s variance and its suppression with increasing stimulus strength (Figure S8). Here and in the next section we specifically focus on the structure of the quenched noise variability after stimulus onset.

After stimulus onset, most of the shared variability (87%; Figure S8) arose from variability in the location, μ, and width, σ, of the bump of activity (Figure 5A, middle and right). Notably, fluctuations in bump amplitude and width scaled inversely with one another, as the nonlinear interactions among neurons in our network resulted in strong normalization (Ahmadian et al., 2013, Rubin et al., 2015), preserving overall activity. Each of these small transformations resulted in a characteristic pattern of momentary deviation of network activity from the mean bump (Figure 5B). In turn, these two patterns of momentary fluctuations (Figure 5C) contributed two distinct spatial covariance templates (Figure 5D). For example, sideways motion of the bump increased the firing rates of all the cells with preferred orientations on one side of the stimulus orientation and decreased firing rates for all cells on the other side (Figure 5B, top). This resulted in positive covariances between cells with preferred orientations on the same side of the stimulus orientation and negative covariances for cells on opposite sides (Figure 5D, top: μ-template; Moreno-Bote et al., 2014). Conversely, an increase in bump width (and thus a decrease in amplitude) increased the activities of cells on the flanks of the bump, tuned away from the stimulus, while decreasing the activity of cells near the peak, tuned for the stimulus (Figure 5B, bottom). This generated positive covariances within each of these groups and negative covariances between the two groups (Figure 5D, bottom: σ-template).

Taken together, the ongoing jitter in bump location and width contributed a highly structured pattern of response covariances, which accounted for most of the structure in the full covariance matrix of the network (Figure 5D, compare “bump kinetics” with “full”). In particular, bump kinetics correctly predicted the $V_{\text{m}}$ variances of cells (given by the diagonal of the full covariance matrix indicated by the filled arrow in Figure 5D), showing less variance for cells tuned to the stimulus orientation of $0^{\text{o}}$ than for cells tuned to orthogonal orientations (see Figure 4G, bottom, green), and hence explained the U-shaped modulation of Fano factors (Figure 4G, middle, green). Moreover, the recurrent dynamics generated negative correlations in the $V_{\text{m}}$ fluctuations of cells with orthogonal tuning, despite such pairs receiving positively correlated inputs (Figure 5D, “input” versus “bump kinetics,” secondary diagonal with open arrow).

### Experimental Predictions: Stimulus Tuning

For a direct comparison of the dynamical regime of the SSN with previously proposed mechanisms for variability modulation, based on marginally stable or chaotic dynamics, we first studied the predictions of the models for the spatial patterns of spike count noise correlations. Chaotic models have not (Rajan et al., 2010), and probably can not, predict the tuning of mean responses, let alone that of variability suppression, so we focused on a comparison with a multi-attractor ring model. This model has been suggested to account for stimulus-modulated changes in variability in area MT (Ponce-Alvarez et al., 2013). We matched it to our model such that it produced similar tuning curves and overall levels of variability (Figure S9).

**Figure 6:**
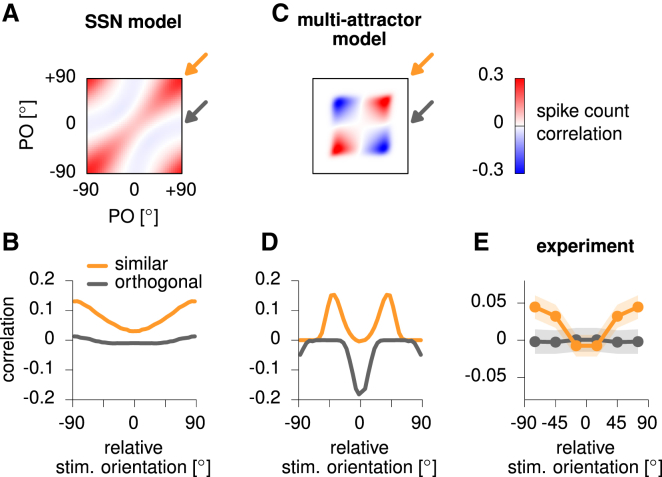
Stimulus Tuning of Spike Count Correlations in the Ring SSN versus the Multi-attractor Ring Model (A) Spike count correlation matrix in the ring SSN during evoked activity (100% contrast). Color map shows correlations between cells with preferred orientation (PO) indicated on the axes, relative to stimulus orientation at $0^{\circ }$. Arrows indicate axes along which cell pairs are similarly (orange) or orthogonally tuned (gray). Spike count correlations along the diagonal show correlation for identically tuned cells, rather than for identical cells, and are thus less than one due to private spiking noise. (B) Average spike count correlations in the SSN, for pairs of similarly tuned cells (orange, PO difference less than $45^{\circ }$) and orthogonally tuned cells (gray, PO difference greater than $45^{\circ }$), as a function of stimulus orientation relative to the average PO of the two cells. (C and D) Same as (A) and (B), for the multi-attractor ring network. (E) Same as (B) and (D), for data from awake monkey V1 (Ecker et al., 2010). Data were symmetrized for negative and positive stimulus orientations. Shaded regions denote 95% CI. SSN simulations in this figure used the same parameters as in Figures 4 and 5.

While there were several differences apparent in the detailed correlations predicted by the two models (Figures 6A and 6C), many of these could be explained away by trivial factors that neither model captured fully. For example, the average correlation was substantially larger in the SSN than in the attractor network—but this difference could be eliminated by invoking, in the attractor model, an additional (potentially extrinsic) mechanism that adds a single source of shared variability across neurons, resulting in a uniform (possibly stimulus strength-dependent) positive offset to all correlations (Lin et al., 2015). As another example, the attractor network always exhibited an M-shaped modulation of correlations, whereas, just as for Fano factors (see above), the SSN mostly showed a U-shaped modulation but could show an M shape for particular parameters (Figure S7).

Therefore, we focused on distinctions that were robust to model details and followed from a fundamental difference of bump kinetics in the two models: in contrast to the richer patterns of variability generated by the SSN, multi-attractor dynamics showed a more limited repertoire, dominated by sideways motion of the bump with barely any fluctuations in bump width (Figure S9; Burak and Fiete, 2012). As fluctuations in bump location and width had opposite effects on the correlations between orthogonally tuned cells in the SSN model (Figure 5D insets, gray), their cancellation made these correlations only very weakly modulated by the stimulus (Figure 6A, gray arrow; Figure 6B, gray). In particular, this modulation was much shallower than that for similarly tuned cells (Figure 6A, orange arrow; Figure 6B, orange), for which variability in bump location and width had congruent effects (Figure 5D insets, orange) that added to rather than cancelled each other. In contrast, in the attractor model, there was no such cancellation even for orthogonally tuned cells due to the absence of fluctuations in bump width (Figure S9). This meant that correlations between orthogonally tuned cells were just as deeply modulated as those between similarly tuned cells (Figures 6C and 6D).

Previous reports on the stimulus-tuning of noise correlations examined only similarly tuned cells and reported mostly M-shaped modulation, which does not distinguish between the models. Therefore, we conducted our own analyses of a previously published dataset of V1 responses in the awake monkey (Ecker et al., 2010) (Figure 6E). The modulation of these correlations by the stimulus could only be accounted for by the SSN. First, we found that correlations between similarly tuned cells were significantly modulated by the stimulus (Figure 6E, orange; repeated-measures ANOVA F(2,274)=5.29, p=0.006), and this modulation had a U rather than an M shape. More critically, also in agreement with the predictions of the SSN but not of the attractor model, correlations between orthogonally tuned cells were unaffected by the stimulus (Figure 6E, gray; repeated-measures ANOVA F(2,274)=0.04, p=0.961). While the magnitude of correlations in either model was overall larger than in the data, this simply reflected the relatively small number of neurons in the models (model correlations could be decreased without affecting the shape and extent of their stimulus tuning by substituting each model unit by several neurons with independent spiking noise).

### Experimental Predictions: Temporal Dynamics of Variability Modulation

**Figure 7:**
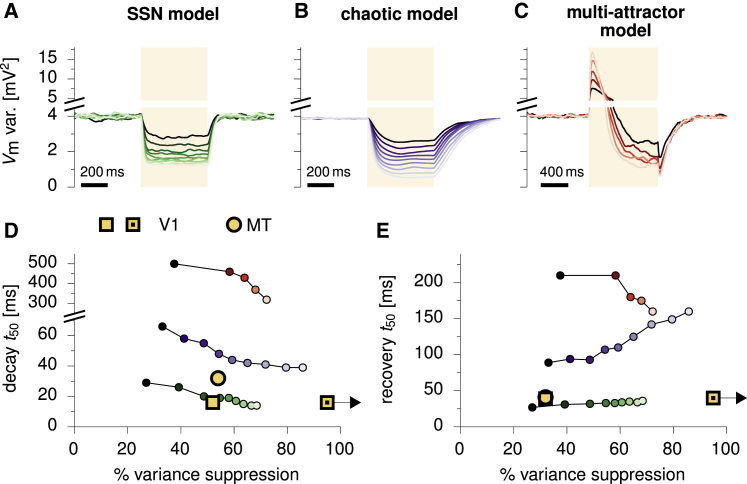
Temporal Dynamics of Variability Modulation in the SSN versus Other Models (A) Time course of variability reduction and recovery in the ring SSN in response to a step input (shaded area, 500 ms duration) of increasing amplitude (dark to light). Variability is quantified by the population-averaged across-trial $V_{\text{m}}$ variance. (B) Same as (A), for chaotic network dynamics (Rajan et al., 2010). (C) Same as (A), for a continuous, multi-attractor network (Ponce-Alvarez et al., 2013). The stimulus is twice as long as in (A) and (B), so that variability suppression can be observed following the characteristic transient increase. (D) Timescale of variability suppression (time to reach half of the total suppression) as a function of the percentage of variance suppression in the three models, extracted from their corresponding variability trajectories (colors as in A–C). (E) Same as (D), for recovery timescales (time to recover half of the total suppression). In both (D) and (E), open yellow squares indicate V1 data from anesthetized cat (estimated from Figure S4 in Churchland et al., 2010); yellow circles show data from anesthetized monkey MT (Figure S4 in Churchland et al., 2010); dotted yellow circles show our analysis of the awake monkey V1 data of Ecker et al., 2010. Variability refers to the above-Poisson part of spike count variability (i.e., population-averaged Fano factor minus one), and time constants discard latencies in data. In the data of Ecker et al., 2010, the Fano factor dropped below one, effectively resulting in >100% variance suppression with our definition (right-pointing arrows). All results regarding the SSN and the multi-attractor model shown in this figure were obtained by using the same parameters as in previous figures (Figures 4, 5, and 6).

We hypothesized that the fundamentally different mechanisms responsible for variability modulation in the SSN, the multi-attractor, and the chaotic dynamical regimes (Figure 1) should be revealed in the dynamics of variability suppression at stimulus onset and of variability recovery at stimulus offset. In order to test this, we used the same models for the SSN and multi-attractor models as above, and we implemented the classical chaotic model of Rajan et al. (2010) (STAR Methods), in which variability suppression had previously been shown to occur. We then measured the across-trial variability (averaged across neurons) following the onset and offset of a step stimulus in each model (Figures 7A–7C, shaded areas), as we parametrically varied the amplitude of the stimulus and therefore the degree of variability suppression (Figures 7A–7C, dark to light colors).

In the SSN, the timescales on which both suppression and recovery of variability occurred were nearly as fast as the single-neuron time constant (20 ms in these simulations; Figures 7D and 7E, green). In contrast, in chaotic networks, both these timescales were several (4–15) times longer than the single-neuron membrane time constant (Figures 7D and 7E, blue). More importantly, recovery times were much longer than suppression times in the chaotic network and increased with increasing stimulus strength and thus increasing amount of variability suppression during the stimulus period, neither of which was the case in the SSN. In the multi-attractor network, both the dynamics of the network activity and those of variability were much slower than in the SSN (Figures 7D and 7E, red). Moreover, we found that, unlike in the SSN or the chaotic model, variability increased transiently immediately following stimulus onset (before eventually decreasing to its new steady state; Figure 7C). The cause of this behavior was the slow rotation of the activity bump from its random position at the time of stimulus onset to the location where cells’ preferred orientation matched the stimulus orientation (Figure S10). Thus, we expect this behavior to be generic at least to the subclass of multi-attractor models that have a continuous ring of attractors and thus show such rotational response, which likely include those that can address the orientation- or direction-tuning of variability reduction in V1 and MT.

The timescales of variability suppression and recovery found experimentally in anaesthetized cat V1 and awake monkey MT (Figures 7D and 7E, open square and circle; Churchland et al., 2010) and by our own analysis of awake monkey V1 data (Figures 7D and 7E, dotted square; Ecker et al., 2010) were short and nearly identical. Moreover, recovery times showed little dependence on the amount of variability suppression (comparing across areas), and there was no transient increase in variability at stimulus onset (Figure 4F; Churchland et al., 2010). These results confirm the predictions of the SSN and are at odds with the dynamics of variability modulation as predicted by the multi-attractor and chaotic regimes.

## Discussion

We studied the modulation of variability in a stochastic, nonlinear model of cortical circuit dynamics. We focused on a simple circuit motif that captured the essence of cortical networks: noisy excitatory and inhibitory populations interacting in a recurrent but stable way despite expansive single-neuron nonlinearities. This stochastic stabilized supralinear network (SSN) reproduced key aspects of variability in the cortex. During spontaneous activity, i.e., for weak external inputs, model neurons showed large and relatively slow synchronous fluctuations in their membrane potentials. These fluctuations were considerably amplified by the network relative to that expected from the input alone and were quickly quenched and decorrelated by stimuli. The model thus explains and unifies a large body of experimental observations made in diverse systems under various conditions (Churchland et al., 2006, Churchland et al., 2010, Finn et al., 2007, Poulet and Petersen, 2008, Gentet et al., 2010, Poulet et al., 2012, Tan et al., 2014, Chen et al., 2014). Moreover, the drop in variability was tuned to specific stimulus features in a model of V1/MT, also capturing recent experimental findings (Ponce-Alvarez et al., 2013, Lin et al., 2015, Lombardo et al., 2015) as well as our own analyses of a previously published dataset (Ecker et al., 2010).

The main insight of our analysis was that in a network of nonlinear neurons with an expansive firing rate nonlinearity, increasing the input increases the effective connection strengths of the network, which in turn modulates the variability of responses. We identified two opposing effects of increasing effective connectivity on variability: the amplification of variability by excitatory-inhibitory interactions (balanced amplification), which dominates at very low (spontaneous) levels of input, and the quenching of variability by increased inhibitory feedback, which dominates for stimulus-driven input. Critically, these network effects preferentially act on smooth patterns of activity that are aligned with the anatomical connectivity of the network, so that it is the shared component of variability that is suppressed and modulated by stimuli. Taken together, we showed that these mechanisms robustly produced experimentally observed spatial and temporal patterns of variability quenching and modulation, whereas the dynamics of the network always remained in the vicinity of a single attractor state, unlike previously proposed mechanisms based on multi-attractor or chaotic dynamical regimes.

### Sources and Effects of Stochasticity

We focused on how the network shapes variability and assumed that the variability originates in correlated noise input to the network; such input correlations could arise due to upstream areas already exhibiting noise correlations (e.g., thalamic input to V1, Sadagopan and Ferster, 2012) and/or because of feedforward connectivity implying shared inputs (e.g., Kanitscheider et al., 2015). In contrast, other models have focused on how circuits intrinsically generate slow correlated variability (Litwin-Kumar and Doiron, 2012, Stringer et al., 2016). Nevertheless, our model also points to an important mechanism for creating shared variability, namely the strong amplification of the input noise by balanced amplification (see also Kriener et al., 2008, Murphy and Miller, 2009, Hennequin et al., 2014).

Although most of our analyses were based on rates, rather than spikes, the effect of fast fluctuations resulting from spiking noise were not ignored, but were incorporated implicitly in the power-law input/output nonlinearity of neurons in the model (Equation 3) and in the stochastic spike-generation mechanism used in our spiking network simulations (Figure 3, STAR Methods). Theoretical work (Miller and Troyer, 2002, Hansel and van Vreeswijk, 2002) shows that these fast fluctuations are the key factor causing momentary firing rates (on the 30–50 ms timescale of $V_{\text{m}}$ fluctuations considered here) to be a supralinear, power-law function of mean voltages, a critical feature of our model. As experiments, as well as our model, show that only the shared but not the private part of variability is modulated by stimuli (Churchland et al., 2010), we expect our assumption that the exponent of the threshold power-law nonlinearity can be considered constant (implying that fast private spiking fluctuations are not affected by stimuli) to be valid to a good approximation. We also expect that a more detailed model explicitly including these fast fluctuations would allow a more systematic study of the effects of stimuli on high-frequency (gamma) oscillations (Ray and Maunsell, 2010), which our current model could only partially account for (Figure 3B).

### Tight versus Loose E-I Balance

While we focused on the sources and modulation of slower, correlated fluctuations, a classical model of cortical variability, the “balanced network” (van Vreeswijk and Sompolinsky, 1998), focused on the origin of fast fluctuations from spiking noise. In that model, very large external and recurrent inputs cancel or “balance” to yield a much smaller net input. This mechanism can self-consistently generate the voltage variability to generate irregular spiking. However, the very strong, very fast inhibitory feedback in the balanced network suppresses correlated rate fluctuations away from the stable state (van Vreeswijk and Sompolinsky, 1998, Renart et al., 2010, Tetzlaff et al., 2012), leaving only fast, private variability due to irregular spiking (though “breaking balance” can restore correlated variability; Litwin-Kumar and Doiron, 2012, Rosenbaum et al., 2017). Because the shared variability is already eliminated, stimuli cannot modulate that variability.

As opposed to the “tight balance” between excitation and inhibition in the classical balanced network model, the SSN in the stimulus-driven regime is “loosely balanced”: the same mathematical cancellation of external and recurrent input occurs, but in a regime in which inputs are not large and the net input after cancellation is comparable in size to the factors that cancel (Ahmadian et al., 2013). This regime is supported by observations that external input is comparable to, rather than very much larger than, the net input received by cortical cells (Ferster et al., 1996, Chung and Ferster, 1998, Lien and Scanziani, 2013, Li et al., 2013). This loose balance allows correlated variability to persist and be modulated by stimuli. Variability quenching in the stochastic SSN robustly occurred as the input pushed the dynamics to stronger and stronger inhibitory dominance. Consistent with this, with increasing strength of external input, the ratio of inhibition to recurrent excitation received by neurons in the network increases (Rubin et al., 2015), as observed in layers 2/3 of mouse S1 (Shao et al., 2013) and V1 (Adesnik, 2017). In the balanced network, the ratio of inhibitory to excitatory activity would be fixed regardless of the strength of activation. The balanced network also only yields responses that are linear functions of the input (though see Mongillo et al., 2012), whereas the loosely balanced regime replicates many nonlinear cortical response properties (Rubin et al., 2015), including the profound dependence of correlated variability on stimuli. Although our model does not focus on the origins of fast spiking variability, spiking models in the loosely balanced SSN regime can, given noisy inputs (e.g., Sadagopan and Ferster, 2012), yield the irregular spiking characteristic of cortex (unpublished data).

### Further Factors Modulating Variability

We analyzed variability modulation solely as arising from intrinsic network interactions, but other factors may also contribute (Doiron et al., 2016). External inputs may be modulated; for example, the drop with contrast in Fano factors in the lateral geniculate nucleus (LGN) has been argued to underlie $V_{\text{m}}$ variability decreases in V1 simple cells (Sadagopan and Ferster, 2012; but see Malina et al., 2016). However, since high-contrast stimuli also cause firing rates to increase in LGN, the total variance of LGN-to-V1 inputs (scaling with the product of the LGN Fano factor and mean rate) is modulated far less by contrast. This provides some justification for our model choice that input variance did not scale with contrast. Changes in input correlations have also been suggested as a potential mechanism underlying variability modulation (Bujan et al., 2015). However, the proposed mechanism would require a stimulus to specifically increase the correlations of the different inputs onto individual cells (and this increase should be tuned to the stimulus) while leaving the correlation of inputs between cells unchanged. This seems difficult to achieve in cortex, where nearby cells are likely to share a significant amount of input and correlations are generally observed to decrease, rather than increase, with stimulus strength (Churchland et al., 2010).

One particular form of external input modulation, that involving changes in brain state, has been proposed to directly contribute to correlated variability in both awake (Poulet and Petersen, 2008, Ecker et al., 2016) and anesthetized cortex (Ecker et al., 2014, Goris et al., 2014, Lin et al., 2015, Mochol et al., 2015), so that a reduction of state switching would underlie the reduction of shared variability (Mochol et al., 2015, Ecker et al., 2016). To the extent that correlated noise in the input to our model is aligned with a uniform activity pattern, this input can also be regarded as having a single scalar “brain state”-like component that is changing in time. However, our analysis suggests that the variability of this component needs not be modulated directly by the stimulus to account for variability quenching in network responses. Instead, our network used its intrinsic mechanisms to quench variability in response to a stimulus. Importantly, these intrinsic mechanisms not only quenched this uniform component of variability (Figure S8), but also produced more complex patterns of variability modulation via “bump” kinetics that a single brain state-dependent mechanism could not account for.

Cellular factors may also modulate variability. For example, inhibitory reversal potential or spike threshold may set boundaries limiting voltage fluctuations, which would more strongly limit voltage fluctuations in more hyperpolarized or more depolarized states, respectively; conductance increases will reduce voltage fluctuations; and dendritic spikes may contribute more to voltage fluctuations in some states than others (Stuart and Spruston, 2015). A joint treatment of external input, cellular, and recurrent effects may be needed to explain, for example, why $V_{\text{m}}$ variability appears strongest near the preferred stimulus in anaesthetized cat V1 (Finn et al., 2007) or why overall $V_{\text{m}}$ variability grows with visual stimulation in some neurons of awake macaque V1 (Tan et al., 2014).

Cellular properties may themselves be subject to change over time, thereby causing changes in variability. For example, various mechanisms (e.g., attention, intrinsic and synaptic plasticity, neuromodulators, anesthetics) can change the input/output gain of single neurons and the synaptic efficacies of the network. As all these changes eventually lead to changes in effective connectivity, our work offers a principled approach to study their effects on variability and is thus complementary to previous studies that focused on the consequences of different anatomical connectivity patterns on correlations (Kriener et al., 2008, Tetzlaff et al., 2012, Ostojic, 2014, Hennequin et al., 2014).

### Effects of Normalization on Variability

The nonlinear response properties of our network were crucial for the modulation of variability by stimuli. These nonlinearities had been shown to capture ubiquitous phenomena involving nonlinear response summation to multiple stimuli, including normalization, surround suppression, and their dependencies on stimulus contrast (Rubin et al., 2015, Ahmadian et al., 2013). As such, the SSN reproduces much of the phenomenology of the “normalization model” of cortical responses (Carandini and Heeger, 2011) and provides a circuit substrate for it.

However, while response normalization has previously been studied for deterministic steady-state responses, our results can be interpreted as showing that it also plays a role in the suppression of ongoing variability by stimuli, as well as shaping the structure of stimulus-evoked noise correlations. Specifically, in the deterministic SSN, steady-state responses to multiple stimuli add sublinearly, and as one stimulus becomes stronger than another, the response to their simultaneous presentation becomes “winner take all,” i.e., dominated by the response to the stronger stimulus alone (Rubin et al., 2015). This provides an alternative conceptual explanation of why, in the stochastic SSN, a stronger mean input drive relative to the noise input leads to greater suppression of the noise’s contribution to the total network response, thus quenching variability.

Our results go beyond what could be predicted based on this simple qualitative link between steady-state normalization and variability quenching. First, we found a specific quantitative form of normalization in our network: an approximate conservation of the integrated activity across a bump of activity that forms around cells tuned to the stimulus orientation, despite fluctuations in its width. In turn, this predicted a specific pattern of noise correlations that we found contributed substantially to noise variability in V1 of the awake monkey (Figure 6). Second, we were able to study the dynamics with which variability was suppressed following stimulus onset and recovered following stimulus offset and found a good match to experimental data (Figure 7).

### The Origin and Role of Inhibitory Dominance

We found that an increase in inhibitory dominance was necessary for the suppression of variability and correlations in the SSN. In line with that, Stringer et al. (2016) studied rodent A1 and V1 in various awake and anesthetized brain states and found that desynchronized states with weaker correlations were accompanied by enhanced activity of putative fast-spiking inhibitory neurons. By fitting a recurrent spiking E-I network model to the data, they found that enhanced inhibitory feedback was the key factor capturing the suppression of correlations. However, the enhanced dominance of inhibition with increasing network activation, which suppresses correlations, was artificially incorporated into the model by making the inhibitory conductance an exponential function of the inhibitory spike count. In contrast, our model provides a dynamical mechanism by which inhibition becomes increasingly dominant with increasing network activation.

Kanashiro et al. (2017) proposed a mechanism similar to ours for the top-down suppression of correlated variability by attention, rather than bottom-up suppression by a stimulus. They also proposed that this arises from enhanced inhibitory feedback resulting from increased effective connectivity due to expansive input/output functions. However, their conclusions differed significantly from ours. They found that, for attention to suppress variability, attentional input had to be directed dominantly to inhibitory cells, while for attention to increase the gain of response to stimuli, stimuli had to give input dominantly to excitatory cells. Note that this implies that stimuli would not suppress variability. We have found that neither of these conditions are necessary (Methods S4) and that stimuli robustly suppress variability. In particular, increasing input strength decreased variability across a wide range of relative strengths of input to excitatory versus inhibitory cells (Figure S2). The main reason for these differences in conclusions is the special, non-generic parametrization of the model studied by Kanashiro et al. (2017) in which a neuron’s projections to excitatory and to inhibitory neurons were statistically identical, which precluded the SSN regime (Methods S2).

### The Dynamical Regime of Cortical Activity

Two proposals have been made previously to explain quenching of variability by a stimulus: a stimulus may quench multi-attractor dynamics to create single-attractor dynamics (Blumenfeld et al., 2006, Litwin-Kumar and Doiron, 2012, Deco and Hugues, 2012, Ponce-Alvarez et al., 2013, Doiron and Litwin-Kumar, 2014, Mochol et al., 2015), and a stimulus may quench chaotic dynamics to produce non-chaotic dynamics (Molgedey et al., 1992, Bertschinger and Natschläger, 2004, Sussillo and Abbott, 2009, Rajan et al., 2010, Laje and Buonomano, 2013). Our results propose a very different dynamical regime underlying variability quenching, which can be distinguished from the multi-attractor or chaos-suppression models.

Conceptually, the stochastic SSN differs from previous models of stimulus-driven quenching of shared variability in exhibiting a single stable state in all conditions—spontaneous, weakly driven, strongly driven—whereas the others show this only when strongly driven. Furthermore, quenching of variability and correlations in the SSN is highly robust, arising from two basic properties of cortical circuits: inhibitory stabilization of strong excitatory feedback (Tsodyks et al., 1997, Ozeki et al., 2009) and supralinear input/output functions in single neurons (Priebe and Ferster, 2008). In contrast, models of multi-attractor or chaotic dynamics can either account only for the modulation of average pairwise correlations (Mochol et al., 2015) or else require considerable fine tuning of connections (Litwin-Kumar and Doiron, 2012, Ponce-Alvarez et al., 2013) to account for more detailed correlation patterns. Moreover, as studied thus far (Rajan et al., 2010, Ponce-Alvarez et al., 2013, Mochol et al., 2015; but see Harish and Hansel, 2015, Kadmon and Sompolinsky, 2015, Mastrogiuseppe and Ostojic, 2017), they typically ignore Dale’s law (the separation of E and I neurons) and its consequences for variability, e.g., balanced amplification. These differences between the SSN and previous models also lead to two main experimentally testable features that we used to distinguish their respective dynamical regimes: the tuning and the timing of variability modulation.

With respect to the stimulus tuning of spike count Fano factors and noise correlations, we found that multi-attractor networks could only predict an M-shaped modulation while the SSN could produce either M- or U-shaped modulations depending on the tuning width of inputs relative to that of connectivity. Indeed, while most types of stimuli in MT were found to result in an M-shaped modulation (Ponce-Alvarez et al., 2013), coherent plaids (Ponce-Alvarez et al., 2013) and random moving dots (Lombardo et al., 2015) in the macaque as well as moving dot fields and drifting gratings in the marmoset (Selina Solomon, personal communication; Solomon et al., 2015) result in a pronounced U-shaped modulation of Fano factors in MT, and our own analyses of V1 data also revealed a U-shaped modulation. Interestingly, our results also suggested that irrespective of the precise shape of the modulation of spike count statistics, membrane potential variability in the SSN should always exhibit a U-shaped profile (Figure 4), which could be tested in future experiments. Critically, we also identified a rarely analyzed aspect of spatial correlation patterns that could most clearly distinguish between different models: the modulation of correlations between orthogonally tuned cells. The SSN predicted only very weak modulation for such cell pairs, while multi-attractor dynamics resulted in modulations that were as deep as for pairs of similarly tuned cells. We found that data from awake macaque V1 supported the SSN.

Another distinctive feature of the SSN regime is the speed of its dynamics, and in particular the speed with which variability is modulated as the stimulus is changed. In contrast to multi-attractor and chaotic dynamics, in which variability modulation happens on timescales that are considerably slower than the single neuron time constant, the SSN produces fast variability modulation on a timescale comparable to the neural time constant. The timescales of variability modulation we extracted from data recorded in monkey visual cortical areas (Churchland et al., 2010, Ecker et al., 2010) were fast, on the order of 20–50 ms, providing further support to the SSN.

In summary, the SSN robustly captures multiple aspects of stimulus modulation of correlated variability and suggests a dynamical regime that uniquely captures a wide array of behaviors of sensory cortex. In turn, our work suggests a principled approach to use data on cortical variability to identify the dynamical regime in which the cortex operates.

## STAR★Methods

### Key Resources Table

**Table:** 

REAGENT or RESOURCE	SOURCE	IDENTIFIER
**Deposited Data**		

Awake monkey V1 dataset	Ecker et al., 2010	http://bethgelab.org/datasets/v1gratings

**Software and Algorithms**		

OCaml (for all simulations)	Open source	http://www.ocaml.org
Sqlite3 (for V1 data analysis)	Sqlite Consortium	https://sqlite.org/index.html
Mathematica	Wolfram	https://www.wolfram.com/mathematica
Gnuplot	Open source	http://www.gnuplot.info

### Contact for Reagent and Resource Sharing

As Lead Contact, Guillaume Hennequin is responsible for all reagent and resource requests. Please contact Guillaume Hennequin at g.hennequin@eng.cam.ac.uk with requests and inquiries.

### Method Details

The values of all the parameters mentioned below are listed in the tables below. All differential equations detailed below were integrated using a simple Euler scheme with time step 0.1 ms.

**Table:** Parameters Used in the SSN Simulations

Symbol	Figure 2	Figure 3	Figures 4, 5, 6, and 7	Unit	Description
N_E_	1	4,000	50	–	Number of excitatory units
N_I_	1	1,000	50	–	Number of inhibitory units
τ_E_	20			ms	Membrane time constant (E neurons)
τ_I_	10			ms	Membrane time constant (I neurons)
V_rest_	−70			mV	Resting membrane potential
V_0_	−70			mV	Rectification threshold potential
k	0.3			mV^−n^ ⋅ s^−1^	Nonlinearity gain
n	2			–	Nonlinearity exponent
W_EE_	1.25			mV ⋅ s	E→E connection weight (or sum thereof)
W_IE_	1.2			mV ⋅ s	E→I connection weight (or sum thereof)
W_EI_	0.65			mV ⋅ s	I→E connection weight (or sum thereof)
W_II_	0.5			mV ⋅ s	I→I connection weight (or sum thereof)
τ_noise_	50			ms	Noise correlation time constant
σ_0,E_	0.2	1		mV	Noise standard deviation (E neurons)
σ_0,I_	0.1	0.5		mV	Noise standard deviation (I neurons)
p_E_	–	0.1	–	–	Outgoing connection probability (E neurons)
p_I_	–	0.4	–	–	Outgoing connection probability (I neurons)
τ_syn_	–	2	–	ms	Synaptic time constants
Δ	–	0.5	–	ms	Axonal delay
ℓ_syn_	–		45	deg.	Connectivity length scale
ℓ_noise_	–		60	deg.	Noise correlation length scale
ℓ_stim_	–		60	deg.	Stimulus tuning length scale of the input
b	–		2	mV	Input baseline
A_max_	–		20	mV	Maximum input modulation (100% contrast)
θ_stim_	–		0	deg.	Stimulus direction

**Table:** Parameters Used in the Multi-attractor Network Simulations

Symbol	Figures 6 and 7	Unit	Description
N	100	–	Number of units
τ_m_	10	ms	Membrane time constant
k	0.1	mV^−1^	Nonlinearity gain
g_max_	100	ms^−1^ ⋅ mV^−1^	Maximal firing rate
W	−40/g_max_	mV ⋅ s	Average connection weight
W_Δ_	33/g_max_	mV ⋅ s	Tuning-dependent modulation of connection weight
τ_noise_	50	ms	Noise correlation time constant
σ_0_	0.15	mV	Noise standard deviation
ℓ_noise_	60	deg.	Noise correlation length scale
ℓ_stim_	60	deg.	Stimulus tuning length scale of the input
b	2	mV	Input baseline
A	0.1	mV	Depth of input tuning
θ_stim_	0	deg.	Stimulus direction

**Table:** Parameters Used in the Chaotic Network Simulations

Symbol	Figure 7	Unit	Description
N	2,000	–	Number of units
τ_m_	10	ms	Membrane time constant
σ_W_	2	–	Standard deviation of connection weights

#### SSN model

Our rate-based networks contained $N_{E}$ excitatory and $N_{I}$ inhibitory units, yielding a total $N=N_{E}+N_{I}$ units. The circuit dynamics were governed by (see also Methods S1):

(Equation 2) $$\tau_{i}\;\frac{dV_{i}}{dt}=-V_{i}+V_{\text{rest}}+h_{i}\left(t\right)+\eta_{i}\left(t\right)+\sum_{j\in \text{E cells}}W_{ij}\;r\left(V_{j}\right)-\sum_{j\in \text{I cells}}W_{ij}\;r\left(V_{j}\right)\text{,}$$

where $V_{i}$ denotes the $V_{\text{m}}$ of neuron *i*, $\tau_{i}$ is its membrane time constant, $V_{\text{rest}}$ is a resting potential, $W_{ij}$ is the (positive or zero) strength of the synaptic connection from neuron *j* to neuron *i*, and $h_{i}\left(t\right)$ is the potentially time-varying but deterministic component (the mean) of external input to which a noise term $\eta_{i}\left(t\right)$ is added (see below, “Input noise”). The momentary firing rate of cell *j* was given by a threshold-powerlaw function of its membrane potential:

(Equation 3) $$r\left(V_{j}\right)=k\;{\left\lfloor V_{j}-V_{0}\right\rfloor }_{+}^{n}\text{.}$$

Experiments support Equation 3 when both membrane potentials and spike counts are averaged in 30 ms time bins (Priebe and Ferster, 2008). Accordingly, $V_{i}$ in Equation 2 can be understood as the coarse-grained (low-pass filtered) version of the raw somatic membrane potential; in particular it does not incorporate the action potentials themselves. Thus the effective time resolution of our model was around 30 ms which allowed studying the effects of inputs that did not change significantly on timescales shorter than that. Accordingly, in Equation 2 we assumed that external noise had a time constant $\tau_{\text{noise}}=50$ ms, in line with membrane potential and spike count autocorrelation timescales found across the cortex (Azouz and Gray, 1999, Berkes et al., 2011, Murray et al., 2014).

Equations 2 and 3 together define the stabilized supralinear network model studied in Ahmadian et al. (2013) and Rubin et al. (2015), but formulated with voltages rather than rates as the dynamical variables (the two formulations are mathematically equivalent when all neurons have the same time constant, Miller and Fumarola, 2012) and with the crucial addition of noise that enables us to study variability. In all the figures of the main text, the exponent of the power-law nonlinearity was set to n=2 (but see Figure S2 for n > 2). Methods S2 explores more general scenarios.

##### Mean external input

In the reduced rate model of Figure 2, each unit received the same constant mean input *h*. In the ring model, the mean input to neuron *i* was the sum of two components,

(Equation 4) $$h_{i}\left(\theta_{\text{stim}}\right)=b+c\cdot A_{\text{max}}\cdot \text{exp}\left(\frac{\text{cos}\left(\theta_{i}-\theta_{\text{stim}}\right)-1}{\ell_{\text{stim}}^{2}}\right)\text{.}$$

The first term b=2 mV is a constant baseline which drives spontaneous activity. The second term models the presence of a stimulus with orientation $\theta_{\text{stim}}$ in the visual field as a circular-Gaussian input bump of “half width” $\ell_{\text{stim}}$ centered around $\theta_{\text{stim}}$ and scaled by a factor *c* (increasing *c* represents increasing stimulus contrast), taking values from 0 to 1, times a maximum amplitude $A_{\text{max}}$. We assumed for simplicity that E and I cells are driven equally strongly by the stimulus, though this could be relaxed.

##### Input noise

The input noise term $\eta_{i}\left(t\right)$ in Equation 2 was modeled as a multivariate Ornstein-Uhlenbeck process:

(Equation 5) $$\tau_{\text{noise}}d\eta =-\eta dt+\sqrt{2\tau_{\text{noise}}\Sigma^{\text{noise}}}\;d\xi \text{,}$$

where dξ is a collection of *N* independent Wiener processes and $\Sigma^{\text{noise}}$ is an N×N input covariance matrix (see below). Note that Equation 5 implies ${\langle \eta_{i}\left(t\right)\;\eta_{j}\left(t+\tau \right)\rangle }_{t}={\text{Σ}}_{ij}^{\text{noise}}e^{-\left|\tau \right|/\tau_{\text{noise}}}$.

In the reduced two-population model (Figure 2), noise terms were chosen to be uncorrelated, i.e., ${\text{Σ}}_{ij}^{\text{noise}}=\sigma_{\alpha \left(i\right)}^{2}\delta_{ij}$ (where $\delta_{ij}=1$ if i=j and 0 otherwise), α(i)∈{E,I} is the E/I type of neuron *i*, and $\sigma_{\alpha }^{2}$ is the variance of noise fed to population α (see Equation 7 below). In the spiking two-population model (Figure 3), input noise covariance was uniform, such that ${\text{Σ}}_{ij}^{\text{noise}}=\sigma_{\text{noise}}^{2}\left[\delta_{ij}\;\left(1-\rho \right)+\rho \right]$, with the pairwise correlation coefficient set to ρ=0.2 (see Figure S5 for the dependence of our results on ρ). In the ring model (Figures 4, 5, 6, and 7), the noise had spatial structure, with correlations among neurons decreasing with the difference in their preferred directions following a circular-Gaussian:

(Equation 6) $${\text{Σ}}_{ij}^{\text{noise}}=\sigma_{\alpha \left(i\right)}\;\sigma_{\alpha \left(j\right)}\;\text{exp}\left(\frac{\text{cos}\left(\theta_{i}-\theta_{j}\right)-1}{\ell_{\text{noise}}^{2}}\right)\text{,}$$

where $\theta_{i}$ and $\theta_{j}$ are the preferred orientations of neurons *i* and *j* (exc. or inh.), and $\ell_{\text{noise}}$ is the correlation length (see table “Parameters Used in the SSN Simulations”). The noise amplitude has the natural scaling

(Equation 7) $$\sigma_{\alpha }=\sigma_{0,\alpha }\;\sqrt{1+\frac{\tau_{\alpha }}{\tau_{\text{noise}}}}\;\left(\alpha \in \left\{E,I\right\}\right)$$

such that, in the absence of recurrent connectivity (W=0), the input noise alone would drive $V_{\text{m}}$ fluctuations of standard deviation $\sigma_{0,E}$ or $\sigma_{0,I}$, measured in mV, in the E or I cells, respectively. We chose values of $\sigma_{0,E}$ that yielded spontaneous Fano factors in the range 1.3-1.5 where appropriate, and chose $\sigma_{0,I}=\sigma_{0,E}/2$ to make up for the difference in membrane time constants between E and I cells (see table “Parameters Used in the SSN Simulations”).

##### Connectivity

The synaptic weight matrix in the reduced model was given by

(Equation 8) $$W=\left(\begin{array}{cc} W_{\mathrm{EE}} & -W_{\mathrm{EI}}\\ W_{\mathrm{IE}} & -W_{\mathrm{II}} \end{array}\right)\text{,}$$

where $W_{AB}$ is the magnitude of the connection from the unit of type *B* (E or I) to that of type *A* (see table “Parameters Used in the SSN Simulations” for parameter values). In the ring model, connectivity fell off with angular distance on the ring, following a circular-Gaussian profile:

(Equation 9) $$W_{ij}\propto \text{exp}\left(\frac{\text{cos}\left(\theta_{i}-\theta_{j}\right)-1}{\ell_{\text{syn}}^{2}}\right)\text{,}$$

where $\theta_{i}$ and $\theta_{j}$ are the preferred orientations of neurons *i* and *j* (exc. or inh.), and $\ell_{\text{syn}}$ sets the length scale over which synaptic weights decay (see table “Parameters Used in the SSN Simulations”). The connectivity matrix W was further rescaled in each row and in each quadrant, such that the sum of incoming E and I weights onto each E and I neuron (4 cases) matched the values of $W_{\mathrm{EE}}$, $W_{\mathrm{IE}}$, $-W_{\mathrm{EI}}$ and $-W_{\mathrm{II}}$ in the reduced model. Thus, all connectivity matrices used in the SSN model obeyed Dale’s law.

##### Simulated spike counts

To relate the firing rate model to spiking data in Figures 4 and 6, we assumed that action potentials were emitted as inhomogeneous (doubly stochastic) Poisson processes with time-varying rate $r\left(V_{m}\right)$ given by Equation 3. Unlike in the full spiking model (see below), spikes did not “re-enter” the dynamics of Equation 2, according to which neurons influence each other through their firing rates. Spikes were counted in 100 ms time bins and spike count statistics such as Fano factors and pairwise correlations were computed as standard.

#### Spiking SSN model

##### Dynamics

In the spiking model (Figure 3), neuron *i* emitted spikes stochastically with an instantaneous probability equal to $dt\;r\left(V_{i}\right)$, with time-varying rate $r\left(V_{i}\right)$ given by Equation 3, consistent with how (hypothetical) spikes were modeled in the rate-based case (*cf.* above). Presynaptic spikes were filtered by synaptic dynamics into exponentially decaying postsynaptic currents (E or I):

(Equation 10) $$\frac{da_{j}}{dt}=-\frac{a_{j}}{\tau_{\text{syn}}}+\sum_{t_{j}}\delta \left(t-t_{j}-\text{Δ}\right)\text{,}$$

where the $t_{j}$’s are the firing times of neuron *j*, $\tau_{\text{syn}}=2$ ms is the synaptic time constant, and Δ=0.5 ms is a small axonal transmission delay (which enabled the distribution of the simulations onto multiple compute cores; Morrison et al., 2005). Synaptic currents then contributed to membrane potential dynamics according to

(Equation 11) $$\tau_{i}\;\frac{dV_{i}}{dt}=-V_{i}+V_{\text{rest}}+h_{i}\left(t\right)+\eta_{i}\left(t\right)+\sum_{j\in \text{E}\;\text{cells}}J_{ij}\;a_{j}\left(t\right)-\sum_{j\in \text{I}\;\text{cells}}J_{ij}\;a_{j}\left(t\right)\text{,}$$

where the synaptic efficacies $J_{ij}$ are described below, and the noise term $\eta_{i}$ was modeled exactly as described above.

##### Connectivity

For each neuron *i*, we drew $p_{E}N_{E}$ excitatory and $p_{I}N_{I}$ inhibitory presynaptic partners, uniformly at random. Connection probabilities were set to $p_{E}=0.1$ and $p_{I}=0.4$ respectively. The corresponding synaptic weights took on values $J_{ij}=W_{\alpha \beta }/\left(\tau_{\text{syn}}\;p_{\beta }\;N_{\beta }\right)$ where {α,β}∈{E,I} denote the populations to which neuron *i* and *j* belong respectively, and $W_{\alpha \beta }$ are the connections in the reduced model (see table “Parameters Used in the SSN Simulations”). This choice was such that, for a given set of mean firing rates in the E and I populations, average E and I synaptic inputs to E and I cells matched the corresponding recurrent inputs in the rate-based model. Synapses that were not drawn were obviously set to $J_{ij}=0$.

##### Local field potential

As a proxy for LFP in Figure 3, we took the momentary population-averaged $V_{\text{m}}$ (Mazzoni et al., 2015 simulated various proxies and, although some proxies were more accurate, they found the average $V_{\text{m}}$ to be reasonably accurate).

#### Multi-attractor model

We compared our ring SSN model to a version of the ring attractor model published by Ponce-Alvarez et al. (2013). The ring attractor model had a single population with a similar ring topology, and—using the same notation as above—the connectivity took the form (*cf.* Equation 9)

(Equation 12) $$W_{ij}=\bar{W}+\frac{W_{\text{Δ}}}{N}\text{cos}\left(\theta_{i}-\theta_{j}\right)\text{,}$$

where N=100 is the number of neurons, and $\bar{W}$ and $W_{\text{Δ}}$ are two parameters that control the average connection strength and modulation with tuning dissimilarity, respectively. Note that, in general, this connectivity matrix could violate Dale’s law but with the specific parameters used here it did not (see table “Parameters Used in the Multi-attractor Network Simulations”). Instead, all connections were inhibitory to keep the system in the marginally stable regime (as in Ponce-Alvarez et al., 2013). The dynamics of the network obeyed a similar stochastic differential equation as for the ring SSN (Equation 2), namely

(Equation 13) $$\tau_{\text{m}}\;\frac{dV_{i}}{dt}=-V_{i}+h_{i}\left(t\right)+\eta_{i}\left(t\right)+\sum_{j}W_{ij}\;r\left(V_{j}\right)\text{,}$$

with the momentary firing rate of cell *j* given by a rectified saturating firing rate nonlinearity (*cf.* Equation 3):

(Equation 14) $$r\left(V_{j}\right)=g_{\text{max}}\text{tanh}\left(k{\left\lfloor V_{j}\right\rfloor }_{+}\right)\text{,}$$

and a noise process η identical to the one we used in the SSN (same spatial and temporal correlations, Equations 5 and 6), with a variance adjusted so as to obtain Fano factors of about 1.5 during spontaneous activity (Figure S9B, black). The external input had both a constant baseline, *b*, and a contrast-dependent modulated component (*cf.* Equation 4):

(Equation 15) $$h_{i}=b+c\cdot \left(1-A+A\;\text{cos}\left(\theta_{i}-\theta_{\text{stim}}\right)\right)\text{,}$$

where *A* controlled the depth of the modulation, and *c* represents stimulus strength.

Note that although the phenomenology and dynamical regime of this model was consistent with that of Ponce-Alvarez et al. (2013) (Figure S9), the model differed from their original implementation in some of the details: our dynamics were written in voltage form, not in rate form, we had only one unit at each location on the ring (as opposed to small pools of neurons), and our input noise process had spatial correlations to allow for a more direct and consistent comparison with the ring SSN.

Our analysis of variability in this ring attractor network is presented in Figure S9 in a format identical to that of Figure 5, and shows that shared variability is entirely dominated by the fluctuations in the location of an otherwise very stable bump of activity.

#### Chaos suppression model

We also implemented a chaotic rate network of size N=2,000 with the following (deterministic) dynamics (*cf.* Equations 2 and 13):

(Equation 16) $$\tau_{\text{m}}\;\frac{dV_{i}}{dt}=-V_{i}+h_{i}\left(t\right)+\sum_{j}W_{ij}\;r\left(V_{j}\right)\text{,}$$

with an (unrectified) saturating firing rate nonlinearity (*cf.* Equations 3 and 14)

(Equation 17) $$r\left(V_{j}\right)=\text{tanh}\left(V_{j}\right)$$

(which could thus go negative as well as positive). Elements of the synaptic weight matrix were sampled i.i.d. from a normal distribution (thus violating Dale’s law, *cf.* Equations 9 and 12):

(Equation 18) $$W_{ij}∼N\left(0,\sigma_{\text{W}}^{2}/N\right)\text{,}$$

with $\sigma_{\text{W}}=2$, which placed the network in the chaotic regime (Sompolinsky et al., 1988). The external input was a constant input vector of the form (*cf.* Equations 4 and 15)

(Equation 19) $$h_{i}=c\cdot \text{cos}\left(\phi_{i}\right)\text{,}$$

where $\phi_{i}$ is a phase sampled i.i.d. from a uniform distribution between 0 and 2π, and *c* represents stimulus strength. See table “Parameters Used in the Chaotic Network Simulations” for all parameter values. As shown in Rajan et al. (2010), chaos is suppressed for large enough *c*.

### Quantification and Statistical Analysis

#### Dataset

We analyzed neural recordings from the V1 of two awake monkeys (Figures 4, 6, and 7). A full description of the experimental protocol and recordings can be found in the original publication (Ecker et al., 2010). We discarded all cells that were poorly isolated (contamination >5%), leaving us with 330 cells to analyze. The stimuli consisted of moving gratings of various orientations, all at 100% contrast. We fitted orientation tuning curves (Figure S11; average firing rate in the first 500 ms following stimulus onset, as a function of stimulus orientation) of the form $f\left(\theta \right)\equiv f_{0}+f_{\text{m}}\text{exp}\left[\kappa \left(\text{cos}\left(2\left(\theta -\theta_{\text{pref}}\right)\right)-1\right)\right]$, where θ is the stimulus orientation (thus, we neglected the direction of motion, which could be in either of the two directions orthogonal to the orientation of the grating). The fit was achieved using nonlinear least-squares regression.

For each neuron, we calculated an orientation tuning index (OTI), defined based on the fitted tuning curve as

(Equation 20) $$\text{OTI}=\frac{f\left(\theta_{\text{pref}}\right)-f\left(\theta_{\text{orth}}\right)}{f\left(\theta_{\text{pref}}\right)+f\left(\theta_{\text{orth}}\right)}\text{,}$$

where $\theta_{\text{orth}}=\theta_{\text{pref}}+\pi /2$. As the ring architecture we studied in Figures 4, 5, 6, and 7 only applied to neurons with well-defined tuning curves, we excluded cells that had OTI<0.75 as well as average evoked rates (measured during the stimulus period) below 1 spike/sec. This left us with 99 well-tuned cells to analyze.

Our analysis of the stimulus tuning of Fano factors and pairwise spike-count correlations was based on a time window of 100 ms starting at stimulus onset.

#### Factor analysis

We performed factor analysis of spike counts, either for a single stimulus condition in the model (the model had a natural rotational symmetry), or separately for each stimulus condition (direction) in the V1 dataset, subsequently averaging the reported quantities across conditions. We worked with normalized spike counts, defined as ${\tilde{c}}_{ik}=c_{ik}/{\sqrt{\langle c_{ik}\rangle }}_{k}$ where $c_{ik}$ is the spike count of neuron *i* in trial *k* and ${\langle \cdot \rangle }_{k}$ denotes averaging across trials. Note that the variances of these normalized spike counts are exactly the Fano factors, i.e., the usual measure of spike count variability. This prevented the normalized spike count covariance matrix $\tilde{C}$ from being contaminated by a rank-1 pattern of network covariance merely reflecting the tuning of single-neuron firing rates (the “Poisson” part of variability, which tends to scale with the mean count). Factor analysis decomposes $\tilde{C}$ as the sum of a rank-*k* covariance matrix ${\tilde{C}}_{\text{shared}}$ representing *k* modes of network covariances, and a diagonal matrix ${\tilde{C}}_{\text{private}}$. In the rate model, we could near-perfectly estimate the spike count covariance matrix, so we performed factor analysis by direct eigendecomposition of $\tilde{C}$, thus defining ${\tilde{C}}_{\text{shared}}=\sum_{i=1}^{k}\lambda_{i}v_{i}v_{i}^{⊤}$ whereby the top *k* eigenvectors $v_{1},\ldots ,v_{k}$ of $\tilde{C}$ contributed to shared variability in proportion of the corresponding eigenvalues $\lambda_{i}$. For factor analysis of the monkey V1 data, we performed direct maximization of the data likelihood (Cunningham and Ghahramani, 2015), also keeping *k* factors. In Figure 4, we set k=3, but we observed quenching of shared variability irrespective of *k* (Figure S12).

### Data and Software Availability

The code used for model simulations and data analysis is available from the Lead Contact, Dr Guillaume Hennequin, upon request.

## Supplemental Information

Document S1. Methods and Figures S1–S12

Document S2. Article plus Supplemental Information
