# Supplementary material for: The Dynamical Regime of Sensory Cortex: Stable Dynamics around a Single Stimulus-Tuned Attractor Account for Patterns of Noise Variability
Source: Neuron. 2018 May 16;98(4):846–860.e5. doi: 10.1016/j.neuron.2018.04.017 (PMC5971207; doi:10.1016/j.neuron.2018.04.017)
Supplement: Document S1. Methods and Figures S1–S12 [file mmc1.pdf]

Neuron, Volume *98*

Supplemental Information

The Dynamical Regime of Sensory Cortex: Stable Dynamics around a Single Stimulus-Tuned Attractor Account for Patterns of Noise Variability

Guillaume Hennequin, Yashar Ahmadian, Daniel B. Rubin, Máté Lengyel, and Kenneth D. Miller

The dynamical regime of sensory cortex: stable dynamics around a single stimulus-tuned attractor account for patterns of noise variability — Supplemental Methods —

Guillaume Hennequin, Yashar Ahmadian*, Daniel B. Rubin*,
Máté Lengyel† and Kenneth D. Miller†

*,† Equal contributions

Contents

Methods S1 Model setup **2**

Methods S2 Mean responses in the stabilized supralinear regime **2**

2.1 Input-dependence of mean responses . . . 2
2.2 The behavior of typical networks: numerical simulations . . . 4

Methods S3 Membrane potential variability in the two-population SSN model **5**

3.1 Linearization of the dynamics . . . 5
3.2 General result . . . 6
3.3 Analysis in simplified scenarios . . . 7
3.4 Effects of input correlations . . . 10
3.5 Mechanisms of variability modulation: Schur decomposition . . . 10
3.6 How do shear and restoring flow fields depend on the input? . . . 13

Methods S4 Firing rate and spike count variability **14**

4.1 Generic results in the SSN regime . . . 14
4.2 The specific regime of Kanashiro et al. (2017) . . . 14

Supplemental Figures **18**

Figure S1 . . . 18
Figure S2 . . . 19
Figure S3 . . . 20
Figure S4 . . . 21
Figure S5 . . . 22
Figure S6 . . . 23
Figure S7 . . . 24
Figure S8 . . . 25
Figure S9 . . . 26
Figure S10 . . . 27
Figure S11 . . . 28
Figure S12 . . . 29

Methods S1 Model setup

We consider the stochastic and nonlinear rate model of Equations 2 and 3 of the main text. To simplify notation, we assume $V_{\text{rest}} = 0$ mV without loss of generality as it can be absorbed in the external input, and rewrite:

$$\tau_{\text{E}}\,\mathbf{T}\,\frac{\mathrm{d}\mathbf{V}}{\mathrm{d}t} = -\mathbf{V}(t) + k\,\mathbf{W}\,\lfloor \mathbf{V}(t) \rfloor_+^n + \mathbf{h}(t) + \boldsymbol{\eta}(t) \tag{S1}$$

with $n > 1$ ($n = 2$ throughout the main text). In Equation S1, $\lfloor \mathbf{x} \rfloor_+^n$ denotes the pointwise application of the threshold power-law nonlinearity to the vector $\mathbf{x}$, that is, $\lfloor \mathbf{x} \rfloor_+^n$ is the vector whose i^{th} element is x_i^n if $x_i > 0$, or 0 otherwise; $\mathbf{T}$ is a diagonal matrix of relative membrane time constants measured in units of τ_{E}; $\mathbf{W}$ is a matrix of synaptic connections, consisting of N_{E} positive columns (corresponding to excitatory presynaptic neurons) and N_{I} negative columns (inhibitory neurons) for a total size of $N = N_{\text{E}} + N_{\text{I}}$; $\mathbf{h}(t)$ is a possibly time-varying but deterministic external input to neuron i; and $\boldsymbol{\eta}$ is a multivariate Ornstein-Uhlenbeck process with separable spatiotemporal correlations given by

$$\langle \boldsymbol{\eta}(t)\,\boldsymbol{\eta}(t+\tau) \rangle_t = e^{-|\tau|/\tau_\eta}\,\boldsymbol{\Sigma}_\eta \tag{S2}$$

where $\boldsymbol{\Sigma}_\eta$ is the covariance matrix of the input noise and τ_η is its correlation time. In particular, we are going to study how τ_η and correlations in $\boldsymbol{\Sigma}_\eta$ affect network variability. We adopt the following notations for relative time constants:

$$\bar{\tau}_{\text{I}} \equiv \frac{\tau_{\text{I}}}{\tau_{\text{E}}} \quad \text{and} \quad \bar{\tau}_\eta \equiv \frac{\tau_\eta}{\tau_{\text{E}}} \tag{S3}$$

In general, recurrent processing in the network is prone to instabilities due to the expansive, non-saturating V_{m}-rate relationship in single neurons. However, there are generous portions of parameter space in which inhibition dynamically stabilizes the network. We refer to this case as the "stabilized supralinear network", or SSN (Ahmadian et al., 2013; Rubin et al., 2015).

Methods S2 Mean responses in the stabilized supralinear regime

2.1 Input-dependence of mean responses

Our analysis of the stochastic SSN developed in Methods S3 will show that the modulation of variability relies on the nonlinear behavior of *mean* responses to varying inputs (Figure 2D of the main text), which in turn were studied previously (Ahmadian et al., 2013). We repeat these analyses here for completeness focusing in particular on the transition from superlinear integration of small inputs to sublinear responses to larger inputs. Note that here we have written the circuit dynamics in voltage form (Equation S1), while Ahmadian et al., 2013 chose a slightly different rate form; accordingly, the equations we now derive differ from the original equations in their form, but not in their nature (in fact, steady state solutions studied in Ahmadian et al., 2013 are mathematically equivalent in the two formulations, and moreover when $\mathbf{T}$ is proportional to the identity matrix, dynamic solutions are also exactly equivalent; see Miller and Fumarola, 2012).

As this section is devoted to mean responses, we neglect the input noise $\boldsymbol{\eta}$ for now. We thus write the deterministic dynamics of the mean potentials $\overline{V}_i$ as

$$\tau_{\text{E}}\,\mathbf{T}\,\frac{\mathrm{d}\overline{\mathbf{V}}}{\mathrm{d}t} = -\overline{\mathbf{V}} + k\,\mathbf{W}\,\lfloor \overline{\mathbf{V}} \rfloor_+^n + h\,\mathbf{g} \tag{S4}$$

and ask how neurons collectively respond to a constant external stimulus h fed to them through a vector $\mathbf{g} \sim \mathcal{O}(1)$ of feedforward weights. After some transient, and assuming the network is stable (see below), the network settles in a steady state $\overline{\mathbf{V}}$ which must obey the following fixed point equation, obtained by setting the l.h.s. of Equation S4 to zero:

$$\overline{\mathbf{V}} = h\,\mathbf{g} + k\,\mathbf{W}\,\lfloor \overline{\mathbf{V}} \rfloor_+^n \tag{S5}$$

As in the main text, we focus on the case of a threshold-quadratic nonlinearity, $n = 2$, though the following derivations can be extended to arbitrary $n > 1$. Following Ahmadian et al. (2013), we begin by defining $\mathbf{J} \equiv \mathbf{W}/\psi$ where $\psi = \|\mathbf{W}\|$ for some matrix norm $\|\cdots\|$, so that the dimensionless vector $\mathbf{J}$ has $\|\mathbf{J}\| = 1$. We also define dimensionless mean voltage and input respectively as

$$\bar{\mathbf{y}} \equiv 2\,k\,\psi\,\bar{\mathbf{V}} \tag{S6}$$

$$\alpha \equiv 2\,k\,\psi\,h \tag{S7}$$

(note that the definition of α differs from that in Ahmadian et al., 2013 by a factor of 2). With these definitions, and $n = 2$, the fixed point equation for the mean potentials, Equation S5, becomes

$$\bar{\mathbf{y}} = \alpha\,\mathbf{g} + \frac{1}{2}\mathbf{J}\,\lfloor\bar{\mathbf{y}}\rfloor_+^2 \tag{S8}$$

Network responses to small inputs When α is small (i.e. h is small, given fixed connectivity strength ψ), it is easy to see that

$$\bar{\mathbf{y}} \approx \alpha\,\mathbf{g} + \mathcal{O}(\alpha^2) \tag{S9}$$

In essence, the fixed point Equation S8 is already the first-order Taylor expansion of $\bar{\mathbf{y}}$ for small α (indeed, the recurrent term $\mathbf{J}\,\lfloor\bar{\mathbf{y}}\rfloor_+^2$ is $\mathcal{O}(\alpha^2)$, self-consistently). Thus, for small input α, membrane potentials scale linearly with α, and firing rates are quadratic in α, merely reflecting the single-neuron nonlinearity. In other words, the network behaves mostly as a relay of its feedforward inputs, with only minor corrections due to recurrent interactions.

More generally, by repeatedly substituting the right side of Equation S8 for $\bar{\mathbf{y}}$ into Equation S8, we arrive at the expansion

$$\bar{\mathbf{y}} = \alpha\,\mathbf{g} + \frac{1}{2}\mathbf{J}\left\lfloor \alpha\,\mathbf{g} + \frac{1}{2}\mathbf{J}\left\lfloor \alpha\,\mathbf{g} + \frac{1}{2}\mathbf{J}\,\lfloor\cdots\rfloor_+^2 \right\rfloor_+^2 \right\rfloor_+^2 \tag{S10}$$

The net result involves a series of terms of order α, α^2, α^4 …, which can be expected to converge for small α ($\alpha \ll 1$).

Network responses to larger inputs For large α ($\alpha \gg 1$), the expansion of Equation S10 will not converge and so cannot describe responses. Physically this tends to correspond to the excitatory subnetwork becoming unstable by itself. At the level of the fixed point Equation S8, recurrent processing involves squaring $\bar{\mathbf{V}}$, passing it through the recurrent connectivity, adding the feedforward input, squaring the result again, …, which for large enough input and purely excitatory connectivity would yield activity that grows arbitrarily large. A finite-activity solution is achieved through stabilization by inhibitory feedback. Mathematically, for this to occur, the recurrent term $\mathbf{J}\,\lfloor\bar{\mathbf{y}}\rfloor_+^2$ must cancel the linear dependence of $\bar{\mathbf{y}}$ on α in Equation S8 (since any linear dependence would be squared by the right side of Equation S8, then squared again, …, to yield an explosive series as in Equation S10). That is, we must have

$$\frac{1}{2}\mathbf{J}\,\lfloor\bar{\mathbf{y}}\rfloor_+^2 = -\alpha\,\mathbf{g} + \mathcal{O}(\sqrt{\alpha}) \tag{S11}$$

such that (again from Equation S8)

$$\bar{\mathbf{y}} \sim \mathcal{O}(\sqrt{\alpha}) \tag{S12}$$

at most. This means that membrane potentials scale at most as $\sqrt{\alpha}$, i.e. firing rates scale at most linearly in α. However, in many cases, firing rates too will be sublinear in α. This is best exemplified in the context of our two-population E/I model, by following Ahmadian et al. (2013) and introducing the notation:

$$\Omega_\mathrm{E} \equiv \left(-\mathbf{J}^{-1}\,\mathbf{g}\right)_\mathrm{E} \mathrm{Det}\,\mathbf{J} = J_\mathrm{II}\,g_\mathrm{E} - J_\mathrm{EI}\,g_\mathrm{I} \tag{S13}$$

$$\Omega_\mathrm{I} \equiv \left(-\mathbf{J}^{-1}\,\mathbf{g}\right)_\mathrm{I} \mathrm{Det}\,\mathbf{J} = J_\mathrm{IE}\,g_\mathrm{E} - J_\mathrm{EE}\,g_\mathrm{I} \tag{S14}$$

(note that we only consider networks in which $\text{Det}\,\mathbf{J} > 0$, as it must for stabilization to occur for all input levels α; Ahmadian et al., 2013). Equation S11 can then be rewritten as

$$\lfloor \overline{\mathbf{y}} \rfloor_+^2 = \frac{2\,\alpha}{\text{Det}\,\mathbf{J}} \begin{pmatrix} \Omega_E \\ \Omega_I \end{pmatrix} + \mathcal{O}(\sqrt{\alpha}) \tag{S15}$$

Now, depending on the choice of parameters (recurrent weights $\mathbf{J}$ and feedforward weights $\mathbf{g}$), Ω_E in particular can be negative. Since $\lfloor \overline{y}_E \rfloor_+^2$ is positive, it must be that the sublinear term $\mathcal{O}(\sqrt{\alpha})$ dominates over the (negative) linear term $2\,\Omega_E\,\alpha/\text{Det}\,\mathbf{J}$, at least over some range of α over which the E firing rate is non-zero. In this case, $\lfloor \overline{y}_E \rfloor_+^2$ behaves roughly as $\sqrt{\alpha}$ over some range[1] before it gets pushed to zero, and accordingly $\overline{y}_E$ must be approximately $\sqrt{\sqrt{\alpha}}$ over the same range, i.e. the E unit responds strongly sublinearly. Ahmadian et al. (2013) referred to this regime of eventual decrease of $\overline{y}_E$ with increasing stimulus strength as "supersaturation", and showed that it occurs for physiologically plausible parameter regimes. Our choice of parameters for the two-population model of the main text falls within this class of strongly sublinear E responses ($\Omega_E < 0$), but we will show in Methods S3 that the SSN displays the same input modulation of variability irrespective of the sign of Ω_E.

In summary, the SSN responds superlinearly to small inputs, and sublinearly to larger inputs. Firing rates become at most linear (but will be sublinear if $\Omega_E < 0$) with large inputs. Accordingly, membrane potentials show a transition from linear to (potentially strongly) sublinear responses to increasing inputs. Moreover, this transition occurs for $\alpha \sim \mathcal{O}(1)$.

2.2 The behavior of typical networks: numerical simulations

In the context of the reduced two-population model of the main text, we now complement the above theoretical arguments with a numerical analysis of the SSN's responses across a wide range of parameters, in order to form a picture of the "typical" behavior of the SSN in physiologically realistic regimes. We will later (Methods S3) reuse these numerical explorations to show that the modulation of variability by external input in the SSN is robust to changes of parameters.

The dynamics of the trial-averaged dimensionless "population voltages" are given by

$$\begin{aligned} \tau_E\,\dot{\overline{y}}_E &= -\overline{y}_E + \frac{1}{2}\left(J_{EE}\,\lfloor \overline{y}_E \rfloor_+^2 - J_{EI}\,\lfloor \overline{y}_I \rfloor_+^2\right) + \alpha\, g_E \\ \tau_I\,\dot{\overline{y}}_I &= -\overline{y}_I + \frac{1}{2}\left(J_{IE}\,\lfloor \overline{y}_E \rfloor_+^2 - J_{II}\,\lfloor \overline{y}_I \rfloor_+^2\right) + \alpha\, g_I \end{aligned} \tag{S16}$$

It is difficult to get good estimates of the values of the 6 free parameters (feedforward weights and recurrent weights) directly from biology. Therefore, our approach is to construct a large number of networks by randomly sampling these parameters within broad intervals, and rejecting those networks that produce unphysiological responses according to conservative criteria that we detail below. We then examine the behavior of each of these networks and perform statistics on the various kinds of responses that have been identified in the theoretical analysis of 2.1.

We thus constructed 1000 networks by sampling both feedforward weights $\{g_\alpha\}$ and recurrent weights $\{J_{\alpha\beta}\}$ (for $\alpha, \beta \in \{E, I\}$) uniformly from the interval [0.1; 1], and subsequently normalizing their (vector) L_∞-norm such that $\max(g_\alpha) = \max(J_{\alpha\beta}) = 1$. We then sampled the overall connectivity strength ψ (cf. 2.1) from the interval [0.1; 10]. This interval was based on rough estimates of the average number of input connections from the local network per neuron (between 200 and 1000), average PSP amplitude (between 0.1 mV and 0.5 mV) and decay time constants (5 to 20 ms), giving a range of connectivity strengths – which in our model is the product of these three quantities – between 0.1 and 10 mV/Hz.

Instead of choosing a range of α and simulating the dynamics of Equation S16 to compute mean voltages, we observed that $\overline{y}_I$ increases monotonically with α and for each network we chose a range

[1] Arguments about how $\overline{y}_E$ scales with large α actually become invalid when $\Omega_E < 0$ precisely because for large enough α the E unit stops firing; but the point here is that because $\overline{y}_E$ must decrease at some point, it will necessarily become strongly sublinear in α over some range before it starts to decrease.

of $\overline{y}_\mathrm{I}$ corresponding to mean I firing rates ($(\overline{y}_\mathrm{I}/2\,\psi)^2/k$) in the 0–200 Hz range, thus assuming that mean I responses above 200 Hz would be unphysiological. For each $\overline{y}_\mathrm{I}$ in this discretized range we solved for $\overline{y}_\mathrm{E}$ analytically by noting that the input α can be eliminated from the pair of fixed-point equations (Equation S16 with l.h.s. set to zero), yielding a fixed-point curve in the $(\overline{y}_\mathrm{E}, \overline{y}_\mathrm{I})$ plane:

$$\Omega_\mathrm{I}\,\overline{y}_\mathrm{E}^{\,2} + 2\,g_\mathrm{I}\,\overline{y}_\mathrm{E} = \Omega_\mathrm{E}\,\overline{y}_\mathrm{I}^{\,2} + 2\,g_\mathrm{E}\,\overline{y}_\mathrm{I} \tag{S17}$$

Given $\overline{y}_\mathrm{I}$ it is easy to solve this quadratic equation for $\overline{y}_\mathrm{E}$. We rejected those parameters sets for which we encountered either i) complex solutions for $\overline{y}_\mathrm{E}$, or ii) real but unstable solutions, as assessed by the stability conditions $\mathrm{Tr}\,\mathcal{J} < 0$ and $\mathrm{Det}\,\mathcal{J} > 0.01$ (with the Jacobian matrix $\mathcal{J}$ defined in Equations S19 and S22), or iii) stable solutions that involved E firing rates ($(\overline{y}_\mathrm{E}/2\,\psi)^2/k$) either greater than 200 Hz, or smaller than 1 Hz for the largest value of $\overline{y}_\mathrm{I}$. Finally, for each fixed point $(\overline{y}_\mathrm{E}, \overline{y}_\mathrm{I})$, we computed the corresponding α from either of the two fixed-point equations (Equation S16 with l.h.s. set to zero), e.g. $\alpha = \left[\overline{y}_\mathrm{E} - (J_\mathrm{EE}\,\overline{y}_\mathrm{E}^{\,2} - J_\mathrm{EI}\,\overline{y}_\mathrm{I}^{\,2})/2\right]/g_\mathrm{E}$. This procedure was numerically much more efficient than simulating the dynamics of Equation S16 until convergence to steady-state.

The parameters of the retained networks spanned a large chunk of the intervals in which they were sampled (Figure S1A and B). Because stability for large α requires $\mathrm{Det}\,\mathbf{J} > 0$, i.e. $J_\mathrm{EI}\,J_\mathrm{IE} > J_\mathrm{EE}\,J_\mathrm{II}$, the largest of all sampled $J_{\alpha\beta}$'s was often either J_EI or J_IE which then, due to the L_∞-norm normalization, assumed a value of one (Figure S1A). We also observed that the input weight g_E was often larger than g_I (Figure S1B). About 90% of the sampled networks had $\Omega_\mathrm{E} > 0$, implying $\sim\sqrt{\alpha}$ scaling of $\overline{y}_\mathrm{E}$ and $\overline{y}_\mathrm{I}$ for large α (example in Figure S1D, top). In these networks, E and I rates were linear in α for α large enough, and so were also linear in each other when large enough (Figure S1E, black). The rest of the networks (10%) had $\Omega_\mathrm{E} < 0$ and therefore showed supersaturation of the E firing rate for large input (Figure S1D, bottom) and E responses that were sublinear in I responses (Figure S1E, orange).

It is worth noting that for networks with small overall connectivity strength ψ, the proportion of $\Omega_\mathrm{E} < 0$ and $\Omega_\mathrm{E} > 0$ cases tend to even out (Figure S1C). This is because, for supersaturating networks, the peak E firing rate is inversely proportional to ψ^2 (Ahmadian et al., 2013), so for large ψ the peak firing rate is low and therefore the final value of $\overline{r}_\mathrm{E}$ reached for $\overline{r}_\mathrm{I} = 200$ Hz likely falls below our threshold of 1 Hz, resulting in a rejection of the parameter set.

In sum, the nonlinear properties of the SSN's responses to growing inputs, summarized in 2.1, are robust to changes in parameters so long as these keep the network in a regime "not too unphysiological" in a conservative sense. Using the same collection of sampled networks, we will show below that the modulation of variability with input described in the main text is equally robust to parameter changes.

Methods S3 Membrane potential variability in the two-population SSN model

In this section, we derive the theoretical results regarding activity variability in the two-population model of the main text. We use these analytical results to demonstrate robustness of our results to changes in parameters, which we also verify numerically using the collection of networks with randomly sampled parameters introduced in 2.2.

3.1 Linearization of the dynamics

We now consider the noisy dynamics of the two-population model of the main text in which the E and I units represent the average activity of large E and I populations. To study variability analytically, we linearize Equation S1 around the mean, thus examining the local behavior of small fluctuations $\delta\mathbf{V}$:

$$\tau_\mathrm{E}\,\mathbf{T}\,\frac{\mathrm{d}\delta\mathbf{V}}{\mathrm{d}t} = \mathbf{A}(\alpha)\,\delta\mathbf{V}(t) + \boldsymbol{\eta}(t) \tag{S18}$$

$$\text{with}\quad \mathbf{A}(\alpha) \equiv -\mathbf{I} + \mathbf{W}^\mathrm{eff}(\alpha) \tag{S19}$$

The effective connectivity $\mathbf{W}^{\text{eff}}$ depends on the (dimensionless) input α through its dependence on mean responses, following

$$W_{ij}^{\text{eff}}(\alpha) = n\,k\,W_{ij}\,\lfloor \overline{V}_j(\alpha)\rfloor_+^{n-1} \qquad \text{for} \quad i,j \in \{\text{E},\text{I}\} \tag{S20}$$

For $n = 2$, Equation S20 can also be written using the definition of the dimensionless voltage $\bar{\mathbf{y}}$ and dimensionless connections $\mathbf{J}$ introduced in 2.1 as

$$W_{ij}^{\text{eff}}(\alpha) = J_{ij}\,\lfloor \bar{y}_j(\alpha)\rfloor_+ \tag{S21}$$

With our notations, the Jacobian matrix

$$\mathcal{J}(\alpha) \equiv \mathbf{T}^{-1}\,\mathbf{A}(\alpha) \tag{S22}$$

is unitless, so that, e.g., the interpretation of a real negative eigenvalue λ of $\mathcal{J}$ is that the corresponding eigenmode decays asymptotically with time constant $\tau_{\text{E}}/|\lambda|$ as a result of the recurrent dynamics. We parameterize the input noise covariance as

$$\left\langle \boldsymbol{\eta}(t)\,\boldsymbol{\eta}(t+\tau)^{\mathsf{T}}\right\rangle = \left(1+\frac{1}{\bar{\tau}_\eta}\right)\,e^{-|\tau|/\tau_\eta}\begin{pmatrix} c_{\text{E}}^2 & c_{\text{EI}} \\ c_{\text{EI}} & c_{\text{I}}^2 \end{pmatrix} \qquad \text{with} \quad c_{\text{EI}} \equiv \rho_{\text{EI}}\,c_{\text{E}}\,c_{\text{I}} \tag{S23}$$

such that, in the limit of small α – in which the network is effectively unconnected, because $\lfloor \bar{\mathbf{y}} \rfloor$ in Equation S20 is small – the E unit has variance c_{E}^2; the I unit then has variance $\frac{1+\bar{\tau}_\eta}{\bar{\tau}_{\text{I}}+\bar{\tau}_\eta}c_{\text{I}}^2$. The parameter ρ_{EI} determines the correlation between input noise to the E and I units.

3.2 General result

The full output covariance matrix $\boldsymbol{\Sigma} \equiv \langle \delta\mathbf{V}\,\delta\mathbf{V}^{\mathsf{T}}\rangle$ can be calculated by solving a set of linear equations[2], which yields:

$$\boldsymbol{\Sigma} = \frac{(1+\bar{\tau}_\eta)(1-\bar{\tau}_\eta\,\text{Tr}\,\mathcal{J})}{-\text{Tr}\,\mathcal{J}\,\text{Det}\,\mathbf{A}\,(\bar{\tau}_{\text{I}} - \bar{\tau}_{\text{I}}\,\bar{\tau}_\eta\,\text{Tr}\,\mathcal{J} + \bar{\tau}_\eta^2\,\text{Det}\,\mathbf{A})}\begin{pmatrix} \Sigma_{\text{EE}}^\star & \Sigma_{\text{EI}}^\star \\ \Sigma_{\text{EI}}^\star & \Sigma_{\text{II}}^\star \end{pmatrix} \tag{S26}$$

with

$$\Sigma_{\text{EE}}^\star = c_{\text{E}}^2\left(\frac{\bar{\tau}_{\text{I}}\,\text{Det}\,\mathbf{A}}{1-\bar{\tau}_\eta\,\text{Tr}\,\mathcal{J}} + A_{\text{II}}^2\right) + c_{\text{I}}^2\,A_{\text{EI}}^2 - 2\,c_{\text{EI}}\,A_{\text{EI}}\,A_{\text{II}} \tag{S27}$$

$$\Sigma_{\text{II}}^\star = c_{\text{I}}^2\left(\frac{\bar{\tau}_{\text{I}}^{-1}\,\text{Det}\,\mathbf{A}}{1-\bar{\tau}_\eta\,\text{Tr}\,\mathcal{J}} + A_{\text{EE}}^2\right) + c_{\text{E}}^2\,A_{\text{IE}}^2 - 2\,c_{\text{EI}}\,A_{\text{IE}}\,A_{\text{EE}} \tag{S28}$$

$$\Sigma_{\text{EI}}^\star = c_{\text{E}}^2\,A_{\text{IE}}\,A_{\text{II}} + c_{\text{I}}^2\,A_{\text{EI}}\,A_{\text{EE}} - 2\,c_{\text{EI}}\left(A_{\text{EE}}\,A_{\text{II}} - \frac{\bar{\tau}_\eta\,\text{Tr}\,\mathcal{J}\,\text{Det}\,\mathbf{A}}{2\,(1-\bar{\tau}_\eta\,\text{Tr}\,\mathcal{J})}\right) \tag{S29}$$

---

[2] Since the spatial and temporal correlations in the noise term $\boldsymbol{\eta}$ in Equation S18 are separable, we can augment the state space with two noise units and write their (linear) Langevin dynamics as

$$\tau_{\text{E}}\,d\begin{pmatrix} \delta\mathbf{V} \\ \boldsymbol{\eta} \end{pmatrix} = \begin{pmatrix} \mathbf{A}(h) & \mathbf{I} \\ 0 & -\frac{\tau_{\text{E}}}{\tau_\eta}\mathbf{I} \end{pmatrix}\begin{pmatrix} \delta\mathbf{V} \\ \boldsymbol{\eta} \end{pmatrix} dt + \begin{pmatrix} 0 & 0 \\ 0 & \tau_{\text{E}}\sqrt{\frac{2}{\tau_\eta}}\mathbf{B} \end{pmatrix} d\boldsymbol{\xi} \tag{S24}$$

where $d\boldsymbol{\xi}$ is a unit-variance, spherical Wiener process, and $\mathbf{B}$ is the Cholesky factor of the desired noise covariance matrix, that is, $\boldsymbol{\Sigma}_\eta = \mathbf{B}\mathbf{B}^{\mathsf{T}}$ (the $\tau_{\text{E}}\sqrt{2/\tau_\eta}$ factor is such that this equality holds). Then, from multivariate Ornstein-Uhlenbeck process theory (e.g. Hennequin et al., 2014), we know that the covariance matrix of the compound process satisfies the following Lyapunov equation:

$$\begin{pmatrix} \mathbf{A} & \mathbf{I} \\ 0 & -\frac{\tau_{\text{E}}}{\tau_\eta}\mathbf{I} \end{pmatrix}\begin{pmatrix} \boldsymbol{\Sigma} & \boldsymbol{\Lambda} \\ \boldsymbol{\Lambda}^{\mathsf{T}} & \boldsymbol{\Sigma}_\eta \end{pmatrix} + \begin{pmatrix} \boldsymbol{\Sigma} & \boldsymbol{\Lambda} \\ \boldsymbol{\Lambda}^{\mathsf{T}} & \boldsymbol{\Sigma}_\eta \end{pmatrix}\begin{pmatrix} \mathbf{A}^{\mathsf{T}} & 0 \\ \mathbf{I} & -\frac{\tau_{\text{E}}}{\tau_\eta}\mathbf{I} \end{pmatrix} = -\begin{pmatrix} 0 & 0 \\ 0 & 2\frac{\tau_{\text{E}}}{\tau_\eta}\mathbf{B}\mathbf{B}^{\mathsf{T}} \end{pmatrix} \tag{S25}$$

where $\boldsymbol{\Sigma}$ is the covariance we are trying to compute. By vectorizing Equation S25, neglecting the bottom right quadrant (which by itself only confirms $\boldsymbol{\Sigma}_\eta = \mathbf{B}\mathbf{B}^{\mathsf{T}}$ as promised above), and taking into account the symmetry, one ends up with a system of 7 coupled but *linear* equations to solve for the 3 unknowns of $\boldsymbol{\Sigma}$ and the 4 unknowns of $\boldsymbol{\Lambda}$. This can be done by hand using some patience, or automatically using a symbolic solver such as Mathematica, and yields the expression in Equation S26.

In Equations S26 to S29, each term that depends on $\mathbf{A}$ or $\mathcal{J}$ depends implicitly on the (dimensionless) constant input α delivered to both E and I populations, because $\mathbf{A}$ (and $\mathcal{J}$) depends on mean voltages (through Equation S20) which themselves depend on α. Note also that, for the network to be stable at a given input level α, the Jacobian matrix $\mathcal{J}(\alpha)$ should obey $\mathrm{Tr}\,\mathcal{J} < 0$ and $\mathrm{Det}\,\mathcal{J} > 0$ (with the latter equivalent to $\mathrm{Det}\,\mathbf{A} > 0$).

Among other things, we will analyze the behaviour of the total variance, i.e. the trace of $\mathbf{\Sigma}$ given by

$$\mathrm{Tr}(\mathbf{\Sigma}) = (1 + \bar{\tau}_\eta)\,\frac{\beta(\mathbf{A})\,(1 - \bar{\tau}_\eta \mathrm{Tr}\,\mathcal{J}) + \mathrm{Det}\,\mathbf{A}\,(\bar{\tau}_\mathrm{I}\, c_\mathrm{E}^2 + \bar{\tau}_\mathrm{I}^{-1}\, c_\mathrm{I}^2)}{-\mathrm{Tr}\,\mathcal{J}\ \mathrm{Det}\,\mathbf{A}\left(\bar{\tau}_\mathrm{I} - \bar{\tau}_\mathrm{I}\,\bar{\tau}_\eta\,\mathrm{Tr}\,\mathcal{J} + \bar{\tau}_\eta^2\,\mathrm{Det}\,\mathbf{A}\right)} \tag{S30}$$

with $\mathbf{A}$ defined in Equation S19 and

$$\beta(\mathbf{A}) \equiv (A_\mathrm{IE}^2 + A_\mathrm{II}^2)\,c_\mathrm{E}^2 + (A_\mathrm{EI}^2 + A_\mathrm{EE}^2)\,c_\mathrm{I}^2 - 2\,(A_\mathrm{IE}\,A_\mathrm{EE} + A_\mathrm{EI}\,A_\mathrm{II})\,c_\mathrm{EI} \tag{S31}$$

3.3 Analysis in simplified scenarios

In order to understand what Equation S30 tells us about the modulation of variability with the input α, we make a couple of assumptions that greatly simplify the expression for the total variance with little loss of generality. First, we consider the limit of slow[3] input noise which we find empirically is approached rather fast, with $\tau_\eta = 50$ ms already giving a close approximation given $\tau_\mathrm{E} = 20$ ms and $\tau_\mathrm{I} = 10$ ms. Next, we assume that

$$c_\mathrm{E} = \frac{c_\mathrm{I}}{\kappa} \equiv c \tag{S32}$$

and $\rho_\mathrm{EI} = 0$ (implying $c_\mathrm{EI} = 0$), i.e. the E and I units have uncorrelated input fluctuations of equal amplitude (the impact of positive input correlations, $\rho_\mathrm{EI} > 0$, will be discussed in 3.4). With these two assumptions, the total variance simplifies into

$$\mathrm{Tr}(\mathbf{\Sigma}) = c^2\,\frac{\beta_0(\mathbf{A})}{\mathrm{Det}\,\mathbf{A}^2} = c^2\,\frac{A_\mathrm{IE}^2 + A_\mathrm{II}^2 + A_\mathrm{EI}^2 + A_\mathrm{EE}^2}{(A_\mathrm{EE}\,A_\mathrm{II} - A_\mathrm{EI}\,A_\mathrm{IE})^2} \tag{S33}$$

where we defined $\beta_0(\mathbf{A}) \equiv \beta(\mathbf{A})/c^2$.

There are two ways to understand how total variance scales with inputs. First, somewhat loosely and indirectly, via its scaling with mean responses. As mean voltage responses increase with the stimulus, so do the effective weights, which – for a large enough input and a general threshold-powerlaw input/output nonlinearity with exponent n – are proportional to $\bar{y}^{n-1}$ (Equation S20). As the numerator of Equation S33 is quadratic in $\mathbf{A}$ and thus also in the effective weights in the large input limit, while the denominator is quartic, the overall scaling is going to be inverse quadratic in the effective weights, yielding a total voltage variance which scales with mean responses approximately as

$$\mathrm{Tr}(\mathbf{\Sigma}) \propto 1/\bar{y}^{2(n-1)} \tag{S34}$$

Second, for the special case of a threshold-quadratic nonlinearity ($n = 2$), we can also understand the scaling of the total variance directly with the input strength, α, in more precise terms. The typical behavior of $\beta_0(\mathbf{A})^{1/2}$ and $\mathrm{Det}\,\mathbf{A}$ is shown in Figure S2A. Both can be expressed as functions of mean responses using Equations S19 and S20:

$$\beta_0(\mathbf{A}) = \kappa^2\,(J_\mathrm{EE}\,\bar{y}_\mathrm{E} - 1)^2 + \kappa^2\,(J_\mathrm{EI}\,\bar{y}_\mathrm{I})^2 + (J_\mathrm{IE}\,\bar{y}_\mathrm{E})^2 + (1 + J_\mathrm{II}\,\bar{y}_\mathrm{I})^2 \tag{S35}$$

$$\mathrm{Det}\,\mathbf{A}^2 = \left[(J_\mathrm{IE}\,\bar{y}_\mathrm{E})\,(J_\mathrm{EI}\,\bar{y}_\mathrm{I}) + (1 - J_\mathrm{EE}\,\bar{y}_\mathrm{E})\,(1 + J_\mathrm{II}\,\bar{y}_\mathrm{I})\right]^2 \tag{S36}$$

[3] The other limit (fast noise, $\tau_\eta \to 0$) also greatly simplifies Equation S30, but would not make much sense in the context of this study, since Equation S1 is meant to model the dynamics of the voltage on a timescale ≥ 30 ms, which is the timescale on which a threshold power-law relationship between voltage and rate has been measured in cat V1. Therefore, the input noise that we explicitly model here is meant to capture the slowly fluctuating components of external inputs, the fast components having been "absorbed" into the threshold power-law gain function.

Note that to simplify notations we have dropped the $\lfloor\cdot\rfloor_+$ that should surround every $\bar{y}$. Based on these expressions, we now examine the behavior of variability in the small and large α limits and show that the total variance should typically grow and then decay with increasing α, and therefore should exhibit a maximum which empirically we find occurs for $\alpha \sim 1$.

Behavior of the total variance for small α Using Equations S33, S35 and S36, we find the slope of the total variance at $\alpha = 0$ to be

$$\frac{\mathrm{d}}{\mathrm{d}\alpha}\mathrm{Tr}(\boldsymbol{\Sigma})\bigg|_{\alpha=0} = 2\,c^2\,\left(g_\mathrm{E}\,J_\mathrm{EE} - \kappa^2\,g_\mathrm{I}\,J_\mathrm{II}\right) \tag{S37}$$

Thus, when the noise power fed to inhibitory cells is sufficiently small, $\kappa = c_\mathrm{I}/c_\mathrm{E}$ will be small enough that the expression in Equation S37 will stay positive, and therefore total variability will grow with small increasing α. Indeed, we find that this happens for most (>90%) of the randomly sampled networks of 2.2 with κ as large as 1/2 (Figure S2A, bottom). Moreover, restricting the analysis to the E unit gives $\mathrm{d}\Sigma_\mathrm{EE}/\mathrm{d}\alpha|_{\alpha=0} = 2\,c^2\,g_\mathrm{E}\,J_\mathrm{EE}$ which is always positive, independently of κ. Thus, for slow enough input noise, the variability in the E unit always increases with small α.

We can extend this argument to slightly larger values of α by further inspecting the numerator and denominator in Equation S33. Although the first term in the numerator, $(J_\mathrm{EE}\,\bar{y}_\mathrm{E} - 1)^2$, originally decays with α as $\bar{y}_\mathrm{E}$ grows from 0 to $1/J_\mathrm{EE}$, the other three terms always grow with α as long as mean voltages do, and thus we expect the numerator to typically grow. This is indeed what we find in all sampled networks (Figure S2A). On the other hand, the denominator (Equation S36) is the square of the sum of two terms, the first one initially small and growing, and the second one initially large and decaying. Indeed, the second term starts at 1 for $\alpha = 0$, because the $\bar{y}$ terms are all zero, and then decays to zero as the network enters the inhibition-stabilized (ISN) regime and the effective excitatory feedback gain $J_\mathrm{EE}\,\bar{y}_\mathrm{E}$ becomes larger than one[4] (Tsodyks et al., 1997; Ozeki et al., 2009). Thus, due to this partial cancellation of growing and decaying terms, we expect the denominator to either decrease, or grow very slowly, with increasing α (Figure S2A), until it starts growing faster (see arguments below for the large α case) in the very rough neighborhood of the ISN transition. All in all, the ratio of a fast growing numerator to a slower growing denominator suggests that the total variance should robustly grow with small increasing α (Figure S2A, bottom).

Behavior of the total variance for large α As the input grows, so do the mean (dimensionless) voltages $\bar{y}_\mathrm{E}$ and $\bar{y}_\mathrm{I}$ at least over some range of α. Therefore, we expect *both* the numerator *and* the denominator that make up the total variance in Equation S33 to grow with large enough and increasing α. However, loosely speaking, the numerator grows as $\bar{y}^2$ while the denominator grows as $\bar{y}^4$, which can be seen by inspecting Equations S35 and S36. Thus, their ratio should decrease roughly as

$$\mathrm{Tr}(\Sigma) \propto \frac{1}{\bar{y}^2} \tag{S38}$$

which is just a special case for $n = 2$ of the generic result in Equation S34 for arbitrary n.

However, here (for $n = 2$) this argument can be made more rigorous in the case of $\Omega_\mathrm{E} > 0$, i.e. when the E unit does not supersaturate. In this case, from Equation S15 we have $\bar{y}_\mathrm{E} \approx \sqrt{2\,\Omega_\mathrm{E}\,\alpha/\mathrm{Det}\,\mathbf{J}}$ and $\bar{y}_\mathrm{I} \approx \sqrt{2\,\Omega_\mathrm{I}\,\alpha/\mathrm{Det}\,\mathbf{J}}$ for α large enough. Therefore, in the large α limit, the numerator and denominator of Equation S33 respectively behave as

$$\beta_0(\mathbf{A}) \approx \frac{2}{\mathrm{Det}\,\mathbf{J}}\left[(J_\mathrm{IE}^2 + \kappa^2\,J_\mathrm{EE}^2)\,\Omega_\mathrm{E} + (J_\mathrm{II}^2 + \kappa^2\,J_\mathrm{EI}^2)\,\Omega_\mathrm{I}\right]\alpha \tag{S39}$$

$$\mathrm{Det}\,\mathbf{A}^2 \approx 4\Omega_\mathrm{E}\,\Omega_\mathrm{I}\,\alpha^2 \tag{S40}$$

[4] In this regime, $J_\mathrm{EE}\,\bar{y}_\mathrm{E} > 1 \Leftrightarrow A_\mathrm{EE} > 0$ implies instability of the excitatory subnetwork in isolation, and therefore the need for dynamic, stabilizing feedback inhibition (hence the name "inhibition-stabilized network").

therefore the total variance (their ratio) decreases as $1/\alpha$. For $\Omega_E < 0$, the large α limit is irrelevant strictly speaking, as in this limit $\lfloor \bar{y}_E \rfloor_+$ and $\bar{r}_E$ go to zero. In this case the total variance does not decrease asymptotically but reaches a finite limit of $c^2 \left[1 + (\bar{\tau}_I J_{EI}/J_{II})^2\right]$. However, we find empirically that the peak of variability always occurs well before the onset of supersaturation, in a regime where both $\bar{y}_E$ and $\bar{y}_I$ are still growing with α while remaining roughly proportional to each other (Figure S1E), so that the argument made above can be repeated: the total variance decreases as $1/\bar{y}^2$ for a while after having peaked.

Where does variability peak? The above arguments, derived for slow noise $\tau_\eta \to \infty$, show that growing inputs typically increase, and then suppress, total variability in the two-population SSN. Thus, total variability (and even more certainly, variability in the E unit) typically exhibits a maximum for some intermediate value of α. We find empirically that, even for finite τ_η, the location of this variance peak is well approximated by its location in the limit of fast inhibition, $\bar{\tau}_I \to 0$, which we can estimate analytically. Indeed, in this limit, the I cell responds instantaneously to changes in E activity and input noise, such that

$$\delta V_I(t) = \frac{J_{IE}\, \bar{y}_E\, \delta V_E(t) + \eta_I(t)}{1 + J_{II}\, \bar{y}_I} \tag{S41}$$

Consequently, δV_E now obeys one-dimensional dynamics given by

$$\tau_E\, \delta\dot{V}_E = -\lambda\, \delta V_E(t) + \eta_{\text{eff}}(t) \tag{S42}$$

where

$$\lambda = 1 + \frac{\bar{y}_E\, (\text{Det}\,\mathbf{J}\, \bar{y}_I - J_{EE})}{1 + J_{II}\, \bar{y}_I} \tag{S43}$$

and η_{eff} is a noise process (a linear combination of η_E and η_I) with temporal correlation length τ_η and a variance that is empirically irrelevant for the arguments below[5]. In this case, the variance of δV_E is inversely proportional to $\lambda\,(\frac{1}{\bar{\tau}_\eta} + \lambda)$, and therefore should be maximum at the input level α that minimizes λ. Observing from Figure S1E that $\bar{y}_E$ and $\bar{y}_I$ are roughly proportional over a large range of α (for $\Omega_E < 0$), if not the entire range (for $\Omega_E > 0$), we can make the following approximation:

$$\lambda - 1 \propto \frac{\bar{y}_I\, (\text{Det}\,\mathbf{J}\, \bar{y}_I - J_{EE})}{1 + J_{II}\, \bar{y}_I} \tag{S44}$$

whose minimum is straightforward to calculate and is attained for

$$\bar{y}_I = \frac{1}{J_{II}} \left(\sqrt{\frac{J_{EI}\, J_{IE}}{\text{Det}\,\mathbf{J}}} - 1 \right) \tag{S45}$$

We find that the α of maximum variance in the E unit is indeed very well approximated by the α at which $\bar{y}_I$ reaches the threshold value of Equation S45, especially in the absence of input correlations ($\rho_{EI} = 0$, Figure S2B, left). For correlated noisy inputs, the criterion of Equation S45 deteriorates slightly but still consistently provides an upper bound on the α of maximum E variance (Figure S2B, right).

Interestingly, the criterion for maximum variance in Equation S45 is equivalent to a criterion about the effective I→I connection, given by $W_{II}^{\text{eff}} \equiv 2\,k\,\lfloor \bar{V}_I \rfloor_+ W_{II}$ (cf. Equation 1 in main text). Specifically, at the peak of variance we expect to have

$$W_{II}^{\text{eff}} = \sqrt{\frac{1}{1-\beta}} - 1 \qquad \text{with } \beta \equiv \frac{W_{EE}\, W_{II}}{W_{EI}\, W_{IE}} \tag{S46}$$

where $\beta < 1$ is in some sense the ratio of what contributes positively to the activity of the E cell (product of self-excitation W_{EE} with disinhibition W_{II}) to what contributes negatively to it (the product

[5] The variance of the effective noise process is proportional to $1 + \frac{J_{IE}^2\, \bar{y}_I^{\,2}}{(1+J_{II}\, \bar{y}_I)^2}$, and so has some dependence on α especially for small α before $\bar{y}_I$ grows large. However, empirically, the quality of the approximation in Equation S44 – which is derived under the assumption of constant effective noise variance – suggests that we can neglect this effect.

$W_{\text{IE}}\,W_{\text{EI}}$ quantifying the strength of the E $\to$ I $\to$ E inhibitory feedback loop). Thus, in networks with inhibition-dominated connectivity, i.e. ones in which $\beta \ll 1$, we expect $W_{\text{II}}^{\text{eff}}$ to reach the criterion of Equation S46 earlier as the input grows (this argument implictly assumes that the rate of growth of $W_{\text{II}}^{\text{eff}}$ itself doesn't depend too much on β, which we could confirm numerically).

Finally, we note that since variability peaks for $\alpha \sim \mathcal{O}(1)$ and $y \sim \mathcal{O}(1)$, networks with stronger connectivity (large ψ) will exhibit a peak of variance for smaller external input h (because $\alpha \propto \psi\,h$) – and this peak will occur for lower voltages/firing rates (because $\overline{V} \propto y/\psi$).

3.4 Effects of input correlations

To see the effect of input correlations on variability, we return to the expression for Σ_{EE} in Equation S30, assume again that $\tau_\eta \to \infty$ and $c_{\text{E}} = \frac{c_{\text{I}}}{\kappa} = c$, but now with $\rho_{\text{EI}} \neq 0$. We thus obtain:

$$\Sigma_{\text{EE}} = c^2\,\frac{A_{\text{II}}^2 + \kappa^2\,A_{\text{EI}}^2}{\text{Det}\,\mathbf{A}^2} - 2\,c^2\,\rho_{\text{EI}}\,\frac{\kappa\,A_{\text{II}}\,A_{\text{EI}}}{\text{Det}\,\mathbf{A}^2} \tag{S47}$$

Thus, total E variability is equal to that without input correlation (the first term), minus a positive term proportional to ρ_{EI}. Thus, positive input correlations always decrease variability in the E unit (and, in particular, its peak; Figure S2C, right), while negative correlations increase it. Moreover, the subtracted term has the same large-α behavior as the first term, because the two terms share the same denominator and for large alpha both numerators are $\mathcal{O}(\overline{y}_{\text{I}}^{\,2})$. Thus, input correlations should not affect the qualitative, decreasing behaviour of E variance for large increasing inputs. For small α and large ρ_{EI}, however, we expect $A_{\text{II}}^2+\kappa^2\,A_{\text{EI}}^2-2\,\rho_{\text{EI}}\,\kappa\,A_{\text{II}}\,A_{\text{EI}}$ to grow much more slowly than $A_{\text{II}}^2+\kappa^2\,A_{\text{EI}}^2$; and indeed, in the extreme case $\rho_{\text{EI}} = 1$, the total numerator becomes $(1 + (J_{\text{II}} - \kappa\,J_{\text{EI}})\,\overline{y}_{\text{I}})^2$, which can even decrease transiently with increasing α if $\kappa\,J_{\text{EI}} > J_{\text{II}}$ (this occurs in about half of our thousand networks). This, in effect, shifts the peak of E variability to smaller values of α (Figure S2C, left).

The situation for the I unit is a bit different, as input correlations affect the I variance differently depending on whether the network has already made the transition to the ISN regime. Indeed, under the same assumptions as above, the I variance is given by

$$\Sigma_{\text{II}} = c^2\,\frac{\kappa^2\,A_{\text{EE}}^2 + A_{\text{IE}}^2}{\text{Det}\,\mathbf{A}^2} - 2\,c^2\,\rho_{\text{EI}}\,\frac{\kappa\,A_{\text{EE}}\,A_{\text{IE}}}{\text{Det}\,\mathbf{A}^2} \tag{S48}$$

In the ISN regime, $A_{\text{EE}} > 0$, so that input correlations decrease I variability, just as they do for E variability as seen above. For small enough inputs, however, the network is not yet an ISN ($A_{\text{EE}} < 0$), so that the effect of correlations is reversed: larger input correlations increase I variability.

In sum, input correlations modify the fine details of how large the variance grows and how early it peaks with increasing inputs, but they do not modify the qualitative aspects – in particular, the non-monotonic behavior – of variability modulation with external inputs in this two-population SSN model.

3.5 Mechanisms of variability modulation: Schur decomposition

We now unpack the mechanistic aspects of variability modulation in the SSN, by decomposing the effects of effective connectivity into two qualitatively different flow fields that shape the covariance of activities in the network in distinct ways (Figure S3): "shear" and "restoring" fields. To do this, we focus on the linearized dynamics of Equation S18 and perform a Schur decomposition of the Jacobian matrix in Equation S22 (which includes both the single-neuron leak and the effective connectivity; Murphy and Miller, 2009):

$$\mathcal{J}(\alpha) = \mathbf{U}(\alpha)\,\mathbf{T}_{\text{Schur}}(\alpha)\,\mathbf{U}(\alpha)^\star \qquad \text{with} \quad \mathbf{T}_{\text{Schur}}(\alpha) \equiv \begin{pmatrix} \lambda_{\text{s}} & w_{\text{FF}} \\ 0 & \lambda_{\text{d}} \end{pmatrix} \tag{S49}$$

where $\cdot^\star$ denotes the conjugate transpose, $\boldsymbol{\lambda}_\mathrm{s}$ and $\boldsymbol{\lambda}_\mathrm{d}$ are the two (either real or complex-conjugate[6]) eigenvalues of $\mathcal{J}(\alpha)$, the columns of $\mathbf{U}$ are the (orthonormal) Schur vectors such that $\mathbf{U}\,\mathbf{U}^\star = \mathbf{U}^\star\,\mathbf{U} = \mathbf{I}$, and $\mathbf{w}_\mathrm{FF}$ is the feedforward weight coupling the dynamics of the Schur vectors. Expressing the E and I voltage fluctuations in the Schur basis as $\mathbf{z} \equiv \mathbf{U}^\star\,\delta\mathbf{V}$, their dynamics become

$$\tau_\mathrm{E}\,\frac{\mathrm{d}\mathbf{z}}{\mathrm{d}t} = \mathbf{T}_\mathrm{Schur}\,\mathbf{z} + \mathbf{U}^\star\,\mathbf{T}^{-1}\,\boldsymbol{\eta} \tag{S50}$$

In the case of the 2-population E/I architecture considered here ($\mathbf{W}$ given by Equation 8 of the main text), the first Schur vector is a "sum mode" in the generalized sense (Murphy and Miller, 2009), i.e. its excitatory and inhibitory components have the same sign[7]. This corresponds to patterns of network activity in which the excitatory and inhibitory units are simultaneously either more active or less active than average. The second Schur mode is a generalized "difference mode" in that its excitatory and inhibitory components have opposive signs. (Hence the notations $\boldsymbol{\lambda}_\mathrm{s}$ and $\boldsymbol{\lambda}_\mathrm{d}$.) In theory, $\mathbf{U}$ depends on the input α, because $\mathcal{J}$ does. However, we find that past a relatively small value of α, the Schur vectors do not change much and are indeed sum-like and difference-like across all thousand networks studied in Methods S2 and Methods S3 (Figure S2E).

The Schur decomposition reveals through $\mathbf{T}_\mathrm{Schur}(\alpha)$ a feedforward structure hidden in the effective, recurrent connectivity $\mathcal{J}(\alpha)$. The difference mode feeds the sum mode with an effective feedforward weight $\mathbf{w}_\mathrm{FF}$ (also a complex number if the eigenvalues have an imaginary component), given by the upper right element of the triangular matrix $\mathbf{T}_\mathrm{Schur}$ – graphically, this corresponds to the "shear" flow field in Figure S3. On top of this, both patterns inhibit themselves with the corresponding negative weight $\boldsymbol{\lambda}_\mathrm{d}$ or $\boldsymbol{\lambda}_\mathrm{s}$ – the "restoring" flow field in Figure S3. Note that the sum of squared moduli (squared Frobenius norm $\|\cdot\|_\mathrm{F}^2$) is preserved by the unitary transformation $\mathcal{J} \mapsto \mathbf{U}^\star\,\mathcal{J}\,\mathbf{U} \equiv \mathbf{T}_\mathrm{Schur}$, such that $\|\mathcal{J}\|_\mathrm{F}^2 = \|\mathbf{T}_\mathrm{Schur}\|_\mathrm{F}^2$, i.e.

$$|\mathbf{w}_\mathrm{FF}| = \sqrt{\|\mathcal{J}\|_\mathrm{F}^2 - (|\boldsymbol{\lambda}_\mathrm{s}|^2 + |\boldsymbol{\lambda}_\mathrm{d}|^2)} \tag{S52}$$

The calculation of the network covariance matrix (Equation S30) can also be performed in the Schur basis, and doing this sheds further light on the roles of $\boldsymbol{\lambda}_\mathrm{d}$, $\boldsymbol{\lambda}_\mathrm{s}$ and $\mathbf{w}_\mathrm{FF}$ in shaping variability. We begin by observing that

$$\begin{aligned}\mathrm{Tr}\,(\boldsymbol{\Sigma}) &= \mathrm{Tr}\left(\langle\delta\mathbf{V}\,\delta\mathbf{V}^\mathsf{T}\rangle\right)\\ &= \mathrm{Tr}\left(\langle\mathbf{U}\,\mathbf{z}\,\mathbf{z}^\star\,\mathbf{U}^\star\rangle\right)\\ &= \mathrm{Tr}\left(\mathbf{U}\langle\,\mathbf{z}\,\mathbf{z}^\star\,\rangle\mathbf{U}^\star\right)\\ &= \mathrm{Tr}\left(\langle\mathbf{z}\,\mathbf{z}^\star\rangle\right)\end{aligned} \tag{S53}$$

(the last step following from $\mathbf{U}\,\mathbf{U}^\star = \mathbf{I}$). Thus, the total variance is preserved in the Schur basis. Next, taking the Fourier transform of Equation S50 and rearranging term yields

$$\hat{\mathbf{z}}(\omega) = (i\,\omega\,\mathbf{I} - \mathbf{T}_\mathrm{Schur})^{-1}\,\mathbf{U}^\star\,\mathbf{T}^{-1}\,\hat{\boldsymbol{\eta}}(\omega) \tag{S54}$$

[6] The eigenvalues remain real over the entire input range for about half of the 1000 random networks studied throughout (all with $\bar{\tau}_\mathrm{I} = 1/2$). In the second half, they go from real to complex-conjugate and then sometimes to real again.

[7] This holds when the eigenvalues of $\mathbf{A}$ are real. When they are complex conjugate, one can still perform a real Schur decomposition by orthogonalizing the imaginary part of the eigenvector against the real part, which yields

$$\mathbf{T}_\mathrm{Schur} = \begin{pmatrix}\mathrm{Re}(\boldsymbol{\lambda}) & a_+\\ a_- & \mathrm{Re}(\boldsymbol{\lambda})\end{pmatrix} \qquad a_\pm \equiv \frac{\mathbf{w}_\mathrm{FF} \pm \sqrt{\mathbf{w}_\mathrm{FF}^2 + 4\,\mathrm{Im}(\boldsymbol{\lambda})^2}}{2} \tag{S51}$$

and the two Schur vectors in this case are also sum-like and difference-like, in this order. At this point (anticipating to some extent what follows this footnote), we note that in the imaginary case, there is a small feedback term proportional to a_- from the sum-mode to the difference-mode. Thus, the picture of the flow fields drawn in Figure S3 is incomplete. However, we will see that in the slow-noise limit (which gives a very good approximation to the output covariance as seen in 3.3), the purely feedforward picture remains exact provided one replaces $\mathbf{w}_\mathrm{FF}$, $\boldsymbol{\lambda}_\mathrm{d}$ and $\boldsymbol{\lambda}_\mathrm{s}$ by their moduli.

where $\hat{\cdot}$ denotes the Fourier transform and $\omega \equiv 2\pi f \tau_E$ is a dimensionless frequency. Moreover, according to Parseval's theorem we have

$$\mathrm{Tr}\left(\langle \mathbf{z}\,\mathbf{z}^\star \rangle\right) = \frac{1}{2\pi\,\tau_E} \int_{-\infty}^{+\infty} \mathrm{Tr}\left(\hat{\mathbf{z}}\,\hat{\mathbf{z}}^\star\right) \mathrm{d}\omega \tag{S55}$$

Thus, combining Equations S53 to S55 we get

$$\mathrm{Tr}(\boldsymbol{\Sigma}) = \frac{2\,\bar{\tau}_\eta}{\pi} \int_{-\infty}^{+\infty} \frac{\mathrm{Tr}\left[(i\,\omega\,\mathbf{I} - \mathbf{T}_{\mathrm{Schur}})^{-1}\,\mathbf{U}^\star\,\tilde{\boldsymbol{\Sigma}}_\eta\,\mathbf{U}\,(i\,\omega\,\mathbf{I} - \mathbf{T}_{\mathrm{Schur}})^{-\star}\right]}{1 + (\bar{\tau}_\eta\,\omega)^2} \mathrm{d}\omega \tag{S56}$$

where $\tilde{\boldsymbol{\Sigma}}_\eta \equiv \mathbf{T}^{-1}\,\boldsymbol{\Sigma}_\eta\,\mathbf{T}^{-1}$. To simplify the calculation we now assume uncorrelated input noise terms, with the power of noise input to E and I balanced such that $\kappa = \bar{\tau}_I$ and $\tilde{\boldsymbol{\Sigma}}_\eta = c^2\,(1 + 1/\bar{\tau}_\eta)\,\mathbf{I}$, leading to:

$$\begin{aligned}\mathrm{Tr}(\boldsymbol{\Sigma}) &= \frac{(1+\bar{\tau}_\eta)\,c^2}{\pi} \int_{-\infty}^{+\infty} \frac{\mathrm{Tr}\left((i\,\omega\,\mathbf{I} - \mathbf{T}_{\mathrm{Schur}})^{-1}(i\,\omega\,\mathbf{I} - \mathbf{T}_{\mathrm{Schur}})^{-\star}\right)}{1 + (\bar{\tau}_\eta\,\omega)^2} \mathrm{d}\omega \\ &= \frac{(1+\bar{\tau}_\eta)\,c^2}{\pi} \int_{-\infty}^{+\infty} \frac{1}{1 + (\bar{\tau}_\eta\,\omega)^2} \left(\frac{1}{|i\,\omega - \lambda_d|^2} + \frac{1}{|i\,\omega - \lambda_s|^2} + \frac{|w_{FF}|^2}{|i\,\omega - \lambda_d|^2 |i\,\omega - \lambda_s|^2} \right) \mathrm{d}\omega\end{aligned} \tag{S57}$$

where the second equality comes from having inverted the upper-triangular matrix $i\,\omega\,\mathbf{I} - \mathbf{T}_{\mathrm{Schur}}$ analytically and taken its squared Frobenius norm. Carrying out the integral gives

$$\mathrm{Tr}(\boldsymbol{\Sigma}) = (1+\bar{\tau}_\eta)\,c^2 \left(\frac{1 - \bar{\tau}_\eta\,\lambda_s^r}{-\lambda_s^r\,(1 - 2\,\bar{\tau}_\eta\,\lambda_s^r + \bar{\tau}_\eta^2\,|\lambda_s|^2)} + \frac{1 - \bar{\tau}_\eta\,\lambda_d^r}{-\lambda_d^r\,(1 - 2\,\bar{\tau}_\eta\,\lambda_d^r + \bar{\tau}_\eta^2\,|\lambda_d|^2)} \right. \tag{S58}$$

$$\left. + \frac{|w_{FF}|^2\left[1 - \bar{\tau}_\eta\,(\lambda_s + \lambda_d)\right]}{-(\lambda_s + \lambda_d)\,|\lambda_s|\,|\lambda_d|\left[1 - \bar{\tau}_\eta\,(\lambda_s + \lambda_d) + \bar{\tau}_\eta^2\,|\lambda_s|\,|\lambda_d|\right]} \right) \tag{S59}$$

where λ_s^r and λ_d^r stand for the real parts of λ_s and λ_d respectively (they must both be negative for the dynamics to be stable).

This expression simplifies in the slow noise limit, $\bar{\tau}_\eta \to \infty$:[8]

$$\mathrm{Tr}(\boldsymbol{\Sigma}) \overset{\bar{\tau}_\eta \to \infty}{\longrightarrow} c^2 \left(\frac{1}{|\lambda_s|^2} + \frac{1}{|\lambda_d|^2} + \frac{|w_{FF}|^2}{|\lambda_s|^2\,|\lambda_d|^2} \right) \tag{S60}$$

In this limit, the simplified picture of the flow fields drawn in a plane of sum and difference activity (Figure S3) which assumed that they were real quantities, becomes accurate even when the eigenvalues of $\mathcal{J}$ are complex-conjugate (in which case, as mentioned above in Footnote 7, the sum-like mode feeds back onto the difference mode, although this interaction is much weaker than the opposite one). Indeed, in Equation S60, the elements of $\mathbf{T}_{\mathrm{Schur}}$ are reduced to their moduli, so even when they are complex one can still interpret Equation S60 as the total variance in a system with the same real Schur vectors, real eigenvalues equal to $-|\lambda_d|$ and $-|\lambda_s|$ respectively, and a real feedforward weight equal to $|w_{FF}|$.

Equation S60 shows in more detail how the shear and restoring flows contribute to variability. In loose terms, the total variance is a sum of two contributions: one that does not depend on w_{FF} and decreases with $1/|\lambda|^2$, and one that grows with $|w_{FF}|^2$ but is also divided by a term of order λ^4 (where λ is a loose notation to denote the overall magnitude of the eigenvalues). Thus, as the input grows, the effect of the eigenvalues on variability becomes much stronger than that of balanced amplification. Such a

[8] More generally, for arbitrary $\bar{\tau}_I$, κ and ρ_{EI}, in the limit $\bar{\tau}_\eta \to \infty$, Equation S60 still holds, in precisely the same form, but in terms of the eigenvalues and feedforward Schur weight of $\mathbf{B}(\alpha) \equiv c\,\boldsymbol{\Sigma}_\eta^{-\frac{1}{2}}\,\mathbf{A}(\alpha)$ rather than of $\mathcal{J}(\alpha)$. This is because, in that limit, $\mathrm{Tr}(\boldsymbol{\Sigma}) = c^2\,\|\mathbf{B}^{-1}\|_F^2$. Note that $\bar{\tau}_I$ cannot affect the result in the limit $\tau_\eta \to \infty$; and that when $\kappa = \bar{\tau}_I$ and $\rho_{EI} = 0$, then $\mathcal{J}(\alpha) = \mathbf{B}(\alpha)$ and hence Equation S60 holds. To see why $\mathrm{Tr}(\boldsymbol{\Sigma}) = c^2\,\|\mathbf{B}^{-1}\|_F^2$ in this limit: most simply, in the slow noise limit, one can think of the noise $\boldsymbol{\eta}(t)$ in Equation S18 as a constant input and solve for its steady state $\delta\mathbf{V} = -\mathbf{A}^{-1}\,\boldsymbol{\eta}$, then form $\boldsymbol{\Sigma} = \langle \delta\mathbf{V}\,\delta\mathbf{V}^{\mathsf{T}} \rangle$.

dominance can also be understood from the structure of the flow fields that negative self-couplings and balanced amplification induce. Restoring flows are proportional to the distance from the origin: the stronger the momentary V_m deviation from the mean in any direction, the stronger the pull towards the origin in the same direction (Figure S3C, green arrows). In contrast, the shear flow grow along the difference axis while pointing in the orthogonal, sum direction, such that larger deviations in the sum do not imply larger shear flow (Figure S3C, orange arrows). Thus, self-inhibition leads to exponential temporal decay of activity fluctuations, whereas balanced amplification gives only linear growth. This explains why, for large enough input, V_m variability decreases with increasing input even when all flow fields grow in magnitude at the same rate (Figure S2A).

Equation S60 also shows that if one of the eigenvalues transiently weakens with increasing input, then variability should transiently grow. This explains a large part of the variability peak observed in the network of the main text, and indeed, it also predicts variability growth in most of the thousand networks investigated here. However, there are cases where variability transiently grows, without any weakening of eigenvalues (Figure S4A). In those cases, setting $\mathbf{w}_{FF}$ to 0 in Equation S60 wrongly predicts purely decaying variability (compare dashed and solid black lines in Figure S4A, bottom). Thus, in general, initial variability growth results from the combined effects of weaker inhibitory self-couplings *and* strong balanced amplification.

3.6 How do shear and restoring flow fields depend on the input?

The input dependence of the shear ($|\mathbf{w}_{FF}|$) and restoring ($|\boldsymbol{\lambda}_s|$, $|\boldsymbol{\lambda}_s|$) flows can be understood from the input dependence of mean responses ($\bar{y}_E$ and $\bar{y}_I$), which was examined previously in Methods S2. First, at $\alpha = 0$ (no input) the effective connectivity is zero, thus $\mathcal{J} = \mathrm{diag}(-1, -\tilde{\tau}_I^{-1})$ and therefore the two eigenvalues are -1 and $-1/\tilde{\tau}_I$. To see how the eigenvalues change with the input, let us note that for a 2×2 matrix, the sum of the eigenvalues is equal to the trace of the matrix while their product is equal to its determinant. Thus, when both eigenvalues are real (which they are for small enough α), both the arithmetic and geometric mean of $|\boldsymbol{\lambda}_s|$ and $|\boldsymbol{\lambda}_d|$ can be related to the elements of $\mathcal{J}$, which themselves depend directly on $\bar{y}_E$ and $\bar{y}_I$. This yields:

$$|\boldsymbol{\lambda}_s| + |\boldsymbol{\lambda}_d| = \tilde{\tau}_I^{-1}\left[1 + \tilde{\tau}_I + \left(J_{II}\,\bar{y}_I - \tilde{\tau}_I\,J_{EE}\,\bar{y}_E\right)\right] \tag{S61}$$

and (S62)

$$|\boldsymbol{\lambda}_s|\,|\boldsymbol{\lambda}_d| = \tilde{\tau}_I^{-1}\left[1 + \mathrm{Det}\,\mathbf{J}\;\bar{y}_E\,\bar{y}_I + \left(J_{II}\,\bar{y}_I - J_{EE}\,\bar{y}_E\right)\right] \tag{S63}$$

We see that, by both measures, the overall restoring flow tends to grow with increasing input α, because i) mean responses grow too, and therefore so does the product term in Equation S63, and ii) $\bar{y}_I$ tends to grow larger than $\bar{y}_E$ (Figure S1E), so that the weighted difference terms inside round brackets in both Equations S61 and S63 increase, at least for large enough α. However, when $g_E\,J_{EE} > g_I\,J_{II}$, the difference term in Equation S63 will initially grow negative with increasing – but small – α, before it increases again for larger α. This means that at least one of the eigenvalues will decrease. In such a case, whether or not *both* eigenvalues decrease transiently depends on the behavior of the difference term in Equation S61. The requirement for this difference term to decrease initially is $\tilde{\tau}_I\,g_E\,J_{EE} > g_I\,J_{II}$ which is harder to satisfy especially when inhibition is fast ($\tilde{\tau}_I$ is small). Thus, we typically expect that one eigenvalue should decrease (or, at least, its growth should be delayed) before growing again (Figure S2A).

As for the shear flow, a similarly simple expression can be obtained in the case of real eigenvalues by noting that the sum of squared eigenvalues in 2×2 matrix $\mathcal{J}$ is equal to $(\mathrm{Tr}\,\mathcal{J})^2 - 2\,\mathrm{Det}\,\mathcal{J}$. This observation yields

$$\begin{aligned}|\mathbf{w}_{FF}| &= \sqrt{\|\mathcal{J}\|_F^2 - (\mathrm{Tr}\,\mathcal{J})^2 + 2\,\mathrm{Det}\,\mathcal{J}} \\ &= \tilde{\tau}_I^{-1}\left(J_{IE}\,\bar{y}_E + \tilde{\tau}_I\,J_{EI}\,\bar{y}_I\right)\end{aligned} \tag{S64}$$

i.e. the shear flow is proportional to a weighted average of mean V_m responses in the E and I units, which, in the SSN, shows linear growth for small α and sublinear growth for larger α (cf. Methods S2 and Figure S1D). Thus, we have a situation in which the flow that boosts variability grows faster initially than those that quench variability, causing a transient increase in total variance for small increasing inputs. For large α, all flows ($|\boldsymbol{\lambda}_s|$, $|\boldsymbol{\lambda}_d|$ and w_{FF}) grow as $\sqrt{\alpha}$ (Figure S2A), because $\mathcal{J}$ is dominated by its $J_{\alpha\beta}\,\overline{y}_\beta$ components and the $\overline{y}$ terms grow as $\sqrt{\alpha}$ as seen in Methods S2. Thus, the total variance in Equation S60 should decay as $1/\alpha$ in this limit, consistent with what we concluded in 3.3.

When the eigenvalues of $\mathcal{J}$ turn complex-conjugate, Equations S61, S63 and S64 above become more complicated expressions, which nevertheless does not change the main insights.

Methods S4 Firing rate and spike count variability

4.1 Generic results in the SSN regime

In Equation S34, we derived a generic scaling of membrane potential variances, $\boldsymbol{\Sigma}$, with mean responses in the SSN. What does it imply for rate variances and Fano factors? Firing rate variability, $\boldsymbol{\Sigma}^{\mathrm{r}}$, is straightforwardly related to voltage variability through a linearization of the input/output nonlinearity, yielding the following relationship:

$$\Sigma^{\mathrm{r}}_{ij} \propto \overline{V}_i^{(n-1)}\,\overline{V}_j^{(n-1)}\,\Sigma_{ij} \approx \mathcal{O}(1) \tag{S65}$$

Therefore, whether $\boldsymbol{\Sigma}^{\mathrm{r}}$ grows or shrinks with increasing activation will depend on parameter details. (Note that this is valid only to the extent that mean responses keep growing with large stimuli, which occurs when $\Omega_E > 0$ – see 3.3 above. For $\Omega_E < 0$ we observe a decline of firing rate variance with increasing stimulus.)

Under the assumption that spikes are emitted according to an inhomogeneous Poisson process with underlying rate given by a threshold-powerlaw nonlinearity, we have shown in Hennequin and Lengyel (2016) that the above-Poisson contribution to Fano factors (FF-1), due to slow voltage variability, scales as

$$\mathrm{FF}_i - 1 \propto \overline{V}_i^{n-2}\,\Sigma_{ii} \tag{S66}$$

Substituting Equation S34 into this, we have that

$$\mathrm{FF}_i - 1 \propto \overline{V}_i^{-n} \tag{S67}$$

Thus, Fano factors are generally expected to decrease (towards a Poisson lower-bound of 1) as long as the stimulus increases mean responses.

4.2 The specific regime of Kanashiro et al. (2017)

Kanashiro et al. (2017) studied a two-population E-I model (analogous to what we analyzed in Fig. 2 of the main text) in which they analyzed conditions for attention to suppress variability and increase stimulus gain. In apparently conflict with our main result that variability suppression should occur generically, they reported very specific conditions for variability quenching. In this section, we relate their model to ours directly to understand the sources of this apparent contradiction.

Kanashiro et al. (2017) studied mean-field dynamics for firing rates $\mathbf{r} = \begin{pmatrix} r_E \\ r_I \end{pmatrix}$ (an 'r-equation') of the form

$$\tau_E \mathbf{T}\,\frac{\mathrm{d}}{\mathrm{d}t}\mathbf{r} = -\mathbf{r} + \mathbf{f}\left(\mathbf{W}\,\mathbf{r} + c\,\mathbf{g} + a\,\boldsymbol{\mu} + \boldsymbol{\eta}(t)\right) \tag{S68}$$

where boldface small or Greek letters denote two-vectors with an E and I component, the function $\mathbf{f}(\cdot)$ represents applying the function $f_{\mathrm{E}}(\cdot)$ to the E component and $f_{\mathrm{I}}(\cdot)$ to the I component, $\boldsymbol{\eta}(t)$ is a zero-mean unit-variance noise, $c\,\mathbf{g}$ represents a stimulus-driven input, of strength c, while $a\,\boldsymbol{\mu}$ represents an attentional input, of strength $0 \leq a \leq 1$.

For the input-output function $\mathbf{f}(\cdot)$, Kanashiro et al. (2017) used the firing rate of an integrate-and-fire neuron responding to a given mean and variance of input. This is an expansive function that, for a fixed level of fast noise, can be well approximated as a power law (Hansel and van Vreeswijk, 2002) (precisely the form of nonlinearity we used in our model (S1). Nevertheless, most of the results we derive in this section (unless otherwise noted) hold for an arbitrary monotonically increasing, expansive $\mathbf{f}(\cdot)$, and thus wherever possible, we will express them in terms of $\mathbf{f}(\cdot)$ and its slope, $\mathbf{f}'(\cdot)$, rather than using our previous approach to express the scaling of variability in terms of $\overline{V}$ and its powers, which was specific to a powerlaw nonlinearity[9].

Although in contrast to Kanashiro et al.'s r-equation, we studied an equation for voltage dynamics (a 'v-equation'; Equation S1), there is a simple equivalence between these two forms of model[10]. In particular, linearizing Equation S68 gives the covariance matrix of rate fluctuations as $\boldsymbol{\Sigma}^{\mathrm{r}} = \mathbf{F}\,\Sigma\,\mathbf{F}$ where $\boldsymbol{\Sigma}$ is the covariance matrix of voltage fluctuations implied by our (linearized) voltage equation Equation S18 with the same input noise $\boldsymbol{\eta}(t)$, and $\mathbf{F} = \begin{pmatrix} f'_{\mathrm{E}} & 0 \\ 0 & f'_{\mathrm{I}} \end{pmatrix}$ (cf. Equation S65).

Like us, Kanashiro et al. (2017) analyzed variability by linearizing the dynamics about a fixed point, and they studied the slow-noise limit; thus we shall also restrict our analysis to this limit here. They concluded that, to reduce variability, attentional input had to be biased toward inhibitory cells ($\mu_{\mathrm{I}} > \mu_{\mathrm{E}}$); while for attentional input to increase the gain of response to a stimulus, stimulus-driven input had to be directed to excitatory cells ($g_{\mathrm{E}} > g_{\mathrm{I}}$). As we will show, these conclusions depend on the specific, non-generic parameter choices they made, which eliminate the more generic suppression of variability by increasing activity seen in the SSN.

In particular, Kanashiro et al. (2017) simplified the weight matrix $\begin{pmatrix} W_{\mathrm{EE}} & -W_{\mathrm{EI}} \\ W_{\mathrm{IE}} & -W_{\mathrm{II}} \end{pmatrix}$ to the special, non-generic form $\begin{pmatrix} W_{\mathrm{E}} & -W_{\mathrm{I}} \\ W_{\mathrm{E}} & -W_{\mathrm{I}} \end{pmatrix}$. This assumption on the weights means that Det $\mathbf{W} = 0$ and $\Omega_{\mathrm{E}} = \Omega_{\mathrm{I}} = 0$, which eliminates many SSN behaviors.[11] Furthermore this means that Det $\mathbf{A}$ scales as $(f')^1$ instead of the generic $(f')^2$ (because one of the eigenvalues of $\mathbf{A}$ is -1, independent of the values of the f''s, cf. Footnote 9), so that $\mathbf{A}^{-1}$ scales as $(f')^0$. Therefore, $\boldsymbol{\Sigma}$ scales as $(f')^0$ instead of the generic $(f')^{-2}$, $\boldsymbol{\Sigma}^{\mathrm{r}}$ scales as $(f')^2$ instead of the generic $(f')^0$ (cf. Footnote 9). To see the implications of this scaling for Fano factors, we recall that the firing rate nonlinearity f used by Kanashiro et al. is well approximated by a threshold powerlaw with some exponent n; thus, from the general results developed in 4.1, we expect the above-Poisson part of the Fano factor, $\propto \overline{V}^{n-2}\Sigma$, to scale as $(f')^{(n-2)/(n-1)}$ instead of the generic $(f')^{-\frac{n}{n-1}}$. In short, this choice of parameters causes $\boldsymbol{\Sigma}$ and the Fano factor to lose their generic decrease with increasing activity (and in fact, causes the Fano factor to generically increase instead), and causes $\boldsymbol{\Sigma}^{\mathrm{r}}$ to change from going to a constant for large f''s to generically increasing with increasing activity. This renders any decrease in these measures of variability with increasing activity much more

[9]For example, it is easy to show that the scaling of effective connectivity, membrane potential and rate variability developed in Equations S34 and S65 can be written using this more general approach as Det $\mathbf{A} \propto (f')^2$, $\boldsymbol{\Sigma} \propto (f')^{-2}$ and $\boldsymbol{\Sigma}^{\mathrm{r}} \propto (f')^0$, respectively.

[10]When $\mathbf{T}$ (a diagonal matrix of relative time constants) is proportional to the identity matrix, the r-equation $\tau \frac{\mathrm{d}}{\mathrm{d}t}\mathbf{r} = -\mathbf{r} + \mathbf{f}\left(\mathbf{W}\mathbf{r} + \mathbf{h}_r(t)\right)$ is equivalent to the v-equation $\tau \frac{\mathrm{d}}{\mathrm{d}t}\mathbf{v} = -\mathbf{v} + W\mathbf{f}(\mathbf{v}) + \mathbf{h}_v(t)$, under the equivalence $\mathbf{v} = \mathbf{W}\mathbf{r} + \mathbf{h}_r$, $\tau \frac{\mathrm{d}}{\mathrm{d}t}\mathbf{h}_r = -\mathbf{h}_r + \mathbf{h}_v$ (Miller and Fumarola, 2012). For steady states or in the slow noise limit, the rate and voltage equations are equivalent under the simpler relationship $\mathbf{r} = f(\mathbf{v})$, $\mathbf{h}_r = \mathbf{h}_v$, regardless of the structure of $\mathbf{T}$.

[11]The SSN (Ahmadian et al., 2013) relies on a positive determinant of $\mathbf{W}$ to ensure stability and also to ensure that the "loosely balanced" solution exists, which depends on $\mathbf{W}$ being invertible (this solution is illustrated in Equation S11-Equation S15). The loosely balanced solution characterizes SSN dynamics for stronger input (roughly, for stimulus-driven rather than spontaneous input). Furthermore, SSN dynamical regimes are characterized by the nonzero values of Ω_{E} and Ω_{I} (Equations S13 and S14).

dependent on specific parameter choices, including in particular the choice of relative strength of external input to E vs. I cells ($\mu_{\mathrm{I}} > \mu_e$).

More specifically, Kanashiro et al. (2017) focused on the variance of r_{E} (the EE component of $\boldsymbol{\Sigma}^{\mathrm{r}}$). In the general case in the slow noise limit, this is (from Equation S26)

$$\Sigma^{\mathrm{r}}_{\mathrm{EE}} = \frac{(1+f'_{\mathrm{I}}\,W_{\mathrm{II}})((1+f'_{\mathrm{I}}\,W_{\mathrm{II}})\,(f'_{\mathrm{E}}\,c_{\mathrm{E}})^2 - 2f'^2_{\mathrm{E}}f'_{\mathrm{I}}\,W_{\mathrm{EI}}\,c_{\mathrm{EI}}) + (f'_{\mathrm{E}}\,W_{\mathrm{EI}})^2\,(f'_{\mathrm{I}}\,c_{\mathrm{I}})^2}{(\mathrm{Det}\,\mathbf{A})^2} \tag{S69}$$

where $\mathrm{Det}\,\mathbf{A} = 1 - f'_{\mathrm{E}}\,W_{\mathrm{EE}} + f'_{\mathrm{I}}\,W_{\mathrm{II}} + f'_{\mathrm{I}}\,f'_{\mathrm{E}}\,\mathrm{Det}\,\mathbf{W}$. In addition to the assumption on the structure of $\mathbf{W}$, Kanashiro et al. (2017) also assumed that the inhibitory and excitatory input noise were perfectly correlated, $c_{\mathrm{EI}} = c_{\mathrm{E}}\,c_{\mathrm{I}}$ (because they assumed a single, global noise process in Equation S68). Using these assumptions, the excitatory rate variance instead becomes

$$\Sigma^{\mathrm{r}}_{\mathrm{EE}}\big|_{\mathrm{Kanashiro}} = \frac{f'^2_{\mathrm{E}}\,(f'_{\mathrm{E}}\,c_{\mathrm{E}} + f'_{\mathrm{I}}\,W_{\mathrm{I}}\,f'_{\mathrm{E}}\,c_{\mathrm{E}} - f'^2_{\mathrm{I}}\,W_{\mathrm{I}}\,c_{\mathrm{I}})^2}{\left(\mathrm{Det}\,\mathbf{A}|_{\mathrm{Kanashiro}}\right)^2} \tag{S70}$$

where $\mathrm{Det}\,\mathbf{A}|_{\mathrm{Kanashiro}} = 1 - f'_{\mathrm{E}}\,W_{\mathrm{E}} + f'_{\mathrm{I}}\,W_{\mathrm{I}}$. The numerator of $\Sigma^{\mathrm{r}}_{\mathrm{EE}}|_{\mathrm{Kanashiro}}$ increases with increasing f'_{E} and decreases with increasing f'_{I}, while the opposite is true of the denominator; this is why their increase in f'_{I} had to dominate the increase in f'_{E} for them to find a decrease in variability. For the generic $\Sigma^{\mathrm{r}}_{\mathrm{EE}}$ (Equation S69), there is no such simple monotonic dependence on f'_{E} or f'_{I}; while results may be parameter dependent, there is no obvious reason for this generic $\Sigma^{\mathrm{r}}_{\mathrm{EE}}$ why external input must be biased towards I cells in order for increasing activity to suppress excitatory rate variability.

Kanashiro et al. (2017) did, in one figure, consider the effects of a more general weight matrix. They considered the same attention-induced trajectory in r_{E} and r_{I} that decreased variability for their restricted weight matrix, and showed that this also decreased variability for a parametric range of weights. However, they did not examine whether their conclusion that variability reduction required $\mu_{\mathrm{I}} > \mu_{\mathrm{E}}$ held in this more general case.

Finally, Kanashiro et al. (2017) considered the attention-induced change in gain of excitatory cells to a stimulus-driven input. The excitatory stimulus gain is $\frac{dr^{\mathrm{SS}}_{\mathrm{E}}}{dc}$, where $r^{\mathrm{SS}}_{\mathrm{E}}$ is the E-component of the deterministic steady-state value $\mathbf{r}^{\mathrm{SS}} = \mathbf{f}\left(\mathbf{W}\,\mathbf{r}^{\mathrm{SS}} + c\,\mathbf{g} + a\,\boldsymbol{\mu}\right)$. We can compute $\frac{\mathrm{d}\mathbf{r}^{\mathrm{SS}}}{\mathrm{d}c} = \mathbf{F}\,(\mathbf{W}\,\frac{\mathrm{d}\mathbf{r}^{\mathrm{SS}}}{\mathrm{d}c} + \mathbf{g})$, which can be solved to give $\frac{\mathrm{d}\mathbf{r}^{\mathrm{SS}}}{\mathrm{d}c} = (\mathbf{I} - \mathbf{F}\,\mathbf{W})^{-1}\,\mathbf{F}\,\mathbf{g}$. The E-component is then

$$\frac{\mathrm{d}r^{\mathrm{SS}}_{\mathrm{E}}}{\mathrm{d}c} = \frac{f'_{\mathrm{E}}\,((1+f'_{\mathrm{I}}\,W_{\mathrm{II}})\,g_{\mathrm{E}} - f'_{\mathrm{I}}\,W_{\mathrm{EI}}\,g_{\mathrm{I}})}{\mathrm{Det}\,\mathbf{A}} \tag{S71}$$

We now note that, if we write Σ^{*}_{EE} for $\Sigma^{\mathrm{r}}_{\mathrm{EE}}$ under the special condition of perfect correlation and equal variances ($c_{\mathrm{EI}} = c_{\mathrm{E}}\,c_{\mathrm{I}}$, and $c_{\mathrm{E}} = c_{\mathrm{I}}$) then the numerator of Σ^{*}_{EE} is $f'^2_{\mathrm{E}}\,c^2_{\mathrm{E}}\,(1 + f'_{\mathrm{I}}\,(W_{\mathrm{II}} - W_{\mathrm{EI}}))^2$ (the denominator remains $(\mathrm{Det}\,\mathbf{A})^2$). This allows us to write

$$\frac{\mathrm{d}r^{\mathrm{SS}}_{\mathrm{E}}}{\mathrm{d}c} = \frac{g_{\mathrm{E}}}{c_{\mathrm{E}}}\,\sqrt{\Sigma^{*}_{\mathrm{EE}}} + f'_{\mathrm{E}}f'_{\mathrm{I}}\,W_{\mathrm{EI}}\,(g_{\mathrm{E}} - g_{\mathrm{I}})/\mathrm{Det}\,\mathbf{A} \tag{S72}$$

Note that $\mathrm{Det}\,\mathbf{A} > 0$ is a necessary condition for the fixed point to be stable and $f'_{\mathrm{E}} > 0, f'_{\mathrm{I}} > 0$. This means that, if an attentional manipulation lowers Σ^{*}_{EE}, then in order for it to raise the excitatory stimulus gain it must either be the case that $g_{\mathrm{E}} > g_{\mathrm{I}}$ and the attentional stimulus increases the second term, by increasing $f'_{\mathrm{E}}f'_{\mathrm{I}}/\mathrm{Det}\,\mathbf{A}$, more than the first term decreases; or $g_{\mathrm{E}} < g_{\mathrm{I}}$ and the attentional stimulus decreases the magnitude of the 2nd term, by decreasing $f'_{\mathrm{E}}f'_{\mathrm{I}}/\mathrm{Det}\,\mathbf{A}$, by more than the decrease in the first term. However, an attentional stimulus may raise Σ^{*}_{EE} while lowering $\Sigma^{\mathrm{r}}_{\mathrm{EE}}$.

With the assumptions of Kanashiro et al. (2017), Equation S71 becomes $\frac{\mathrm{d}r^{\mathrm{SS}}_{\mathrm{E}}}{\mathrm{d}c} = \frac{f'_{\mathrm{E}}\,(g_{\mathrm{E}} + W_{\mathrm{I}}\,f'_{\mathrm{I}}\,(g_{\mathrm{E}} - g_{\mathrm{I}}))}{\mathrm{Det}\,\mathbf{A}|_{\mathrm{Kanashiro}}}$ and the numerator of Σ^{*}_{EE} becomes $f'^2_{\mathrm{E}}\,c^2_{\mathrm{E}}$. Kanashiro et al. (2017) restricted their analysis to the case $c_{\mathrm{I}} = c_{\mathrm{E}}$, so that with their other assumptions $\Sigma^{\mathrm{r}}_{\mathrm{EE}} = \Sigma^{*}_{\mathrm{EE}}$, and so simply wrote

$$\frac{\mathrm{d}r^{\mathrm{SS}}_{\mathrm{E}}}{\mathrm{d}c} = \frac{g_{\mathrm{E}}}{c_{\mathrm{E}}}\,\sqrt{\Sigma^{\mathrm{r}}_{\mathrm{EE}}} + f'_{\mathrm{E}}f'_{\mathrm{I}}\,W_{\mathrm{I}}\,(g_{\mathrm{E}} - g_{\mathrm{I}})/\mathrm{Det}\,\mathbf{A}|_{\mathrm{Kanashiro}} \tag{S73}$$

Note that the numerator of the second term is quadratic in the increasing f' terms, while their denominator (unlike the denominator in Equation S72) is only linear in these terms. Thus, under Kanashiro et al., 2017's assumptions, the second term should generically increase in magnitude with the increased activation induced by attention. Perhaps on this basis, they predicted that attention's observed effects of lowering excitatory variability and raising excitatory stimulus gain required $g_{\mathrm{E}} > g_{\mathrm{I}}$.

Defining $\Sigma^{\Delta}_{\mathrm{EE}} \equiv \Sigma^{\mathrm{r}}_{\mathrm{EE}} - \Sigma^{*}_{\mathrm{EE}}$,[12] Equation S72 can be rewritten as

$$\frac{\mathrm{d}r^{\mathrm{SS}}_{\mathrm{E}}}{\mathrm{d}c} = \frac{g_{\mathrm{E}}}{c_{\mathrm{E}}} \sqrt{\Sigma^{\mathrm{r}}_{\mathrm{EE}} - \Sigma^{\Delta}_{\mathrm{EE}}} + f'_{\mathrm{E}} f'_{\mathrm{I}}\, W_{\mathrm{EI}}\, (g_{\mathrm{E}} - g_{\mathrm{I}}) / \mathrm{Det}\,\mathbf{A} \tag{S76}$$

Note that, with $\Sigma^{\mathrm{r}}_{\mathrm{EE}}$ decreasing, the first term of $\frac{\mathrm{d}r^{\mathrm{SS}}_{\mathrm{E}}}{\mathrm{d}c}$ can be increasing if $\Sigma^{\Delta}_{\mathrm{EE}}$ decreases by more than $\Sigma^{\mathrm{r}}_{\mathrm{EE}}$. Thus, for generic parameters, we conclude that attention can increase excitatory stimulus gain while lowering $\Sigma^{\mathrm{r}}_{\mathrm{EE}}$ by virtue of the first term of Equation S76 increasing with attention, which will occur if $\Sigma^{\Delta}_{\mathrm{EE}}$ decreases more than $\Sigma^{\mathrm{r}}_{\mathrm{EE}}$, and/or of the second term increasing with attention, which will occur for $g_{\mathrm{E}} > g_{\mathrm{I}}$ or $g_{\mathrm{E}} < g_{\mathrm{I}}$ if $f'_{\mathrm{E}} f'_{\mathrm{I}} / \mathrm{Det}\,\mathbf{A}$ increases or decreases, respectively, with attention.

[12]With the assumptions of Kanashiro et al. (2017),

$$\Sigma^{\Delta}_{\mathrm{EE}}\big|_{\mathrm{Kanashiro}} = \left(f'^{2}_{\mathrm{E}} f'_{\mathrm{I}}\, W_{\mathrm{I}}\, (c_{\mathrm{E}} - c_{\mathrm{I}})\, \left(2\, c_{\mathrm{E}} + f'_{\mathrm{I}}\, W_{\mathrm{I}}\, (c_{\mathrm{E}} - c_{\mathrm{I}})\right)\right) / \left(\mathrm{Det}\,\mathbf{A}\big|_{\mathrm{Kanashiro}}\right)^{2} \tag{S74}$$

More generally,

$$\Sigma^{\Delta}_{\mathrm{EE}} = \left(f'^{2}_{\mathrm{E}} f'_{\mathrm{I}}\, W_{\mathrm{EI}}\, \left(2\,(1 + f'_{\mathrm{I}}\, W_{\mathrm{II}})\,(c^{2}_{\mathrm{E}} - c_{\mathrm{EI}}) + f'_{\mathrm{I}}\, W_{\mathrm{EI}}\,(c^{2}_{\mathrm{I}} - c^{2}_{\mathrm{E}})\right)\right) / (\mathrm{Det}\,\mathbf{A})^{2} \tag{S75}$$

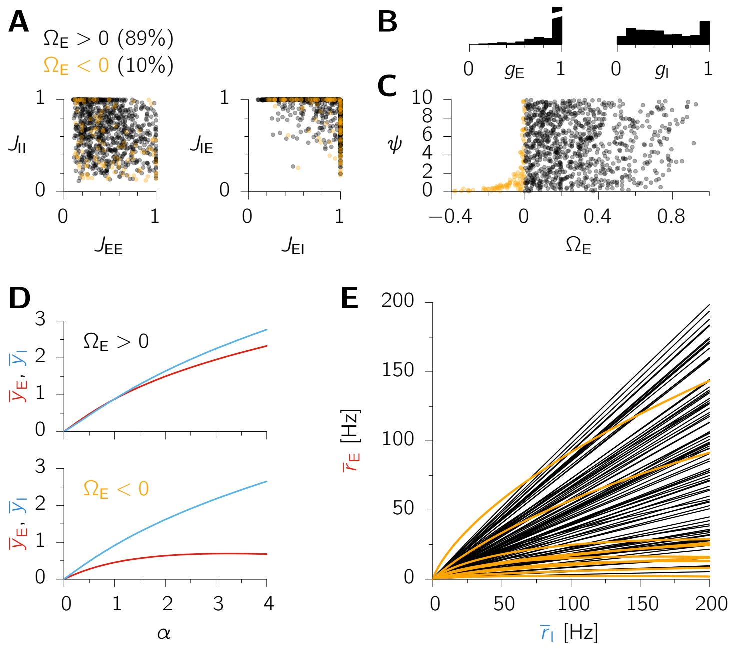

Figure S1. Related to Figure 2. Typical behavior of mean responses to increasing inputs in 1000 randomly sampled 2-population SSNs. **(A)** Dimensionless recurrent weights $\{J_{\alpha\beta}\}$ (Equation S8); these are normalized such that the largest of the four weights is one for each network. Colors indicate the sign of Ω_E (Equation S13). **(B)** Distribution of feedforward weights g_E and g_I, also normalized for each network so that their maximum is one. **(C)** Overall connection strength ψ (in units of W, see table "Parameters Used in the SSN Simulations" in STAR Methods, such that $W_{\alpha\beta} \equiv \psi J_{\alpha\beta}$) vs. Ω_E. **(D)** Example responses (dimensionless voltages $\overline{y}_E$ and $\overline{y}_I$) to increasing inputs (dimensionless α) for a network with $\Omega_E > 0$ (top) and one with $\Omega_E < 0$ showing supersaturation (bottom). **(E)** Mean E firing rate $\overline{r}_E$ as a function of the mean I firing rate $\overline{r}_I$, for a subset of networks; each point on these curves corresponds to a different input level, increased from zero to a maximum value chosen such that $\overline{r}_I$ = 200 Hz.

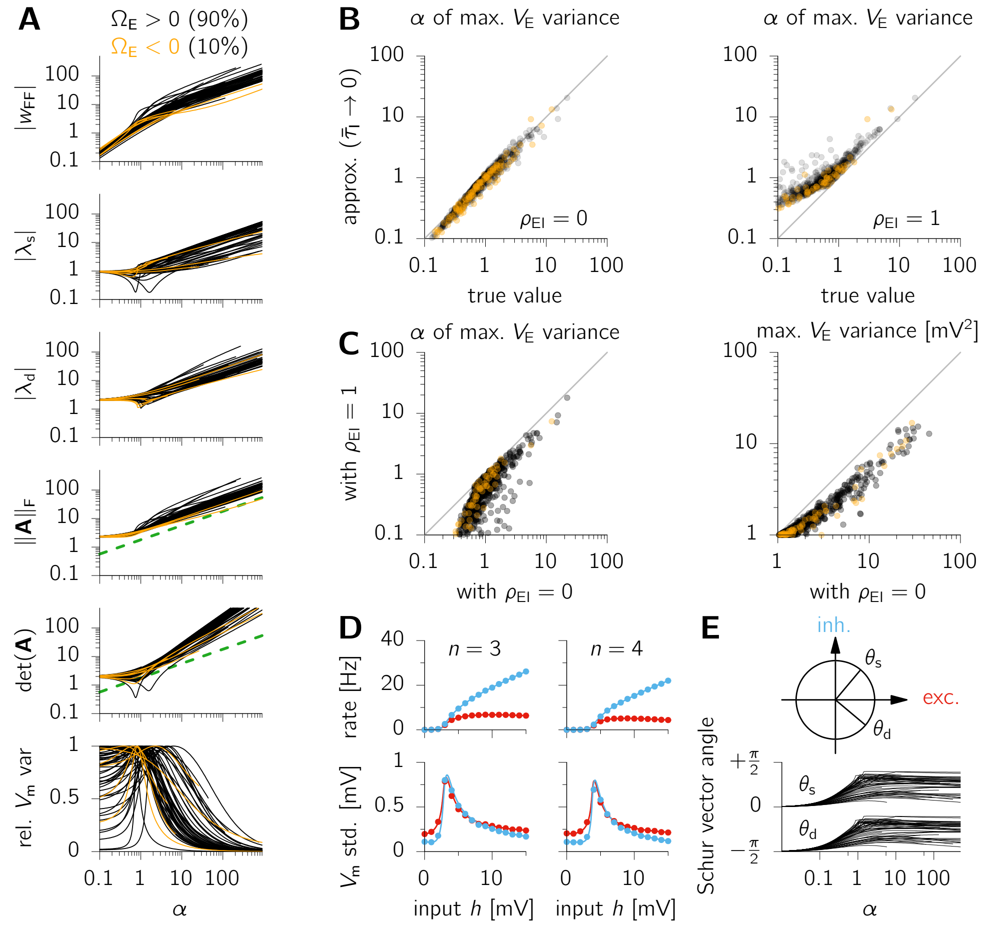

Figure S2. Related to Figure 2. Robustness of variability modulation to changes in network parameters. We examined the modulation of variability by external input in the 1000 randomly parameterized, 2-population networks of Figure S1. **(A)** Behavior of $|w_{FF}|$, $|\lambda_s|$, $|\lambda_d|$, $\|\mathbf{A}\|_F$, $\det(\mathbf{A})$ (Equation S49) and the total variance (normalized to unit peak), as a function of the (dimensionless) input α. The dashed green line is proportional to $\sqrt{\alpha}$. Only a random subset of the thousand random networks are shown. Following the same convention as in Figure S1, cases with $\Omega_E > 0$ are shown in black, those with $\Omega_E < 0$ in orange. **(B)** Scatter plot of the α at which the E variance reaches its maximum ("true value"), and that given by the approximate criterion of Equation S45 (which assumes very fast inhibition, i.e. $\bar{\tau}_I \to 0$), for uncorrelated (left, $\rho_{EI} = 0$) and fully correlated (right, $\rho_{EI} = 1$) input noise term to the E and I units. **(C)** Scatter plot of the input α at which the E variance peaks (left), as well as the value of the variance peak (right), for $\rho_{EI} = 0$ vs. $\rho_{EI} = 1$. **(D)** Mean E (red) and I (blue) firing rates (top) and V_m std. (bottom) for two example networks with larger values of the power-law exponent n; parameters were otherwise the same as in Figure 2 of the main text. **(E)** Orientation of the two Schur vectors for a subset of the 1000 random networks. Their "sum-like" and "difference-like" nature emerges quite rapidly for small α and then persists for larger α.

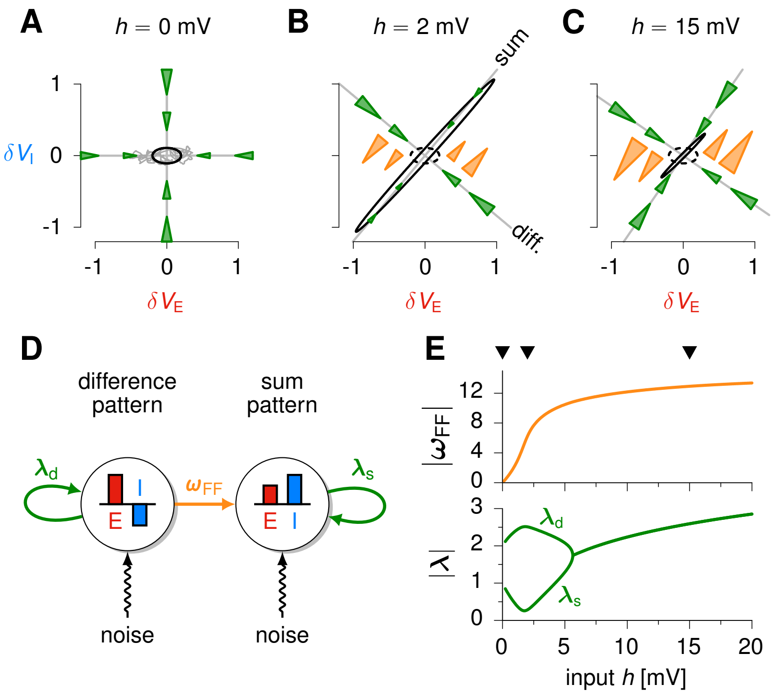

Figure S3. Related to Figure 2. Mechanism of input-dependent modulation of variability in the SSN. (A–C) Visualization of the influence of single-neuron leak and effective connectivity as restoring and shear flow fields shaping the (co-)variability of E/I activity in the two-population SSN. Cardinal axes (δV_E and δV_I) measure (in mV) the deviation of E and I membrane potentials from their respective steady-state values (Equation S18) defined such that the origin ($\delta V = 0$ mV) corresponds to the stationary mean population activity for the given input strength h (labels on top). Gray axes show directions of Schur vectors (Equation S49) along which the restoring flow field acts (green triangular arrows) and which are also coupled by the shear flow field (w_{FF}, orange triangular arrows) such that deviations along the "difference" axis give rise to deviations along the "sum" axis. Triangular arrows are proportional in area to the contribution they make to the total flow of fluctuations. Gray traces show example membrane potential fluctuations of the network, black covariance ellipses show contour lines of the corresponding joint distribution of δV_E and δV_I at one standard deviation, dashed ellipses in (B) and (C) reproduce covariance ellipse at $h = 0$ mV (A) for comparison. At $h = 0$ **(A)**, the only contributor to the flow of trajectories is the leak in each population (green flow field) acting along the cardinal axes of E/I fluctuations – the flow is stronger (suppresses fluctuations more) along the I axis due to the shorter membrane time constant in I cells. This flow contains the diffusion due to input noise (cf. example trajectory in gray), resulting in uncorrelated baseline E/I fluctuations (black ellipse is axis aligned). As the network is driven by $h > 0$ **(B–C)**, the effective recurrent connectivity adds to the leak to instate two types of flow fields steering fluctuations: a restoring flow field (green, generalizing the leak in (A)) and a "shear-like" flow field (orange). The relative contributions of the two flow fields determine the size and elongation of the E/I covariance (solid black ellipses). **(D)** Illustration of the decomposition of the effective connectivity (for a given mean stimulus h; Equation S49) as couplings between a difference-like pattern (left) and a sum-like pattern (right; cf. rotated gray axes in B-C). The difference mode feeds the sum mode with weight w_{FF} (orange arrow), and the difference and sum patterns inhibit themselves with negative weight λ_d and λ_s respectively (green arrows). These three h-dependent couplings scale the corresponding flow fields in (A-C) (consistent colors). **(E)** Input-dependence of w_{FF} (top, orange) and $|\lambda_d|$ and $|\lambda_s|$ (bottom, green). Black triangular marks indicate input levels illustrated in (A-C).

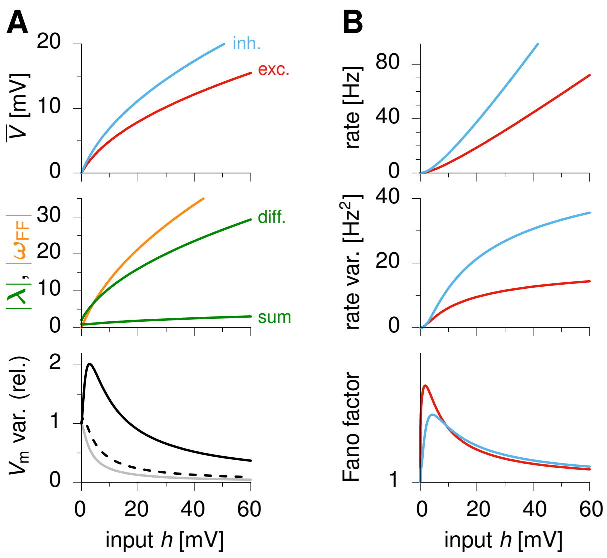

Figure S4. Related to Figure 2. Variability modulation cannot be understood based on the restoring flows (i.e. the eigenvalues of the Jacobian of the dynamics) alone. (A) Example 2-population network showing transient increase in variability with increasing external input h (bottom, black, normalized to variance at $h = 0$ mV), *without* any substantial decrease in any of the eigenvalues, and in $|\boldsymbol{\lambda}_s|$ in particular (middle, green; cf. Figure S3E, bottom). The dashed black curve (bottom) shows the predicted variability (Equation S60) assuming $\mathbf{w}_{FF} = 0$ uniformly (cf. middle, orange), i.e. taking into account only the magnitude of the restoring flows $\boldsymbol{\lambda}_d$ and $\boldsymbol{\lambda}_s$ (middle, green). The gray curve (bottom) is the prediction made by assuming fully correlated input noise terms with variance g_E^2 and g_I^2 respectively for the E and I units. Variability in this case can be read off from the slope of the $\overline{V}_E$ (top, red) and $\overline{V}_I$ curves (top, blue), because input noise becomes equivalent to fluctuations in h to which the network has time to respond. Neither of these two predictions capture the initial growth of variability and, consequently, both grossly underestimate the overall magnitude of variability across the whole range of inputs. **(B)** Mean firing rates (top), variances of firing rate fluctuations (middle) and Fano factors (assuming Poisson spike emission on top of rate fluctuations), in the same network as in (A) for the E (red) and I populations (blue). Note that the overall scale of super-Poisson variability (Fano factor minus one) is arbitrary here, and in general depends on the counting window, autocorrelation time constants, and the variance of the input noise. Parameters: $\tau_\eta \to \infty, g_E = 0.77, g_I = 1, J_{EE} = 0.38, J_{EI} = 0.27, J_{IE} = 1, J_{II} = 0.6, \psi = 2.37$.

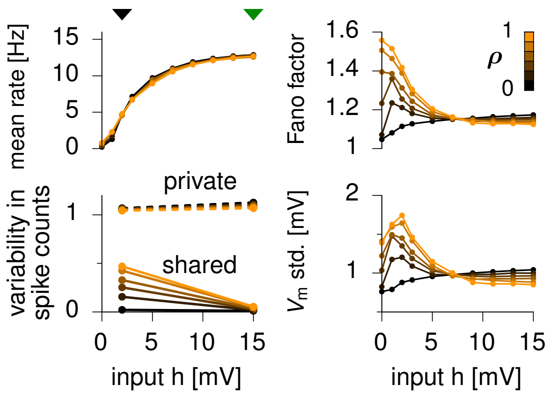

Figure S5. Related to Figure 3. Input correlation-dependent behavior of the spiking SSN. Top left: mean population firing rates (top left) as a function of input strength h, and for different values of the input correlation ρ (color coded). Triangular marks denote the values of h used in spontaneous (black) and evoked (green) conditions in Figure 3. **Top right**: Fano factors (population average $\pm$ std.) **Bottom right**: V_{m} std. (population average $\pm$ std.) **Bottom left**: factor analysis applied to normalized spike counts (such that the total variance equals the average Fano factor; see STAR Methods) to decompose variability into a shared component (one single factor), and a private component (Churchland et al., 2010). Note that only the shared part of variability is quenched by increasing stimulus, and that shared variability and its quenching both require a non-zero input correlation coefficient ρ.

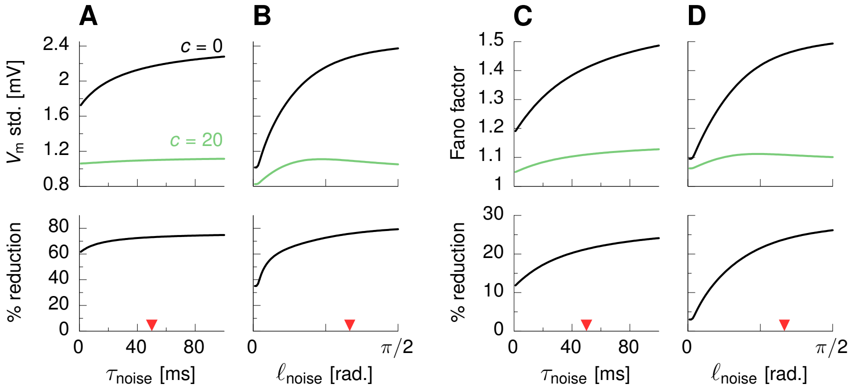

Figure S6. Related to Figure 4. Dependence of variability reduction in the ring SSN model on spatial and temporal correlations in the input noise. Dependence of the network-averaged V_m std. **(A-B)** and Fano factor **(C-D)** on either the temporal correlation time constant τ_{noise} in the external input noise term (for fixed $\ell_{noise} = 60°$) **(A, C)**, or its spatial correlation length ℓ_{noise} (for fixed $\tau_{noise} = 50$ ms) **(B, D)**, in the spontaneous ($c = 0$, black) and high-contrast ($c = 20$, green) input regimes. Red arrows indicate the parameter values used in the main text (see table "Parameters Used in the SSN Simulations" in STAR Methods). Top panels show absolute magnitude of variability, bottom panels show the amount of relative variability suppression for the high contrast input, as a percentage of spontaneous variability.

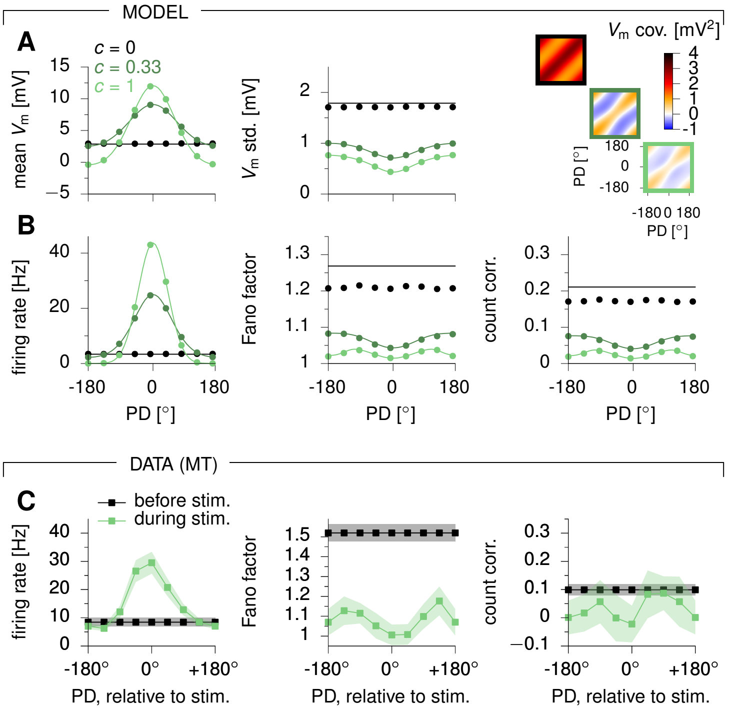

Figure S7. Related to Figure 4. A ring SSN accounts for the stimulus dependence of across-trial variability in area MT. (A) V_m mean (left) and std. (center) as a function of the model neuron's preferred direction (PD, relative to stimulus at 0°), for increasing values of stimulus strength c. The full V_m covariance matrices are shown on the right for the E population, box color indicating c. **(B)** Mean firing rates (left), spike count Fano factors (center), and spike count correlations between similarly tuned neurons (right), as a function of the neurons' (mean) preferred direction. **(C)** Experimental data (awake monkey MT) adapted from (Ponce-Alvarez et al., 2013), with average firing rates (left), average Fano factors (center), and average spike count correlations among similarly tuned cells (right), as a function of the cells' preferred direction. Data is shown for spontaneous (pre-stimulus, black) and evoked (high-contrast stimulus, green) activity periods. Error bars denote s.e.m. Dots in panels A–B were obtained from 400 s epochs of simulated stationary activity, and denote averages among cells with similar tuning preferences (PD difference$< 18°$); solid lines show analytical approximations (Hennequin and Lengyel, 2016). In panels B-C, spikes were counted in 100 ms bins. The only parameters that differed from Figure 4 of the main text were: $\ell_{syn} = \ell_{noise} = \ell_{stim} = 80°$ (see table "Parameters Used in the SSN simulations" in STAR Methods).

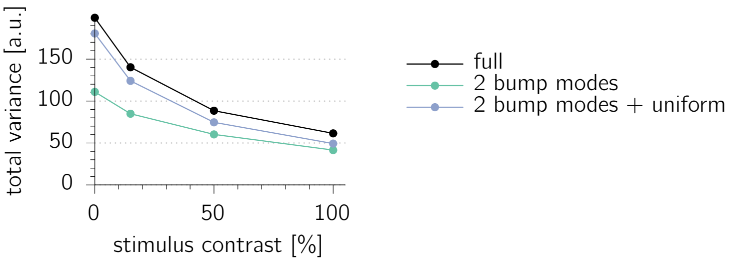

Figure S8. Related to Figure 5. Bump kinetics capture a substantial amount of variability in the ring SSN model. Black: total V_m variance in the ring SSN (E neurons) as a function of stimulus contrast. This is compared to the total variance captured by the two main modes of bump kinetics (green), and by a basis of 3 vectors formed by the same two modes + the uniform mode orthogonalized against the other two (blue). This three-dimensional subspace is virtually identical to the subspace spanned by the top three principal components of V_m fluctuations, at all stimulus contrasts, but yields a more interpretable basis. Note that while a substantial fraction of variability *suppression* with increasing stimulus contrast is due to quenched fluctuations in the uniform mode (difference between blue and green curves), the two modes of bump kinetics alone capture most of the variance at high contrast. Also note that the amount of variance captured by these linear projections is slightly smaller than that captured by the full, nonlinear fit shown in Figure 5A.

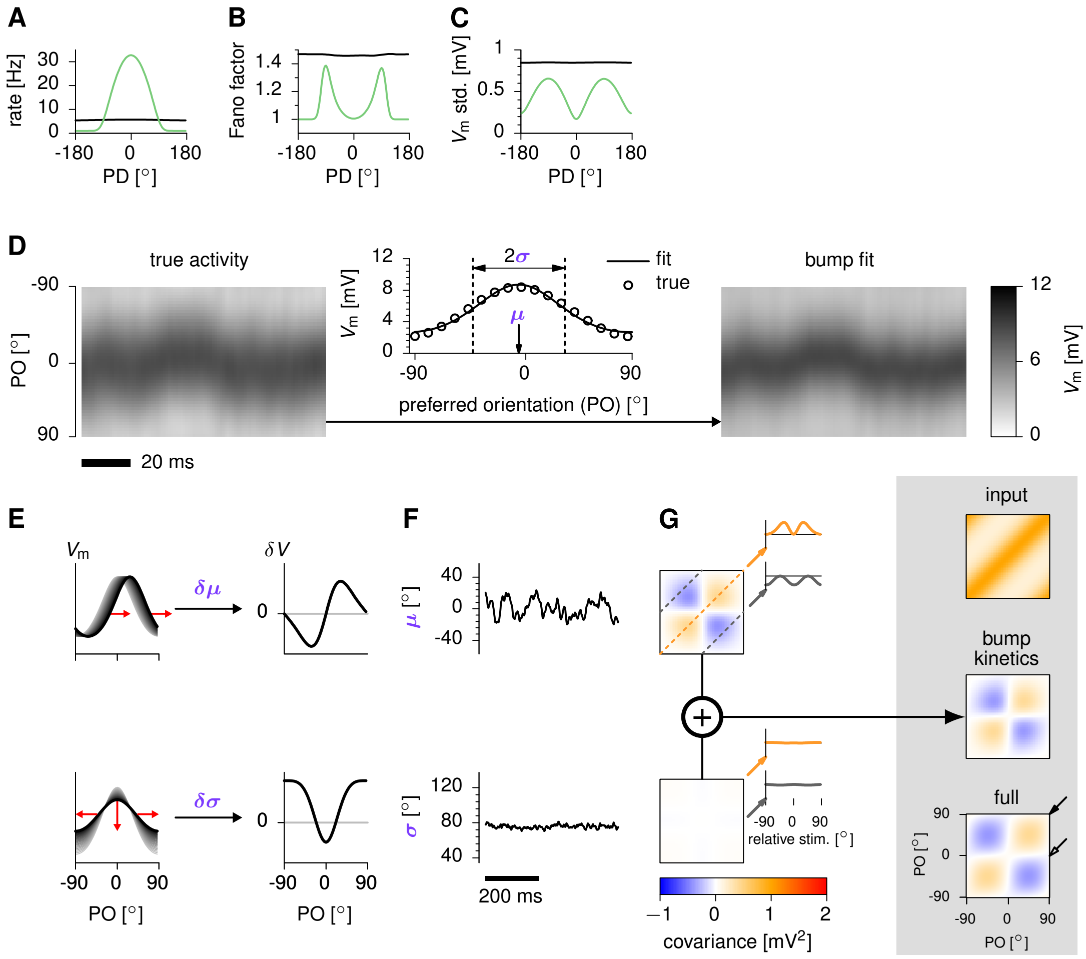

Figure S9. Related to Figures 5-6. Activity variability in a ring *multi-attractor* network. (**A–C**) Tuning of mean firing rates, Fano factors, and V_m std. in spontaneous (c = 0, black) and evoked (c = 3, green) conditions. (**D–G**) Analogous to Figure 5A-D, for the ring *attractor* network. By a large margin, the dominant contributor to activity variability in this network for a strong stimulus is the sideways jittering of the activity bump (E-G, top), with an almost complete absence of variability in the width of the bump (E-G, bottom).

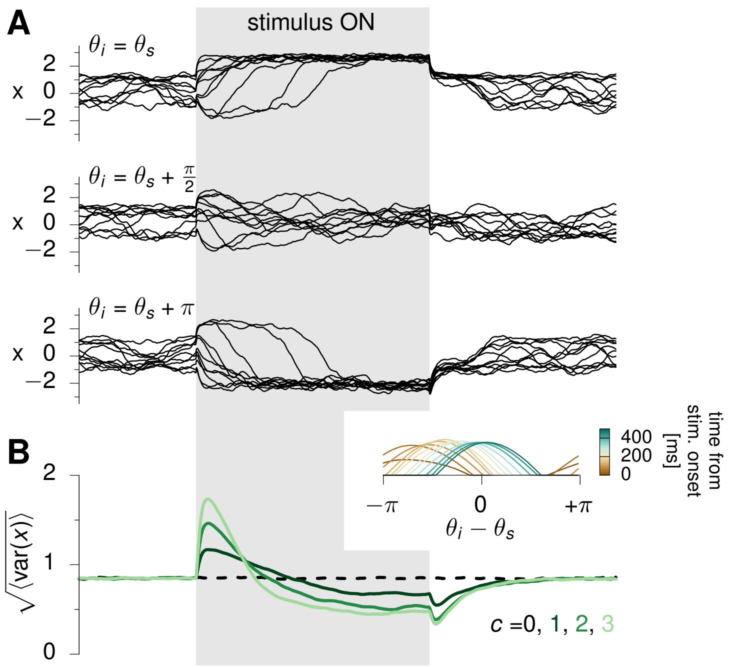

Figure S10. Related to Figure 7. Dynamics of variability quenching in the ring multi-attractor model. **(A)** Sample membrane potentials (10 trials, individual lines showing V_i in Equation 13; STAR Methods) for a neuron tuned to the stimulus direction (top), to the orthogonal direction (middle) and to the opposite direction (bottom). Here the stimulus, θ_s, and thus also the preferred stimuli of neurons, θ_i, are defined to be between $-\pi$ and π. Stimulus strength is stepped up for a 1-sec duration (gray shading; $c = 2$). **(B)** Time course of the standard-deviation across trials of the membrane potential, averaged across neurons, for different values of input strength, c (color coded). The inset shows the spatial profile of network activity (firing rate r, Equation 14; STAR Methods) in an example trial over 400 ms following stimulus onset (time is color coded). First, the activity bump quickly scales up and then it slowly moves from its initial random location (here, around $-3\pi/4$) to the new position determined by the stimulus (at $\theta_s = 0$). The initial growth of bump amplitude increases variability because of the random location of the bump across trials, while the slow movement to a location that is the same across trials decreases variability.

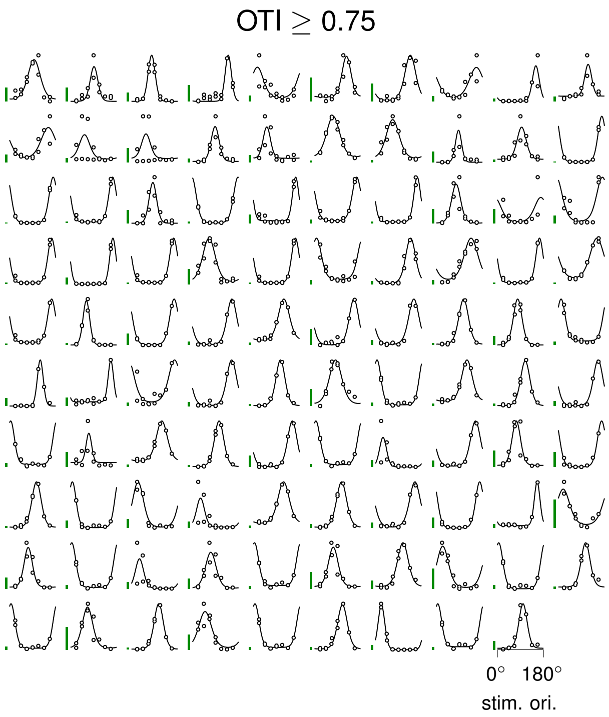

Figure S11. Related to Figures 4, 6, and 7. Tuning curves of V1 cells analysed from the data set of Ecker et al. (2010). Only cells with an orientation tuning index (OTI) of at least 0.75 are shown here and were included in subsequent analyses (STAR Methods). Green vertical scale bars: 2 spikes/sec. Note that some cells were also direction selective, hence responded at two different levels at some orientations depending on the motion direction.

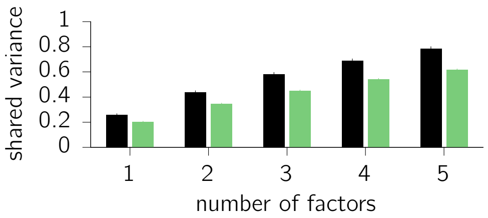

Figure S12. Related to Figure 4. Parameter-dependence of shared variability suppression as measured by factor analysis. Reduction of shared variability from spontaneous (black) to stimulus-evoked (green) activity in the monkey V1 dataset (Ecker et al., 2010), as estimated via factor analysis (STAR Methods). The x-axis shows the number of latent factors used. Only conditions with at least 8 simultaneously recorded well-isolated cells were analyzed (151 conditions).
