# Supplementary material for: The Dynamical Regime of Sensory Cortex: Stable Dynamics around a Single Stimulus-Tuned Attractor Account for Patterns of Noise Variability
Source: Neuron. 2018 May 16;98(4):846–860.e5. doi: 10.1016/j.neuron.2018.04.017 (PMC5971207; doi:10.1016/j.neuron.2018.04.017)
Supplement: Document S2. Article plus Supplemental Information [file mmc2.pdf]

Article

Neuron

The Dynamical Regime of Sensory Cortex: Stable Dynamics around a Single Stimulus-Tuned Attractor Account for Patterns of Noise Variability

Highlights

- A simple network model explains stimulus-tuning of cortical variability suppression
- Inhibition stabilizes recurrently interacting neurons with supralinear I/O functions
- Stimuli strengthen inhibitory stabilization around a stable state, quenching variability
- Single-trial V1 data are compatible with this model and rules out competing proposals

Authors

Guillaume Hennequin,
Yashar Ahmadian, Daniel B. Rubin,
Máté Lengyel, Kenneth D. Miller

Correspondence

g.hennequin@eng.cam.ac.uk

In Brief

Stimuli suppress cortical correlated variability. Hennequin et al. show that a cortical operating regime of inhibitory stabilization around a single stable state—the “stabilized supralinear network”—explains this suppression’s tuning and timing, while alternative proposed regimes do not.

Hennequin et al., 2018, Neuron *98*, 846–860
May 16, 2018 © 2018 The Author(s). Published by Elsevier Inc.
https://doi.org/10.1016/j.neuron.2018.04.017

CellPress

The Dynamical Regime of Sensory Cortex: Stable Dynamics around a Single Stimulus-Tuned Attractor Account for Patterns of Noise Variability

Guillaume Hennequin,[1,10,*] Yashar Ahmadian,[2,3,4,5,9] Daniel B. Rubin,[2,6,9] Máté Lengyel,[1,7,8] and Kenneth D. Miller[2,3,8]
[1]Computational and Biological Learning Lab, Department of Engineering, University of Cambridge, Cambridge CB2 1PZ, UK
[2]Center for Theoretical Neuroscience, College of Physicians and Surgeons, Columbia University, New York, NY 10032, USA
[3]Department of Neuroscience, Swartz Program in Theoretical Neuroscience, Kavli Institute for Brain Science, College of Physicians and Surgeons, Columbia University, New York, NY 10032, USA
[4]Centre de Neurophysique, Physiologie, et Pathologie, CNRS, 75270 Paris Cedex 06, France
[5]Institute of Neuroscience, Department of Biology and Mathematics, University of Oregon, Eugene, OR 97403, USA
[6]Department of Neurology, Massachusetts General Hospital and Brigham and Women's Hospital, Harvard Medical School, Boston, MA 02115, USA
[7]Department of Cognitive Science, Central European University, 1051 Budapest, Hungary
[8]Senior author
[9]These authors contributed equally
[10]Lead Contact
*Correspondence: g.hennequin@eng.cam.ac.uk
https://doi.org/10.1016/j.neuron.2018.04.017

SUMMARY

Correlated variability in cortical activity is ubiquitously quenched following stimulus onset, in a stimulus-dependent manner. These modulations have been attributed to circuit dynamics involving either multiple stable states ("attractors") or chaotic activity. Here we show that a qualitatively different dynamical regime, involving fluctuations about a single, stimulus-driven attractor in a loosely balanced excitatory-inhibitory network (the stochastic "stabilized supralinear network"), best explains these modulations. Given the supralinear input/output functions of cortical neurons, increased stimulus drive strengthens effective network connectivity. This shifts the balance from interactions that amplify variability to suppressive inhibitory feedback, quenching correlated variability around more strongly driven steady states. Comparing to previously published and original data analyses, we show that this mechanism, unlike previous proposals, uniquely accounts for the spatial patterns and fast temporal dynamics of variability suppression. Specifying the cortical operating regime is key to understanding the computations underlying perception.

INTRODUCTION

Neuronal activity throughout cerebral cortex is variable, both temporally during epochs of stationary dynamics and across repeated trials despite constant stimulus or task conditions (Softky and Koch, 1993; Churchland et al., 2010). Moreover, variability is modulated by a variety of factors, most notably by external sensory stimuli (Churchland et al., 2010; Kohn and Smith, 2005; Ponce-Alvarez et al., 2013), planning and execution of limb movements (Churchland et al., 2006, 2010), and attention (Cohen and Maunsell, 2009; Mitchell et al., 2009). Modulation of variability occurs at the level of single-neuron activity, e.g., membrane potentials or spike counts (Finn et al., 2007; Poulet and Petersen, 2008; Cardin et al., 2008; Gentet et al., 2010; Churchland et al., 2010; Tan et al., 2014), as well as in the patterns of joint activity across populations, as seen in multiunit activity or the local field potential (LFP) (Tan et al., 2014; Chen et al., 2014; Lin et al., 2015). Variability modulation shows stereotypical patterns. First, the onset of a stimulus quenches variability overall and, in particular, correlated variability in firing rates that is "shared" across many neurons (Lin et al., 2015; Goris et al., 2014; Ecker et al., 2014, 2016; Churchland et al., 2010). Moreover, the degree of variability reduction can depend systematically on the tuning of individual cells. For example, in area MT, variability is quenched more strongly in cells that respond best to the stimulus, and correlations decrease more among neurons with similar stimulus preferences (Ponce-Alvarez et al., 2013; Lombardo et al., 2015). Although these patterned modulations of variability are increasingly included in quantitative analyses of neural recordings (Renart and Machens, 2014; Orbán et al., 2016), it is still unclear what they imply about the dynamical regime in which the cortex operates.

There have been two dynamical mechanisms proposed to explain selected aspects of the modulation of cortical variability by stimuli. In "multi-attractor" models, the network operates in a multi-stable regime in the absence of a stimulus, such that it noisily wanders among multiple possible stable states ("attractors"). This wandering among attractors occurs in a concerted way across the population, resulting in substantial shared variability (Figure 1A, top). Stimuli then suppress this

 © 2018 The Author(s). Published by Elsevier Inc.
This is an open access article under the CC BY license (http://creativecommons.org/licenses/by/4.0/).

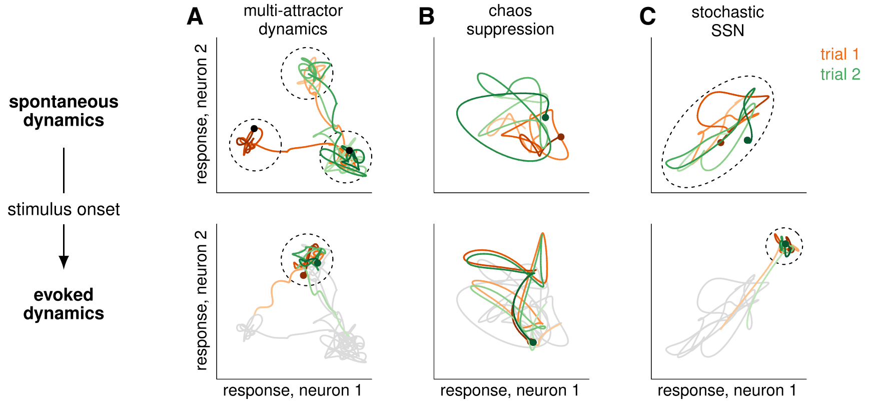

Figure 1. Three Different Dynamical Regimes that Could Explain Variability Modulation by Stimuli

(A–C) Two schematic neural trajectories (red and green) corresponding to two separate trials are plotted for each dynamical regime, before (top) and after (bottom) stimulus onset. Spontaneous activity is redrawn in gray beneath evoked activity to allow comparison of variability. Dotted ellipses outline activity covariances around the fixed point(s) of the dynamics (if any exist).

(A) Multi-attractor dynamics: spontaneous activity wanders stochastically between a set of attractor states (three shown), resulting in large trial-by-trial variability (top). Stimulus onset constrains fluctuations to the vicinity of a single attractor, reducing variability across both time and trials (bottom).

(B) Chaos suppression: chaos yields large across-trial variability in spontaneous dynamics (top), which is suppressed by the stimulus, leading to a reduction of variability across trials but not necessarily across time (bottom).

(C) Stochastic SSN: both spontaneous and evoked dynamics are stable with a single fixed point, but the stimulus can shrink the effective size of the basin of attraction of the fixed point (as well as shifting its location), resulting in a reduction of both across-time and across-trial variability.

shared variability by pinning fluctuations to the vicinity of one particular attractor (Figure 1A, bottom; Blumenfeld et al., 2006; Litwin-Kumar and Doiron, 2012; Deco and Hugues, 2012; Burak and Fiete, 2012; Ponce-Alvarez et al., 2013; Doiron and Litwin-Kumar, 2014; Mochol et al., 2015). In chaotic network models (Sompolinsky et al., 1988), firing rates exhibit strong chaotic fluctuations, and certain types of stimuli can suppress chaos by forcing the dynamical state of the network to follow a specific trajectory, thus quenching across-trial variability (Figure 1B; Molgedey et al., 1992; Bertschinger and Natschläger, 2004; Sussillo and Abbott, 2009; Rajan et al., 2010). While both the multi-attractor and the chaotic mechanisms can explain the general phenomenon of stimulus-induced reduction of variability, only the former has been proposed to explain the stimulus-tuning of variability reduction. However, even in that case, a considerable fine-tuning of parameters or very strong noise was needed to keep the network in the regime with multiple attractors, such that the system stays near attractors, yet noise can move the system between them (Ponce-Alvarez et al., 2013).

Here, we explore a qualitatively different regime of cortical dynamics. We describe activity fluctuations as being driven by noise but shaped by nonlinear, recurrent interactions. In contrast to previous models, our network operates around a single stable point that depends on the stimulus (Figure 1C). Crucially, individual neurons have supralinear (expansive) input/output functions. This causes the gains of neurons, and thus the effective synaptic strengths in the network, to increase with network activation. This is a stochastic generalization of the stabilized supralinear network (SSN) model that has successfully accounted for a range of phenomena related to the stimulus dependence of trial-averaged responses in visual cortex (Ahmadian et al., 2013; Rubin et al., 2015). Introducing stochasticity allows us to model the variability of responses and thus use data on neural variability to identify hallmarks of this regime and distinguish it from previous proposals.

In our network, stimulus-dependent changes in effective connectivity shape the magnitude and structure of activity fluctuations in the network. Specifically, stimuli change the balance of two opposing effects of recurrent network dynamics on variability: hidden feedforward interactions ("balanced amplification"; Murphy and Miller, 2009; Hennequin et al., 2014) and recurrent excitation, which amplify variability and dominate for very weak (spontaneous) inputs; and stabilizing inhibitory feedback, which quenches variability (Renart et al., 2010; Tetzlaff et al., 2012) and dominates for stronger inputs.

By studying this network mechanism in a progression of recurrent architectures with increasingly detailed structure, we find that it naturally and robustly explains the modulation of shared cortical variability by stimuli, including its tuning dependence. We first analyze variability in the simplest instantiation of the model, with two unstructured populations of excitatory (E) and inhibitory (I) cells, and find that an external stimulus can strongly modulate the variability of population activities. In particular, the model predicts stimulus-induced quenching of variability, as well as a reduction of the low-temporal-frequency coherence between local population activity and single-cell responses, as found experimentally (Poulet and Petersen, 2008; Churchland et al., 2010; Chen et al., 2014; Tan et al., 2014). Next, we extend our analysis to a more detailed architecture with structured connectivity to account for the tuning-dependent modulations of Fano factors and noise correlations by stimuli. Critically, these results reveal robust qualitative differences between the predictions of our model and those of previously proposed network mechanisms, based on multi-attractor or chaotic dynamics, for both the spatial patterns and temporal dynamics of variability suppression. We tested these predictions against experimental data and found the SSN model to be the most consistent with previously analyzed data from primary visual cortex (V1) and MT (Churchland et al., 2010; Ponce-Alvarez et al., 2013) as well as with our novel analyses of published V1 recordings in the awake monkey (Ecker et al., 2010). Such comparisons of different models are crucial for guiding future experiments that can make targeted measurements to fully resolve the dynamical regime in which the cortex operates—a key first step in identifying the computational strategies underlying perception.

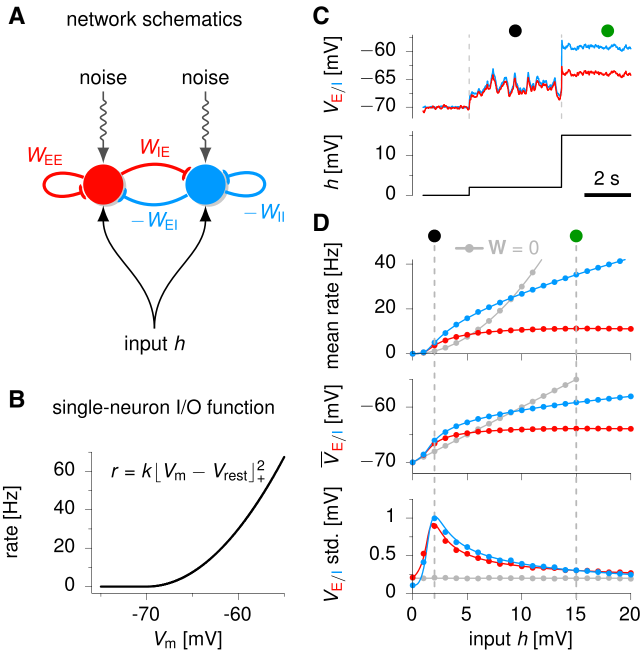

Figure 2. Activity Variability in a Reduced, Two-Population Stochastic SSN

(A) The network is composed of two recurrently connected units, summarizing the activity of two populations of excitatory (red) and inhibitory (blue) neurons. Both units receive private input noise and a common constant input h.

(B) Threshold-quadratic neural input/output function determining the relationship between membrane potential and momentary firing rate of model neurons (Equation 2).

(C) Sample $V_{E/I}$ traces for the two units (top), as the input is increased in steps from $h = 0$ to 2 mV to 15 mV (bottom).

(D) Dependence of population activity statistics on stimulus strength h. Top: mean E (red) and I (blue) firing rates; middle: mean $V_{E/I}$; bottom: standard deviation of $V_{E/I}$ fluctuations. The comparison with a purely feedforward network ($\mathbf{W} = \mathbf{0}$) receiving the same input h is shown in gray. Dots are based on numerical simulations of 500 trials. Solid lines show analytical approximations (Hennequin and Lengyel, 2016).

RESULTS

We used a standard model to study the dynamical evolution of momentary firing rates in a recurrently coupled network of excitatory and inhibitory neurons (Figure 2A; Dayan and Abbott, 2001; see also STAR Methods). In this model, neurons integrate their external and recurrent inputs linearly in their membrane potentials, V_m, but their output firing rates, r, are a nonlinear function of the voltage: $r = f(V_m)$ (Figure 2B). Crucially, we studied variants of this model in which the nonlinearity f is an expansive (supralinear) function (Figure 2B) and in which inhibition was both sufficiently fast and strong and appropriately structured to stabilize the network in the face of recurrent excitation and the supralinear input/output function. This is the stabilized supralinear network (SSN) model (Ahmadian et al., 2013). In order to study response variability, we added to this model a stochastic component (slow noise) in the membrane potential dynamics of all cells. Stabilization meant that the network operated around a single steady state, albeit a stimulus-dependent one.

Real neurons, of course, have an input/output function that ultimately saturates. We focus on an expansive, non-saturating input/output function because V1 cortical neurons show such a relationship between mean voltage and firing rate across their full dynamic range, without saturation even for the strongest visual stimuli (Priebe and Ferster, 2008). Thus, saturation does not appear to play a role in stabilizing cortical activity, a fact that we capture by using a non-saturating input/output function. Such an expansive input/output function arises in spiking neurons when their firing is driven by voltage fluctuations, with the mean voltage sub- or peri-threshold (Hansel and van Vreeswijk, 2002; Miller and Troyer, 2002), a firing regime that produces the highly variable spiking seen in cortical neurons (Troyer and Miller, 1997; Amit and Brunel, 1997). We assume that the voltage fluctuations giving rise to the expansive input/output function are fast compared to the timescales of variability studied here and do not explicitly model them.

We focused on analyzing how the intrinsic dynamics of the network shaped fixed input noise to give rise to stimulus-dependent patterns of response variability. We studied a progression of connectivity architectures of increasing complexity, all involving two separate populations of excitatory and inhibitory neurons. We also validated our results in large-scale simulations of spiking neuronal networks.

Variability of Population Activity: Modulation by External Input

We first considered a simple circuit motif: an excitatory (E) unit and an inhibitory (I) unit, recurrently coupled and receiving the same mean external input h as well as their own independent noise (Figure 2A). In this reduced model, the two units represent two randomly connected populations of E and I neurons, a canonical model of cortical networks (Amit and Brunel, 1997; Vogels et al., 2005). Thus, their time-varying activity, $V_E(t)$ and $V_I(t)$, represents the momentary population-average membrane potential of all the E and I cells, respectively. Despite its simplicity, this architecture accounted well for the overall population response properties in the larger networks, with more detailed connectivity patterns, that we analyzed later.

Activity in the network exhibited temporal variability due to the stochastic component of the dynamics. We found that this (correlated) variability of V_E and V_I fluctuations, together with their means, $\overline{V}_{E/I}$, was strongly modulated by the external steady input h (Figures 2C and 2D). When $h = 0$, there was no input to drive the network, and V_E and V_I both hovered around $V_{rest} = -70$ mV, fluctuating virtually independently, with standard deviations essentially matching those that would arise without recurrent connections (gray line in Figure 2D, bottom). For a somewhat larger input, $h = 2$ mV, both E and I populations fired at moderate rates (3–4 Hz) (Figure 2D, top), but now also exhibited large and synchronous population V_m fluctuations (Figure 2C, black circle mark). For yet larger inputs ($h = 15$ mV), fluctuations remained highly correlated, but their magnitude was strongly quenched (Figure 2C, green circle mark).

Figure 2D shows how the temporal (or, equivalently, the across-trial) mean and variability of activities varied over a broad range of input strengths. We observed that population mean V_m increased monotonically with growing external input, first linearly

or supralinearly for small inputs, but strongly sublinearly for larger inputs, with $\overline{V}_I$ growing faster than $\overline{V}_E$ (Figure 2D, middle; Ahmadian et al., 2013; Rubin et al., 2015). In contrast, variability in both V_E and V_I typically increased for small inputs, peaking around this transition between supralinear and sublinear growth, and then decreased with increasing input (Figure 2D, bottom). Importantly, input modulation of variability required recurrent network interactions. This was revealed by comparing our network to a purely feedforward circuit that exhibited qualitatively different behavior (Figure 2D, gray). In the feedforward circuit, mean V_m remained linear in h, so that mean rates rose quadratically with V_m or h (reflecting the input/output nonlinearity; Figure 2B), and fluctuations in V_m no longer depended on the input strength.

Variability Suppression with a Single Stable State Is a Robust Phenomenon

In order to demonstrate that the overall dynamical regime of the stabilized supralinear network, rather than just a particular instantiation of our model, underlies variability modulation, we used a combination of numerical simulations and analytical results to confirm the robustness of our findings.

We simulated 1,000 model networks with random parameter values within wide brackets. We found that variability suppression was robust over a broad range of network parameters (connection weights, input strengths and correlations, and the exponent and scale of the firing-rate nonlinearity), as long as they ensured dynamical stability even for strong inputs (Figures S1 and S2). Although the precise amplitude and position of the peak of V_m variance depended on network parameters, the overall non-monotonic shape of variability modulation was largely conserved. In particular, we could show analytically that variability suppression occurs earlier (for smaller input h) in networks with strong connections or, for fixed overall connection strength, in networks that are more dominated by feedback inhibition (Methods S3). More generally, we found that the firing rates at the peak of variability are typically low (2.5 Hz on average over a thousand randomly parameterized stable networks and below 6 Hz for 90% of them; *cf.* Methods S2). As these rates are comparable to cortical spontaneous firing rates, this predicts that increased sensory drive should generally result in variability quenching in cortical LFPs.

In order to better understand the robustness of variability suppression in the model, we took advantage of the fact that our network was characterized by a single attractor at each level of the input, h, and analyzed the dynamics of small activity fluctuations, $\delta\mathbf{V}$, around this stable state (such that $\mathbf{V} = \overline{\mathbf{V}}(h) + \delta\mathbf{V}$, where $\overline{\mathbf{V}}(h)$ is the mean activity in the stable state; STAR Methods). These dynamics are governed by a set of effective connection weights, $\mathbf{W}^{\mathrm{eff}}$, that quantify the impact of a small momentary change in the V_m of the presynaptic neuron on the total input to its postsynaptic partner. The dependence of the effective connection weights on the stable state and thus on the external input, h, that determines the stable state is simply given by:

$$W_{ij}^{\mathrm{eff}}(h) \propto W_{ij}\, f'\left[\overline{V}_j(h)\right] \quad \text{(Equation 1)}$$

where W_{ij} is the strength of the "biophysical" connection from unit j to unit i, and f' is the slope of the single-neuron firing-rate nonlinearity at the stable state. Importantly, f' increases with increasing $\overline{V}(h)$, because f is an expansive, convex nonlinearity (Figure 2B). Thus, in general, effective connectivity increases with increasing h, reflecting the growth of $\overline{V}(h)$ (Figure 2D, middle).

An increase in effective connectivity can have conflicting effects: it can increase excitatory or driving effects that amplify fluctuations and increase variability (Murphy and Miller, 2009; Hennequin et al., 2014), but it can also increase inhibitory feedback, suppressing fluctuations and decreasing variability (Renart et al., 2010; Tetzlaff et al., 2012). Thus, understanding how changes in effective connectivity translate into changes in variability required further analysis (Methods S3). We found that the net behavior of the network indeed included a combination of both effects (Figure S3). As the input grew from zero, variability first rapidly increased, due primarily to the growth of effective feedforward weights ("balanced amplification"; Murphy and Miller, 2009) but also of recurrent excitatory loops. Then, beginning at firing rates comparable to spontaneous activity as described above, variability steadily decreased with increasing stimulus strength due to increasingly strong inhibitory feedback (Figure 2D, bottom).

Crucially, we were able to show analytically that variability quenching effects must ultimately dominate, leading to progressively stronger quenching of variability as the input increases. This is due to the faster growth of I activity relative to E activity in the network, which is a robust outcome of dynamic stabilization by feedback inhibition (Figure S1; Ahmadian et al., 2013; Rubin et al., 2015) and which has been observed in rodent S1 (Shao et al., 2013) and V1 (Adesnik, 2017). We also found that ignoring the variability-increasing effects, which are characteristic of excitatory-inhibitory dynamics (Kriener et al., 2008; Murphy and Miller, 2009) and thus largely absent from models that do not include separate excitatory and inhibitory populations, can fail to capture the full extent of variability modulation and lead to an underestimation of the level of spontaneous variability obtained at zero-to-weak input levels (Figure S4).

Variability Quenching and Synchronization in Single Neurons

In order to study variability in single neurons and at the level of spike counts, we implemented the two-population architecture of Figure 2A in a network of spiking neurons (Figure 3; STAR Methods). The network consisted of 4,000 E neurons and 1,000 I neurons, randomly connected with low probability and with synaptic weights chosen such that the overall connectivity matched that of the reduced model. Each neuron emitted action potentials stochastically with an instantaneous rate given by Equation 3 (this additional stochasticity accounted for the effects of unmodelled fluctuations in synaptic inputs that occur on timescales faster than the 30 ms effective time resolution of our model; Methods S4). The external input to the network again included a constant term, h, and a noise term that was temporally correlated on a 50 ms timescale with uniform spatial correlations of strength 0.2.

At the population level, the network behaved as predicted by the reduced model. Neurons fired irregularly (Figure 3A, top), with firing rates that grew superlinearly with small input h but

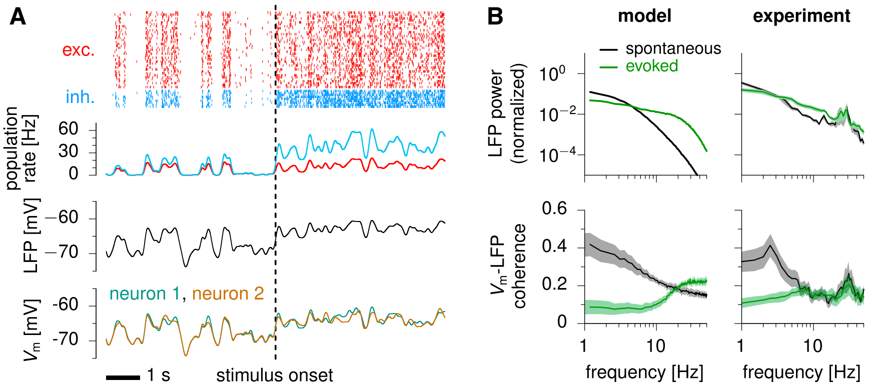

Figure 3. Modulation of Variability in a Randomly Connected Stochastic Spiking SSN

(A) Top: raster plot of spiking activity, for 40 (out of 4,000) excitatory neurons (red) and 10 (out of 1,000) inhibitory neurons (blue). Upper middle: momentary E and I population firing rates. Lower middle: LFP (momentary population-averaged V_m). Bottom: V_m of two randomly chosen excitatory neurons. The dashed vertical line marks the onset of stimulus, when h switches from 2 mV to 15 mV. Population firing rates, LFP, and V_m traces were smoothed with a Gaussian kernel of 50 ms width.

(B) Top, normalized LFP power in spontaneous (black) and evoked (green) conditions; bottom, average (± SEM) spectral coherence between single-cell V_m and the LFP; left, model; right, data from V1 of the awake monkey, reproduced from Tan et al. (2014).

sublinearly with stronger input (Figure S5). Moreover, fluctuations in E and I population activities were strongly synchronized (Figure 3A, upper middle), and LFP variability decreased with increasing h (Figure 3A, lower middle). Importantly, variability quenching also occurred at the level of individual neurons' V_m, accompanied by a reduction of pairwise correlations (Figure 3A, bottom; these required that single neurons shared part of their input noise; Methods S3).

The model primarily suppressed shared rather than private (to individual neurons) variability (Figure S5), as in experiments (Churchland et al., 2010). This was because the spatially uniform average connectivity of the network meant that its dynamics were only significantly coupled to patterns of uniform activity across E or across I cells. These patterns were thus the ones affected by stimulus-induced changes in effective connectivity (Figure S3). Correlated noise drove such uniform patterns so that they carried significant variability. Thus, these shared excitatory and inhibitory activity patterns behaved as the activity of the individual units of the previous reduced two-population model, and so variability suppression in the reduced model implied the suppression specifically of shared variability in this more detailed model.

Our model also accounted for the stimulus-induced modulation of the power spectrum and cross-coherence of LFP and single-cell V_m fluctuations, as observed in V1 of the awake monkey (Figure 3B; Tan et al., 2014). Strong external input reduced the LFP power at low frequencies, due to enhanced effects of feedback inhibition; increased it at intermediate frequencies, due to the faster timescales associated with relatively enhanced inhibition; and also increased it at high frequencies, due to the larger firing rates, which contributed additional, high-frequency fluctuations in synaptic drive (Figure 3B, top left). This asymmetric modulation of LFP power at low and high frequencies is also seen in experiments (Figure 3B, top right). Moreover, as increasing inputs suppressed variability at the population level, the private noise in the activity of each neuron had a proportionately larger contribution to its overall variability, leading to a drop in pairwise correlations (Figure 3A) and V_m-LFP coherence specifically at low frequencies where the suppression of population variability occurred, as seen in experiments (Figure 3B, bottom).

Stimulus-Tuning of Variability Suppression in V1

Neuronal recordings in visual areas have shown that Fano factors drop at the onset of the stimulus (drifting gratings or plaids) in almost every neuron, which was well accounted for by the randomly connected network we studied above. However, in the experiments, variability did not drop uniformly across cells, but exhibited systematic dependencies on stimulus tuning (Ponce-Alvarez et al., 2013; Lombardo et al., 2015; Lin et al., 2015). This could not be explained by randomly connected architectures, so we extended our model to include tuning dependence in connectivity and input noise correlations.

We studied an architecture in which the preferred stimulus of E/I neuron pairs varied systematically around a "ring" representing an angular stimulus variable, such as stimulus orientation in V1 or motion direction in MT (Figure 4A; STAR Methods). We describe the case in which the variable is orientation, which ranges from 0 to 180°; identical results describe direction if all angles are doubled. The average input to a cell (either E or I) was composed of a constant baseline, which drove spontaneous activity in the network, and a term that depended on the angular distance between the stimulus orientation and the preferred orientation (PO) of the cell, and that scaled with image contrast, c (Figure 4C). Input noise correlations depended on tuning differences (STAR Methods): cells with more similar tuning received more strongly correlated inputs. The strength of recurrent connections depended on the difference in preferred orientation between pre- and postsynaptic neurons and whether they were excitatory or inhibitory (Figure 4B).

The bump of stimulus-driven input drove a similar, but narrower, bump of network response (Figures 4D and 4G). Although this architecture appears similar to a form of multi-attractor model that has a continuum of attractors—a bump of activity that (in the absence of stimuli) can be centered at any location (the so-called "ring attractor model"; Goldberg et al., 2004; Ben-Yishai et al., 1995; Ponce-Alvarez et al., 2013)—our model was actually quite different. While multi-attractor networks show a bump of sustained activity even once the stimulus is removed (leaving only non-specific background excitation), in our network the bump of activity depends on the similar bump of stimulus-driven input. When the stimulus is removed, our

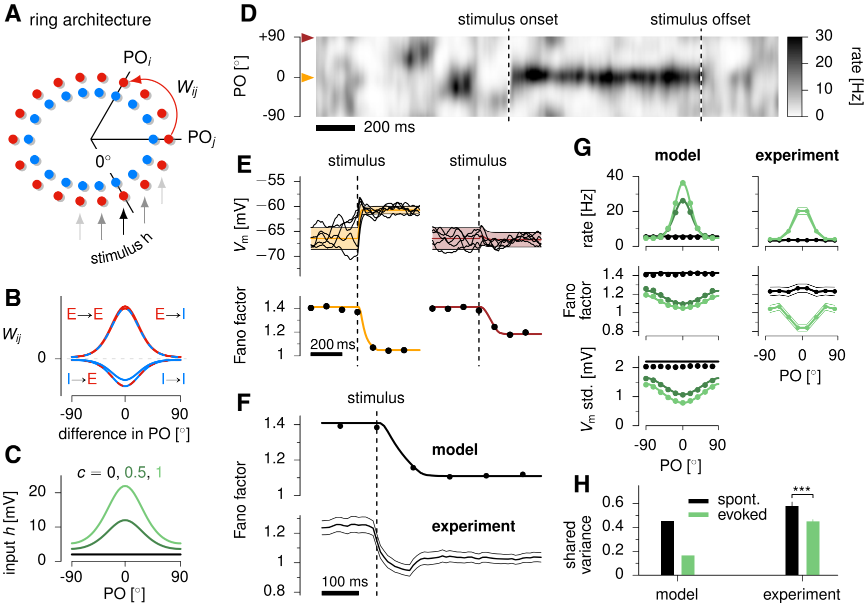

Figure 4. Modulation of Variability in a Stochastic SSN with a Ring Architecture

(A) Schematics of the ring architecture. Excitatory (red) and inhibitory neurons (blue) are arranged on a ring, their angular position indicating their preferred stimulus (expressed here as preferred stimulus orientation, PO). The stimulus is presented at 0°.

(B) Synaptic connectivities all follow the same circular Gaussian profiles with peak strengths that depend on the type of pre- and post-synaptic populations (excitatory, E, or inhibitory, I).

(C) Each neuron receives a constant input with a baseline (black line, 0% contrast), which drives spontaneous activity, and a tuned component with a bell-shaped dependence on the neuron's preferred orientation and proportional to contrast, c (dark and light green, 50% and 100% contrast, respectively). Neurons also receive spatially and temporally correlated noise, with spatial correlations that decrease with tuning difference (see Figure 5D).

(D) Single-trial network activity (E cells), before and after the onset of the stimulus (100% contrast). Neurons are arranged on the y axis according to their preferred stimuli.

(E) Reduction in membrane potential variability across trials: membrane potential traces in 5 independent trials (top) and Fano factors (bottom) for an E cell tuned to the stimulus orientation (left) or tuned to the orthogonal orientation (right). For V_m, orange and brown lines and shading show (analytical approximation of) across-trial mean ± SD.

(F) Reduction of average spike count Fano factor in the population following stimulus onset in the model (top) and experimental data (bottom). Spikes were counted in 100 ms time windows centered on the corresponding time points.

(G) Mean firing rates (top), Fano factors (middle), and std. of voltage fluctuations (bottom) at different contrast levels as a function of the neuron's preferred stimulus in the model (left) and, for rate and Fano factor, experimental data (right, averaged across 99 neurons). Colors indicate different contrast levels (model: colors as in C; data: black, spontaneous, green, 100% contrast).

(H) Shared variability in normalized spike counts, as estimated via factor analysis (STAR Methods; Churchland et al., 2010), before (spontaneous, black) and after stimulus onset (evoked, green) in the model (left) and experimental data (right). Dots in (F) and (G) are based on numerical simulations of 500 trials. For the model, colored lines and shaded areas in (E) and solid lines in (F) and (G) show analytical approximations (Hennequin and Lengyel, 2016). Experimental data analyzed in (F)–(H) are from awake monkey V1 (Ecker et al., 2010), with error bars denoting 95% CI.

network returns within a single membrane time constant to a homogeneous level of baseline activity, driven by the homogeneous baseline input (Figure 4D). As we show below, this dynamical regime is also characterized by fundamentally different patterns of response variability than multi-attractor dynamics.

We applied this model to study the stimulus dependence of variability quenching in V1 and compared our results to a new analysis we performed of previously published recordings in V1 of the awake monkey (Ecker et al., 2010). In the absence of visual input (0% contrast), the network exhibited spatially patterned fluctuations in momentary firing rates around a few Hz (Figure 4D) with large across-trial variability in single-cell V_m (Figure 4E). In evoked conditions, the input drove a hill of network activity around the stimulus orientation as in the data (Figures 4D and 4G), resulting in approximately contrast-invariant tuning curves (Priebe and Ferster, 2008). At stimulus offset, activity rapidly decayed back to spontaneous levels with the cellular time constant (Figure 4D), as observed in cortex when thalamic input is silenced (Reinhold et al., 2015; Guo et al., 2017).

The fluctuating firing rates in spontaneous activity implied super-Poisson variability in spike counts—Fano factors greater than 1 (Figure 4F, top) —given the stochastic spiking mechanism described above (Figure 3). This was consistent with the high level of spontaneous variability in the data (Figure 4F, bottom). Both the model and the data exhibited a pronounced drop in Fano factor following stimulus onset (Figure 4F) and displayed a U-shaped tuning of variability suppression with stimulus orientation (Figure 4G, middle), such that variability suppression was stronger for cells whose preferred orientation was close to the stimulus. The model made similar predictions for variability in membrane potentials: a U-shaped profile of V_m variance suppression in stimulus-evoked conditions relative to spontaneous fluctuations (Figure 4G, bottom).

Notably, for similar reasons as in the randomly connected network (Figure 3; Figure S5), it was primarily the shared and not the private part of variability that was quenched by stimuli in the model (Figure 4H, left), and this required some degree of spatial correlations in the input noise (Figure S6). This was because the spatially smooth nature of the connectivity meant that only spatially smooth patterns of activity were strongly coupled to the network dynamics. A substantial suppression of shared variability at stimulus onset has been observed across many cortical areas (Churchland et al., 2010) as well as in our analysis of the V1 data (Figure 4H, right; Ecker et al., 2010; see also Lombardo et al., 2015).

We again explored a broad range of parameters to show that the tuning of variability suppression was a robust outcome of the

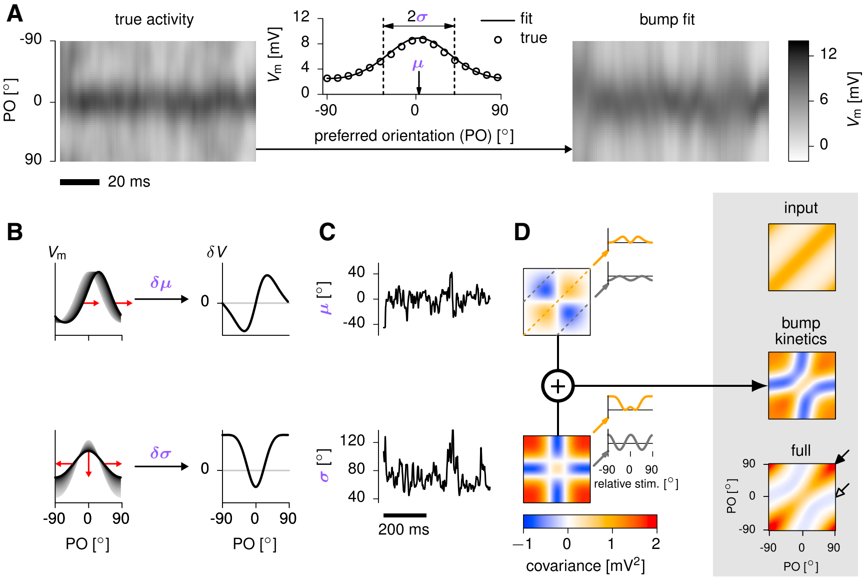

Figure 5. Low-Dimensional Bump Kinetics Explain Noise Variability in the Ring SSN

(A) Sample of V_m fluctuations across the network in the evoked condition (left, "true activity," 100% contrast), to which we fitted a circular-Gaussian function (bump) $V_i(t) = a(t)\exp[(\cos(\theta_i - \mu(t)) - 1)/\sigma^2(t)]$ across the excitatory population in each time step (center), parametrized by its location, μ, and width, σ. The amplitude of the bump, a, was chosen in each time step so as to keep total population firing rate constant. Fluctuations in location and width were independent, and the fit captured 87% of the variability in V_m (right).

(B) The two principal modes of bump kinetics: small changes (red arrows) in location (top) and width (bottom) of the activity bump result in the hill of network activity deviating from the prototypical bump (gray shadings). Plots on the right show how the activity of each neuron changes due to these modes of bump kinetics.

(C) Time series of μ and σ extracted from the fit.

(D) Ongoing fluctuations in each bump parameter contribute a template matrix of V_m covariances (color maps show covariances between cells with preferred orientation [PO] indicated on the axes of the "full" matrix, bottom right), obtained from (the outer product of) the differential patterns on the right of (B). Insets show V_m covariance implied by each template for pairs of identically tuned cells (orange, PO difference $\simeq 0^\circ$) and orthogonally tuned cells (gray, PO difference $= 90^\circ$), as a function of stimulus orientation relative to the average PO of the two cells. The two templates sum up to a total covariance matrix ("bump kinetics"), which captures the key qualitative features of the full V_m covariance matrix ("full"). The covariance matrix of the input noise ("input") is also shown above for reference. The stimulus is at 0° throughout.

model. We found that Fano factor and V_m variance were always most strongly suppressed in the neurons that were most strongly driven by the stimulus (the "dip" of the U shape) consistent with the V1 data (see above). Interestingly, there were some cases when neurons tuned to the opposite stimulus also showed a strong reduction of Fano factor (though not of membrane potential variance; Figure S7)—consistent with recent findings of an M-shaped modulation of Fano factors (and spike count correlations of similarly tuned cells) in area MT of the awake macaque (Figure S7; Ponce-Alvarez et al., 2013). However, while such an M-shaped modulation was previously attributed to marginally stable multi-attractor dynamics (Ben-Yishai et al., 1995; Ponce-Alvarez et al., 2013), our model still produced this with a single stable attractor: the spike count variability of oppositely tuned cells dropped when input tuning in the model was as narrow as, or narrower than, the tuning of recurrent connections. In this configuration, oppositely tuned cells received so small a net input on average that their membrane potential fluctuations barely crossed the threshold of the firing rate nonlinearity, thus producing very little spiking variability. In turn, this loss of firing rate variance even overcame the effect of dividing by very small firing rates in computing Fano factors for these neurons. Under the same conditions, a similar M shape was apparent for spike count correlations between similarly tuned neurons, as a function of their (common) preferred orientation (Figure S7).

Patterns of Noise Variability Arise from Low-Dimensional Bump Kinetics

Next, we analyzed the origin and mechanism of the stimulus-tuning of noise variability in the ring architecture. As mentioned above, for a fixed stimulus, the most prominent feature of population activity was a "bump" of high V_m in the cells with preferred orientations near the stimulus orientation and a lower baseline of activity in the surround (Figure 5A, left and middle). In general, variability in the bump and the baseline captured most of the network's variance and its suppression with increasing stimulus strength (Figure S8). Here and in the next section we specifically focus on the structure of the quenched noise variability after stimulus onset.

After stimulus onset, most of the shared variability (87%; Figure S8) arose from variability in the location, μ, and width, σ, of the bump of activity (Figure 5A, middle and right). Notably, fluctuations in bump amplitude and width scaled inversely with one another, as the nonlinear interactions among neurons in our network resulted in strong normalization (Ahmadian et al., 2013; Rubin et al., 2015), preserving overall activity. Each of these small transformations resulted in a characteristic pattern of momentary deviation of network activity from the mean bump (Figure 5B). In turn, these two patterns of momentary fluctuations (Figure 5C) contributed two distinct spatial covariance templates (Figure 5D). For example, sideways motion of the bump increased the firing rates of all the cells with preferred orientations on one side of the stimulus orientation and decreased firing rates for all cells on the other side (Figure 5B, top). This resulted in positive covariances between cells with preferred orientations on the same side of the stimulus orientation and negative covariances for cells on opposite sides (Figure 5D, top: μ-template; Moreno-Bote et al., 2014). Conversely, an increase in bump width (and thus a decrease in amplitude) increased the activities of cells on the flanks of the bump, tuned away from the stimulus, while

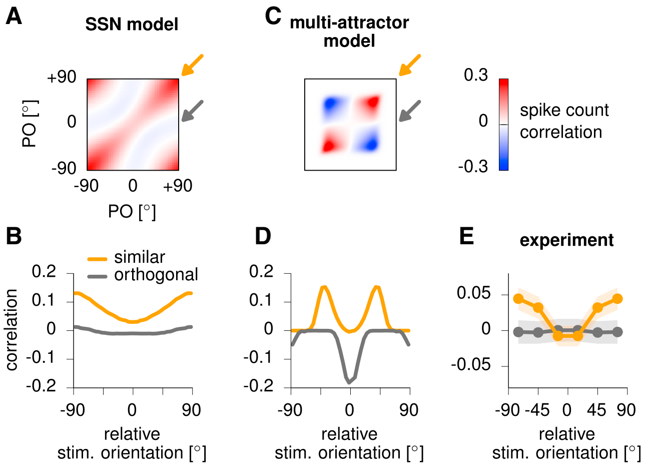

Figure 6. Stimulus Tuning of Spike Count Correlations in the Ring SSN versus the Multi-attractor Ring Model

(A) Spike count correlation matrix in the ring SSN during evoked activity (100% contrast). Color map shows correlations between cells with preferred orientation (PO) indicated on the axes, relative to stimulus orientation at 0°. Arrows indicate axes along which cell pairs are similarly (orange) or orthogonally tuned (gray). Spike count correlations along the diagonal show correlation for identically tuned cells, rather than for identical cells, and are thus less than one due to private spiking noise.

(B) Average spike count correlations in the SSN, for pairs of similarly tuned cells (orange, PO difference less than 45°) and orthogonally tuned cells (gray, PO difference greater than 45°), as a function of stimulus orientation relative to the average PO of the two cells.

(C and D) Same as (A) and (B), for the multi-attractor ring network.

(E) Same as (B) and (D), for data from awake monkey V1 (Ecker et al., 2010). Data were symmetrized for negative and positive stimulus orientations. Shaded regions denote 95% CI. SSN simulations in this figure used the same parameters as in Figures 4 and 5.

decreasing the activity of cells near the peak, tuned for the stimulus (Figure 5B, bottom). This generated positive covariances within each of these groups and negative covariances between the two groups (Figure 5D, bottom: σ-template).

Taken together, the ongoing jitter in bump location and width contributed a highly structured pattern of response covariances, which accounted for most of the structure in the full covariance matrix of the network (Figure 5D, compare "bump kinetics" with "full"). In particular, bump kinetics correctly predicted the V_m variances of cells (given by the diagonal of the full covariance matrix indicated by the filled arrow in Figure 5D), showing less variance for cells tuned to the stimulus orientation of 0° than for cells tuned to orthogonal orientations (see Figure 4G, bottom, green), and hence explained the U-shaped modulation of Fano factors (Figure 4G, middle, green). Moreover, the recurrent dynamics generated negative correlations in the V_m fluctuations of cells with orthogonal tuning, despite such pairs receiving positively correlated inputs (Figure 5D, "input" versus "bump kinetics," secondary diagonal with open arrow).

Experimental Predictions: Stimulus Tuning

For a direct comparison of the dynamical regime of the SSN with previously proposed mechanisms for variability modulation, based on marginally stable or chaotic dynamics, we first studied the predictions of the models for the spatial patterns of spike count noise correlations. Chaotic models have not (Rajan et al., 2010), and probably can not, predict the tuning of mean responses, let alone that of variability suppression, so we focused on a comparison with a multi-attractor ring model. This model has been suggested to account for stimulus-modulated changes in variability in area MT (Ponce-Alvarez et al., 2013). We matched it to our model such that it produced similar tuning curves and overall levels of variability (Figure S9).

While there were several differences apparent in the detailed correlations predicted by the two models (Figures 6A and 6C), many of these could be explained away by trivial factors that neither model captured fully. For example, the average correlation was substantially larger in the SSN than in the attractor network—but this difference could be eliminated by invoking, in the attractor model, an additional (potentially extrinsic) mechanism that adds a single source of shared variability across neurons, resulting in a uniform (possibly stimulus strength-dependent) positive offset to all correlations (Lin et al., 2015). As another example, the attractor network always exhibited an M-shaped modulation of correlations, whereas, just as for Fano factors (see above), the SSN mostly showed a U-shaped modulation but could show an M shape for particular parameters (Figure S7).

Therefore, we focused on distinctions that were robust to model details and followed from a fundamental difference of bump kinetics in the two models: in contrast to the richer patterns of variability generated by the SSN, multi-attractor dynamics showed a more limited repertoire, dominated by sideways motion of the bump with barely any fluctuations in bump width (Figure S9; Burak and Fiete, 2012). As fluctuations in bump location and width had opposite effects on the correlations between orthogonally tuned cells in the SSN model (Figure 5D insets, gray), their cancellation made these correlations only very weakly modulated by the stimulus (Figure 6A, gray arrow; Figure 6B, gray). In particular, this modulation was much shallower than that for similarly tuned cells (Figure 6A, orange arrow; Figure 6B, orange), for which variability in bump location and width had congruent effects (Figure 5D insets, orange) that added to rather than cancelled each other. In contrast, in the attractor model, there was no such cancellation even for orthogonally tuned cells due to the absence of fluctuations in bump width (Figure S9). This meant that correlations between orthogonally tuned cells were just as deeply modulated as those between similarly tuned cells (Figures 6C and 6D).

Previous reports on the stimulus-tuning of noise correlations examined only similarly tuned cells and reported mostly M-shaped modulation, which does not distinguish between the models. Therefore, we conducted our own analyses of a previously published dataset of V1 responses in the awake monkey (Ecker et al., 2010) (Figure 6E). The modulation of these correlations by the stimulus could only be accounted for by the SSN. First, we found that correlations between similarly tuned cells were significantly modulated by the stimulus (Figure 6E, orange; repeated-measures ANOVA $F(2,274) = 5.29$, $p = 0.006$), and this modulation had a U rather than an M shape. More critically, also in agreement with the predictions of the SSN but not of the attractor model, correlations between orthogonally tuned cells

were unaffected by the stimulus (Figure 6E, gray; repeated-measures ANOVA $F(2,274) = 0.04$, $p = 0.961$). While the magnitude of correlations in either model was overall larger than in the data, this simply reflected the relatively small number of neurons in the models (model correlations could be decreased without affecting the shape and extent of their stimulus tuning by substituting each model unit by several neurons with independent spiking noise).

Experimental Predictions: Temporal Dynamics of Variability Modulation

We hypothesized that the fundamentally different mechanisms responsible for variability modulation in the SSN, the multi-attractor, and the chaotic dynamical regimes (Figure 1) should be revealed in the dynamics of variability suppression at stimulus onset and of variability recovery at stimulus offset. In order to test this, we used the same models for the SSN and multi-attractor models as above, and we implemented the classical chaotic model of Rajan et al. (2010) (STAR Methods), in which variability suppression had previously been shown to occur. We then measured the across-trial variability (averaged across neurons) following the onset and offset of a step stimulus in each model (Figures 7A–7C, shaded areas), as we parametrically varied the amplitude of the stimulus and therefore the degree of variability suppression (Figures 7A–7C, dark to light colors).

In the SSN, the timescales on which both suppression and recovery of variability occurred were nearly as fast as the single-neuron time constant (20 ms in these simulations; Figures 7D and 7E, green). In contrast, in chaotic networks, both these timescales were several (4–15) times longer than the single-neuron membrane time constant (Figures 7D and 7E, blue). More importantly, recovery times were much longer than suppression times in the chaotic network and increased with increasing stimulus strength and thus increasing amount of variability suppression during the stimulus period, neither of which was the case in the SSN. In the multi-attractor network, both the dynamics of the network activity and those of variability were much slower than in the SSN (Figures 7D and 7E, red). Moreover, we found that, unlike in the SSN or the chaotic model, variability increased transiently immediately following stimulus onset (before eventually decreasing to its new steady state; Figure 7C). The cause of this behavior was the slow rotation of the activity bump from its random position at the time of stimulus onset to the location where cells' preferred orientation matched the stimulus orientation (Figure S10). Thus, we expect this behavior to be generic at least to the subclass of multi-attractor models that have a continuous ring of attractors and thus show such rotational response, which likely include those that can address the orientation- or direction-tuning of variability reduction in V1 and MT.

The timescales of variability suppression and recovery found experimentally in anaesthetized cat V1 and awake monkey MT (Figures 7D and 7E, open square and circle; Churchland et al., 2010) and by our own analysis of awake monkey V1 data (Figures 7D and 7E, dotted square; Ecker et al., 2010) were short and nearly identical. Moreover, recovery times showed little dependence on the amount of variability suppression (comparing across areas), and there was no transient increase in variability at stimulus onset (Figure 4F; Churchland et al., 2010). These results confirm the predictions of the SSN and are at odds with the dynamics of variability modulation as predicted by the multi-attractor and chaotic regimes.

DISCUSSION

We studied the modulation of variability in a stochastic, nonlinear model of cortical circuit dynamics. We focused on a simple circuit motif that captured the essence of cortical networks: noisy excitatory and inhibitory populations interacting in a recurrent but stable way despite expansive single-neuron nonlinearities. This stochastic stabilized supralinear network (SSN) reproduced key aspects of variability in the cortex. During spontaneous activity, i.e., for weak external inputs, model neurons showed large and relatively slow synchronous fluctuations in their membrane potentials. These fluctuations were considerably amplified by the network relative to that expected from the input alone and were quickly quenched and decorrelated by stimuli. The model thus explains and unifies a large body of experimental observations made in diverse systems under various conditions (Churchland et al., 2006, 2010; Finn et al., 2007; Poulet and Petersen, 2008; Gentet et al., 2010; Poulet et al., 2012; Tan et al., 2014; Chen et al., 2014). Moreover, the drop in variability was tuned to specific stimulus features in a model of V1/MT, also capturing recent experimental findings (Ponce-Alvarez et al., 2013; Lin et al., 2015; Lombardo et al., 2015) as well as our own analyses of a previously published dataset (Ecker et al., 2010).

The main insight of our analysis was that in a network of nonlinear neurons with an expansive firing rate nonlinearity, increasing the input increases the effective connection strengths of the network, which in turn modulates the variability of responses. We identified two opposing effects of increasing effective connectivity on variability: the amplification of variability by excitatory-inhibitory interactions (balanced amplification), which dominates at very low (spontaneous) levels of input, and the quenching of variability by increased inhibitory feedback, which dominates for stimulus-driven input. Critically, these network effects preferentially act on smooth patterns of activity that are aligned with the anatomical connectivity of the network, so that it is the shared component of variability that is suppressed and modulated by stimuli. Taken together, we showed that these mechanisms robustly produced experimentally observed spatial and temporal patterns of variability quenching and modulation, whereas the dynamics of the network always remained in the vicinity of a single attractor state, unlike previously proposed mechanisms based on multi-attractor or chaotic dynamical regimes.

Sources and Effects of Stochasticity

We focused on how the network shapes variability and assumed that the variability originates in correlated noise input to the network; such input correlations could arise due to upstream areas already exhibiting noise correlations (e.g., thalamic input to V1, Sadagopan and Ferster, 2012) and/or because of feedforward connectivity implying shared inputs (e.g., Kanitscheider et al., 2015). In contrast, other models have focused on how circuits intrinsically generate slow correlated variability (Litwin-Kumar and Doiron, 2012; Stringer et al., 2016). Nevertheless, our

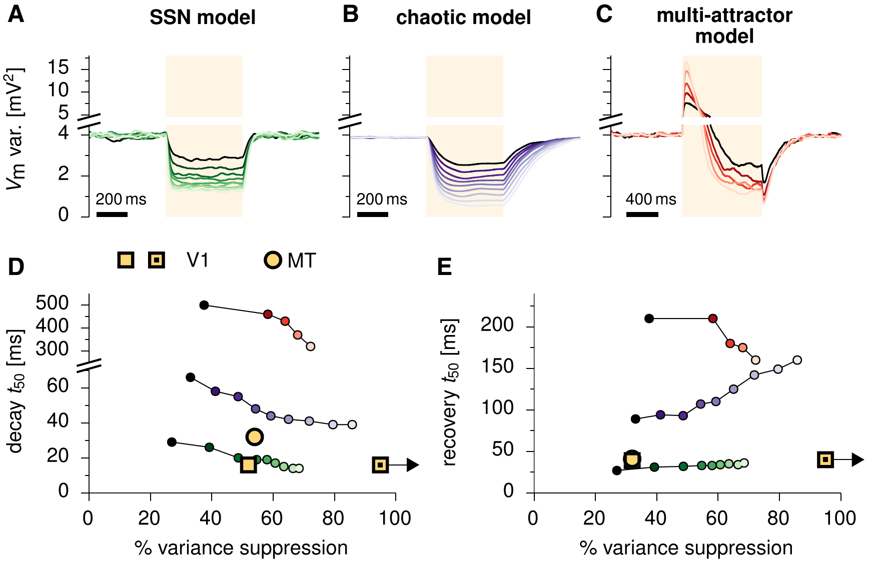

Figure 7. Temporal Dynamics of Variability Modulation in the SSN versus Other Models

(A) Time course of variability reduction and recovery in the ring SSN in response to a step input (shaded area, 500 ms duration) of increasing amplitude (dark to light). Variability is quantified by the population-averaged across-trial V_m variance.

(B) Same as (A), for chaotic network dynamics (Rajan et al., 2010).

(C) Same as (A), for a continuous, multi-attractor network (Ponce-Alvarez et al., 2013). The stimulus is twice as long as in (A) and (B), so that variability suppression can be observed following the characteristic transient increase.

(D) Timescale of variability suppression (time to reach half of the total suppression) as a function of the percentage of variance suppression in the three models, extracted from their corresponding variability trajectories (colors as in A–C).

(E) Same as (D), for recovery timescales (time to recover half of the total suppression). In both (D) and (E), open yellow squares indicate V1 data from anesthetized cat (estimated from Figure S4 in Churchland et al., 2010); yellow circles show data from anesthetized monkey MT (Figure S4 in Churchland et al., 2010); dotted yellow circles show our analysis of the awake monkey V1 data of Ecker et al., 2010. Variability refers to the above-Poisson part of spike count variability (i.e., population-averaged Fano factor minus one), and time constants discard latencies in data. In the data of Ecker et al., 2010, the Fano factor dropped below one, effectively resulting in >100% variance suppression with our definition (right-pointing arrows). All results regarding the SSN and the multi-attractor model shown in this figure were obtained by using the same parameters as in previous figures (Figures 4, 5, and 6).

model also points to an important mechanism for creating shared variability, namely the strong amplification of the input noise by balanced amplification (see also Kriener et al., 2008; Murphy and Miller, 2009; Hennequin et al., 2014).

Although most of our analyses were based on rates, rather than spikes, the effect of fast fluctuations resulting from spiking noise were not ignored, but were incorporated implicitly in the power-law input/output nonlinearity of neurons in the model (Equation 3) and in the stochastic spike-generation mechanism used in our spiking network simulations (Figure 3, STAR Methods). Theoretical work (Miller and Troyer, 2002; Hansel and van Vreeswijk, 2002) shows that these fast fluctuations are the key factor causing momentary firing rates (on the 30–50 ms timescale of V_m fluctuations considered here) to be a supralinear, power-law function of mean voltages, a critical feature of our model. As experiments, as well as our model, show that only the shared but not the private part of variability is modulated by stimuli (Churchland et al., 2010), we expect our assumption that the exponent of the threshold power-law nonlinearity can be considered constant (implying that fast private spiking fluctuations are not affected by stimuli) to be valid to a good approximation. We also expect that a more detailed model explicitly including these fast fluctuations would allow a more systematic study of the effects of stimuli on high-frequency (gamma) oscillations (Ray and Maunsell, 2010), which our current model could only partially account for (Figure 3B).

Tight versus Loose E-I Balance

While we focused on the sources and modulation of slower, correlated fluctuations, a classical model of cortical variability, the "balanced network" (van Vreeswijk and Sompolinsky, 1998), focused on the origin of fast fluctuations from spiking noise. In that model, very large external and recurrent inputs cancel or "balance" to yield a much smaller net input. This mechanism can self-consistently generate the voltage variability to generate irregular spiking. However, the very strong, very fast inhibitory feedback in the balanced network suppresses correlated rate fluctuations away from the stable state (van Vreeswijk and Sompolinsky, 1998; Renart et al., 2010; Tetzlaff et al., 2012), leaving only fast, private variability due to irregular spiking (though "breaking balance" can restore correlated variability; Litwin-Kumar and Doiron, 2012; Rosenbaum et al., 2017). Because the shared variability is already eliminated, stimuli cannot modulate that variability.

As opposed to the "tight balance" between excitation and inhibition in the classical balanced network model, the SSN in the stimulus-driven regime is "loosely balanced": the same mathematical cancellation of external and recurrent input occurs, but in a regime in which inputs are not large and the net input after cancellation is comparable in size to the factors that cancel (Ahmadian et al., 2013). This regime is supported by observations that external input is comparable to, rather than very much larger than, the net input received by cortical cells (Ferster et al., 1996; Chung and Ferster, 1998; Lien and Scanziani, 2013; Li et al., 2013). This loose balance allows correlated variability to persist and be modulated by stimuli. Variability quenching in the stochastic SSN robustly occurred as the input pushed the dynamics to stronger and stronger inhibitory dominance. Consistent with this, with increasing strength of external input, the ratio of inhibition to recurrent excitation received by neurons in the network increases (Rubin et al., 2015), as observed in layers 2/3 of mouse S1 (Shao et al., 2013) and V1 (Adesnik, 2017). In the balanced

network, the ratio of inhibitory to excitatory activity would be fixed regardless of the strength of activation. The balanced network also only yields responses that are linear functions of the input (though see Mongillo et al., 2012), whereas the loosely balanced regime replicates many nonlinear cortical response properties (Rubin et al., 2015), including the profound dependence of correlated variability on stimuli. Although our model does not focus on the origins of fast spiking variability, spiking models in the loosely balanced SSN regime can, given noisy inputs (e.g., Sadagopan and Ferster, 2012), yield the irregular spiking characteristic of cortex (unpublished data).

Further Factors Modulating Variability

We analyzed variability modulation solely as arising from intrinsic network interactions, but other factors may also contribute (Doiron et al., 2016). External inputs may be modulated; for example, the drop with contrast in Fano factors in the lateral geniculate nucleus (LGN) has been argued to underlie V_m variability decreases in V1 simple cells (Sadagopan and Ferster, 2012; but see Malina et al., 2016). However, since high-contrast stimuli also cause firing rates to increase in LGN, the total variance of LGN-to-V1 inputs (scaling with the product of the LGN Fano factor and mean rate) is modulated far less by contrast. This provides some justification for our model choice that input variance did not scale with contrast. Changes in input correlations have also been suggested as a potential mechanism underlying variability modulation (Bujan et al., 2015). However, the proposed mechanism would require a stimulus to specifically increase the correlations of the different inputs onto individual cells (and this increase should be tuned to the stimulus) while leaving the correlation of inputs between cells unchanged. This seems difficult to achieve in cortex, where nearby cells are likely to share a significant amount of input and correlations are generally observed to decrease, rather than increase, with stimulus strength (Churchland et al., 2010).

One particular form of external input modulation, that involving changes in brain state, has been proposed to directly contribute to correlated variability in both awake (Poulet and Petersen, 2008; Ecker et al., 2016) and anesthetized cortex (Ecker et al., 2014; Goris et al., 2014; Lin et al., 2015; Mochol et al., 2015), so that a reduction of state switching would underlie the reduction of shared variability (Mochol et al., 2015; Ecker et al., 2016). To the extent that correlated noise in the input to our model is aligned with a uniform activity pattern, this input can also be regarded as having a single scalar "brain state"-like component that is changing in time. However, our analysis suggests that the variability of this component needs not be modulated directly by the stimulus to account for variability quenching in network responses. Instead, our network used its intrinsic mechanisms to quench variability in response to a stimulus. Importantly, these intrinsic mechanisms not only quenched this uniform component of variability (Figure S8), but also produced more complex patterns of variability modulation via "bump" kinetics that a single brain state-dependent mechanism could not account for.

Cellular factors may also modulate variability. For example, inhibitory reversal potential or spike threshold may set boundaries limiting voltage fluctuations, which would more strongly limit voltage fluctuations in more hyperpolarized or more depolarized states, respectively; conductance increases will reduce voltage fluctuations; and dendritic spikes may contribute more to voltage fluctuations in some states than others (Stuart and Spruston, 2015). A joint treatment of external input, cellular, and recurrent effects may be needed to explain, for example, why V_m variability appears strongest near the preferred stimulus in anaesthetized cat V1 (Finn et al., 2007) or why overall V_m variability grows with visual stimulation in some neurons of awake macaque V1 (Tan et al., 2014).

Cellular properties may themselves be subject to change over time, thereby causing changes in variability. For example, various mechanisms (e.g., attention, intrinsic and synaptic plasticity, neuromodulators, anesthetics) can change the input/output gain of single neurons and the synaptic efficacies of the network. As all these changes eventually lead to changes in effective connectivity, our work offers a principled approach to study their effects on variability and is thus complementary to previous studies that focused on the consequences of different anatomical connectivity patterns on correlations (Kriener et al., 2008; Tetzlaff et al., 2012; Ostojic, 2014; Hennequin et al., 2014).

Effects of Normalization on Variability

The nonlinear response properties of our network were crucial for the modulation of variability by stimuli. These nonlinearities had been shown to capture ubiquitous phenomena involving nonlinear response summation to multiple stimuli, including normalization, surround suppression, and their dependencies on stimulus contrast (Rubin et al., 2015; Ahmadian et al., 2013). As such, the SSN reproduces much of the phenomenology of the "normalization model" of cortical responses (Carandini and Heeger, 2011) and provides a circuit substrate for it.

However, while response normalization has previously been studied for deterministic steady-state responses, our results can be interpreted as showing that it also plays a role in the suppression of ongoing variability by stimuli, as well as shaping the structure of stimulus-evoked noise correlations. Specifically, in the deterministic SSN, steady-state responses to multiple stimuli add sublinearly, and as one stimulus becomes stronger than another, the response to their simultaneous presentation becomes "winner take all," i.e., dominated by the response to the stronger stimulus alone (Rubin et al., 2015). This provides an alternative conceptual explanation of why, in the stochastic SSN, a stronger mean input drive relative to the noise input leads to greater suppression of the noise's contribution to the total network response, thus quenching variability.

Our results go beyond what could be predicted based on this simple qualitative link between steady-state normalization and variability quenching. First, we found a specific quantitative form of normalization in our network: an approximate conservation of the integrated activity across a bump of activity that forms around cells tuned to the stimulus orientation, despite fluctuations in its width. In turn, this predicted a specific pattern of noise correlations that we found contributed substantially to noise variability in V1 of the awake monkey (Figure 6). Second, we were able to study the dynamics with which variability was suppressed following stimulus onset and recovered following stimulus offset and found a good match to experimental data (Figure 7).

The Origin and Role of Inhibitory Dominance

We found that an increase in inhibitory dominance was necessary for the suppression of variability and correlations in the SSN. In line with that, Stringer et al. (2016) studied rodent A1 and V1 in various awake and anesthetized brain states and found that desynchronized states with weaker correlations were accompanied by enhanced activity of putative fast-spiking inhibitory neurons. By fitting a recurrent spiking E-I network model to the data, they found that enhanced inhibitory feedback was the key factor capturing the suppression of correlations. However, the enhanced dominance of inhibition with increasing network activation, which suppresses correlations, was artificially incorporated into the model by making the inhibitory conductance an exponential function of the inhibitory spike count. In contrast, our model provides a dynamical mechanism by which inhibition becomes increasingly dominant with increasing network activation.

Kanashiro et al. (2017) proposed a mechanism similar to ours for the top-down suppression of correlated variability by attention, rather than bottom-up suppression by a stimulus. They also proposed that this arises from enhanced inhibitory feedback resulting from increased effective connectivity due to expansive input/output functions. However, their conclusions differed significantly from ours. They found that, for attention to suppress variability, attentional input had to be directed dominantly to inhibitory cells, while for attention to increase the gain of response to stimuli, stimuli had to give input dominantly to excitatory cells. Note that this implies that stimuli would not suppress variability. We have found that neither of these conditions are necessary (Methods S4) and that stimuli robustly suppress variability. In particular, increasing input strength decreased variability across a wide range of relative strengths of input to excitatory versus inhibitory cells (Figure S2). The main reason for these differences in conclusions is the special, non-generic parametrization of the model studied by Kanashiro et al. (2017) in which a neuron's projections to excitatory and to inhibitory neurons were statistically identical, which precluded the SSN regime (Methods S2).

The Dynamical Regime of Cortical Activity

Two proposals have been made previously to explain quenching of variability by a stimulus: a stimulus may quench multi-attractor dynamics to create single-attractor dynamics (Blumenfeld et al., 2006; Litwin-Kumar and Doiron, 2012; Deco and Hugues, 2012; Ponce-Alvarez et al., 2013; Doiron and Litwin-Kumar, 2014; Mochol et al., 2015), and a stimulus may quench chaotic dynamics to produce non-chaotic dynamics (Molgedey et al., 1992; Bertschinger and Natschläger, 2004; Sussillo and Abbott, 2009; Rajan et al., 2010; Laje and Buonomano, 2013). Our results propose a very different dynamical regime underlying variability quenching, which can be distinguished from the multi-attractor or chaos-suppression models.

Conceptually, the stochastic SSN differs from previous models of stimulus-driven quenching of shared variability in exhibiting a single stable state in all conditions—spontaneous, weakly driven, strongly driven—whereas the others show this only when strongly driven. Furthermore, quenching of variability and correlations in the SSN is highly robust, arising from two basic properties of cortical circuits: inhibitory stabilization of strong excitatory feedback (Tsodyks et al., 1997; Ozeki et al., 2009) and supralinear input/output functions in single neurons (Priebe and Ferster, 2008). In contrast, models of multi-attractor or chaotic dynamics can either account only for the modulation of average pairwise correlations (Mochol et al., 2015) or else require considerable fine tuning of connections (Litwin-Kumar and Doiron, 2012; Ponce-Alvarez et al., 2013) to account for more detailed correlation patterns. Moreover, as studied thus far (Rajan et al., 2010; Ponce-Alvarez et al., 2013; Mochol et al., 2015; but see Harish and Hansel, 2015; Kadmon and Sompolinsky, 2015; Mastrogiuseppe and Ostojic, 2017), they typically ignore Dale's law (the separation of E and I neurons) and its consequences for variability, e.g., balanced amplification. These differences between the SSN and previous models also lead to two main experimentally testable features that we used to distinguish their respective dynamical regimes: the tuning and the timing of variability modulation.

With respect to the stimulus tuning of spike count Fano factors and noise correlations, we found that multi-attractor networks could only predict an M-shaped modulation while the SSN could produce either M- or U-shaped modulations depending on the tuning width of inputs relative to that of connectivity. Indeed, while most types of stimuli in MT were found to result in an M-shaped modulation (Ponce-Alvarez et al., 2013), coherent plaids (Ponce-Alvarez et al., 2013) and random moving dots (Lombardo et al., 2015) in the macaque as well as moving dot fields and drifting gratings in the marmoset (Selina Solomon, personal communication; Solomon et al., 2015) result in a pronounced U-shaped modulation of Fano factors in MT, and our own analyses of V1 data also revealed a U-shaped modulation. Interestingly, our results also suggested that irrespective of the precise shape of the modulation of spike count statistics, membrane potential variability in the SSN should always exhibit a U-shaped profile (Figure 4), which could be tested in future experiments. Critically, we also identified a rarely analyzed aspect of spatial correlation patterns that could most clearly distinguish between different models: the modulation of correlations between orthogonally tuned cells. The SSN predicted only very weak modulation for such cell pairs, while multi-attractor dynamics resulted in modulations that were as deep as for pairs of similarly tuned cells. We found that data from awake macaque V1 supported the SSN.

Another distinctive feature of the SSN regime is the speed of its dynamics, and in particular the speed with which variability is modulated as the stimulus is changed. In contrast to multi-attractor and chaotic dynamics, in which variability modulation happens on timescales that are considerably slower than the single neuron time constant, the SSN produces fast variability modulation on a timescale comparable to the neural time constant. The timescales of variability modulation we extracted from data recorded in monkey visual cortical areas (Churchland et al., 2010; Ecker et al., 2010) were fast, on the order of 20–50 ms, providing further support to the SSN.

In summary, the SSN robustly captures multiple aspects of stimulus modulation of correlated variability and suggests a dynamical regime that uniquely captures a wide array of behaviors of sensory cortex. In turn, our work suggests a principled approach to use data on cortical variability to identify the dynamical regime in which the cortex operates.

STAR★METHODS

Detailed methods are provided in the online version of this paper and include the following:

- KEY RESOURCES TABLE
- CONTACT FOR REAGENT AND RESOURCE SHARING
- METHOD DETAILS
  - SSN model
  - Spiking SSN model
  - Multi-attractor model
  - Chaos suppression model
- QUANTIFICATION AND STATISTICAL ANALYSIS
  - Dataset
  - Factor analysis
- DATA AND SOFTWARE AVAILABILITY

SUPPLEMENTAL INFORMATION

Supplemental Information includes 12 figures and additional methods and can be found with this article online at https://doi.org/10.1016/j.neuron.2018.04.017.

ACKNOWLEDGMENTS

This work was supported by NIH grant R01-EY11001 (K.D.M.); NSF award DBI-1707398 (K.D.M.); the Gatsby Charitable Foundation (K.D.M.); Medical Scientist Training Program grant 5 T32 GM007367-36 (D.B.R.); the Swartz Program in Computational Neuroscience at Columbia University (Y.A.); the Post-doc Program of École des Neurosciences, Paris, France (Y.A.); Swiss National Science Foundation Advanced Postdoctoral Fellowship P300P3.154636 (G.H.); Wellcome Trust Investigator Award 095621/Z/11/Z (M.L., G.H.); and Wellcome Trust Seed Award 202111/Z/16/Z (G.H.). We are grateful to A. Ecker, P. Berens, M. Bethge, and A. Tolias for making their data publicly available. We thank L. Abbott, A. Tan, S. Solomon, A. Renart, and P. Latham for helpful discussions. Y.A. would like to thank D. Hansel and the Centre de Neurophysique, Physiologie, et Pathologie, Paris, for their hospitality.

AUTHOR CONTRIBUTIONS

G.H. performed the model simulations and data analysis and made the figures. G.H., Y.A., D.B.R., M.L., and K.D.M. conceived the study. G.H., Y.A., M.L., and K.D.M. performed mathematical analysis, discussed and planned approaches throughout the research, and wrote the manuscript.

DECLARATION OF INTERESTS

The authors declare no competing interests.

Received: August 2, 2016
Revised: February 14, 2018
Accepted: April 12, 2018
Published: May 16, 2018

REFERENCES

Adesnik, H. (2017). Synaptic Mechanisms of Feature Coding in the Visual Cortex of Awake Mice. Neuron *95*, 1147–1159.e4.

Ahmadian, Y., Rubin, D.B., and Miller, K.D. (2013). Analysis of the stabilized supralinear network. Neural Comput. *25*, 1994–2037.

Amit, D.J., and Brunel, N. (1997). Dynamics of a recurrent network of spiking neurons before and following learning. Network: Computation in Neural Systems *8*, 373–404.

Azouz, R., and Gray, C.M. (1999). Cellular mechanisms contributing to response variability of cortical neurons in vivo. J. Neurosci. *19*, 2209–2223.

Ben-Yishai, R., Bar-Or, R.L., and Sompolinsky, H. (1995). Theory of orientation tuning in visual cortex. Proc. Natl. Acad. Sci. USA *92*, 3844–3848.

Berkes, P., Orbán, G., Lengyel, M., and Fiser, J. (2011). Spontaneous cortical activity reveals hallmarks of an optimal internal model of the environment. Science *331*, 83–87.

Bertschinger, N., and Natschläger, T. (2004). Real-time computation at the edge of chaos in recurrent neural networks. Neural Comput. *16*, 1413–1436.

Blumenfeld, B., Bibitchkov, D., and Tsodyks, M. (2006). Neural network model of the primary visual cortex: from functional architecture to lateral connectivity and back. J. Comput. Neurosci. *20*, 219–241.

Bujan, A.F., Aertsen, A., and Kumar, A. (2015). Role of input correlations in shaping the variability and noise correlations of evoked activity in the neocortex. J. Neurosci. *35*, 8611–8625.

Burak, Y., and Fiete, I.R. (2012). Fundamental limits on persistent activity in networks of noisy neurons. Proc. Natl. Acad. Sci. USA *109*, 17645–17650.

Carandini, M., and Heeger, D.J. (2011). Normalization as a canonical neural computation. Nat. Rev. Neurosci. *13*, 51–62.

Cardin, J.A., Palmer, L.A., and Contreras, D. (2008). Cellular mechanisms underlying stimulus-dependent gain modulation in primary visual cortex neurons in vivo. Neuron *59*, 150–160.

Chen, M., Wei, L., and Liu, Y. (2014). Motor preparation attenuates neural variability and beta-band LFP in parietal cortex. Sci. Rep. *4*, 6809.

Chung, S., and Ferster, D. (1998). Strength and orientation tuning of the thalamic input to simple cells revealed by electrically evoked cortical suppression. Neuron *20*, 1177–1189.

Churchland, M.M., Afshar, A., and Shenoy, K.V. (2006). A central source of movement variability. Neuron *52*, 1085–1096.

Churchland, M.M., Yu, B.M., Cunningham, J.P., Sugrue, L.P., Cohen, M.R., Corrado, G.S., Newsome, W.T., Clark, A.M., Hosseini, P., Scott, B.B., et al. (2010). Stimulus onset quenches neural variability: a widespread cortical phenomenon. Nat. Neurosci. *13*, 369–378.

Cohen, M.R., and Maunsell, J.H.R. (2009). Attention improves performance primarily by reducing interneuronal correlations. Nat. Neurosci. *12*, 1594–1600.

Cunningham, J.P., and Ghahramani, Z. (2015). Linear dimensionality reduction: survey, insights, and generalizations. Journal of Machine Learning Research *16*, 2859–2900.

Dayan, P., and Abbott, L.F. (2001). Theoretical neuroscience (Cambridge, MA: MIT Press).

Deco, G., and Hugues, E. (2012). Neural network mechanisms underlying stimulus driven variability reduction. PLoS Comput. Biol. *8*, e1002395.

Doiron, B., and Litwin-Kumar, A. (2014). Balanced neural architecture and the idling brain. Front. Comput. Neurosci. *8*, 56.

Doiron, B., Litwin-Kumar, A., Rosenbaum, R., Ocker, G.K., and Josić, K. (2016). The mechanics of state-dependent neural correlations. Nat. Neurosci. *19*, 383–393.

Ecker, A.S., Berens, P., Keliris, G.A., Bethge, M., Logothetis, N.K., and Tolias, A.S. (2010). Decorrelated neuronal firing in cortical microcircuits. Science *327*, 584–587.

Ecker, A.S., Berens, P., Cotton, R.J., Subramaniyan, M., Denfield, G.H., Cadwell, C.R., Smirnakis, S.M., Bethge, M., and Tolias, A.S. (2014). State dependence of noise correlations in macaque primary visual cortex. Neuron *82*, 235–248.

Ecker, A.S., Denfield, G.H., Bethge, M., and Tolias, A.S. (2016). On the structure of neuronal population activity under fluctuations in attentional state. J. Neurosci. *36*, 1775–1789.

Ferster, D., Chung, S., and Wheat, H. (1996). Orientation selectivity of thalamic input to simple cells of cat visual cortex. Nature *380*, 249–252.

Finn, I.M., Priebe, N.J., and Ferster, D. (2007). The emergence of contrast-invariant orientation tuning in simple cells of cat visual cortex. Neuron *54*, 137–152.

Gentet, L.J., Avermann, M., Matyas, F., Staiger, J.F., and Petersen, C.C.H. (2010). Membrane potential dynamics of GABAergic neurons in the barrel cortex of behaving mice. Neuron *65*, 422–435.

Goldberg, J.A., Rokni, U., and Sompolinsky, H. (2004). Patterns of ongoing activity and the functional architecture of the primary visual cortex. Neuron *42*, 489–500.

Goris, R.L.T., Movshon, J.A., and Simoncelli, E.P. (2014). Partitioning neuronal variability. Nat. Neurosci. *17*, 858–865.

Guo, Z.V., Inagaki, H.K., Daie, K., Druckmann, S., Gerfen, C.R., and Svoboda, K. (2017). Maintenance of persistent activity in a frontal thalamocortical loop. Nature *545*, 181–186.

Hansel, D., and van Vreeswijk, C. (2002). How noise contributes to contrast invariance of orientation tuning in cat visual cortex. J. Neurosci. *22*, 5118–5128.

Harish, O., and Hansel, D. (2015). Asynchronous rate chaos in spiking neuronal circuits. PLoS Comput. Biol. *11*, e1004266.

Hennequin, G., and Lengyel, M. (2016). Characterizing variability in nonlinear recurrent neuronal networks. arXiv, arXiv:1610.03110, https://arxiv.org/abs/1610.03110.

Hennequin, G., Vogels, T.P., and Gerstner, W. (2014). Optimal control of transient dynamics in balanced networks supports generation of complex movements. Neuron *82*, 1394–1406.

Kadmon, J., and Sompolinsky, H. (2015). Transition to chaos in random neuronal networks. Phys. Rev. X *5*, 041030.

Kanashiro, T., Ocker, G.K., Cohen, M.R., and Doiron, B. (2017). Attentional modulation of neuronal variability in circuit models of cortex. eLife *6*, e23978.

Kanitscheider, I., Coen-Cagli, R., and Pouget, A. (2015). Origin of information-limiting noise correlations. Proc. Natl. Acad. Sci. USA *112*, E6973–E6982.

Kohn, A., and Smith, M.A. (2005). Stimulus dependence of neuronal correlation in primary visual cortex of the macaque. J. Neurosci. *25*, 3661–3673.

Kriener, B., Tetzlaff, T., Aertsen, A., Diesmann, M., and Rotter, S. (2008). Correlations and population dynamics in cortical networks. Neural Comput. *20*, 2185–2226.

Laje, R., and Buonomano, D.V. (2013). Robust timing and motor patterns by taming chaos in recurrent neural networks. Nat. Neurosci. *16*, 925–933.

Li, Y.T., Ibrahim, L.A., Liu, B.H., Zhang, L.I., and Tao, H.W. (2013). Linear transformation of thalamocortical input by intracortical excitation. Nat. Neurosci. *16*, 1324–1330.

Lien, A.D., and Scanziani, M. (2013). Tuned thalamic excitation is amplified by visual cortical circuits. Nat. Neurosci. *16*, 1315–1323.

Lin, I.-C., Okun, M., Carandini, M., and Harris, K.D. (2015). The nature of shared cortical variability. Neuron *87*, 644–656.

Litwin-Kumar, A., and Doiron, B. (2012). Slow dynamics and high variability in balanced cortical networks with clustered connections. Nat. Neurosci. *15*, 1498–1505.

Lombardo, J., Macellaio, M., Liu, B., Osborne, L.C., and Palmer, S.E. (2015). Direction tuning of response variability in populations of MT neurons is different in awake versus anesthetized recordings. In 2015 Neuroscience Meeting Planner (online) (Washington, DC: Society for Neuroscience).

Malina, K.C.-K., Mohar, B., Rappaport, A.N., and Lampl, I. (2016). Local and thalamic origins of ongoing and sensory evoked cortical correlations. bioRxiv, 058727.

Mastrogiuseppe, F., and Ostojic, S. (2017). Intrinsically-generated fluctuating activity in excitatory-inhibitory networks. PLoS Comput. Biol. *13*, e1005498.

Mazzoni, A., Lindén, H., Cuntz, H., Lansner, A., Panzeri, S., and Einevoll, G.T. (2015). Computing the local field potential (LFP) from integrate-and-fire network models. PLoS Comput. Biol. *11*, e1004584.

Miller, K.D., and Fumarola, F. (2012). Mathematical equivalence of two common forms of firing rate models of neural networks. Neural Comput. *24*, 25–31.

Miller, K.D., and Troyer, T.W. (2002). Neural noise can explain expansive, power-law nonlinearities in neural response functions. J. Neurophysiol. *87*, 653–659.

Mitchell, J.F., Sundberg, K.A., and Reynolds, J.H. (2009). Spatial attention decorrelates intrinsic activity fluctuations in macaque area V4. Neuron *63*, 879–888.

Mochol, G., Hermoso-Mendizabal, A., Sakata, S., Harris, K.D., and de la Rocha, J. (2015). Stochastic transitions into silence cause noise correlations in cortical circuits. Proc. Natl. Acad. Sci. USA *112*, 3529–3534.

Molgedey, L., Schuchhardt, J., and Schuster, H.G. (1992). Suppressing chaos in neural networks by noise. Phys. Rev. Lett. *69*, 3717–3719.

Mongillo, G., Hansel, D., and van Vreeswijk, C. (2012). Bistability and spatiotemporal irregularity in neuronal networks with nonlinear synaptic transmission. Phys. Rev. Lett. *108*, 158101.

Moreno-Bote, R., Beck, J., Kanitscheider, I., Pitkow, X., Latham, P., and Pouget, A. (2014). Information-limiting correlations. Nat. Neurosci. *17*, 1410–1417.

Morrison, A., Mehring, C., Geisel, T., Aertsen, A.D., and Diesmann, M. (2005). Advancing the boundaries of high-connectivity network simulation with distributed computing. Neural Comput. *17*, 1776–1801.

Murphy, B.K., and Miller, K.D. (2009). Balanced amplification: a new mechanism of selective amplification of neural activity patterns. Neuron *61*, 635–648.

Murray, J.D., Bernacchia, A., Freedman, D.J., Romo, R., Wallis, J.D., Cai, X., Padoa-Schioppa, C., Pasternak, T., Seo, H., Lee, D., and Wang, X.-J. (2014). A hierarchy of intrinsic timescales across primate cortex. Nat. Neurosci. *17*, 1661–1663.

Orbán, G., Berkes, P., Fiser, J., and Lengyel, M. (2016). Neural variability and sampling-based probabilistic representations in the visual cortex. Neuron *92*, 530–543.

Ostojic, S. (2014). Two types of asynchronous activity in networks of excitatory and inhibitory spiking neurons. Nat. Neurosci. *17*, 594–600.

Ozeki, H., Finn, I.M., Schaffer, E.S., Miller, K.D., and Ferster, D. (2009). Inhibitory stabilization of the cortical network underlies visual surround suppression. Neuron *62*, 578–592.

Ponce-Alvarez, A., Thiele, A., Albright, T.D., Stoner, G.R., and Deco, G. (2013). Stimulus-dependent variability and noise correlations in cortical MT neurons. Proc. Natl. Acad. Sci. USA *110*, 13162–13167.

Poulet, J.F.A., and Petersen, C.C.H. (2008). Internal brain state regulates membrane potential synchrony in barrel cortex of behaving mice. Nature *454*, 881–885.

Poulet, J.F.A., Fernandez, L.M.J., Crochet, S., and Petersen, C.C.H. (2012). Thalamic control of cortical states. Nat. Neurosci. *15*, 370–372.

Priebe, N.J., and Ferster, D. (2008). Inhibition, spike threshold, and stimulus selectivity in primary visual cortex. Neuron *57*, 482–497.

Rajan, K., Abbott, L.F., and Sompolinsky, H. (2010). Stimulus-dependent suppression of chaos in recurrent neural networks. Phys. Rev. E Stat. Nonlin. Soft Matter Phys. *82*, 011903.

Ray, S., and Maunsell, J.H.R. (2010). Differences in gamma frequencies across visual cortex restrict their possible use in computation. Neuron *67*, 885–896.

Reinhold, K., Lien, A.D., and Scanziani, M. (2015). Distinct recurrent versus afferent dynamics in cortical visual processing. Nat. Neurosci. *18*, 1789–1797.

Renart, A., and Machens, C.K. (2014). Variability in neural activity and behavior. Curr. Opin. Neurobiol. *25*, 211–220.

Renart, A., de la Rocha, J., Bartho, P., Hollender, L., Parga, N., Reyes, A., and Harris, K.D. (2010). The asynchronous state in cortical circuits. Science *327*, 587–590.

Rosenbaum, R., Smith, M.A., Kohn, A., Rubin, J.E., and Doiron, B. (2017). The spatial structure of correlated neuronal variability. Nat. Neurosci. *20*, 107–114.

Rubin, D.B., Van Hooser, S.D., and Miller, K.D. (2015). The stabilized supralinear network: a unifying circuit motif underlying multi-input integration in sensory cortex. Neuron *85*, 402–417.

Sadagopan, S., and Ferster, D. (2012). Feedforward origins of response variability underlying contrast invariant orientation tuning in cat visual cortex. Neuron *74*, 911–923.

Shao, Y.R., Isett, B.R., Miyashita, T., Chung, J., Pourzia, O., Gasperini, R.J., and Feldman, D.E. (2013). Plasticity of recurrent l2/3 inhibition and gamma oscillations by whisker experience. Neuron *80*, 210–222.

Softky, W.R., and Koch, C. (1993). The highly irregular firing of cortical cells is inconsistent with temporal integration of random EPSPs. J. Neurosci. *13*, 334–350.

Sompolinsky, H., Crisanti, A., and Sommers, H.J. (1988). Chaos in random neural networks. Phys. Rev. Lett. *61*, 259–262.

Solomon, S.S., Chen, S.C., Morley, J.W., and Solomon, S.G. (2015). Local and global correlations between neurons in the middle temporal area of primate visual cortex. Cereb. Cortex *25*, 3182–3196.

Stringer, C., Pachitariu, M., Steinmetz, N.A., Okun, M., Bartho, P., Harris, K.D., Sahani, M., and Lesica, N.A. (2016). Inhibitory control of correlated intrinsic variability in cortical networks. eLife *5*, e19695.

Stuart, G.J., and Spruston, N. (2015). Dendritic integration: 60 years of progress. Nat. Neurosci. *18*, 1713–1721.

Sussillo, D., and Abbott, L.F. (2009). Generating coherent patterns of activity from chaotic neural networks. Neuron *63*, 544–557.

Tan, A.Y.Y., Chen, Y., Scholl, B., Seidemann, E., and Priebe, N.J. (2014). Sensory stimulation shifts visual cortex from synchronous to asynchronous states. Nature *509*, 226–229.

Tetzlaff, T., Helias, M., Einevoll, G.T., and Diesmann, M. (2012). Decorrelation of neural-network activity by inhibitory feedback. PLoS Comput. Biol. *8*, e1002596.

Troyer, T.W., and Miller, K.D. (1997). Physiological gain leads to high ISI variability in a simple model of a cortical regular spiking cell. Neural Comput. *9*, 971–983.

Tsodyks, M.V., Skaggs, W.E., Sejnowski, T.J., and McNaughton, B.L. (1997). Paradoxical effects of external modulation of inhibitory interneurons. J. Neurosci. *17*, 4382–4388.

van Vreeswijk, C., and Sompolinsky, H. (1998). Chaotic balanced state in a model of cortical circuits. Neural Comput. *10*, 1321–1371.

Vogels, T.P., Rajan, K., and Abbott, L.F. (2005). Neural network dynamics. Annu. Rev. Neurosci. *28*, 357–376.

CellPress

STAR★METHODS

KEY RESOURCES TABLE

REAGENT or RESOURCE	SOURCE	IDENTIFIER
Deposited Data		
Awake monkey V1 dataset	Ecker et al., 2010	http://bethgelab.org/datasets/v1gratings
Software and Algorithms		
OCaml (for all simulations)	Open source	http://www.ocaml.org
Sqlite3 (for V1 data analysis)	Sqlite Consortium	https://sqlite.org/index.html
Mathematica	Wolfram	https://www.wolfram.com/mathematica
Gnuplot	Open source	http://www.gnuplot.info

CONTACT FOR REAGENT AND RESOURCE SHARING

As Lead Contact, Guillaume Hennequin is responsible for all reagent and resource requests. Please contact Guillaume Hennequin at g.hennequin@eng.cam.ac.uk with requests and inquiries.

METHOD DETAILS

The values of all the parameters mentioned below are listed in the tables below. All differential equations detailed below were integrated using a simple Euler scheme with time step 0.1 ms.

Parameters Used in the SSN Simulations

Symbol	Figure 2	Figure 3	Figures 4, 5, 6, and 7	Unit	Description
N_E	1	4,000	50	–	Number of excitatory units
N_I	1	1,000	50	–	Number of inhibitory units
τ_E	20			ms	Membrane time constant (E neurons)
τ_I	10			ms	Membrane time constant (I neurons)
V_{rest}	-70			mV	Resting membrane potential
V_0	-70			mV	Rectification threshold potential
k	0.3			$mV^{-n} \cdot s^{-1}$	Nonlinearity gain
n	2			–	Nonlinearity exponent
W_{EE}	1.25			$mV \cdot s$	E→E connection weight (or sum thereof)
W_{IE}	1.2			$mV \cdot s$	E→I connection weight (or sum thereof)
W_{EI}	0.65			$mV \cdot s$	I→E connection weight (or sum thereof)
W_{II}	0.5			$mV \cdot s$	I→I connection weight (or sum thereof)
τ_{noise}	50			ms	Noise correlation time constant
$\sigma_{0,E}$	0.2	1		mV	Noise standard deviation (E neurons)
$\sigma_{0,I}$	0.1	0.5		mV	Noise standard deviation (I neurons)
p_E	–	0.1	–	–	Outgoing connection probability (E neurons)
p_I	–	0.4	–	–	Outgoing connection probability (I neurons)
τ_{syn}	–	2	–	ms	Synaptic time constants
Δ	–	0.5	–	ms	Axonal delay
ℓ_{syn}	–		45	deg.	Connectivity length scale
ℓ_{noise}	–		60	deg.	Noise correlation length scale
ℓ_{stim}	–		60	deg.	Stimulus tuning length scale of the input
b	–		2	mV	Input baseline
A_{max}	–		20	mV	Maximum input modulation (100% contrast)
θ_{stim}	–		0	deg.	Stimulus direction

Parameters Used in the Multi-attractor Network Simulations			
Symbol	Figures 6 and 7	Unit	Description
N	100	–	Number of units
τ_m	10	ms	Membrane time constant
k	0.1	mV^{-1}	Nonlinearity gain
g_{max}	100	$ms^{-1} \cdot mV^{-1}$	Maximal firing rate
W	$-40/g_{max}$	mV • s	Average connection weight
W_Δ	$33/g_{max}$	mV • s	Tuning-dependent modulation of connection weight
τ_{noise}	50	ms	Noise correlation time constant
σ_0	0.15	mV	Noise standard deviation
ℓ_{noise}	60	deg.	Noise correlation length scale
ℓ_{stim}	60	deg.	Stimulus tuning length scale of the input
b	2	mV	Input baseline
A	0.1	mV	Depth of input tuning
θ_{stim}	0	deg.	Stimulus direction

Parameters Used in the Chaotic Network Simulations			
Symbol	Figure 7	Unit	Description
N	2,000	–	Number of units
τ_m	10	ms	Membrane time constant
σ_W	2	–	Standard deviation of connection weights

SSN model

Our rate-based networks contained N_E excitatory and N_I inhibitory units, yielding a total $N = N_E + N_I$ units. The circuit dynamics were governed by (see also Methods S1):

$$\tau_i \frac{dV_i}{dt} = -V_i + V_{rest} + h_i(t) + \eta_i(t) + \sum_{j \in \text{E cells}} W_{ij}\, r(V_j) - \sum_{j \in \text{I cells}} W_{ij}\, r(V_j), \quad \text{(Equation 2)}$$

where V_i denotes the V_m of neuron i, τ_i is its membrane time constant, V_{rest} is a resting potential, W_{ij} is the (positive or zero) strength of the synaptic connection from neuron j to neuron i, and $h_i(t)$ is the potentially time-varying but deterministic component (the mean) of external input to which a noise term $\eta_i(t)$ is added (see below, "Input noise"). The momentary firing rate of cell j was given by a threshold-powerlaw function of its membrane potential:

$$r(V_j) = k \lfloor V_j - V_0 \rfloor_+^n. \quad \text{(Equation 3)}$$

Experiments support Equation 3 when both membrane potentials and spike counts are averaged in 30 ms time bins (Priebe and Ferster, 2008). Accordingly, V_i in Equation 2 can be understood as the coarse-grained (low-pass filtered) version of the raw somatic membrane potential; in particular it does not incorporate the action potentials themselves. Thus the effective time resolution of our model was around 30 ms which allowed studying the effects of inputs that did not change significantly on timescales shorter than that. Accordingly, in Equation 2 we assumed that external noise had a time constant $\tau_{noise} = 50$ ms, in line with membrane potential and spike count autocorrelation timescales found across the cortex (Azouz and Gray, 1999; Berkes et al., 2011; Murray et al., 2014).

Equations 2 and 3 together define the stabilized supralinear network model studied in Ahmadian et al. (2013) and Rubin et al. (2015), but formulated with voltages rather than rates as the dynamical variables (the two formulations are mathematically equivalent when all neurons have the same time constant, Miller and Fumarola, 2012) and with the crucial addition of noise that enables us to study variability. In all the figures of the main text, the exponent of the power-law nonlinearity was set to $n = 2$ (but see Figure S2 for n > 2). Methods S2 explores more general scenarios.

Mean external input

In the reduced rate model of Figure 2, each unit received the same constant mean input h. In the ring model, the mean input to neuron i was the sum of two components,

$$h_i(\theta_{stim}) = b + c \cdot A_{max} \cdot \exp\left(\frac{\cos(\theta_i - \theta_{stim}) - 1}{\ell^2_{stim}}\right). \quad \text{(Equation 4)}$$

The first term $b = 2$ mV is a constant baseline which drives spontaneous activity. The second term models the presence of a stimulus with orientation θ_{stim} in the visual field as a circular-Gaussian input bump of "half width" ℓ_{stim} centered around θ_{stim} and scaled by a factor c (increasing c represents increasing stimulus contrast), taking values from 0 to 1, times a maximum amplitude $A_{\max}$. We assumed for simplicity that E and I cells are driven equally strongly by the stimulus, though this could be relaxed.

Input noise

The input noise term $\eta_i(t)$ in Equation 2 was modeled as a multivariate Ornstein-Uhlenbeck process:

$$\tau_{\text{noise}} d\boldsymbol{\eta} = -\boldsymbol{\eta} dt + \sqrt{2\tau_{\text{noise}} \boldsymbol{\Sigma}^{\text{noise}}}\, d\xi, \quad \text{(Equation 5)}$$

where $d\xi$ is a collection of N independent Wiener processes and $\boldsymbol{\Sigma}^{\text{noise}}$ is an $N \times N$ input covariance matrix (see below). Note that Equation 5 implies $\langle \eta_i(t)\ \eta_j(t+\tau)\rangle_t = \Sigma_{ij}^{\text{noise}} e^{-|\tau|/\tau_{\text{noise}}}$.

In the reduced two-population model (Figure 2), noise terms were chosen to be uncorrelated, i.e., $\Sigma_{ij}^{\text{noise}} = \sigma^2_{\alpha(i)} \delta_{ij}$ (where $\delta_{ij} = 1$ if $i = j$ and 0 otherwise), $\alpha(i) \in \{\text{E}, \text{I}\}$ is the E/I type of neuron i, and σ^2_α is the variance of noise fed to population α (see Equation 7 below). In the spiking two-population model (Figure 3), input noise covariance was uniform, such that $\Sigma_{ij}^{\text{noise}} = \sigma^2_{\text{noise}} [\delta_{ij}\ (1-\rho) + \rho]$, with the pair-wise correlation coefficient set to $\rho = 0.2$ (see Figure S5 for the dependence of our results on ρ). In the ring model (Figures 4, 5, 6, and 7), the noise had spatial structure, with correlations among neurons decreasing with the difference in their preferred directions following a circular-Gaussian:

$$\Sigma_{ij}^{\text{noise}} = \sigma_{\alpha(i)}\ \sigma_{\alpha(j)}\ \exp\left(\frac{\cos(\theta_i - \theta_j) - 1}{\ell^2_{\text{noise}}}\right), \quad \text{(Equation 6)}$$

where θ_i and θ_j are the preferred orientations of neurons i and j (exc. or inh.), and ℓ_{noise} is the correlation length (see table "Parameters Used in the SSN Simulations"). The noise amplitude has the natural scaling

$$\sigma_\alpha = \sigma_{0,\alpha} \sqrt{1 + \frac{\tau_\alpha}{\tau_{\text{noise}}}} \quad (\alpha \in \{\text{E}, \text{I}\}) \quad \text{(Equation 7)}$$

such that, in the absence of recurrent connectivity ($\mathbf{W} = 0$), the input noise alone would drive V_{m} fluctuations of standard deviation $\sigma_{0,E}$ or $\sigma_{0,I}$, measured in mV, in the E or I cells, respectively. We chose values of $\sigma_{0,E}$ that yielded spontaneous Fano factors in the range 1.3-1.5 where appropriate, and chose $\sigma_{0,I} = \sigma_{0,E}/2$ to make up for the difference in membrane time constants between E and I cells (see table "Parameters Used in the SSN Simulations").

Connectivity

The synaptic weight matrix in the reduced model was given by

$$\mathbf{W} = \begin{pmatrix} W_{\text{EE}} & -W_{\text{EI}} \\ W_{\text{IE}} & -W_{\text{II}} \end{pmatrix}, \quad \text{(Equation 8)}$$

where W_{AB} is the magnitude of the connection from the unit of type B (E or I) to that of type A (see table "Parameters Used in the SSN Simulations" for parameter values). In the ring model, connectivity fell off with angular distance on the ring, following a circular-Gaussian profile:

$$W_{ij} \propto \exp\left(\frac{\cos(\theta_i - \theta_j) - 1}{\ell^2_{\text{syn}}}\right), \quad \text{(Equation 9)}$$

where θ_i and θ_j are the preferred orientations of neurons i and j (exc. or inh.), and ℓ_{syn} sets the length scale over which synaptic weights decay (see table "Parameters Used in the SSN Simulations"). The connectivity matrix $\mathbf{W}$ was further rescaled in each row and in each quadrant, such that the sum of incoming E and I weights onto each E and I neuron (4 cases) matched the values of W_{EE}, W_{IE}, $-W_{\text{EI}}$ and $-W_{\text{II}}$ in the reduced model. Thus, all connectivity matrices used in the SSN model obeyed Dale's law.

Simulated spike counts

To relate the firing rate model to spiking data in Figures 4 and 6, we assumed that action potentials were emitted as inhomogeneous (doubly stochastic) Poisson processes with time-varying rate $r(V_{\text{m}})$ given by Equation 3. Unlike in the full spiking model (see below), spikes did not "re-enter" the dynamics of Equation 2, according to which neurons influence each other through their firing rates. Spikes were counted in 100 ms time bins and spike count statistics such as Fano factors and pairwise correlations were computed as standard.

Spiking SSN model

Dynamics

In the spiking model (Figure 3), neuron i emitted spikes stochastically with an instantaneous probability equal to $dt\, r(V_i)$, with time-varying rate $r(V_i)$ given by Equation 3, consistent with how (hypothetical) spikes were modeled in the rate-based case (*cf.* above). Presynaptic spikes were filtered by synaptic dynamics into exponentially decaying postsynaptic currents (E or I):

$$\frac{da_j}{dt} = -\frac{a_j}{\tau_{\text{syn}}} + \sum_{t_j} \delta(t - t_j - \Delta), \qquad \text{(Equation 10)}$$

where the t_j's are the firing times of neuron j, $\tau_{\text{syn}} = 2$ ms is the synaptic time constant, and $\Delta = 0.5$ ms is a small axonal transmission delay (which enabled the distribution of the simulations onto multiple compute cores; Morrison et al., 2005). Synaptic currents then contributed to membrane potential dynamics according to

$$\tau_i \frac{dV_i}{dt} = -V_i + V_{\text{rest}} + h_i(t) + \eta_i(t) + \sum_{j \in \text{E cells}} J_{ij}\, a_j(t) - \sum_{j \in \text{I cells}} J_{ij}\, a_j(t), \qquad \text{(Equation 11)}$$

where the synaptic efficacies J_{ij} are described below, and the noise term η_i was modeled exactly as described above.

Connectivity

For each neuron i, we drew $p_E N_E$ excitatory and $p_I N_I$ inhibitory presynaptic partners, uniformly at random. Connection probabilities were set to $p_E = 0.1$ and $p_I = 0.4$ respectively. The corresponding synaptic weights took on values $J_{ij} = W_{\alpha\beta} / (\tau_{\text{syn}}\, p_\beta\, N_\beta)$ where $\{\alpha, \beta\} \in \{E, I\}$ denote the populations to which neuron i and j belong respectively, and $W_{\alpha\beta}$ are the connections in the reduced model (see table "Parameters Used in the SSN Simulations"). This choice was such that, for a given set of mean firing rates in the E and I populations, average E and I synaptic inputs to E and I cells matched the corresponding recurrent inputs in the rate-based model. Synapses that were not drawn were obviously set to $J_{ij} = 0$.

Local field potential

As a proxy for LFP in Figure 3, we took the momentary population-averaged V_{m} (Mazzoni et al., 2015 simulated various proxies and, although some proxies were more accurate, they found the average V_{m} to be reasonably accurate).

Multi-attractor model

We compared our ring SSN model to a version of the ring attractor model published by Ponce-Alvarez et al. (2013). The ring attractor model had a single population with a similar ring topology, and—using the same notation as above—the connectivity took the form (*cf.* Equation 9)

$$W_{ij} = \overline{W} + \frac{W_\Delta}{N} \cos(\theta_i - \theta_j), \qquad \text{(Equation 12)}$$

where $N = 100$ is the number of neurons, and $\overline{W}$ and W_Δ are two parameters that control the average connection strength and modulation with tuning dissimilarity, respectively. Note that, in general, this connectivity matrix could violate Dale's law but with the specific parameters used here it did not (see table "Parameters Used in the Multi-attractor Network Simulations"). Instead, all connections were inhibitory to keep the system in the marginally stable regime (as in Ponce-Alvarez et al., 2013). The dynamics of the network obeyed a similar stochastic differential equation as for the ring SSN (Equation 2), namely

$$\tau_{\text{m}} \frac{dV_i}{dt} = -V_i + h_i(t) + \eta_i(t) + \sum_j W_{ij}\, r(V_j), \qquad \text{(Equation 13)}$$

with the momentary firing rate of cell j given by a rectified saturating firing rate nonlinearity (*cf.* Equation 3):

$$r(V_j) = g_{\text{max}} \tanh\left(k \lfloor V_j \rfloor_+\right), \qquad \text{(Equation 14)}$$

and a noise process $\boldsymbol{\eta}$ identical to the one we used in the SSN (same spatial and temporal correlations, Equations 5 and 6), with a variance adjusted so as to obtain Fano factors of about 1.5 during spontaneous activity (Figure S9B, black). The external input had both a constant baseline, b, and a contrast-dependent modulated component (*cf.* Equation 4):

$$h_i = b + c \cdot (1 - A + A \cos(\theta_i - \theta_{\text{stim}})), \qquad \text{(Equation 15)}$$

where A controlled the depth of the modulation, and c represents stimulus strength.

Note that although the phenomenology and dynamical regime of this model was consistent with that of Ponce-Alvarez et al. (2013) (Figure S9), the model differed from their original implementation in some of the details: our dynamics were written in voltage form, not in rate form, we had only one unit at each location on the ring (as opposed to small pools of neurons), and our input noise process had spatial correlations to allow for a more direct and consistent comparison with the ring SSN.

Our analysis of variability in this ring attractor network is presented in Figure S9 in a format identical to that of Figure 5, and shows that shared variability is entirely dominated by the fluctuations in the location of an otherwise very stable bump of activity.

Chaos suppression model

We also implemented a chaotic rate network of size $N = 2,000$ with the following (deterministic) dynamics (*cf.* Equations 2 and 13):

$$\tau_m \frac{dV_i}{dt} = -V_i + h_i(t) + \sum_j W_{ij}\, r(V_j), \qquad \text{(Equation 16)}$$

with an (unrectified) saturating firing rate nonlinearity (*cf.* Equations 3 and 14)

$$r(V_j) = \tanh(V_j) \qquad \text{(Equation 17)}$$

(which could thus go negative as well as positive). Elements of the synaptic weight matrix were sampled i.i.d. from a normal distribution (thus violating Dale's law, *cf.* Equations 9 and 12):

$$W_{ij} \sim \mathcal{N}\left(0, \sigma_W^2/N\right), \qquad \text{(Equation 18)}$$

with $\sigma_W = 2$, which placed the network in the chaotic regime (Sompolinsky et al., 1988). The external input was a constant input vector of the form (*cf.* Equations 4 and 15)

$$h_i = c \cdot \cos(\phi_i), \qquad \text{(Equation 19)}$$

where ϕ_i is a phase sampled i.i.d. from a uniform distribution between 0 and 2π, and c represents stimulus strength. See table "Parameters Used in the Chaotic Network Simulations" for all parameter values. As shown in Rajan et al. (2010), chaos is suppressed for large enough c.

QUANTIFICATION AND STATISTICAL ANALYSIS

Dataset

We analyzed neural recordings from the V1 of two awake monkeys (Figures 4, 6, and 7). A full description of the experimental protocol and recordings can be found in the original publication (Ecker et al., 2010). We discarded all cells that were poorly isolated (contamination >5%), leaving us with 330 cells to analyze. The stimuli consisted of moving gratings of various orientations, all at 100% contrast. We fitted orientation tuning curves (Figure S11; average firing rate in the first 500 ms following stimulus onset, as a function of stimulus orientation) of the form $f(\theta) \equiv f_0 + f_m \exp[\kappa(\cos(2(\theta - \theta_{pref})) - 1)]$, where θ is the stimulus orientation (thus, we neglected the direction of motion, which could be in either of the two directions orthogonal to the orientation of the grating). The fit was achieved using nonlinear least-squares regression.

For each neuron, we calculated an orientation tuning index (OTI), defined based on the fitted tuning curve as

$$\mathrm{OTI} = \frac{f(\theta_{pref}) - f(\theta_{orth})}{f(\theta_{pref}) + f(\theta_{orth})}, \qquad \text{(Equation 20)}$$

where $\theta_{orth} = \theta_{pref} + \pi/2$. As the ring architecture we studied in Figures 4, 5, 6, and 7 only applied to neurons with well-defined tuning curves, we excluded cells that had OTI < 0.75 as well as average evoked rates (measured during the stimulus period) below 1 spike/sec. This left us with 99 well-tuned cells to analyze.

Our analysis of the stimulus tuning of Fano factors and pairwise spike-count correlations was based on a time window of 100 ms starting at stimulus onset.

Factor analysis

We performed factor analysis of spike counts, either for a single stimulus condition in the model (the model had a natural rotational symmetry), or separately for each stimulus condition (direction) in the V1 dataset, subsequently averaging the reported quantities across conditions. We worked with normalized spike counts, defined as $\tilde{c}_{ik} = c_{ik}/\sqrt{\langle c_{ik}\rangle_k}$ where c_{ik} is the spike count of neuron i in trial k and $\langle \cdot \rangle_k$ denotes averaging across trials. Note that the variances of these normalized spike counts are exactly the Fano factors, i.e., the usual measure of spike count variability. This prevented the normalized spike count covariance matrix $\tilde{\mathbf{C}}$ from being contaminated by a rank-1 pattern of network covariance merely reflecting the tuning of single-neuron firing rates (the "Poisson" part of variability, which tends to scale with the mean count). Factor analysis decomposes $\tilde{\mathbf{C}}$ as the sum of a rank-k covariance matrix $\tilde{\mathbf{C}}_{shared}$ representing k modes of network covariances, and a diagonal matrix $\tilde{\mathbf{C}}_{private}$. In the rate model, we could near-perfectly estimate the spike count covariance matrix, so we performed factor analysis by direct eigendecomposition of $\tilde{\mathbf{C}}$, thus defining $\tilde{\mathbf{C}}_{shared} = \sum_{i=1}^{k} \lambda_i \mathbf{v}_i \mathbf{v}_i^\top$ whereby the top k eigenvectors $\mathbf{v}_1, \ldots, \mathbf{v}_k$ of $\tilde{\mathbf{C}}$ contributed to shared variability in proportion of the corresponding eigenvalues λ_i. For factor analysis of the monkey V1 data, we performed direct maximization of the data likelihood (Cunningham and Ghahramani, 2015), also keeping k factors. In Figure 4, we set $k = 3$, but we observed quenching of shared variability irrespective of k (Figure S12).

DATA AND SOFTWARE AVAILABILITY

The code used for model simulations and data analysis is available from the Lead Contact, Dr Guillaume Hennequin, upon request.

Neuron, Volume *98*

Supplemental Information

The Dynamical Regime of Sensory Cortex: Stable Dynamics around a Single Stimulus-Tuned Attractor Account for Patterns of Noise Variability

Guillaume Hennequin, Yashar Ahmadian, Daniel B. Rubin, Máté Lengyel, and Kenneth D. Miller

The dynamical regime of sensory cortex: stable dynamics around a single stimulus-tuned attractor account for patterns of noise variability — Supplemental Methods —

Guillaume Hennequin, Yashar Ahmadian⋆, Daniel B. Rubin⋆,
Máté Lengyel† and Kenneth D. Miller†

⋆,† Equal contributions

Contents

Methods S1 Model setup **2**

Methods S2 Mean responses in the stabilized supralinear regime **2**

2.1 Input-dependence of mean responses . . . 2
2.2 The behavior of typical networks: numerical simulations . . . 4

Methods S3 Membrane potential variability in the two-population SSN model **5**

3.1 Linearization of the dynamics . . . 5
3.2 General result . . . 6
3.3 Analysis in simplified scenarios . . . 7
3.4 Effects of input correlations . . . 10
3.5 Mechanisms of variability modulation: Schur decomposition . . . 10
3.6 How do shear and restoring flow fields depend on the input? . . . 13

Methods S4 Firing rate and spike count variability **14**

4.1 Generic results in the SSN regime . . . 14
4.2 The specific regime of Kanashiro et al. (2017) . . . 14

Supplemental Figures **18**

Figure S1 . . . 18
Figure S2 . . . 19
Figure S3 . . . 20
Figure S4 . . . 21
Figure S5 . . . 22
Figure S6 . . . 23
Figure S7 . . . 24
Figure S8 . . . 25
Figure S9 . . . 26
Figure S10 . . . 27
Figure S11 . . . 28
Figure S12 . . . 29

Methods S1 Model setup

We consider the stochastic and nonlinear rate model of Equations 2 and 3 of the main text. To simplify notation, we assume $V_{\text{rest}} = 0$ mV without loss of generality as it can be absorbed in the external input, and rewrite:

$$\tau_{\text{E}}\,\mathbf{T}\,\frac{d\mathbf{V}}{dt} = -\mathbf{V}(t) + k\,\mathbf{W}\,\lfloor \mathbf{V}(t) \rfloor_+^n + \mathbf{h}(t) + \boldsymbol{\eta}(t) \tag{S1}$$

with $n > 1$ ($n = 2$ throughout the main text). In Equation S1, $\lfloor \mathbf{x} \rfloor_+^n$ denotes the pointwise application of the threshold power-law nonlinearity to the vector $\mathbf{x}$, that is, $\lfloor \mathbf{x} \rfloor_+^n$ is the vector whose i^{th} element is x_i^n if $x_i > 0$, or 0 otherwise; $\mathbf{T}$ is a diagonal matrix of relative membrane time constants measured in units of τ_{E}; $\mathbf{W}$ is a matrix of synaptic connections, consisting of N_{E} positive columns (corresponding to excitatory presynaptic neurons) and N_{I} negative columns (inhibitory neurons) for a total size of $N = N_{\text{E}} + N_{\text{I}}$; $\mathbf{h}(t)$ is a possibly time-varying but deterministic external input to neuron i; and $\boldsymbol{\eta}$ is a multivariate Ornstein-Uhlenbeck process with separable spatiotemporal correlations given by

$$\langle \boldsymbol{\eta}(t)\,\boldsymbol{\eta}(t+\tau) \rangle_t = e^{-|\tau|/\tau_\eta}\,\boldsymbol{\Sigma}_\eta \tag{S2}$$

where $\boldsymbol{\Sigma}_\eta$ is the covariance matrix of the input noise and τ_η is its correlation time. In particular, we are going to study how τ_η and correlations in $\boldsymbol{\Sigma}_\eta$ affect network variability. We adopt the following notations for relative time constants:

$$\bar{\tau}_{\text{I}} \equiv \frac{\tau_{\text{I}}}{\tau_{\text{E}}} \qquad \text{and} \qquad \bar{\tau}_\eta \equiv \frac{\tau_\eta}{\tau_{\text{E}}} \tag{S3}$$

In general, recurrent processing in the network is prone to instabilities due to the expansive, non-saturating V_{m}-rate relationship in single neurons. However, there are generous portions of parameter space in which inhibition dynamically stabilizes the network. We refer to this case as the "stabilized supralinear network", or SSN (Ahmadian et al., 2013; Rubin et al., 2015).

Methods S2 Mean responses in the stabilized supralinear regime

2.1 Input-dependence of mean responses

Our analysis of the stochastic SSN developed in Methods S3 will show that the modulation of variability relies on the nonlinear behavior of *mean* responses to varying inputs (Figure 2D of the main text), which in turn were studied previously (Ahmadian et al., 2013). We repeat these analyses here for completeness focusing in particular on the transition from superlinear integration of small inputs to sublinear responses to larger inputs. Note that here we have written the circuit dynamics in voltage form (Equation S1), while Ahmadian et al., 2013 chose a slightly different rate form; accordingly, the equations we now derive differ from the original equations in their form, but not in their nature (in fact, steady state solutions studied in Ahmadian et al., 2013 are mathematically equivalent in the two formulations, and moreover when $\mathbf{T}$ is proportional to the identity matrix, dynamic solutions are also exactly equivalent; see Miller and Fumarola, 2012).

As this section is devoted to mean responses, we neglect the input noise $\boldsymbol{\eta}$ for now. We thus write the deterministic dynamics of the mean potentials $\overline{V}_i$ as

$$\tau_{\text{E}}\,\mathbf{T}\,\frac{d\overline{\mathbf{V}}}{dt} = -\overline{\mathbf{V}} + k\,\mathbf{W}\,\lfloor \overline{\mathbf{V}} \rfloor_+^n + h\,\mathbf{g} \tag{S4}$$

and ask how neurons collectively respond to a constant external stimulus h fed to them through a vector $\mathbf{g} \sim \mathcal{O}(1)$ of feedforward weights. After some transient, and assuming the network is stable (see below), the network settles in a steady state $\overline{\mathbf{V}}$ which must obey the following fixed point equation, obtained by setting the l.h.s. of Equation S4 to zero:

$$\overline{\mathbf{V}} = h\,\mathbf{g} + k\,\mathbf{W}\,\lfloor \overline{\mathbf{V}} \rfloor_+^n \tag{S5}$$

As in the main text, we focus on the case of a threshold-quadratic nonlinearity, $n = 2$, though the following derivations can be extended to arbitrary $n > 1$. Following Ahmadian et al. (2013), we begin by defining $\mathbf{J} \equiv \mathbf{W}/\psi$ where $\psi = \|\mathbf{W}\|$ for some matrix norm $\|\cdots\|$, so that the dimensionless vector $\mathbf{J}$ has $\|\mathbf{J}\| = 1$. We also define dimensionless mean voltage and input respectively as

$$\bar{\mathbf{y}} \equiv 2\,k\,\psi\,\bar{\mathbf{V}} \tag{S6}$$

$$\alpha \equiv 2\,k\,\psi\,h \tag{S7}$$

(note that the definition of α differs from that in Ahmadian et al., 2013 by a factor of 2). With these definitions, and $n = 2$, the fixed point equation for the mean potentials, Equation S5, becomes

$$\bar{\mathbf{y}} = \alpha\,\mathbf{g} + \frac{1}{2}\mathbf{J}\,\lfloor\bar{\mathbf{y}}\rfloor_+^2 \tag{S8}$$

Network responses to small inputs When α is small (i.e. h is small, given fixed connectivity strength ψ), it is easy to see that

$$\bar{\mathbf{y}} \approx \alpha\,\mathbf{g} + \mathcal{O}(\alpha^2) \tag{S9}$$

In essence, the fixed point Equation S8 is already the first-order Taylor expansion of $\bar{\mathbf{y}}$ for small α (indeed, the recurrent term $\mathbf{J}\,\lfloor\bar{\mathbf{y}}\rfloor_+^2$ is $\mathcal{O}(\alpha^2)$, self-consistently). Thus, for small input α, membrane potentials scale linearly with α, and firing rates are quadratic in α, merely reflecting the single-neuron nonlinearity. In other words, the network behaves mostly as a relay of its feedforward inputs, with only minor corrections due to recurrent interactions.

More generally, by repeatedly substituting the right side of Equation S8 for $\bar{\mathbf{y}}$ into Equation S8, we arrive at the expansion

$$\bar{\mathbf{y}} = \alpha\,\mathbf{g} + \frac{1}{2}\mathbf{J}\left\lfloor \alpha\,\mathbf{g} + \frac{1}{2}\mathbf{J}\left\lfloor \alpha\,\mathbf{g} + \frac{1}{2}\mathbf{J}\,\lfloor\cdots\rfloor_+^2 \right\rfloor_+^2 \right\rfloor_+^2 \tag{S10}$$

The net result involves a series of terms of order α, α^2, α^4 ..., which can be expected to converge for small α ($\alpha \ll 1$).

Network responses to larger inputs For large α ($\alpha \gg 1$), the expansion of Equation S10 will not converge and so cannot describe responses. Physically this tends to correspond to the excitatory subnetwork becoming unstable by itself. At the level of the fixed point Equation S8, recurrent processing involves squaring $\bar{\mathbf{V}}$, passing it through the recurrent connectivity, adding the feedforward input, squaring the result again, ..., which for large enough input and purely excitatory connectivity would yield activity that grows arbitrarily large. A finite-activity solution is achieved through stabilization by inhibitory feedback. Mathematically, for this to occur, the recurrent term $\mathbf{J}\,\lfloor\bar{\mathbf{y}}\rfloor_+^2$ must cancel the linear dependence of $\bar{\mathbf{y}}$ on α in Equation S8 (since any linear dependence would be squared by the right side of Equation S8, then squared again, ..., to yield an explosive series as in Equation S10). That is, we must have

$$\frac{1}{2}\mathbf{J}\,\lfloor\bar{\mathbf{y}}\rfloor_+^2 = -\alpha\,\mathbf{g} + \mathcal{O}(\sqrt{\alpha}) \tag{S11}$$

such that (again from Equation S8)

$$\bar{\mathbf{y}} \sim \mathcal{O}(\sqrt{\alpha}) \tag{S12}$$

at most. This means that membrane potentials scale at most as $\sqrt{\alpha}$, i.e. firing rates scale at most linearly in α. However, in many cases, firing rates too will be sublinear in α. This is best exemplified in the context of our two-population E/I model, by following Ahmadian et al. (2013) and introducing the notation:

$$\Omega_\mathrm{E} \equiv \left(-\mathbf{J}^{-1}\,\mathbf{g}\right)_\mathrm{E} \mathrm{Det}\,\mathbf{J} = J_\mathrm{II}\,g_\mathrm{E} - J_\mathrm{EI}\,g_\mathrm{I} \tag{S13}$$

$$\Omega_\mathrm{I} \equiv \left(-\mathbf{J}^{-1}\,\mathbf{g}\right)_\mathrm{I} \mathrm{Det}\,\mathbf{J} = J_\mathrm{IE}\,g_\mathrm{E} - J_\mathrm{EE}\,g_\mathrm{I} \tag{S14}$$

(note that we only consider networks in which $\text{Det}\,\mathbf{J} > 0$, as it must for stabilization to occur for all input levels α; Ahmadian et al., 2013). Equation S11 can then be rewritten as

$$\lfloor \bar{\mathbf{y}} \rfloor_+^2 = \frac{2\,\alpha}{\text{Det}\,\mathbf{J}} \begin{pmatrix} \Omega_E \\ \Omega_I \end{pmatrix} + \mathcal{O}(\sqrt{\alpha}) \tag{S15}$$

Now, depending on the choice of parameters (recurrent weights $\mathbf{J}$ and feedforward weights $\mathbf{g}$), Ω_E in particular can be negative. Since $\lfloor \bar{y}_E \rfloor_+^2$ is positive, it must be that the sublinear term $\mathcal{O}(\sqrt{\alpha})$ dominates over the (negative) linear term $2\,\Omega_E\,\alpha/\text{Det}\,\mathbf{J}$, at least over some range of α over which the E firing rate is non-zero. In this case, $\lfloor \bar{y}_E \rfloor_+^2$ behaves roughly as $\sqrt{\alpha}$ over some range[1] before it gets pushed to zero, and accordingly $\bar{y}_E$ must be approximately $\sqrt{\sqrt{\alpha}}$ over the same range, i.e. the E unit responds strongly sublinearly. Ahmadian et al. (2013) referred to this regime of eventual decrease of $\bar{y}_E$ with increasing stimulus strength as "supersaturation", and showed that it occurs for physiologically plausible parameter regimes. Our choice of parameters for the two-population model of the main text falls within this class of strongly sublinear E responses ($\Omega_E < 0$), but we will show in Methods S3 that the SSN displays the same input modulation of variability irrespective of the sign of Ω_E.

In summary, the SSN responds superlinearly to small inputs, and sublinearly to larger inputs. Firing rates become at most linear (but will be sublinear if $\Omega_E < 0$) with large inputs. Accordingly, membrane potentials show a transition from linear to (potentially strongly) sublinear responses to increasing inputs. Moreover, this transition occurs for $\alpha \sim \mathcal{O}(1)$.

2.2 The behavior of typical networks: numerical simulations

In the context of the reduced two-population model of the main text, we now complement the above theoretical arguments with a numerical analysis of the SSN's responses across a wide range of parameters, in order to form a picture of the "typical" behavior of the SSN in physiologically realistic regimes. We will later (Methods S3) reuse these numerical explorations to show that the modulation of variability by external input in the SSN is robust to changes of parameters.

The dynamics of the trial-averaged dimensionless "population voltages" are given by

$$\begin{aligned} \tau_E\,\dot{\bar{y}}_E &= -\bar{y}_E + \frac{1}{2}\left(J_{EE}\,\lfloor \bar{y}_E \rfloor_+^2 - J_{EI}\,\lfloor \bar{y}_I \rfloor_+^2\right) + \alpha\,g_E \\ \tau_I\,\dot{\bar{y}}_I &= -\bar{y}_I + \frac{1}{2}\left(J_{IE}\,\lfloor \bar{y}_E \rfloor_+^2 - J_{II}\,\lfloor \bar{y}_I \rfloor_+^2\right) + \alpha\,g_I \end{aligned} \tag{S16}$$

It is difficult to get good estimates of the values of the 6 free parameters (feedforward weights and recurrent weights) directly from biology. Therefore, our approach is to construct a large number of networks by randomly sampling these parameters within broad intervals, and rejecting those networks that produce unphysiological responses according to conservative criteria that we detail below. We then examine the behavior of each of these networks and perform statistics on the various kinds of responses that have been identified in the theoretical analysis of 2.1.

We thus constructed 1000 networks by sampling both feedforward weights $\{g_\alpha\}$ and recurrent weights $\{J_{\alpha\beta}\}$ (for $\alpha, \beta \in \{E, I\}$) uniformly from the interval [0.1; 1], and subsequently normalizing their (vector) L_∞-norm such that $\max(g_\alpha) = \max(J_{\alpha\beta}) = 1$. We then sampled the overall connectivity strength ψ (cf. 2.1) from the interval [0.1; 10]. This interval was based on rough estimates of the average number of input connections from the local network per neuron (between 200 and 1000), average PSP amplitude (between 0.1 mV and 0.5 mV) and decay time constants (5 to 20 ms), giving a range of connectivity strengths – which in our model is the product of these three quantities – between 0.1 and 10 mV/Hz.

Instead of choosing a range of α and simulating the dynamics of Equation S16 to compute mean voltages, we observed that $\bar{y}_I$ increases monotonically with α and for each network we chose a range

[1]Arguments about how $\bar{y}_E$ scales with large α actually become invalid when $\Omega_E < 0$ precisely because for large enough α the E unit stops firing; but the point here is that because $\bar{y}_E$ must decrease at some point, it will necessarily become strongly sublinear in α over some range before it starts to decrease.

of $\overline{y}_{\mathrm{I}}$ corresponding to mean I firing rates $((\overline{y}_{\mathrm{I}}/2\,\psi)^2/k)$ in the 0–200 Hz range, thus assuming that mean I responses above 200 Hz would be unphysiological. For each $\overline{y}_{\mathrm{I}}$ in this discretized range we solved for $\overline{y}_{\mathrm{E}}$ analytically by noting that the input α can be eliminated from the pair of fixed-point equations (Equation S16 with l.h.s. set to zero), yielding a fixed-point curve in the $(\overline{y}_{\mathrm{E}}, \overline{y}_{\mathrm{I}})$ plane:

$$\Omega_{\mathrm{I}}\,\overline{y}_{\mathrm{E}}^{\,2} + 2\,g_{\mathrm{I}}\,\overline{y}_{\mathrm{E}} = \Omega_{\mathrm{E}}\,\overline{y}_{\mathrm{I}}^{\,2} + 2\,g_{\mathrm{E}}\,\overline{y}_{\mathrm{I}} \tag{S17}$$

Given $\overline{y}_{\mathrm{I}}$ it is easy to solve this quadratic equation for $\overline{y}_{\mathrm{E}}$. We rejected those parameters sets for which we encountered either i) complex solutions for $\overline{y}_{\mathrm{E}}$, or ii) real but unstable solutions, as assessed by the stability conditions $\mathrm{Tr}\,\mathcal{J} < 0$ and $\mathrm{Det}\,\mathcal{J} > 0.01$ (with the Jacobian matrix $\mathcal{J}$ defined in Equations S19 and S22), or iii) stable solutions that involved E firing rates $((\overline{y}_{\mathrm{E}}/2\,\psi)^2/k)$ either greater than 200 Hz, or smaller than 1 Hz for the largest value of $\overline{y}_{\mathrm{I}}$. Finally, for each fixed point $(\overline{y}_{\mathrm{E}}, \overline{y}_{\mathrm{I}})$, we computed the corresponding α from either of the two fixed-point equations (Equation S16 with l.h.s. set to zero), e.g. $\alpha = \left[\overline{y}_{\mathrm{E}} - (J_{\mathrm{EE}}\,\overline{y}_{\mathrm{E}}^{\,2} - J_{\mathrm{EI}}\,\overline{y}_{\mathrm{I}}^{\,2})/2\right]/g_{\mathrm{E}}$. This procedure was numerically much more efficient than simulating the dynamics of Equation S16 until convergence to steady-state.

The parameters of the retained networks spanned a large chunk of the intervals in which they were sampled (Figure S1A and B). Because stability for large α requires $\mathrm{Det}\,\mathbf{J} > 0$, i.e. $J_{\mathrm{EI}}\,J_{\mathrm{IE}} > J_{\mathrm{EE}}\,J_{\mathrm{II}}$, the largest of all sampled $J_{\alpha\beta}$'s was often either J_{EI} or J_{IE} which then, due to the L_∞-norm normalization, assumed a value of one (Figure S1A). We also observed that the input weight g_{E} was often larger than g_{I} (Figure S1B). About 90% of the sampled networks had $\Omega_{\mathrm{E}} > 0$, implying $\sim \sqrt{\alpha}$ scaling of $\overline{y}_{\mathrm{E}}$ and $\overline{y}_{\mathrm{I}}$ for large α (example in Figure S1D, top). In these networks, E and I rates were linear in α for α large enough, and so were also linear in each other when large enough (Figure S1E, black). The rest of the networks (10%) had $\Omega_{\mathrm{E}} < 0$ and therefore showed supersaturation of the E firing rate for large input (Figure S1D, bottom) and E responses that were sublinear in I responses (Figure S1E, orange).

It is worth noting that for networks with small overall connectivity strength ψ, the proportion of $\Omega_{\mathrm{E}} < 0$ and $\Omega_{\mathrm{E}} > 0$ cases tend to even out (Figure S1C). This is because, for supersaturating networks, the peak E firing rate is inversely proportional to ψ^2 (Ahmadian et al., 2013), so for large ψ the peak firing rate is low and therefore the final value of $\overline{r}_{\mathrm{E}}$ reached for $\overline{r}_{\mathrm{I}} = 200$ Hz likely falls below our threshold of 1 Hz, resulting in a rejection of the parameter set.

In sum, the nonlinear properties of the SSN's responses to growing inputs, summarized in 2.1, are robust to changes in parameters so long as these keep the network in a regime "not too unphysiological" in a conservative sense. Using the same collection of sampled networks, we will show below that the modulation of variability with input described in the main text is equally robust to parameter changes.

Methods S3 Membrane potential variability in the two-population SSN model

In this section, we derive the theoretical results regarding activity variability in the two-population model of the main text. We use these analytical results to demonstrate robustness of our results to changes in parameters, which we also verify numerically using the collection of networks with randomly sampled parameters introduced in 2.2.

3.1 Linearization of the dynamics

We now consider the noisy dynamics of the two-population model of the main text in which the E and I units represent the average activity of large E and I populations. To study variability analytically, we linearize Equation S1 around the mean, thus examining the local behavior of small fluctuations $\delta\mathbf{V}$:

$$\tau_{\mathrm{E}}\,\mathbf{T}\,\frac{\mathrm{d}\delta\mathbf{V}}{\mathrm{d}t} = \mathbf{A}(\alpha)\,\delta\mathbf{V}(t) + \boldsymbol{\eta}(t) \tag{S18}$$

$$\text{with} \quad \mathbf{A}(\alpha) \equiv -\mathbf{I} + \mathbf{W}^{\mathrm{eff}}(\alpha) \tag{S19}$$

The effective connectivity $\mathbf{W}^{\text{eff}}$ depends on the (dimensionless) input α through its dependence on mean responses, following

$$W_{ij}^{\text{eff}}(\alpha) = n\,k\,W_{ij}\,\lfloor \overline{V}_j(\alpha) \rfloor_+^{n-1} \qquad \text{for} \quad i,j \in \{\text{E}, \text{I}\} \tag{S20}$$

For $n = 2$, Equation S20 can also be written using the definition of the dimensionless voltage $\bar{\mathbf{y}}$ and dimensionless connections $\mathbf{J}$ introduced in 2.1 as

$$W_{ij}^{\text{eff}}(\alpha) = J_{ij}\,\lfloor \bar{y}_j(\alpha) \rfloor_+ \tag{S21}$$

With our notations, the Jacobian matrix

$$\mathcal{J}(\alpha) \equiv \mathbf{T}^{-1}\,\mathbf{A}(\alpha) \tag{S22}$$

is unitless, so that, e.g., the interpretation of a real negative eigenvalue λ of $\mathcal{J}$ is that the corresponding eigenmode decays asymptotically with time constant $\tau_\text{E}/|\lambda|$ as a result of the recurrent dynamics. We parameterize the input noise covariance as

$$\left\langle \boldsymbol{\eta}(t)\,\boldsymbol{\eta}(t+\tau)^\mathsf{T} \right\rangle = \left(1 + \frac{1}{\bar{\tau}_\eta}\right)\, e^{-|\tau|/\tau_\eta} \begin{pmatrix} c_\text{E}^2 & c_\text{EI} \\ c_\text{EI} & c_\text{I}^2 \end{pmatrix} \qquad \text{with} \quad c_\text{EI} \equiv \rho_\text{EI}\,c_\text{E}\,c_\text{I} \tag{S23}$$

such that, in the limit of small α – in which the network is effectively unconnected, because $\lfloor \bar{\mathbf{y}} \rfloor$ in Equation S20 is small – the E unit has variance c_E^2; the I unit then has variance $\frac{1+\bar{\tau}_\eta}{\bar{\tau}_\text{I}+\bar{\tau}_\eta}\,c_\text{I}^2$. The parameter ρ_EI determines the correlation between input noise to the E and I units.

3.2 General result

The full output covariance matrix $\mathbf{\Sigma} \equiv \langle \delta\mathbf{V}\,\delta\mathbf{V}^\mathsf{T} \rangle$ can be calculated by solving a set of linear equations[2], which yields:

$$\mathbf{\Sigma} = \frac{(1+\bar{\tau}_\eta)(1-\bar{\tau}_\eta\,\text{Tr}\,\mathcal{J})}{-\text{Tr}\,\mathcal{J}\,\text{Det}\,\mathbf{A}\,(\bar{\tau}_\text{I} - \bar{\tau}_\text{I}\,\bar{\tau}_\eta\,\text{Tr}\,\mathcal{J} + \bar{\tau}_\eta^2\,\text{Det}\,\mathbf{A})} \begin{pmatrix} \Sigma_\text{EE}^\star & \Sigma_\text{EI}^\star \\ \Sigma_\text{EI}^\star & \Sigma_\text{II}^\star \end{pmatrix} \tag{S26}$$

with

$$\Sigma_\text{EE}^\star = c_\text{E}^2 \left(\frac{\bar{\tau}_\text{I}\,\text{Det}\,\mathbf{A}}{1-\bar{\tau}_\eta\,\text{Tr}\,\mathcal{J}} + A_\text{II}^2 \right) + c_\text{I}^2\,A_\text{EI}^2 - 2\,c_\text{EI}\,A_\text{EI}\,A_\text{II} \tag{S27}$$

$$\Sigma_\text{II}^\star = c_\text{I}^2 \left(\frac{\bar{\tau}_\text{I}^{-1}\,\text{Det}\,\mathbf{A}}{1-\bar{\tau}_\eta\,\text{Tr}\,\mathcal{J}} + A_\text{EE}^2 \right) + c_\text{E}^2\,A_\text{IE}^2 - 2\,c_\text{EI}\,A_\text{IE}\,A_\text{EE} \tag{S28}$$

$$\Sigma_\text{EI}^\star = c_\text{E}^2\,A_\text{IE}\,A_\text{II} + c_\text{I}^2\,A_\text{EI}\,A_\text{EE} - 2\,c_\text{EI} \left(A_\text{EE}\,A_\text{II} - \frac{\bar{\tau}_\eta\,\text{Tr}\,\mathcal{J}\,\text{Det}\,\mathbf{A}}{2\,(1-\bar{\tau}_\eta\,\text{Tr}\,\mathcal{J})} \right) \tag{S29}$$

---

[2] Since the spatial and temporal correlations in the noise term $\boldsymbol{\eta}$ in Equation S18 are separable, we can augment the state space with two noise units and write their (linear) Langevin dynamics as

$$\tau_\text{E}\,d\begin{pmatrix} \delta\mathbf{V} \\ \boldsymbol{\eta} \end{pmatrix} = \begin{pmatrix} \mathbf{A}(h) & \mathbf{I} \\ 0 & -\frac{\tau_\text{E}}{\tau_\eta}\mathbf{I} \end{pmatrix} \begin{pmatrix} \delta\mathbf{V} \\ \boldsymbol{\eta} \end{pmatrix} dt + \begin{pmatrix} 0 & 0 \\ 0 & \tau_\text{E}\sqrt{\frac{2}{\tau_\eta}}\mathbf{B} \end{pmatrix} \text{d}\boldsymbol{\xi} \tag{S24}$$

where $\text{d}\boldsymbol{\xi}$ is a unit-variance, spherical Wiener process, and $\mathbf{B}$ is the Cholesky factor of the desired noise covariance matrix, that is, $\mathbf{\Sigma}_\eta = \mathbf{B}\mathbf{B}^\mathsf{T}$ (the $\tau_\text{E}\sqrt{2/\tau_\eta}$ factor is such that this equality holds). Then, from multivariate Ornstein-Uhlenbeck process theory (e.g. Hennequin et al., 2014), we know that the covariance matrix of the compound process satisfies the following Lyapunov equation:

$$\begin{pmatrix} \mathbf{A} & \mathbf{I} \\ 0 & -\frac{\tau_\text{E}}{\tau_\eta}\mathbf{I} \end{pmatrix} \begin{pmatrix} \mathbf{\Sigma} & \mathbf{\Lambda} \\ \mathbf{\Lambda}^\mathsf{T} & \mathbf{\Sigma}_\eta \end{pmatrix} + \begin{pmatrix} \mathbf{\Sigma} & \mathbf{\Lambda} \\ \mathbf{\Lambda}^\mathsf{T} & \mathbf{\Sigma}_\eta \end{pmatrix} \begin{pmatrix} \mathbf{A}^\mathsf{T} & 0 \\ \mathbf{I} & -\frac{\tau_\text{E}}{\tau_\eta}\mathbf{I} \end{pmatrix} = -\begin{pmatrix} 0 & 0 \\ 0 & 2\frac{\tau_\text{E}}{\tau_\eta}\mathbf{B}\mathbf{B}^\mathsf{T} \end{pmatrix} \tag{S25}$$

where $\mathbf{\Sigma}$ is the covariance we are trying to compute. By vectorizing Equation S25, neglecting the bottom right quadrant (which by itself only confirms $\mathbf{\Sigma}_\eta = \mathbf{B}\mathbf{B}^\mathsf{T}$ as promised above), and taking into account the symmetry, one ends up with a system of 7 coupled but *linear* equations to solve for the 3 unknowns of $\mathbf{\Sigma}$ and the 4 unknowns of $\mathbf{\Lambda}$. This can be done by hand using some patience, or automatically using a symbolic solver such as Mathematica, and yields the expression in Equation S26.

In Equations S26 to S29, each term that depends on $\mathbf{A}$ or $\mathcal{J}$ depends implicitly on the (dimensionless) constant input α delivered to both E and I populations, because $\mathbf{A}$ (and $\mathcal{J}$) depends on mean voltages (through Equation S20) which themselves depend on α. Note also that, for the network to be stable at a given input level α, the Jacobian matrix $\mathcal{J}(\alpha)$ should obey $\mathrm{Tr}\,\mathcal{J} < 0$ and $\mathrm{Det}\,\mathcal{J} > 0$ (with the latter equivalent to $\mathrm{Det}\,\mathbf{A} > 0$).

Among other things, we will analyze the behaviour of the total variance, i.e. the trace of $\mathbf{\Sigma}$ given by

$$\mathrm{Tr}(\mathbf{\Sigma}) = (1 + \bar{\tau}_\eta) \frac{\beta(\mathbf{A})\,(1 - \bar{\tau}_\eta \mathrm{Tr}\,\mathcal{J}) + \mathrm{Det}\,\mathbf{A}\,(\bar{\tau}_\mathrm{I}\, c_\mathrm{E}^2 + \bar{\tau}_\mathrm{I}^{-1}\, c_\mathrm{I}^2)}{-\mathrm{Tr}\,\mathcal{J}\;\mathrm{Det}\,\mathbf{A}\,\left(\bar{\tau}_\mathrm{I} - \bar{\tau}_\mathrm{I}\,\bar{\tau}_\eta\,\mathrm{Tr}\,\mathcal{J} + \bar{\tau}_\eta^2\,\mathrm{Det}\,\mathbf{A}\right)} \tag{S30}$$

with $\mathbf{A}$ defined in Equation S19 and

$$\beta(\mathbf{A}) \equiv (A_\mathrm{IE}^2 + A_\mathrm{II}^2)\, c_\mathrm{E}^2 + (A_\mathrm{EI}^2 + A_\mathrm{EE}^2)\, c_\mathrm{I}^2 - 2\,(A_\mathrm{IE}\, A_\mathrm{EE} + A_\mathrm{EI}\, A_\mathrm{II})\, c_\mathrm{EI} \tag{S31}$$

3.3 Analysis in simplified scenarios

In order to understand what Equation S30 tells us about the modulation of variability with the input α, we make a couple of assumptions that greatly simplify the expression for the total variance with little loss of generality. First, we consider the limit of slow[3] input noise which we find empirically is approached rather fast, with $\tau_\eta = 50$ ms already giving a close approximation given $\tau_\mathrm{E} = 20$ ms and $\tau_\mathrm{I} = 10$ ms. Next, we assume that

$$c_\mathrm{E} = \frac{c_\mathrm{I}}{\kappa} \equiv c \tag{S32}$$

and $\rho_\mathrm{EI} = 0$ (implying $c_\mathrm{EI} = 0$), i.e. the E and I units have uncorrelated input fluctuations of equal amplitude (the impact of positive input correlations, $\rho_\mathrm{EI} > 0$, will be discussed in 3.4). With these two assumptions, the total variance simplifies into

$$\mathrm{Tr}(\mathbf{\Sigma}) = c^2\,\frac{\beta_0(\mathbf{A})}{\mathrm{Det}\,\mathbf{A}^2} = c^2\,\frac{A_\mathrm{IE}^2 + A_\mathrm{II}^2 + A_\mathrm{EI}^2 + A_\mathrm{EE}^2}{(A_\mathrm{EE}\,A_\mathrm{II} - A_\mathrm{EI}\,A_\mathrm{IE})^2} \tag{S33}$$

where we defined $\beta_0(\mathbf{A}) \equiv \beta(\mathbf{A})/c^2$.

There are two ways to understand how total variance scales with inputs. First, somewhat loosely and indirectly, via its scaling with mean responses. As mean voltage responses increase with the stimulus, so do the effective weights, which – for a large enough input and a general threshold-powerlaw input/output nonlinearity with exponent n – are proportional to $\bar{y}^{n-1}$ (Equation S20). As the numerator of Equation S33 is quadratic in $\mathbf{A}$ and thus also in the effective weights in the large input limit, while the denominator is quartic, the overall scaling is going to be inverse quadratic in the effective weights, yielding a total voltage variance which scales with mean responses approximately as

$$\mathrm{Tr}(\mathbf{\Sigma}) \propto 1/\bar{y}^{2(n-1)} \tag{S34}$$

Second, for the special case of a threshold-quadratic nonlinearity ($n = 2$), we can also understand the scaling of the total variance directly with the input strength, α, in more precise terms. The typical behavior of $\beta_0(\mathbf{A})^{1/2}$ and $\mathrm{Det}\,\mathbf{A}$ is shown in Figure S2A. Both can be expressed as functions of mean responses using Equations S19 and S20:

$$\beta_0(\mathbf{A}) = \kappa^2\,(J_\mathrm{EE}\,\bar{y}_\mathrm{E} - 1)^2 + \kappa^2\,(J_\mathrm{EI}\,\bar{y}_\mathrm{I})^2 + (J_\mathrm{IE}\,\bar{y}_\mathrm{E})^2 + (1 + J_\mathrm{II}\,\bar{y}_\mathrm{I})^2 \tag{S35}$$

$$\mathrm{Det}\,\mathbf{A}^2 = \left[(J_\mathrm{IE}\,\bar{y}_\mathrm{E})\,(J_\mathrm{EI}\,\bar{y}_\mathrm{I}) + (1 - J_\mathrm{EE}\,\bar{y}_\mathrm{E})\,(1 + J_\mathrm{II}\,\bar{y}_\mathrm{I})\right]^2 \tag{S36}$$

[3] The other limit (fast noise, $\tau_\eta \to 0$) also greatly simplifies Equation S30, but would not make much sense in the context of this study, since Equation S1 is meant to model the dynamics of the voltage on a timescale $\geq$ 30 ms, which is the timescale on which a threshold power-law relationship between voltage and rate has been measured in cat V1. Therefore, the input noise that we explicitly model here is meant to capture the slowly fluctuating components of external inputs, the fast components having been “absorbed” into the threshold power-law gain function.

Note that to simplify notations we have dropped the $\lfloor \cdot \rfloor_+$ that should surround every $\overline{y}$. Based on these expressions, we now examine the behavior of variability in the small and large α limits and show that the total variance should typically grow and then decay with increasing α, and therefore should exhibit a maximum which empirically we find occurs for $\alpha \sim 1$.

Behavior of the total variance for small α Using Equations S33, S35 and S36, we find the slope of the total variance at $\alpha = 0$ to be

$$\frac{\mathrm{d}}{\mathrm{d}\alpha}\mathrm{Tr}(\mathbf{\Sigma})\bigg|_{\alpha=0} = 2\,c^2\,\left(g_\mathrm{E}\,J_\mathrm{EE} - \kappa^2\,g_\mathrm{I}\,J_\mathrm{II}\right) \tag{S37}$$

Thus, when the noise power fed to inhibitory cells is sufficiently small, $\kappa = c_\mathrm{I}/c_\mathrm{E}$ will be small enough that the expression in Equation S37 will stay positive, and therefore total variability will grow with small increasing α. Indeed, we find that this happens for most (>90%) of the randomly sampled networks of 2.2 with κ as large as 1/2 (Figure S2A, bottom). Moreover, restricting the analysis to the E unit gives $\mathrm{d}\Sigma_\mathrm{EE}/\mathrm{d}\alpha|_{\alpha=0} = 2\,c^2\,g_\mathrm{E}\,J_\mathrm{EE}$ which is always positive, independently of κ. Thus, for slow enough input noise, the variability in the E unit always increases with small α.

We can extend this argument to slightly larger values of α by further inspecting the numerator and denominator in Equation S33. Although the first term in the numerator, $(J_\mathrm{EE}\,\overline{y}_\mathrm{E} - 1)^2$, originally decays with α as $\overline{y}_\mathrm{E}$ grows from 0 to $1/J_\mathrm{EE}$, the other three terms always grow with α as long as mean voltages do, and thus we expect the numerator to typically grow. This is indeed what we find in all sampled networks (Figure S2A). On the other hand, the denominator (Equation S36) is the square of the sum of two terms, the first one initially small and growing, and the second one initially large and decaying. Indeed, the second term starts at 1 for $\alpha = 0$, because the $\overline{y}$ terms are all zero, and then decays to zero as the network enters the inhibition-stabilized (ISN) regime and the effective excitatory feedback gain $J_\mathrm{EE}\,\overline{y}_\mathrm{E}$ becomes larger than one[4] (Tsodyks et al., 1997; Ozeki et al., 2009). Thus, due to this partial cancellation of growing and decaying terms, we expect the denominator to either decrease, or grow very slowly, with increasing α (Figure S2A), until it starts growing faster (see arguments below for the large α case) in the very rough neighborhood of the ISN transition. All in all, the ratio of a fast growing numerator to a slower growing denominator suggests that the total variance should robustly grow with small increasing α (Figure S2A, bottom).

Behavior of the total variance for large α As the input grows, so do the mean (dimensionless) voltages $\overline{y}_\mathrm{E}$ and $\overline{y}_\mathrm{I}$ at least over some range of α. Therefore, we expect *both* the numerator *and* the denominator that make up the total variance in Equation S33 to grow with large enough and increasing α. However, loosely speaking, the numerator grows as $\overline{y}^2$ while the denominator grows as $\overline{y}^4$, which can be seen by inspecting Equations S35 and S36. Thus, their ratio should decrease roughly as

$$\mathrm{Tr}(\Sigma) \propto \frac{1}{\overline{y}^2} \tag{S38}$$

which is just a special case for $n = 2$ of the generic result in Equation S34 for arbitrary n.

However, here (for $n = 2$) this argument can be made more rigorous in the case of $\Omega_\mathrm{E} > 0$, i.e. when the E unit does not supersaturate. In this case, from Equation S15 we have $\overline{y}_\mathrm{E} \approx \sqrt{2\,\Omega_\mathrm{E}\,\alpha/\mathrm{Det}\,\mathbf{J}}$ and $\overline{y}_\mathrm{I} \approx \sqrt{2\,\Omega_\mathrm{I}\,\alpha/\mathrm{Det}\,\mathbf{J}}$ for α large enough. Therefore, in the large α limit, the numerator and denominator of Equation S33 respectively behave as

$$\beta_0(\mathbf{A}) \approx \frac{2}{\mathrm{Det}\,\mathbf{J}}\left[(J_\mathrm{IE}^2 + \kappa^2\,J_\mathrm{EE}^2)\,\Omega_\mathrm{E} + (J_\mathrm{II}^2 + \kappa^2\,J_\mathrm{EI}^2)\,\Omega_\mathrm{I}\right]\alpha \tag{S39}$$

$$\mathrm{Det}\,\mathbf{A}^2 \approx 4\Omega_\mathrm{E}\,\Omega_\mathrm{I}\,\alpha^2 \tag{S40}$$

[4]In this regime, $J_\mathrm{EE}\,\overline{y}_\mathrm{E} > 1 \Leftrightarrow A_\mathrm{EE} > 0$ implies instability of the excitatory subnetwork in isolation, and therefore the need for dynamic, stabilizing feedback inhibition (hence the name "inhibition-stabilized network").

therefore the total variance (their ratio) decreases as $1/\alpha$. For $\Omega_E < 0$, the large α limit is irrelevant strictly speaking, as in this limit $\lfloor \bar{y}_E \rfloor_+$ and $\bar{r}_E$ go to zero. In this case the total variance does not decrease asymptotically but reaches a finite limit of $c^2 \left[1 + (\bar{\tau}_I J_{EI}/J_{II})^2\right]$. However, we find empirically that the peak of variability always occurs well before the onset of supersaturation, in a regime where both $\bar{y}_E$ and $\bar{y}_I$ are still growing with α while remaining roughly proportional to each other (Figure S1E), so that the argument made above can be repeated: the total variance decreases as $1/\bar{y}^2$ for a while after having peaked.

Where does variability peak? The above arguments, derived for slow noise $\tau_\eta \to \infty$, show that growing inputs typically increase, and then suppress, total variability in the two-population SSN. Thus, total variability (and even more certainly, variability in the E unit) typically exhibits a maximum for some intermediate value of α. We find empirically that, even for finite τ_η, the location of this variance peak is well approximated by its location in the limit of fast inhibition, $\bar{\tau}_I \to 0$, which we can estimate analytically. Indeed, in this limit, the I cell responds instantaneously to changes in E activity and input noise, such that

$$\delta V_I(t) = \frac{J_{IE}\, \bar{y}_E\, \delta V_E(t) + \eta_I(t)}{1 + J_{II}\, \bar{y}_I} \tag{S41}$$

Consequently, δV_E now obeys one-dimensional dynamics given by

$$\tau_E\, \delta\dot{V}_E = -\lambda\, \delta V_E(t) + \eta_{eff}(t) \tag{S42}$$

where

$$\lambda = 1 + \frac{\bar{y}_E\, (\mathrm{Det}\, \mathbf{J}\, \bar{y}_I - J_{EE})}{1 + J_{II}\, \bar{y}_I} \tag{S43}$$

and η_{eff} is a noise process (a linear combination of η_E and η_I) with temporal correlation length τ_η and a variance that is empirically irrelevant for the arguments below[5]. In this case, the variance of δV_E is inversely proportional to $\lambda\,(\frac{1}{\bar{\tau}_\eta} + \lambda)$, and therefore should be maximum at the input level α that minimizes λ. Observing from Figure S1E that $\bar{y}_E$ and $\bar{y}_I$ are roughly proportional over a large range of α (for $\Omega_E < 0$), if not the entire range (for $\Omega_E > 0$), we can make the following approximation:

$$\lambda - 1 \propto \frac{\bar{y}_I\, (\mathrm{Det}\, \mathbf{J}\, \bar{y}_I - J_{EE})}{1 + J_{II}\, \bar{y}_I} \tag{S44}$$

whose minimum is straightforward to calculate and is attained for

$$\bar{y}_I = \frac{1}{J_{II}} \left(\sqrt{\frac{J_{EI}\, J_{IE}}{\mathrm{Det}\, \mathbf{J}}} - 1 \right) \tag{S45}$$

We find that the α of maximum variance in the E unit is indeed very well approximated by the α at which $\bar{y}_I$ reaches the threshold value of Equation S45, especially in the absence of input correlations ($\rho_{EI} = 0$, Figure S2B, left). For correlated noisy inputs, the criterion of Equation S45 deteriorates slightly but still consistently provides an upper bound on the α of maximum E variance (Figure S2B, right).

Interestingly, the criterion for maximum variance in Equation S45 is equivalent to a criterion about the effective I→I connection, given by $W_{II}^{eff} \equiv 2\, k\, \lfloor \bar{V}_I \rfloor_+ W_{II}$ (cf. Equation 1 in main text). Specifically, at the peak of variance we expect to have

$$W_{II}^{eff} = \sqrt{\frac{1}{1-\beta}} - 1 \qquad \text{with } \beta \equiv \frac{W_{EE}\, W_{II}}{W_{EI}\, W_{IE}} \tag{S46}$$

where $\beta < 1$ is in some sense the ratio of what contributes positively to the activity of the E cell (product of self-excitation W_{EE} with disinhibition W_{II}) to what contributes negatively to it (the product

[5] The variance of the effective noise process is proportional to $1 + \frac{J_{IE}^2\, \bar{y}_I^{\,2}}{(1+J_{II}\, \bar{y}_I)^2}$, and so has some dependence on α especially for small α before $\bar{y}_I$ grows large. However, empirically, the quality of the approximation in Equation S44 – which is derived under the assumption of constant effective noise variance – suggests that we can neglect this effect.

$W_{\mathrm{IE}}\, W_{\mathrm{EI}}$ quantifying the strength of the E $\to$ I $\to$ E inhibitory feedback loop). Thus, in networks with inhibition-dominated connectivity, i.e. ones in which $\beta \ll 1$, we expect $W_{\mathrm{II}}^{\mathrm{eff}}$ to reach the criterion of Equation S46 earlier as the input grows (this argument implictly assumes that the rate of growth of $W_{\mathrm{II}}^{\mathrm{eff}}$ itself doesn't depend too much on β, which we could confirm numerically).

Finally, we note that since variability peaks for $\alpha \sim \mathcal{O}(1)$ and $y \sim \mathcal{O}(1)$, networks with stronger connectivity (large ψ) will exhibit a peak of variance for smaller external input h (because $\alpha \propto \psi\, h$) – and this peak will occur for lower voltages/firing rates (because $\overline{V} \propto y/\psi$).

3.4 Effects of input correlations

To see the effect of input correlations on variability, we return to the expression for Σ_{EE} in Equation S30, assume again that $\tau_\eta \to \infty$ and $c_{\mathrm{E}} = \frac{c_{\mathrm{I}}}{\kappa} = c$, but now with $\rho_{\mathrm{EI}} \neq 0$. We thus obtain:

$$\Sigma_{\mathrm{EE}} = c^2\, \frac{A_{\mathrm{II}}^2 + \kappa^2 A_{\mathrm{EI}}^2}{\mathrm{Det}\,\mathbf{A}^2} - 2\, c^2\, \rho_{\mathrm{EI}}\, \frac{\kappa\, A_{\mathrm{II}}\, A_{\mathrm{EI}}}{\mathrm{Det}\,\mathbf{A}^2} \tag{S47}$$

Thus, total E variability is equal to that without input correlation (the first term), minus a positive term proportional to ρ_{EI}. Thus, positive input correlations always decrease variability in the E unit (and, in particular, its peak; Figure S2C, right), while negative correlations increase it. Moreover, the subtracted term has the same large-α behavior as the first term, because the two terms share the same denominator and for large alpha both numerators are $\mathcal{O}(\overline{y}_{\mathrm{I}}{}^2)$. Thus, input correlations should not affect the qualitative, decreasing behaviour of E variance for large increasing inputs. For small α and large ρ_{EI}, however, we expect $A_{\mathrm{II}}^2 + \kappa^2\, A_{\mathrm{EI}}^2 - 2\, \rho_{\mathrm{EI}}\, \kappa\, A_{\mathrm{II}}\, A_{\mathrm{EI}}$ to grow much more slowly than $A_{\mathrm{II}}^2 + \kappa^2\, A_{\mathrm{EI}}^2$; and indeed, in the extreme case $\rho_{\mathrm{EI}} = 1$, the total numerator becomes $(1 + (J_{\mathrm{II}} - \kappa\, J_{\mathrm{EI}})\, \overline{y}_{\mathrm{I}})^2$, which can even decrease transiently with increasing α if $\kappa\, J_{\mathrm{EI}} > J_{\mathrm{II}}$ (this occurs in about half of our thousand networks). This, in effect, shifts the peak of E variability to smaller values of α (Figure S2C, left).

The situation for the I unit is a bit different, as input correlations affect the I variance differently depending on whether the network has already made the transition to the ISN regime. Indeed, under the same assumptions as above, the I variance is given by

$$\Sigma_{\mathrm{II}} = c^2\, \frac{\kappa^2\, A_{\mathrm{EE}}^2 + A_{\mathrm{IE}}^2}{\mathrm{Det}\,\mathbf{A}^2} - 2\, c^2\, \rho_{\mathrm{EI}}\, \frac{\kappa\, A_{\mathrm{EE}}\, A_{\mathrm{IE}}}{\mathrm{Det}\,\mathbf{A}^2} \tag{S48}$$

In the ISN regime, $A_{\mathrm{EE}} > 0$, so that input correlations decrease I variability, just as they do for E variability as seen above. For small enough inputs, however, the network is not yet an ISN ($A_{\mathrm{EE}} < 0$), so that the effect of correlations is reversed: larger input correlations increase I variability.

In sum, input correlations modify the fine details of how large the variance grows and how early it peaks with increasing inputs, but they do not modify the qualitative aspects – in particular, the non-monotonic behavior – of variability modulation with external inputs in this two-population SSN model.

3.5 Mechanisms of variability modulation: Schur decomposition

We now unpack the mechanistic aspects of variability modulation in the SSN, by decomposing the effects of effective connectivity into two qualitatively different flow fields that shape the covariance of activities in the network in distinct ways (Figure S3): "shear" and "restoring" fields. To do this, we focus on the linearized dynamics of Equation S18 and perform a Schur decomposition of the Jacobian matrix in Equation S22 (which includes both the single-neuron leak and the effective connectivity; Murphy and Miller, 2009):

$$\mathcal{J}(\alpha) = \mathbf{U}(\alpha)\, \mathbf{T}_{\mathrm{Schur}}(\alpha)\, \mathbf{U}(\alpha)^\star \qquad \text{with} \quad \mathbf{T}_{\mathrm{Schur}}(\alpha) \equiv \begin{pmatrix} \lambda_{\mathrm{s}} & w_{\mathrm{FF}} \\ 0 & \lambda_{\mathrm{d}} \end{pmatrix} \tag{S49}$$

where $\cdot^\star$ denotes the conjugate transpose, $\boldsymbol{\lambda}_s$ and $\boldsymbol{\lambda}_d$ are the two (either real or complex-conjugate[6]) eigenvalues of $\mathcal{J}(\alpha)$, the columns of $\mathbf{U}$ are the (orthonormal) Schur vectors such that $\mathbf{U}\,\mathbf{U}^\star = \mathbf{U}^\star\,\mathbf{U} = \mathbf{I}$, and $\mathbf{w}_{FF}$ is the feedforward weight coupling the dynamics of the Schur vectors. Expressing the E and I voltage fluctuations in the Schur basis as $\mathbf{z} \equiv \mathbf{U}^\star\,\delta\mathbf{V}$, their dynamics become

$$\tau_E \frac{d\mathbf{z}}{dt} = \mathbf{T}_{Schur}\,\mathbf{z} + \mathbf{U}^\star\,\mathbf{T}^{-1}\,\boldsymbol{\eta} \qquad \text{(S50)}$$

In the case of the 2-population E/I architecture considered here ($\mathbf{W}$ given by Equation 8 of the main text), the first Schur vector is a "sum mode" in the generalized sense (Murphy and Miller, 2009), i.e. its excitatory and inhibitory components have the same sign[7]. This corresponds to patterns of network activity in which the excitatory and inhibitory units are simultaneously either more active or less active than average. The second Schur mode is a generalized "difference mode" in that its excitatory and inhibitory components have opposive signs. (Hence the notations $\boldsymbol{\lambda}_s$ and $\boldsymbol{\lambda}_d$.) In theory, $\mathbf{U}$ depends on the input α, because $\mathcal{J}$ does. However, we find that past a relatively small value of α, the Schur vectors do not change much and are indeed sum-like and difference-like across all thousand networks studied in Methods S2 and Methods S3 (Figure S2E).

The Schur decomposition reveals through $\mathbf{T}_{Schur}(\alpha)$ a feedforward structure hidden in the effective, recurrent connectivity $\mathcal{J}(\alpha)$. The difference mode feeds the sum mode with an effective feedforward weight $\mathbf{w}_{FF}$ (also a complex number if the eigenvalues have an imaginary component), given by the upper right element of the triangular matrix $\mathbf{T}_{Schur}$ – graphically, this corresponds to the "shear" flow field in Figure S3. On top of this, both patterns inhibit themselves with the corresponding negative weight $\boldsymbol{\lambda}_d$ or $\boldsymbol{\lambda}_s$ – the "restoring" flow field in Figure S3. Note that the sum of squared moduli (squared Frobenius norm $\|\cdot\|_F^2$) is preserved by the unitary transformation $\mathcal{J} \mapsto \mathbf{U}^\star\,\mathcal{J}\,\mathbf{U} \equiv \mathbf{T}_{Schur}$, such that $\|\mathcal{J}\|_F^2 = \|\mathbf{T}_{Schur}\|_F^2$, i.e.

$$|\mathbf{w}_{FF}| = \sqrt{\|\mathcal{J}\|_F^2 - (|\boldsymbol{\lambda}_s|^2 + |\boldsymbol{\lambda}_d|^2)} \qquad \text{(S52)}$$

The calculation of the network covariance matrix (Equation S30) can also be performed in the Schur basis, and doing this sheds further light on the roles of $\boldsymbol{\lambda}_d$, $\boldsymbol{\lambda}_s$ and $\mathbf{w}_{FF}$ in shaping variability. We begin by observing that

$$\begin{aligned}
\mathrm{Tr}\,(\boldsymbol{\Sigma}) &= \mathrm{Tr}\left(\langle\delta\mathbf{V}\,\delta\mathbf{V}^{\mathsf{T}}\rangle\right) \\
&= \mathrm{Tr}\left(\langle\mathbf{U}\,\mathbf{z}\,\mathbf{z}^\star\,\mathbf{U}^\star\rangle\right) \\
&= \mathrm{Tr}\left(\mathbf{U}\langle\,\mathbf{z}\,\mathbf{z}^\star\,\rangle\mathbf{U}^\star\right) \\
&= \mathrm{Tr}\left(\langle\mathbf{z}\,\mathbf{z}^\star\rangle\right)
\end{aligned} \qquad \text{(S53)}$$

(the last step following from $\mathbf{U}\,\mathbf{U}^\star = \mathbf{I}$). Thus, the total variance is preserved in the Schur basis. Next, taking the Fourier transform of Equation S50 and rearranging term yields

$$\hat{\mathbf{z}}(\omega) = (i\,\omega\,\mathbf{I} - \mathbf{T}_{Schur})^{-1}\,\mathbf{U}^\star\,\mathbf{T}^{-1}\,\hat{\boldsymbol{\eta}}(\omega) \qquad \text{(S54)}$$

[6] The eigenvalues remain real over the entire input range for about half of the 1000 random networks studied throughout (all with $\bar{\tau}_I = 1/2$). In the second half, they go from real to complex-conjugate and then sometimes to real again.

[7] This holds when the eigenvalues of $\mathbf{A}$ are real. When they are complex conjugate, one can still perform a real Schur decomposition by orthogonalizing the imaginary part of the eigenvector against the real part, which yields

$$\mathbf{T}_{Schur} = \begin{pmatrix} \mathrm{Re}(\boldsymbol{\lambda}) & a_+ \\ a_- & \mathrm{Re}(\boldsymbol{\lambda}) \end{pmatrix} \qquad a_\pm \equiv \frac{\mathbf{w}_{FF} \pm \sqrt{\mathbf{w}_{FF}^2 + 4\,\mathrm{Im}(\boldsymbol{\lambda})^2}}{2} \qquad \text{(S51)}$$

and the two Schur vectors in this case are also sum-like and difference-like, in this order. At this point (anticipating to some extent what follows this footnote), we note that in the imaginary case, there is a small feedback term proportional to a_- from the sum-mode to the difference-mode. Thus, the picture of the flow fields drawn in Figure S3 is incomplete. However, we will see that in the slow-noise limit (which gives a very good approximation to the output covariance as seen in 3.3), the purely feedforward picture remains exact provided one replaces $\mathbf{w}_{FF}$, $\boldsymbol{\lambda}_d$ and $\boldsymbol{\lambda}_s$ by their moduli.

where $\hat{\cdot}$ denotes the Fourier transform and $\omega \equiv 2\pi f \tau_E$ is a dimensionless frequency. Moreover, according to Parseval's theorem we have

$$\mathrm{Tr}\left(\langle \mathbf{z}\,\mathbf{z}^\star \rangle\right) = \frac{1}{2\pi\,\tau_E} \int_{-\infty}^{+\infty} \mathrm{Tr}\left(\hat{\mathbf{z}}\,\hat{\mathbf{z}}^\star\right) \mathrm{d}\omega \tag{S55}$$

Thus, combining Equations S53 to S55 we get

$$\mathrm{Tr}(\boldsymbol{\Sigma}) = \frac{2\,\bar{\tau}_\eta}{\pi} \int_{-\infty}^{+\infty} \frac{\mathrm{Tr}\left[(i\,\omega\,\mathbf{I} - \mathbf{T}_{\text{Schur}})^{-1}\,\mathbf{U}^\star\,\tilde{\boldsymbol{\Sigma}}_\eta\,\mathbf{U}\,(i\,\omega\,\mathbf{I} - \mathbf{T}_{\text{Schur}})^{-\star}\right]}{1 + (\bar{\tau}_\eta\,\omega)^2} \mathrm{d}\omega \tag{S56}$$

where $\tilde{\boldsymbol{\Sigma}}_\eta \equiv \mathbf{T}^{-1}\,\boldsymbol{\Sigma}_\eta\,\mathbf{T}^{-1}$. To simplify the calculation we now assume uncorrelated input noise terms, with the power of noise input to E and I balanced such that $\kappa = \bar{\tau}_I$ and $\tilde{\boldsymbol{\Sigma}}_\eta = c^2\,(1 + 1/\bar{\tau}_\eta)\,\mathbf{I}$, leading to:

$$\begin{aligned}
\mathrm{Tr}(\boldsymbol{\Sigma}) &= \frac{(1+\bar{\tau}_\eta)\,c^2}{\pi} \int_{-\infty}^{+\infty} \frac{\mathrm{Tr}\left((i\,\omega\,\mathbf{I} - \mathbf{T}_{\text{Schur}})^{-1}(i\,\omega\,\mathbf{I} - \mathbf{T}_{\text{Schur}})^{-\star}\right)}{1 + (\bar{\tau}_\eta\,\omega)^2} \mathrm{d}\omega \\
&= \frac{(1+\bar{\tau}_\eta)\,c^2}{\pi} \int_{-\infty}^{+\infty} \frac{1}{1 + (\bar{\tau}_\eta\,\omega)^2} \left(\frac{1}{|i\,\omega - \boldsymbol{\lambda}_d|^2} + \frac{1}{|i\,\omega - \boldsymbol{\lambda}_s|^2} + \frac{|\mathbf{w}_{FF}|^2}{|i\,\omega - \boldsymbol{\lambda}_d|^2 |i\,\omega - \boldsymbol{\lambda}_s|^2} \right) \mathrm{d}\omega
\end{aligned} \tag{S57}$$

where the second equality comes from having inverted the upper-triangular matrix $i\,\omega\,\mathbf{I} - \mathbf{T}_{\text{Schur}}$ analytically and taken its squared Frobenius norm. Carrying out the integral gives

$$\mathrm{Tr}(\boldsymbol{\Sigma}) = (1+\bar{\tau}_\eta)\,c^2 \left(\frac{1 - \bar{\tau}_\eta\,\boldsymbol{\lambda}_s^r}{-\boldsymbol{\lambda}_s^r\,(1 - 2\,\bar{\tau}_\eta\,\boldsymbol{\lambda}_s^r + \bar{\tau}_\eta^2\,|\boldsymbol{\lambda}_s|^2)} + \frac{1 - \bar{\tau}_\eta\,\boldsymbol{\lambda}_d^r}{-\boldsymbol{\lambda}_d^r\,(1 - 2\,\bar{\tau}_\eta\,\boldsymbol{\lambda}_d^r + \bar{\tau}_\eta^2\,|\boldsymbol{\lambda}_d|^2)} \right. \tag{S58}$$

$$\left. + \frac{|\mathbf{w}_{FF}|^2\left[1 - \bar{\tau}_\eta\,(\boldsymbol{\lambda}_s + \boldsymbol{\lambda}_d)\right]}{-(\boldsymbol{\lambda}_s + \boldsymbol{\lambda}_d)\,|\boldsymbol{\lambda}_s|\,|\boldsymbol{\lambda}_d|\left[1 - \bar{\tau}_\eta\,(\boldsymbol{\lambda}_s + \boldsymbol{\lambda}_d) + \bar{\tau}_\eta^2\,|\boldsymbol{\lambda}_s|\,|\boldsymbol{\lambda}_d|\right]} \right) \tag{S59}$$

where $\boldsymbol{\lambda}_s^r$ and $\boldsymbol{\lambda}_d^r$ stand for the real parts of $\boldsymbol{\lambda}_s$ and $\boldsymbol{\lambda}_d$ respectively (they must both be negative for the dynamics to be stable).

This expression simplifies in the slow noise limit, $\bar{\tau}_\eta \to \infty$:[8]

$$\mathrm{Tr}(\boldsymbol{\Sigma}) \xrightarrow{\bar{\tau}_\eta \to \infty} c^2 \left(\frac{1}{|\boldsymbol{\lambda}_s|^2} + \frac{1}{|\boldsymbol{\lambda}_d|^2} + \frac{|\mathbf{w}_{FF}|^2}{|\boldsymbol{\lambda}_s|^2\,|\boldsymbol{\lambda}_d|^2} \right) \tag{S60}$$

In this limit, the simplified picture of the flow fields drawn in a plane of sum and difference activity (Figure S3) which assumed that they were real quantities, becomes accurate even when the eigenvalues of $\mathcal{J}$ are complex-conjugate (in which case, as mentioned above in Footnote 7, the sum-like mode feeds back onto the difference mode, although this interaction is much weaker than the opposite one). Indeed, in Equation S60, the elements of $\mathbf{T}_{\text{Schur}}$ are reduced to their moduli, so even when they are complex one can still interpret Equation S60 as the total variance in a system with the same real Schur vectors, real eigenvalues equal to $-|\boldsymbol{\lambda}_d|$ and $-|\boldsymbol{\lambda}_s|$ respectively, and a real feedforward weight equal to $|\mathbf{w}_{FF}|$.

Equation S60 shows in more detail how the shear and restoring flows contribute to variability. In loose terms, the total variance is a sum of two contributions: one that does not depend on $\mathbf{w}_{FF}$ and decreases with $1/|\boldsymbol{\lambda}|^2$, and one that grows with $|\mathbf{w}_{FF}|^2$ but is also divided by a term of order $\boldsymbol{\lambda}^4$ (where $\boldsymbol{\lambda}$ is a loose notation to denote the overall magnitude of the eigenvalues). Thus, as the input grows, the effect of the eigenvalues on variability becomes much stronger than that of balanced amplification. Such a

[8] More generally, for arbitrary $\bar{\tau}_I$, κ and ρ_{EI}, in the limit $\bar{\tau}_\eta \to \infty$, Equation S60 still holds, in precisely the same form, but in terms of the eigenvalues and feedforward Schur weight of $\mathbf{B}(\alpha) \equiv c\,\boldsymbol{\Sigma}_\eta^{-\frac{1}{2}}\,\mathbf{A}(\alpha)$ rather than of $\mathcal{J}(\alpha)$. This is because, in that limit, $\mathrm{Tr}(\boldsymbol{\Sigma}) = c^2\,\|\mathbf{B}^{-1}\|_F^2$. Note that $\bar{\tau}_I$ cannot affect the result in the limit $\tau_\eta \to \infty$; and that when $\kappa = \bar{\tau}_I$ and $\rho_{EI} = 0$, then $\mathcal{J}(\alpha) = \mathbf{B}(\alpha)$ and hence Equation S60 holds. To see why $\mathrm{Tr}(\boldsymbol{\Sigma}) = c^2\,\|\mathbf{B}^{-1}\|_F^2$ in this limit: most simply, in the slow noise limit, one can think of the noise $\boldsymbol{\eta}(t)$ in Equation S18 as a constant input and solve for its steady state $\delta\mathbf{V} = -\mathbf{A}^{-1}\,\boldsymbol{\eta}$, then form $\boldsymbol{\Sigma} = \langle \delta\mathbf{V}\,\delta\mathbf{V}^\mathsf{T} \rangle$.

dominance can also be understood from the structure of the flow fields that negative self-couplings and balanced amplification induce. Restoring flows are proportional to the distance from the origin: the stronger the momentary V_m deviation from the mean in any direction, the stronger the pull towards the origin in the same direction (Figure S3C, green arrows). In contrast, the shear flow grow along the difference axis while pointing in the orthogonal, sum direction, such that larger deviations in the sum do not imply larger shear flow (Figure S3C, orange arrows). Thus, self-inhibition leads to exponential temporal decay of activity fluctuations, whereas balanced amplification gives only linear growth. This explains why, for large enough input, V_m variability decreases with increasing input even when all flow fields grow in magnitude at the same rate (Figure S2A).

Equation S60 also shows that if one of the eigenvalues transiently weakens with increasing input, then variability should transiently grow. This explains a large part of the variability peak observed in the network of the main text, and indeed, it also predicts variability growth in most of the thousand networks investigated here. However, there are cases where variability transiently grows, without any weakening of eigenvalues (Figure S4A). In those cases, setting $\mathbf{w}_{FF}$ to 0 in Equation S60 wrongly predicts purely decaying variability (compare dashed and solid black lines in Figure S4A, bottom). Thus, in general, initial variability growth results from the combined effects of weaker inhibitory self-couplings *and* strong balanced amplification.

3.6 How do shear and restoring flow fields depend on the input?

The input dependence of the shear ($|\mathbf{w}_{FF}|$) and restoring ($|\boldsymbol{\lambda}_s|$, $|\boldsymbol{\lambda}_s|$) flows can be understood from the input dependence of mean responses ($\bar{y}_E$ and $\bar{y}_I$), which was examined previously in Methods S2. First, at $\alpha = 0$ (no input) the effective connectivity is zero, thus $\mathcal{J} = \mathrm{diag}(-1, -\bar{\tau}_I^{-1})$ and therefore the two eigenvalues are -1 and $-1/\bar{\tau}_I$. To see how the eigenvalues change with the input, let us note that for a 2×2 matrix, the sum of the eigenvalues is equal to the trace of the matrix while their product is equal to its determinant. Thus, when both eigenvalues are real (which they are for small enough α), both the arithmetic and geometric mean of $|\boldsymbol{\lambda}_s|$ and $|\boldsymbol{\lambda}_d|$ can be related to the elements of $\mathcal{J}$, which themselves depend directly on $\bar{y}_E$ and $\bar{y}_I$. This yields:

$$|\boldsymbol{\lambda}_s| + |\boldsymbol{\lambda}_d| = \bar{\tau}_I^{-1}\left[1 + \bar{\tau}_I + \left(J_{II}\,\bar{y}_I - \bar{\tau}_I\,J_{EE}\,\bar{y}_E\right)\right] \tag{S61}$$

and (S62)

$$|\boldsymbol{\lambda}_s|\,|\boldsymbol{\lambda}_d| = \bar{\tau}_I^{-1}\left[1 + \mathrm{Det}\,\mathbf{J}\,\bar{y}_E\,\bar{y}_I + \left(J_{II}\,\bar{y}_I - J_{EE}\,\bar{y}_E\right)\right] \tag{S63}$$

We see that, by both measures, the overall restoring flow tends to grow with increasing input α, because i) mean responses grow too, and therefore so does the product term in Equation S63, and ii) $\bar{y}_I$ tends to grow larger than $\bar{y}_E$ (Figure S1E), so that the weighted difference terms inside round brackets in both Equations S61 and S63 increase, at least for large enough α. However, when $g_E\,J_{EE} > g_I\,J_{II}$, the difference term in Equation S63 will initially grow negative with increasing – but small – α, before it increases again for larger α. This means that at least one of the eigenvalues will decrease. In such a case, whether or not *both* eigenvalues decrease transiently depends on the behavior of the difference term in Equation S61. The requirement for this difference term to decrease initially is $\bar{\tau}_I\,g_E\,J_{EE} > g_I\,J_{II}$ which is harder to satisfy especially when inhibition is fast ($\bar{\tau}_I$ is small). Thus, we typically expect that one eigenvalue should decrease (or, at least, its growth should be delayed) before growing again (Figure S2A).

As for the shear flow, a similarly simple expression can be obtained in the case of real eigenvalues by noting that the sum of squared eigenvalues in 2×2 matrix $\mathcal{J}$ is equal to $(\mathrm{Tr}\,\mathcal{J})^2 - 2\,\mathrm{Det}\,\mathcal{J}$. This observation yields

$$\begin{aligned} |\mathbf{w}_{FF}| &= \sqrt{\|\mathcal{J}\|_F^2 - (\mathrm{Tr}\,\mathcal{J})^2 + 2\,\mathrm{Det}\,\mathcal{J}} \\ &= \bar{\tau}_I^{-1}\left(J_{IE}\,\bar{y}_E + \bar{\tau}_I\,J_{EI}\,\bar{y}_I\right) \end{aligned} \tag{S64}$$

i.e. the shear flow is proportional to a weighted average of mean V_m responses in the E and I units, which, in the SSN, shows linear growth for small α and sublinear growth for larger α (cf. Methods S2 and Figure S1D). Thus, we have a situation in which the flow that boosts variability grows faster initially than those that quench variability, causing a transient increase in total variance for small increasing inputs. For large α, all flows ($|\boldsymbol{\lambda}_s|$, $|\boldsymbol{\lambda}_d|$ and w_{FF}) grow as $\sqrt{\alpha}$ (Figure S2A), because $\mathcal{J}$ is dominated by its $J_{\alpha\beta}\,\overline{y}_\beta$ components and the $\overline{y}$ terms grow as $\sqrt{\alpha}$ as seen in Methods S2. Thus, the total variance in Equation S60 should decay as $1/\alpha$ in this limit, consistent with what we concluded in 3.3.

When the eigenvalues of $\mathcal{J}$ turn complex-conjugate, Equations S61, S63 and S64 above become more complicated expressions, which nevertheless does not change the main insights.

Methods S4 Firing rate and spike count variability

4.1 Generic results in the SSN regime

In Equation S34, we derived a generic scaling of membrane potential variances, $\boldsymbol{\Sigma}$, with mean responses in the SSN. What does it imply for rate variances and Fano factors? Firing rate variability, $\boldsymbol{\Sigma}^r$, is straightforwardly related to voltage variability through a linearization of the input/output nonlinearity, yielding the following relationship:

$$\Sigma^r_{ij} \propto \overline{V}_i^{(n-1)}\,\overline{V}_j^{(n-1)}\,\Sigma_{ij} \approx \mathcal{O}(1) \tag{S65}$$

Therefore, whether $\boldsymbol{\Sigma}^r$ grows or shrinks with increasing activation will depend on parameter details. (Note that this is valid only to the extent that mean responses keep growing with large stimuli, which occurs when $\Omega_E > 0$ – see 3.3 above. For $\Omega_E < 0$ we observe a decline of firing rate variance with increasing stimulus.)

Under the assumption that spikes are emitted according to an inhomogeneous Poisson process with underlying rate given by a threshold-powerlaw nonlinearity, we have shown in Hennequin and Lengyel (2016) that the above-Poisson contribution to Fano factors (FF-1), due to slow voltage variability, scales as

$$\mathrm{FF}_i - 1 \propto \overline{V}_i^{n-2}\,\Sigma_{ii} \tag{S66}$$

Substituting Equation S34 into this, we have that

$$\mathrm{FF}_i - 1 \propto \overline{V}_i^{-n} \tag{S67}$$

Thus, Fano factors are generally expected to decrease (towards a Poisson lower-bound of 1) as long as the stimulus increases mean responses.

4.2 The specific regime of Kanashiro et al. (2017)

Kanashiro et al. (2017) studied a two-population E-I model (analogous to what we analyzed in Fig. 2 of the main text) in which they analyzed conditions for attention to suppress variability and increase stimulus gain. In apparently conflict with our main result that variability suppression should occur generically, they reported very specific conditions for variability quenching. In this section, we relate their model to ours directly to understand the sources of this apparent contradiction.

Kanashiro et al. (2017) studied mean-field dynamics for firing rates $\mathbf{r} = \begin{pmatrix} r_E \\ r_I \end{pmatrix}$ (an 'r-equation') of the form

$$\tau_E \mathbf{T}\,\frac{d}{dt}\mathbf{r} = -\mathbf{r} + \mathbf{f}\left(\mathbf{W}\,\mathbf{r} + c\,\mathbf{g} + a\,\boldsymbol{\mu} + \boldsymbol{\eta}(t)\right) \tag{S68}$$

where boldface small or Greek letters denote two-vectors with an E and I component, the function $\mathbf{f}(\cdot)$ represents applying the function $f_\mathrm{E}(\cdot)$ to the E component and $f_\mathrm{I}(\cdot)$ to the I component, $\boldsymbol{\eta}(t)$ is a zero-mean unit-variance noise, $c\,\mathbf{g}$ represents a stimulus-driven input, of strength c, while $a\,\boldsymbol{\mu}$ represents an attentional input, of strength $0 \leq a \leq 1$.

For the input-output function $\mathbf{f}(\cdot)$, Kanashiro et al. (2017) used the firing rate of an integrate-and-fire neuron responding to a given mean and variance of input. This is an expansive function that, for a fixed level of fast noise, can be well approximated as a power law (Hansel and van Vreeswijk, 2002) (precisely the form of nonlinearity we used in our model (S1). Nevertheless, most of the results we derive in this section (unless otherwise noted) hold for an arbitrary monotonically increasing, expansive $\mathbf{f}(\cdot)$, and thus wherever possible, we will express them in terms of $\mathbf{f}(\cdot)$ and its slope, $\mathbf{f}'(\cdot)$, rather than using our previous approach to express the scaling of variability in terms of $\overline{V}$ and its powers, which was specific to a powerlaw nonlinearity[9].

Although in contrast to Kanashiro et al.'s r-equation, we studied an equation for voltage dynamics (a 'v-equation'; Equation S1), there is a simple equivalence between these two forms of model[10]. In particular, linearizing Equation S68 gives the covariance matrix of rate fluctuations as $\boldsymbol{\Sigma}^\mathrm{r} = \mathbf{F}\,\boldsymbol{\Sigma}\,\mathbf{F}$ where $\boldsymbol{\Sigma}$ is the covariance matrix of voltage fluctuations implied by our (linearized) voltage equation Equation S18 with the same input noise $\boldsymbol{\eta}(t)$, and $\mathbf{F} = \begin{pmatrix} f'_\mathrm{E} & 0 \\ 0 & f'_\mathrm{I} \end{pmatrix}$ (cf. Equation S65).

Like us, Kanashiro et al. (2017) analyzed variability by linearizing the dynamics about a fixed point, and they studied the slow-noise limit; thus we shall also restrict our analysis to this limit here. They concluded that, to reduce variability, attentional input had to be biased toward inhibitory cells ($\mu_\mathrm{I} > \mu_\mathrm{E}$); while for attentional input to increase the gain of response to a stimulus, stimulus-driven input had to be directed to excitatory cells ($g_\mathrm{E} > g_\mathrm{I}$). As we will show, these conclusions depend on the specific, non-generic parameter choices they made, which eliminate the more generic suppression of variability by increasing activity seen in the SSN.

In particular, Kanashiro et al. (2017) simplified the weight matrix $\begin{pmatrix} W_\mathrm{EE} & -W_\mathrm{EI} \\ W_\mathrm{IE} & -W_\mathrm{II} \end{pmatrix}$ to the special, non-generic form $\begin{pmatrix} W_\mathrm{E} & -W_\mathrm{I} \\ W_\mathrm{E} & -W_\mathrm{I} \end{pmatrix}$. This assumption on the weights means that $\mathrm{Det}\,\mathbf{W} = 0$ and $\Omega_\mathrm{E} = \Omega_\mathrm{I} = 0$, which eliminates many SSN behaviors.[11] Furthermore this means that $\mathrm{Det}\,\mathbf{A}$ scales as $(f')^1$ instead of the generic $(f')^2$ (because one of the eigenvalues of $\mathbf{A}$ is -1, independent of the values of the f''s, cf. Footnote 9), so that $\mathbf{A}^{-1}$ scales as $(f')^0$. Therefore, $\boldsymbol{\Sigma}$ scales as $(f')^0$ instead of the generic $(f')^{-2}$, $\boldsymbol{\Sigma}^\mathrm{r}$ scales as $(f')^2$ instead of the generic $(f')^0$ (cf. Footnote 9). To see the implications of this scaling for Fano factors, we recall that the firing rate nonlinearity f used by Kanashiro et al. is well approximated by a threshold powerlaw with some exponent n; thus, from the general results developed in 4.1, we expect the above-Poisson part of the Fano factor, $\propto \overline{V}^{n-2}\,\Sigma$, to scale as $(f')^{(n-2)/(n-1)}$ instead of the generic $(f')^{-\frac{n}{n-1}}$. In short, this choice of parameters causes $\boldsymbol{\Sigma}$ and the Fano factor to lose their generic decrease with increasing activity (and in fact, causes the Fano factor to generically increase instead), and causes $\boldsymbol{\Sigma}^\mathrm{r}$ to change from going to a constant for large f''s to generically increasing with increasing activity. This renders any decrease in these measures of variability with increasing activity much more

[9] For example, it is easy to show that the scaling of effective connectivity, membrane potential and rate variability developed in Equations S34 and S65 can be written using this more general approach as $\mathrm{Det}\,\mathbf{A} \propto (f')^2$, $\boldsymbol{\Sigma} \propto (f')^{-2}$ and $\boldsymbol{\Sigma}^\mathrm{r} \propto (f')^0$, respectively.

[10] When $\mathbf{T}$ (a diagonal matrix of relative time constants) is proportional to the identity matrix, the r-equation $\tau\frac{\mathrm{d}}{\mathrm{d}t}\mathbf{r} = -\mathbf{r} + \mathbf{f}\left(\mathbf{W}\mathbf{r} + \mathbf{h}_r(t)\right)$ is equivalent to the v-equation $\tau\frac{\mathrm{d}}{\mathrm{d}t}\mathbf{v} = -\mathbf{v} + W\mathbf{f}(\mathbf{v}) + \mathbf{h}_v(t)$, under the equivalence $\mathbf{v} = \mathbf{W}\mathbf{r} + \mathbf{h}_r$, $\tau\frac{\mathrm{d}}{\mathrm{d}t}\mathbf{h}_r = -\mathbf{h}_r + \mathbf{h}_v$ (Miller and Fumarola, 2012). For steady states or in the slow noise limit, the rate and voltage equations are equivalent under the simpler relationship $\mathbf{r} = f(\mathbf{v})$, $\mathbf{h}_r = \mathbf{h}_v$, regardless of the structure of $\mathbf{T}$.

[11] The SSN (Ahmadian et al., 2013) relies on a positive determinant of $\mathbf{W}$ to ensure stability and also to ensure that the "loosely balanced" solution exists, which depends on $\mathbf{W}$ being invertible (this solution is illustrated in Equation S11-Equation S15). The loosely balanced solution characterizes SSN dynamics for stronger input (roughly, for stimulus-driven rather than spontaneous input). Furthermore, SSN dynamical regimes are characterized by the nonzero values of Ω_E and Ω_I (Equations S13 and S14).

dependent on specific parameter choices, including in particular the choice of relative strength of external input to E vs. I cells ($\mu_I > \mu_e$).

More specifically, Kanashiro et al. (2017) focused on the variance of r_E (the EE component of $\boldsymbol{\Sigma}^r$). In the general case in the slow noise limit, this is (from Equation S26)

$$\Sigma^r_{EE} = \frac{(1 + f_I' W_{II})((1 + f_I' W_{II})(f_E' c_E)^2 - 2 f_E'^2 f_I' W_{EI} c_{EI}) + (f_E' W_{EI})^2 (f_I' c_I)^2}{(\mathrm{Det}\,\mathbf{A})^2} \tag{S69}$$

where $\mathrm{Det}\,\mathbf{A} = 1 - f_E' W_{EE} + f_I' W_{II} + f_I' f_E' \,\mathrm{Det}\,\mathbf{W}$. In addition to the assumption on the structure of $\mathbf{W}$, Kanashiro et al. (2017) also assumed that the inhibitory and excitatory input noise were perfectly correlated, $c_{EI} = c_E c_I$ (because they assumed a single, global noise process in Equation S68). Using these assumptions, the excitatory rate variance instead becomes

$$\Sigma^r_{EE}\big|_{\mathrm{Kanashiro}} = \frac{f_E'^2 (f_E' c_E + f_I' W_I f_E' c_E - f_I'^2 W_I c_I)^2}{(\mathrm{Det}\,\mathbf{A}|_{\mathrm{Kanashiro}})^2} \tag{S70}$$

where $\mathrm{Det}\,\mathbf{A}|_{\mathrm{Kanashiro}} = 1 - f_E' W_E + f_I' W_I$. The numerator of $\Sigma^r_{EE}|_{\mathrm{Kanashiro}}$ increases with increasing f_E' and decreases with increasing f_I', while the opposite is true of the denominator; this is why their increase in f_I' had to dominate the increase in f_E' for them to find a decrease in variability. For the generic Σ^r_{EE} (Equation S69), there is no such simple monotonic dependence on f_E' or f_I'; while results may be parameter dependent, there is no obvious reason for this generic Σ^r_{EE} why external input must be biased towards I cells in order for increasing activity to suppress excitatory rate variability.

Kanashiro et al. (2017) did, in one figure, consider the effects of a more general weight matrix. They considered the same attention-induced trajectory in r_E and r_I that decreased variability for their restricted weight matrix, and showed that this also decreased variability for a parametric range of weights. However, they did not examine whether their conclusion that variability reduction required $\mu_I > \mu_E$ held in this more general case.

Finally, Kanashiro et al. (2017) considered the attention-induced change in gain of excitatory cells to a stimulus-driven input. The excitatory stimulus gain is $\frac{dr_E^{SS}}{dc}$, where r_E^{SS} is the E-component of the deterministic steady-state value $\mathbf{r}^{SS} = \mathbf{f}\big(\mathbf{W}\,\mathbf{r}^{SS} + c\,\mathbf{g} + a\,\boldsymbol{\mu}\big)$. We can compute $\frac{d\mathbf{r}^{SS}}{dc} = \mathbf{F}\,(\mathbf{W}\,\frac{d\mathbf{r}^{SS}}{dc} + \mathbf{g})$, which can be solved to give $\frac{d\mathbf{r}^{SS}}{dc} = (\mathbf{I} - \mathbf{F}\,\mathbf{W})^{-1}\,\mathbf{F}\,\mathbf{g}$. The E-component is then

$$\frac{dr_E^{SS}}{dc} = \frac{f_E'((1 + f_I' W_{II}) g_E - f_I' W_{EI} g_I)}{\mathrm{Det}\,\mathbf{A}} \tag{S71}$$

We now note that, if we write Σ^*_{EE} for Σ^r_{EE} under the special condition of perfect correlation and equal variances ($c_{EI} = c_E c_I$, and $c_E = c_I$) then the numerator of Σ^*_{EE} is $f_E'^2 c_E^2 (1 + f_I' (W_{II} - W_{EI}))^2$ (the denominator remains $(\mathrm{Det}\,\mathbf{A})^2$). This allows us to write

$$\frac{dr_E^{SS}}{dc} = \frac{g_E}{c_E}\sqrt{\Sigma^*_{EE}} + f_E' f_I' W_{EI} (g_E - g_I)/\mathrm{Det}\,\mathbf{A} \tag{S72}$$

Note that $\mathrm{Det}\,\mathbf{A} > 0$ is a necessary condition for the fixed point to be stable and $f_E' > 0, f_I' > 0$. This means that, if an attentional manipulation lowers Σ^*_{EE}, then in order for it to raise the excitatory stimulus gain it must either be the case that $g_E > g_I$ and the attentional stimulus increases the second term, by increasing $f_E' f_I'/\mathrm{Det}\,\mathbf{A}$, more than the first term decreases; or $g_E < g_I$ and the attentional stimulus decreases the magnitude of the 2nd term, by decreasing $f_E' f_I'/\mathrm{Det}\,\mathbf{A}$, by more than the decrease in the first term. However, an attentional stimulus may raise Σ^*_{EE} while lowering Σ^r_{EE}.

With the assumptions of Kanashiro et al. (2017), Equation S71 becomes $\frac{dr_E^{SS}}{dc} = \frac{f_E'(g_E + W_I f_I'(g_E - g_I))}{\mathrm{Det}\,\mathbf{A}|_{\mathrm{Kanashiro}}}$ and the numerator of Σ^*_{EE} becomes $f_E'^2 c_E^2$. Kanashiro et al. (2017) restricted their analysis to the case $c_I = c_E$, so that with their other assumptions $\Sigma^r_{EE} = \Sigma^*_{EE}$, and so simply wrote

$$\frac{dr_E^{SS}}{dc} = \frac{g_E}{c_E}\sqrt{\Sigma^r_{EE}} + f_E' f_I' W_I (g_E - g_I)/\mathrm{Det}\,\mathbf{A}|_{\mathrm{Kanashiro}} \tag{S73}$$

Note that the numerator of the second term is quadratic in the increasing f' terms, while their denominator (unlike the denominator in Equation S72) is only linear in these terms. Thus, under Kanashiro et al., 2017's assumptions, the second term should generically increase in magnitude with the increased activation induced by attention. Perhaps on this basis, they predicted that attention's observed effects of lowering excitatory variability and raising excitatory stimulus gain required $g_E > g_I$.

Defining $\Sigma^{\Delta}_{EE} \equiv \Sigma^{r}_{EE} - \Sigma^{*}_{EE}$,[12] Equation S72 can be rewritten as

$$\frac{\mathrm{d}r_E^{SS}}{\mathrm{d}c} = \frac{g_E}{c_E}\sqrt{\Sigma^{r}_{EE} - \Sigma^{\Delta}_{EE}} + f_E' f_I' \, W_{EI}\,(g_E - g_I)/\mathrm{Det}\,\mathbf{A} \tag{S76}$$

Note that, with Σ^{r}_{EE} decreasing, the first term of $\frac{\mathrm{d}r_E^{SS}}{\mathrm{d}c}$ can be increasing if Σ^{Δ}_{EE} decreases by more than Σ^{r}_{EE}. Thus, for generic parameters, we conclude that attention can increase excitatory stimulus gain while lowering Σ^{r}_{EE} by virtue of the first term of Equation S76 increasing with attention, which will occur if Σ^{Δ}_{EE} decreases more than Σ^{r}_{EE}, and/or of the second term increasing with attention, which will occur for $g_E > g_I$ or $g_E < g_I$ if $f_E' f_I'/\mathrm{Det}\,\mathbf{A}$ increases or decreases, respectively, with attention.

[12] With the assumptions of Kanashiro et al. (2017),

$$\Sigma^{\Delta}_{EE}\big|_{\text{Kanashiro}} = \left(f_E'^2 f_I' \; W_I \; (c_E - c_I) \; \left(2\,c_E + f_I' \; W_I \; (c_E - c_I)\right)\right) / \left(\mathrm{Det}\,\mathbf{A}\big|_{\text{Kanashiro}}\right)^2 \tag{S74}$$

More generally,

$$\Sigma^{\Delta}_{EE} = \left(f_E'^2 f_I' \; W_{EI} \; \left(2\,(1 + f_I' \; W_{II})\,(c_E^2 - c_{EI}) + f_I' \; W_{EI}\,(c_I^2 - c_E^2)\right)\right) / \left(\mathrm{Det}\,\mathbf{A}\right)^2 \tag{S75}$$

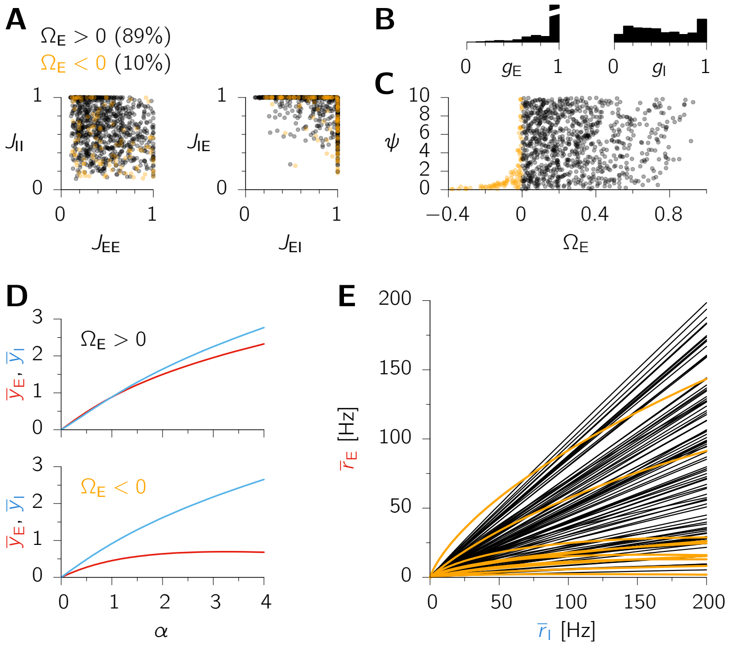

Figure S1. Related to Figure 2. Typical behavior of mean responses to increasing inputs in 1000 randomly sampled 2-population SSNs. (A) Dimensionless recurrent weights $\{J_{\alpha\beta}\}$ (Equation S8); these are normalized such that the largest of the four weights is one for each network. Colors indicate the sign of Ω_E (Equation S13). **(B)** Distribution of feedforward weights g_E and g_I, also normalized for each network so that their maximum is one. **(C)** Overall connection strength ψ (in units of W, see table "Parameters Used in the SSN Simulations" in STAR Methods, such that $W_{\alpha\beta} \equiv \psi J_{\alpha\beta}$) vs. Ω_E. **(D)** Example responses (dimensionless voltages $\bar{y}_E$ and $\bar{y}_I$) to increasing inputs (dimensionless α) for a network with $\Omega_E > 0$ (top) and one with $\Omega_E < 0$ showing supersaturation (bottom). **(E)** Mean E firing rate $\bar{r}_E$ as a function of the mean I firing rate $\bar{r}_I$, for a subset of networks; each point on these curves corresponds to a different input level, increased from zero to a maximum value chosen such that $\bar{r}_I$ = 200 Hz.

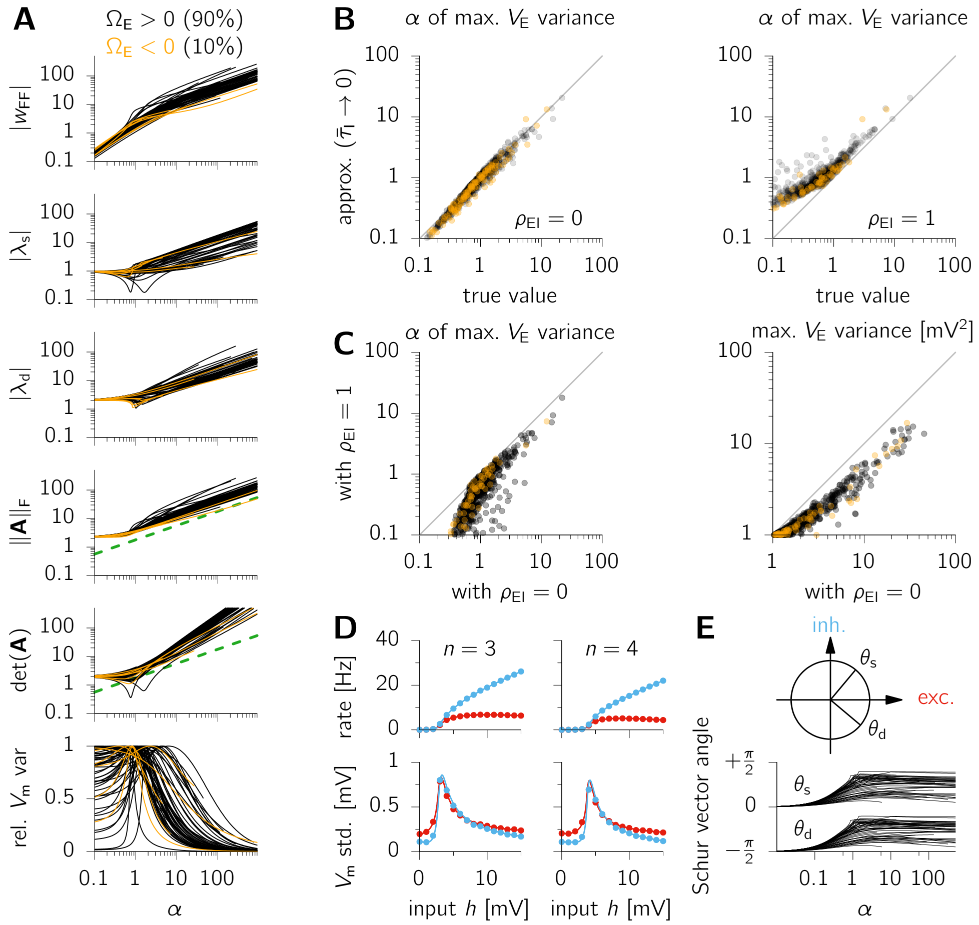

Figure S2. Related to Figure 2. Robustness of variability modulation to changes in network parameters. We examined the modulation of variability by external input in the 1000 randomly parameterized, 2-population networks of Figure S1. **(A)** Behavior of $|w_{\mathrm{FF}}|$, $|\lambda_{\mathrm{s}}|$, $|\lambda_{\mathrm{d}}|$, $\|\mathbf{A}\|_{\mathrm{F}}$, $\det(\mathbf{A})$ (Equation S49) and the total variance (normalized to unit peak), as a function of the (dimensionless) input α. The dashed green line is proportional to $\sqrt{\alpha}$. Only a random subset of the thousand random networks are shown. Following the same convention as in Figure S1, cases with $\Omega_{\mathrm{E}} > 0$ are shown in black, those with $\Omega_{\mathrm{E}} < 0$ in orange. **(B)** Scatter plot of the α at which the E variance reaches its maximum ("true value"), and that given by the approximate criterion of Equation S45 (which assumes very fast inhibition, i.e. $\bar{\tau}_{\mathrm{I}} \to 0$), for uncorrelated (left, $\rho_{\mathrm{EI}} = 0$) and fully correlated (right, $\rho_{\mathrm{EI}} = 1$) input noise term to the E and I units. **(C)** Scatter plot of the input α at which the E variance peaks (left), as well as the value of the variance peak (right), for $\rho_{\mathrm{EI}} = 0$ vs. $\rho_{\mathrm{EI}} = 1$. **(D)** Mean E (red) and I (blue) firing rates (top) and V_{m} std. (bottom) for two example networks with larger values of the power-law exponent n; parameters were otherwise the same as in Figure 2 of the main text. **(E)** Orientation of the two Schur vectors for a subset of the 1000 random networks. Their "sum-like" and "difference-like" nature emerges quite rapidly for small α and then persists for larger α.

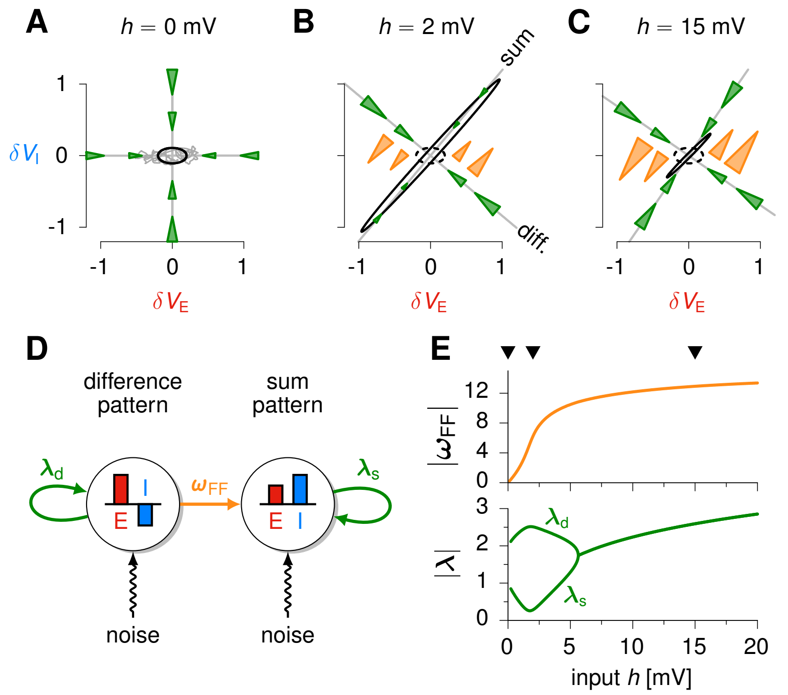

Figure S3. Related to Figure 2. Mechanism of input-dependent modulation of variability in the SSN. (A–C) Visualization of the influence of single-neuron leak and effective connectivity as restoring and shear flow fields shaping the (co-)variability of E/I activity in the two-population SSN. Cardinal axes (δV_E and δV_I) measure (in mV) the deviation of E and I membrane potentials from their respective steady-state values (Equation S18) defined such that the origin ($\delta V = 0$ mV) corresponds to the stationary mean population activity for the given input strength h (labels on top). Gray axes show directions of Schur vectors (Equation S49) along which the restoring flow field acts (green triangular arrows) and which are also coupled by the shear flow field (w_{FF}, orange triangular arrows) such that deviations along the "difference" axis give rise to deviations along the "sum" axis. Triangular arrows are proportional in area to the contribution they make to the total flow of fluctuations. Gray traces show example membrane potential fluctuations of the network, black covariance ellipses show contour lines of the corresponding joint distribution of δV_E and δV_I at one standard deviation, dashed ellipses in (B) and (C) reproduce covariance ellipse at $h = 0$ mV (A) for comparison. At $h = 0$ **(A)**, the only contributor to the flow of trajectories is the leak in each population (green flow field) acting along the cardinal axes of E/I fluctuations – the flow is stronger (suppresses fluctuations more) along the I axis due to the shorter membrane time constant in I cells. This flow contains the diffusion due to input noise (cf. example trajectory in gray), resulting in uncorrelated baseline E/I fluctuations (black ellipse is axis aligned). As the network is driven by $h > 0$ **(B–C)**, the effective recurrent connectivity adds to the leak to instate two types of flow fields steering fluctuations: a restoring flow field (green, generalizing the leak in (A)) and a "shear-like" flow field (orange). The relative contributions of the two flow fields determine the size and elongation of the E/I covariance (solid black ellipses). **(D)** Illustration of the decomposition of the effective connectivity (for a given mean stimulus h; Equation S49) as couplings between a difference-like pattern (left) and a sum-like pattern (right; cf. rotated gray axes in B-C). The difference mode feeds the sum mode with weight w_{FF} (orange arrow), and the difference and sum patterns inhibit themselves with negative weight λ_d and λ_s respectively (green arrows). These three h-dependent couplings scale the corresponding flow fields in (A-C) (consistent colors). **(E)** Input-dependence of w_{FF} (top, orange) and $|\lambda_d|$ and $|\lambda_s|$ (bottom, green). Black triangular marks indicate input levels illustrated in (A-C).

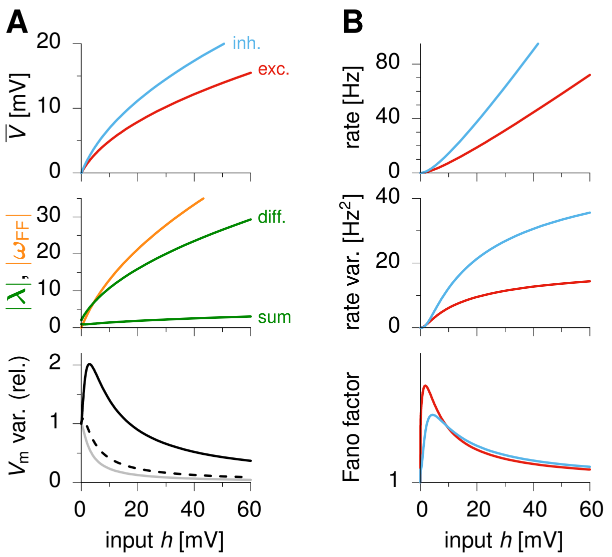

Figure S4. Related to Figure 2. Variability modulation cannot be understood based on the restoring flows (i.e. the eigenvalues of the Jacobian of the dynamics) alone. (A) Example 2-population network showing transient increase in variability with increasing external input h (bottom, black, normalized to variance at $h = 0$ mV), *without* any substantial decrease in any of the eigenvalues, and in $|\boldsymbol{\lambda}_s|$ in particular (middle, green; cf. Figure S3E, bottom). The dashed black curve (bottom) shows the predicted variability (Equation S60) assuming $\mathbf{w}_{FF} = 0$ uniformly (cf. middle, orange), i.e. taking into account only the magnitude of the restoring flows $\boldsymbol{\lambda}_d$ and $\boldsymbol{\lambda}_s$ (middle, green). The gray curve (bottom) is the prediction made by assuming fully correlated input noise terms with variance g_E^2 and g_I^2 respectively for the E and I units. Variability in this case can be read off from the slope of the $\overline{V}_E$ (top, red) and $\overline{V}_I$ curves (top, blue), because input noise becomes equivalent to fluctuations in h to which the network has time to respond. Neither of these two predictions capture the initial growth of variability and, consequently, both grossly underestimate the overall magnitude of variability across the whole range of inputs. **(B)** Mean firing rates (top), variances of firing rate fluctuations (middle) and Fano factors (assuming Poisson spike emission on top of rate fluctuations), in the same network as in (A) for the E (red) and I populations (blue). Note that the overall scale of super-Poisson variability (Fano factor minus one) is arbitrary here, and in general depends on the counting window, autocorrelation time constants, and the variance of the input noise. Parameters: $\tau_\eta \to \infty$, $g_E = 0.77$, $g_I = 1$, $J_{EE} = 0.38$, $J_{EI} = 0.27$, $J_{IE} = 1$, $J_{II} = 0.6$, $\psi = 2.37$.

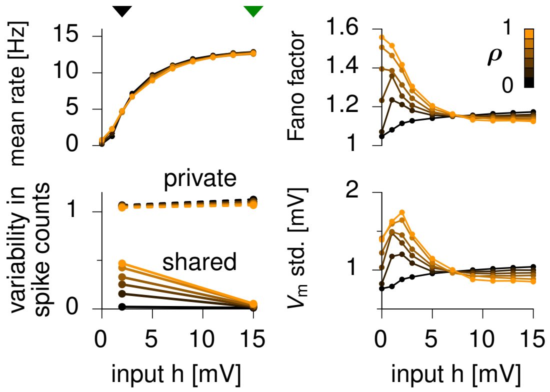

Figure S5. Related to Figure 3. Input correlation-dependent behavior of the spiking SSN. Top left: mean population firing rates (top left) as a function of input strength *h*, and for different values of the input correlation ρ (color coded). Triangular marks denote the values of *h* used in spontaneous (black) and evoked (green) conditions in Figure 3. **Top right**: Fano factors (population average $\pm$ std.) **Bottom right**: V_{m} std. (population average $\pm$ std.) **Bottom left**: factor analysis applied to normalized spike counts (such that the total variance equals the average Fano factor; see STAR Methods) to decompose variability into a shared component (one single factor), and a private component (Churchland et al., 2010). Note that only the shared part of variability is quenched by increasing stimulus, and that shared variability and its quenching both require a non-zero input correlation coefficient ρ.

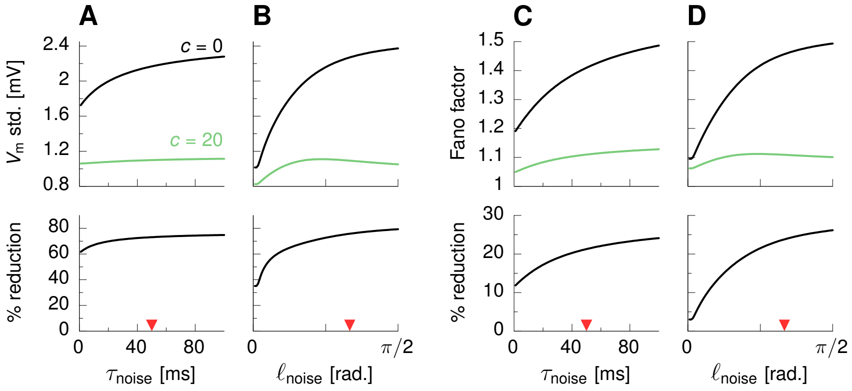

Figure S6. Related to Figure 4. Dependence of variability reduction in the ring SSN model on spatial and temporal correlations in the input noise. Dependence of the network-averaged V_m std. **(A-B)** and Fano factor **(C-D)** on either the temporal correlation time constant τ_{noise} in the external input noise term (for fixed $\ell_{noise} = 60°$) **(A, C)**, or its spatial correlation length ℓ_{noise} (for fixed $\tau_{noise} = 50$ ms) **(B, D)**, in the spontaneous ($c = 0$, black) and high-contrast ($c = 20$, green) input regimes. Red arrows indicate the parameter values used in the main text (see table "Parameters Used in the SSN Simulations" in STAR Methods). Top panels show absolute magnitude of variability, bottom panels show the amount of relative variability suppression for the high contrast input, as a percentage of spontaneous variability.

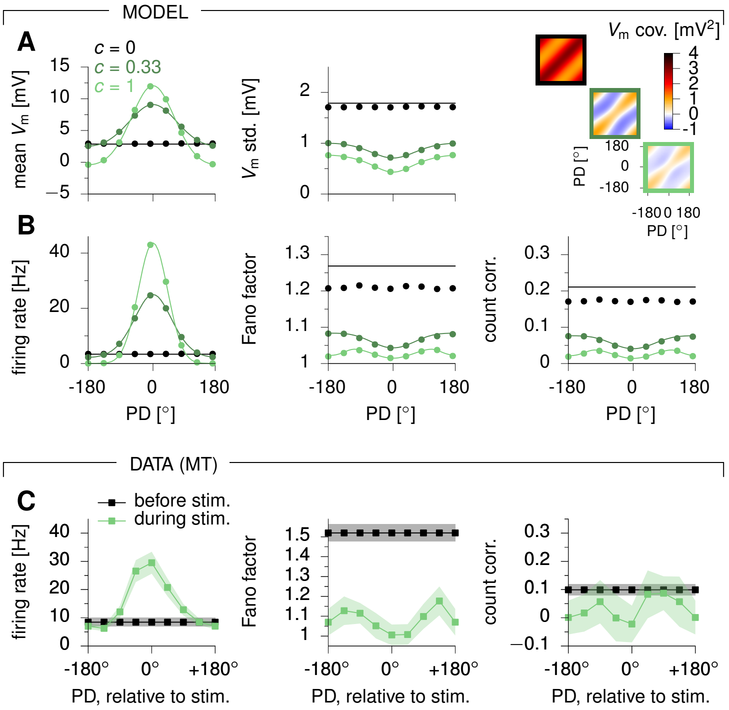

Figure S7. Related to Figure 4. A ring SSN accounts for the stimulus dependence of across-trial variability in area MT. (A) V_m mean (left) and std. (center) as a function of the model neuron's preferred direction (PD, relative to stimulus at 0°), for increasing values of stimulus strength *c*. The full V_m covariance matrices are shown on the right for the E population, box color indicating *c*. **(B)** Mean firing rates (left), spike count Fano factors (center), and spike count correlations between similarly tuned neurons (right), as a function of the neurons' (mean) preferred direction. **(C)** Experimental data (awake monkey MT) adapted from (Ponce-Alvarez et al., 2013), with average firing rates (left), average Fano factors (center), and average spike count correlations among similarly tuned cells (right), as a function of the cells' preferred direction. Data is shown for spontaneous (pre-stimulus, black) and evoked (high-contrast stimulus, green) activity periods. Error bars denote s.e.m. Dots in panels A–B were obtained from 400 s epochs of simulated stationary activity, and denote averages among cells with similar tuning preferences (PD difference$<$ 18°); solid lines show analytical approximations (Hennequin and Lengyel, 2016). In panels B-C, spikes were counted in 100 ms bins. The only parameters that differed from Figure 4 of the main text were: $\ell_{syn} = \ell_{noise} = \ell_{stim} = 80°$ (see table "Parameters Used in the SSN simulations" in STAR Methods).

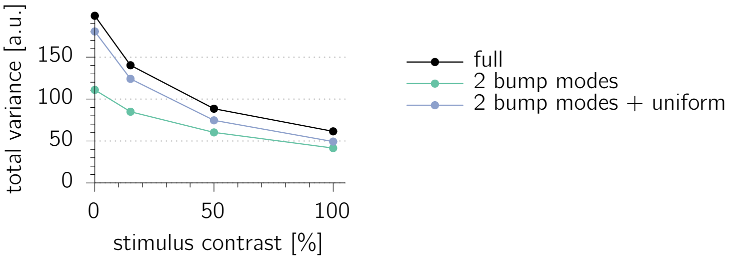

Figure S8. Related to Figure 5. Bump kinetics capture a substantial amount of variability in the ring SSN model. Black: total V_m variance in the ring SSN (E neurons) as a function of stimulus contrast. This is compared to the total variance captured by the two main modes of bump kinetics (green), and by a basis of 3 vectors formed by the same two modes + the uniform mode orthogonalized against the other two (blue). This three-dimensional subspace is virtually identical to the subspace spanned by the top three principal components of V_m fluctuations, at all stimulus contrasts, but yields a more interpretable basis. Note that while a substantial fraction of variability *suppression* with increasing stimulus contrast is due to quenched fluctuations in the uniform mode (difference between blue and green curves), the two modes of bump kinetics alone capture most of the variance at high contrast. Also note that the amount of variance captured by these linear projections is slightly smaller than that captured by the full, nonlinear fit shown in Figure 5A.

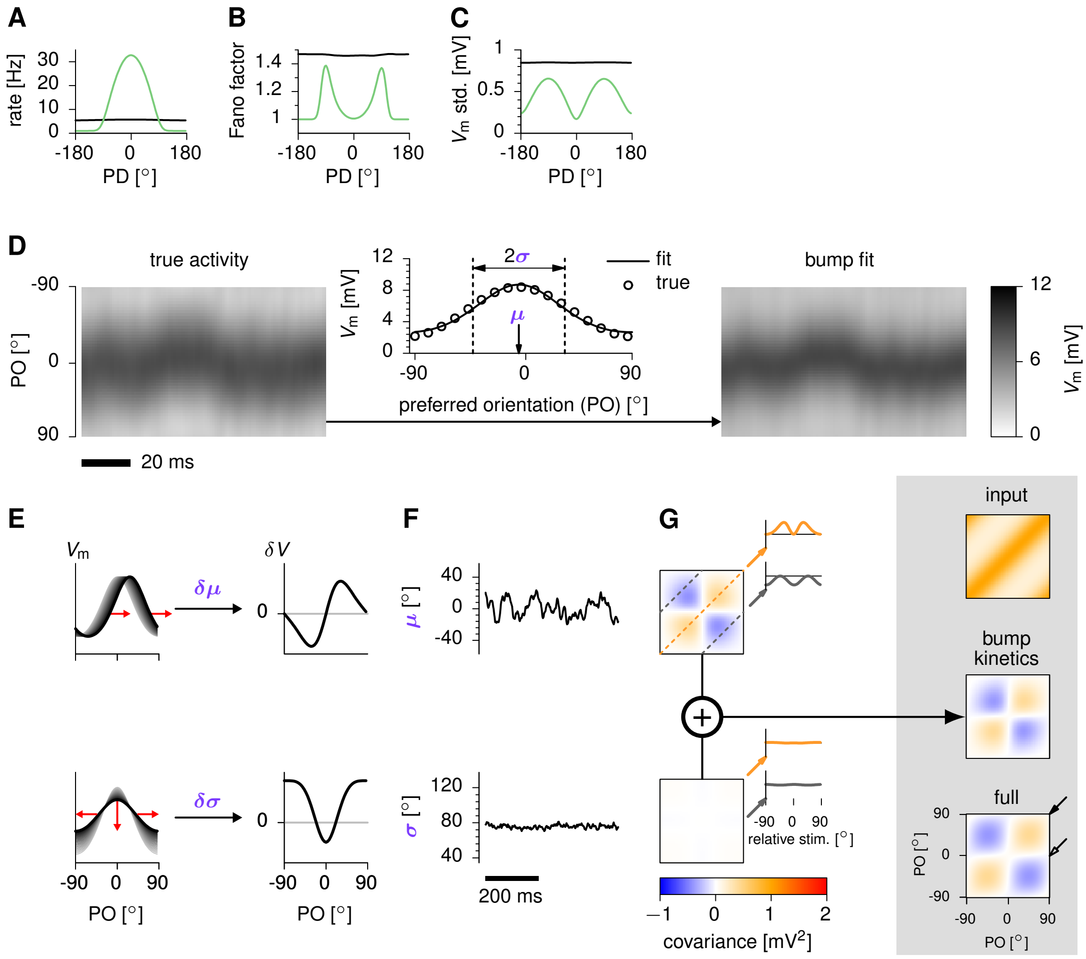

Figure S9. Related to Figures 5-6. Activity variability in a ring *multi-attractor* network. (**A–C**) Tuning of mean firing rates, Fano factors, and V_m std. in spontaneous (c = 0, black) and evoked (c = 3, green) conditions. (**D–G**) Analogous to Figure 5A-D, for the ring *attractor* network. By a large margin, the dominant contributor to activity variability in this network for a strong stimulus is the sideways jittering of the activity bump (E-G, top), with an almost complete absence of variability in the width of the bump (E-G, bottom).

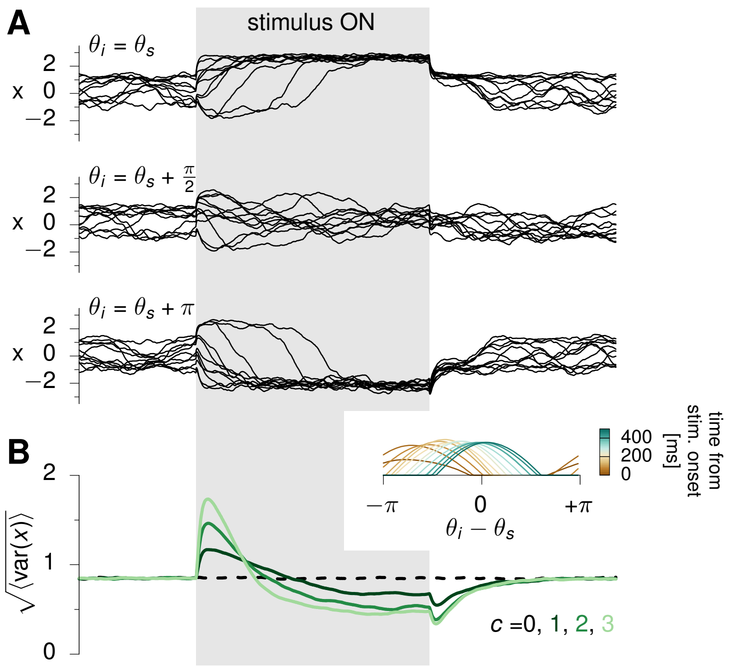

Figure S10. Related to Figure 7. Dynamics of variability quenching in the ring multi-attractor model. (A) Sample membrane potentials (10 trials, individual lines showing V_i in Equation 13; STAR Methods) for a neuron tuned to the stimulus direction (top), to the orthogonal direction (middle) and to the opposite direction (bottom). Here the stimulus, θ_s, and thus also the preferred stimuli of neurons, θ_i, are defined to be between $-\pi$ and π. Stimulus strength is stepped up for a 1-sec duration (gray shading; $c = 2$). **(B)** Time course of the standard-deviation across trials of the membrane potential, averaged across neurons, for different values of input strength, c (color coded). The inset shows the spatial profile of network activity (firing rate r, Equation 14; STAR Methods) in an example trial over 400 ms following stimulus onset (time is color coded). First, the activity bump quickly scales up and then it slowly moves from its initial random location (here, around $-3\pi/4$) to the new position determined by the stimulus (at $\theta_s = 0$). The initial growth of bump amplitude increases variability because of the random location of the bump across trials, while the slow movement to a location that is the same across trials decreases variability.

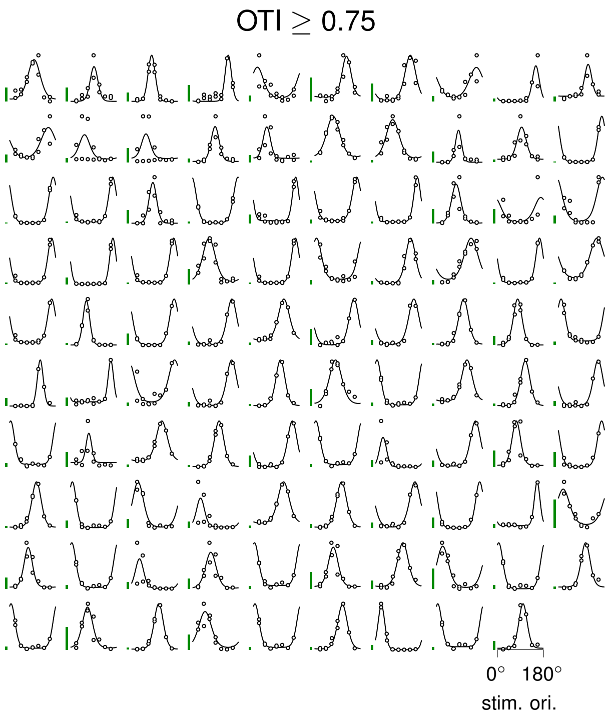

Figure S11. Related to Figures 4, 6, and 7. Tuning curves of V1 cells analysed from the data set of Ecker et al. (2010). Only cells with an orientation tuning index (OTI) of at least 0.75 are shown here and were included in subsequent analyses (STAR Methods). Green vertical scale bars: 2 spikes/sec. Note that some cells were also direction selective, hence responded at two different levels at some orientations depending on the motion direction.

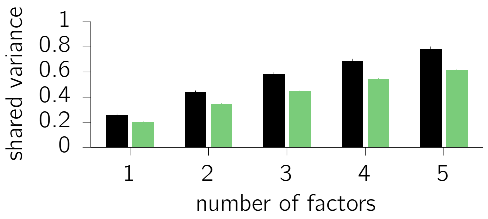

Figure S12. Related to Figure 4. Parameter-dependence of shared variability suppression as measured by factor analysis. Reduction of shared variability from spontaneous (black) to stimulus-evoked (green) activity in the monkey V1 dataset (Ecker et al., 2010), as estimated via factor analysis (STAR Methods). The x-axis shows the number of latent factors used. Only conditions with at least 8 simultaneously recorded well-isolated cells were analyzed (151 conditions).
